# Fe(porphyrin)-Catalyzed Alkene Epoxidation with NaOCl: A Practical Small- and Large-Scale Alternative to *m*CPBA

**DOI:** 10.1021/jacs.6c06785

**Published:** 2026-05-26

**Authors:** Sudip Maiti, Doohyun Baek, Shannon S. Stahl

**Affiliations:** † Department of Chemistry, 5228 University of Wisconsin-Madison, Madison, Wisconsin 53706, United States; ‡ Wisconsin Energy Institute, University of Wisconsin-Madison, Madison, Wisconsin 53726, United States

## Abstract

Epoxides are important intermediates in synthetic chemistry. Stoichiometric peroxyacids, such as *meta*-chloroperoxybenzoic acid (*m*CPBA), are widely used to convert alkenes to epoxides but show poor compatibility with aromatic heterocycles and present hazards when scaled. Herein, we report a highly practical alkene epoxidation method that uses the commercially available iron porphyrin, Fe­(TPFPP)­Cl (TPFPP = tetrakis­(pentafluorophenyl)­porphyrin), as a catalyst (0.05 mol %) and aqueous NaOCl as the oxidant in acetonitrile as the solvent. No additional ligands or additives are needed, and the reactions proceed under ambient conditions. The method shows a broad scope, affording high yields of epoxides in reactions with terminal and internal aromatic and aliphatic alkenes, heterocycle-containing substrates, glycals, and polyenes. The practicality of the method is demonstrated in the 100 g scale epoxidation of tri-*O*-acetyl-*D*-glucal, which proceeds to completion in 15 min at room temperature.

## Graphical abstract

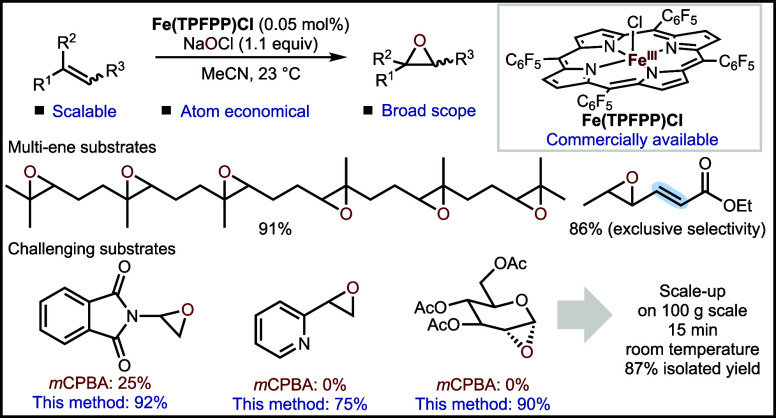

Epoxides are versatile intermediates in chemical synthesis, from pharmaceuticals to commodity chemicals.^1,2^ The most common route to epoxides involves oxygen-atom transfer (OAT) to alkenes using stoichiometric oxidants such as *meta*-chloroperoxybenzoic acid (*m*CPBA)^3^ and dimethyl dioxirane (DMDO) (Figure 1a).^4,5^ These reagents are suitable for laboratory-scale reactions, but safety risks and poor atom economy limit large-scale applications.^6,7^ Accordingly, catalytic methods have been developed for alkene epoxidation that employ oxidants, such as hydrogen peroxide and sodium hypochlorite,^8–11^ which have low cost, high active-oxygen content, and generate benign byproducts (H_2_O and NaCl). Four prominent catalyst systems compatible with such oxidants include Mn^III^(TPP)­OAc (TPP = tetraphenylporphyrin) with NaOCl,^12^ Mn^III^(Salen)Cl (Salen = (*R*,*R*)-(−)-*N*,*N*’-bis­(3,5-di-*tert*-butylsalicylidene)-1,2-cyclohexanediamine) with NaOCl,^13,14^ Na_2_WO_4_ and a phase-transfer catalyst with H_2_O_2_,^15^ and Mn­(ClO_4_)_2_/2-picolinic acid/butanedione with H_2_O_2_ (Figure 1b).^16^ These catalysts can be very effective, but none exhibits the synthetic scope of *m*CPBA. Herein, we report a highly practical Fe­(TPFPP)Cl (TPFPP = 5,10,15,20-tetrakis­(pentafluorophenyl)­porphyrin) catalyst for alkene epoxidation with NaOCl. The catalyst is commercially available, operates at very low catalyst loading (0.05 mol %) at room temperature in acetonitrile, and does not require additional ligands or cocatalysts (Figure 1c). The method features a broad scope, including many substrates that are not compatible with other epoxidation methods.

**Figure 1:**
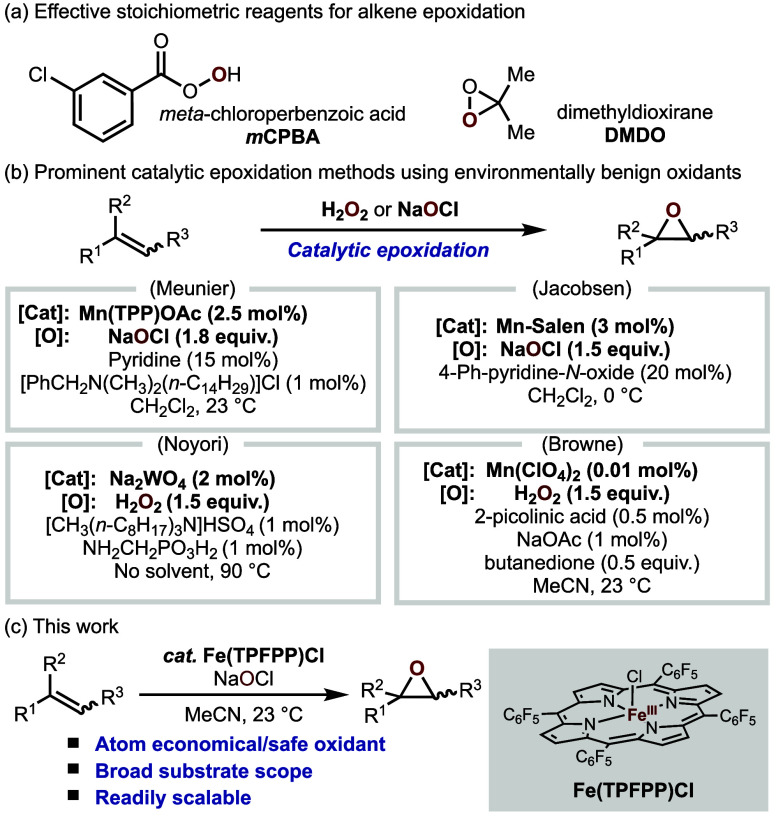
Overview of alkene epoxidation strategies, including (a) widely used stoichiometric epoxidation reagents and (b) prominent catalytic epoxidation methods that use H_2_O_2_ or NaOCl as the oxidant. (c) Fe­(TPFPP)­Cl-catalyzed alkene epoxidation method developed herein, using NaOCl. TPP = tetraphenylporphyrin, TPFPP = 5,10,15,20-tetrakis­(pentafluorophenyl)­porphyrin.

Terminal alkenes can present reactivity challenges for catalytic epoxidations, and 4-phenyl-1-butene (**1a**) was selected to evaluate the different epoxidation methods (Figure 2a). This substrate underwent epoxidation in near-quantitative yield with *m*CPBA (method I) and 66% yield with DMDO generated *in situ* from acetone and oxone (method II; note: higher yields with DMDO would be expected from freshly distilled DMDO^5^). Inferior results relative to those observed with *m*CPBA were obtained under the four catalytic conditions, Methods III–VI (Figure 2a).^17^ These observations prompted us to evaluate a series of other metalloporphyrins and related complexes as catalysts with NaOCl as the oxidant in MeCN (Figure 2b). Negligible epoxide was observed in the majority of cases, but Fe(TPFPP)Cl was unusually effective, affording a nearly quantitative yield of epoxide **2a**. MeCN appears to be a particularly effective solvent, likely reflecting its miscibility with water. For example, no epoxide was observed in CH_2_Cl_2_ or EtOAc, unless 5 mol % of benzyldimethyltetradecylammonium chloride was included as a phase-transfer catalyst, in which case the yield nearly matched that obtained in MeCN (Table S1).

**Figure 2:**
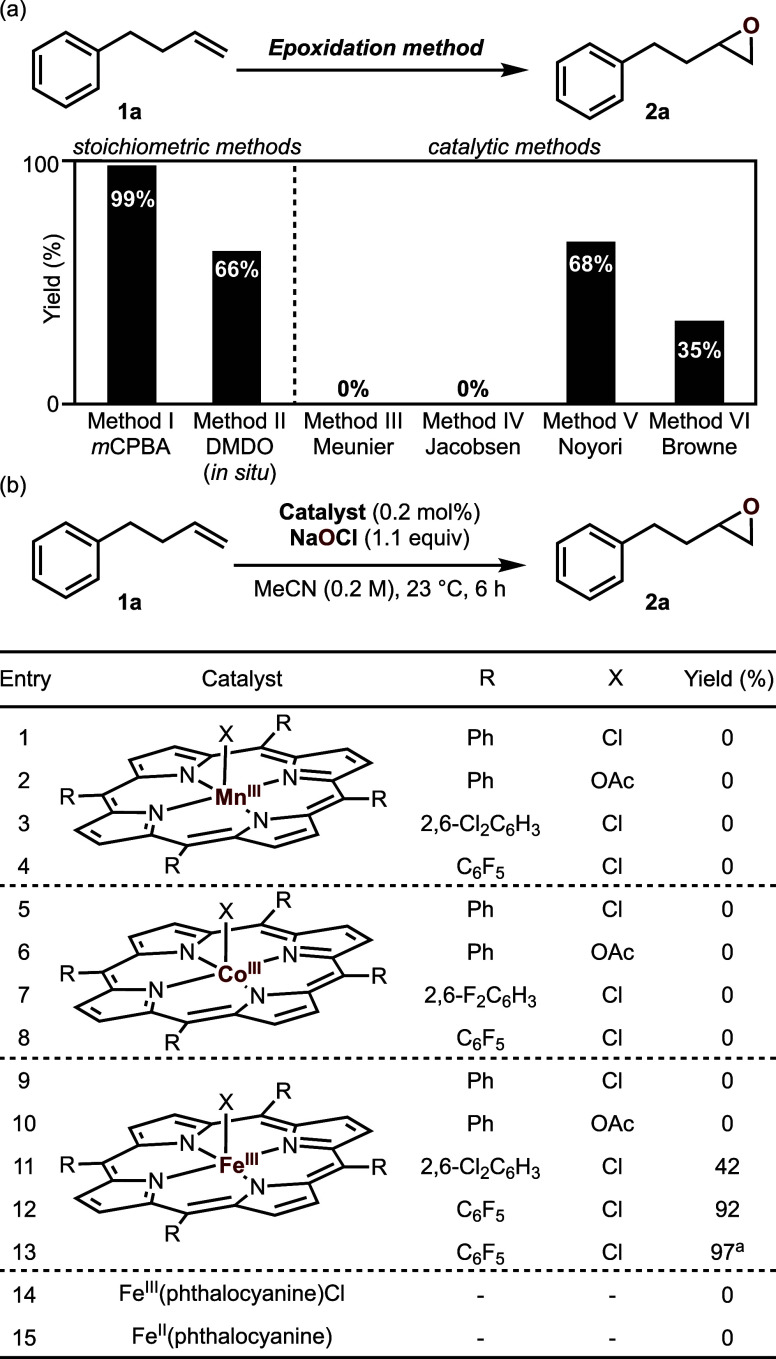
(a) Evaluation of six representative epoxidation methods using 4-phenyl-1-butene (**1a**) as the substrate. Yields based on ^1^H NMR analysis (ext. std. = CH_2_Br_2_; details provided in the Section 2e of Supporting Information). (b) Testing of metalloporphyrin catalysts for epoxidation of **1a** with NaOCl. ^a^The reaction was conducted at a concentration of 0.4 M.

The favorable reactivity of the catalyst prompted us to assess its reactivity in the epoxidation of other alkenes. Twelve alkenes were selected for evaluation, including simple aliphatic terminal alkenes (**1a**, **1b**), internal alkenes with defined *cis*/*trans* geometry (**1c**–**1e**), and oxygen- or nitrogen-containing functional groups (**1e**–**1l**) (Figure 3a). Eight different epoxidation conditions were included in this survey. Methods I–VI noted above were supplemented with a Mn-based catalyst system reported by Stack and co-workers that uses neutralized peracetic acid as the oxidant (Method VII).^18^ Although a peracid oxidant is suboptimal for large-scale applications, this method was reported to have very good substrate scope. The Fe­(TPFPP)Cl catalyst system was included as Method VIII.

**Figure 3:**
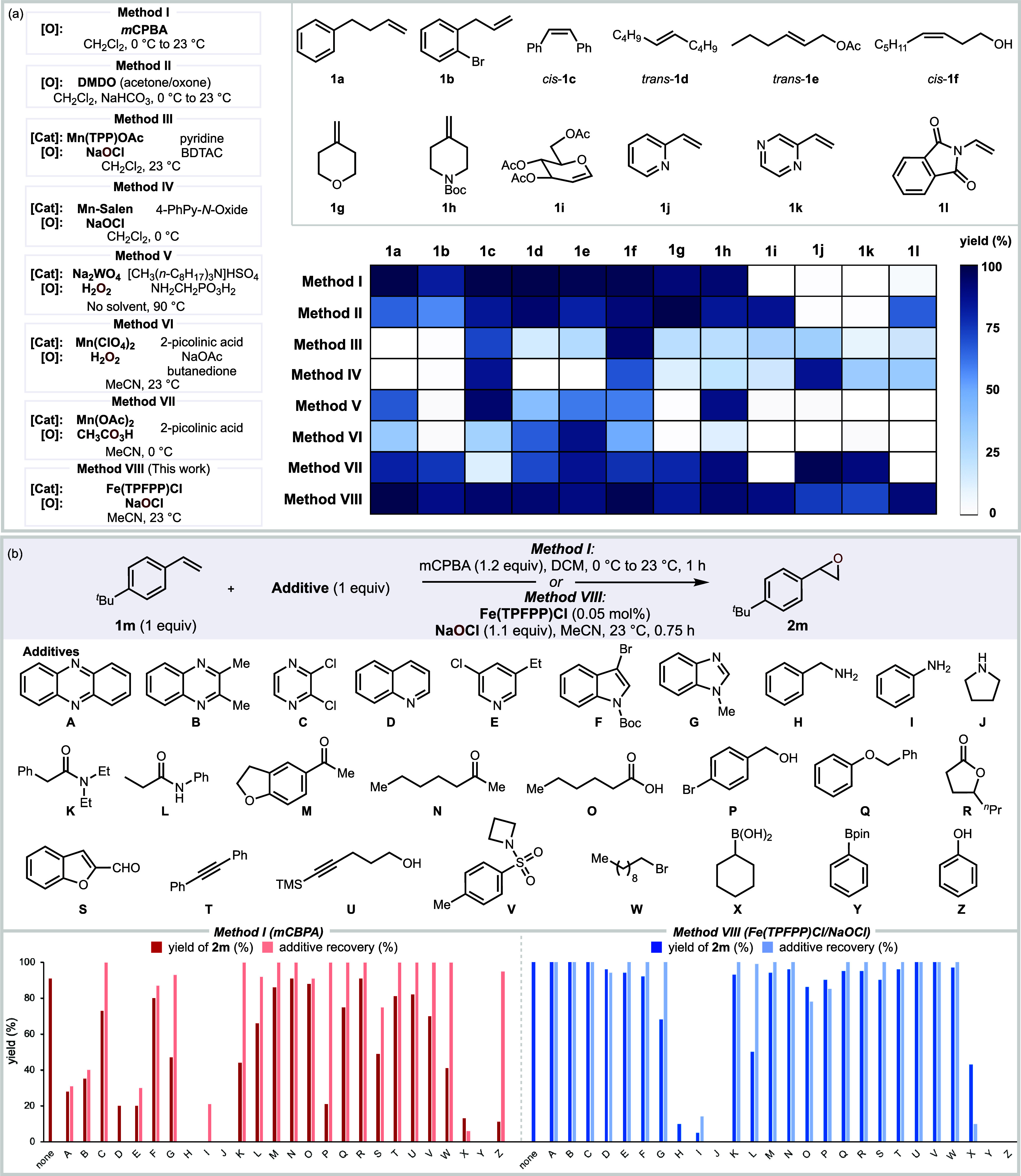
(a) Library of alkene substrates (**1a**-**1l**) for generality test using the conditions noted (method I–VIII). Reaction conditions: Method I–V: same conditions as noted in Figure 2a with corresponding alkenes. See Supporting Information for the reaction details of Method I–VIII. (b) Comparative robustness evaluation of the *m*CPBA epoxidation and Fe­(TPFPP)­Cl/NaOCl-catalyzed epoxidation under various additive conditions. Reaction condition of method I: 4-*tert*-butylstyrene (0.2 mmol), additive (0.2 mmol), *m*CPBA (1.2 equiv) in CH_2_Cl_2_ (1 mL) at 0−23 °C for 1 h. Reaction condition of method VIII: 4-*tert*-butylstyrene (0.2 mmol), additive (0.2 mmol), Fe­(TPFPP)Cl (0.05 mol %), NaOCl (1.1 equiv) in MeCN (1 mL) at 23 °C for 45 min.

Stoichiometric *m*CPBA (Method I) provided high yields of epoxides with eight of the 12 substrates, with the exceptions being glycal **1i**, vinyl heteroarenes **1j** and **1k**, and vinyl imide **1l.** The *in situ* generated DMDO method (Method II) generally gave slightly lower yields than *m*CPBA overall; however, it exhibited a broader substrate scope, successfully converting substrates such as **1i** and **1l** that were not efficiently transformed under the *m*CPBA conditions. Methods III–VI exhibited only limited effectiveness with the 12 substrates. Moderate-to-good reactivity was observed in isolated cases, but many substrates underwent poor conversion and afforded low or negligible yields of epoxide.^17^ Among the literature protocols, the Mn/peracetic acid conditions of Method VII demonstrated the broadest scope, leading to good yields for nine of the 12 substrates. In contrast, Method VIII provided good-to-excellent yields across the entire set of substrates, including **1i** and **1l**, which were ineffective with any of the other protocols other than Method II. With the exception of the two terminal alkyl olefins **1a** and **1b**, which used the originally optimized 0.2 mol % loading of Fe­(TPFPP)­Cl, only 0.05 mol % catalyst was needed to achieve these results.

A ″robustness screen″^19^ was then used to assess the versatility of the Fe­(TPFPP)­Cl/NaOCl conditions relative to *m*CPBA, using 4-*tert*-butylstyrene **1m** as the substrate in the presence of additives bearing diverse functional groups (Figure 3b). The assay quantified the yield of styrene oxide **2m** and the recovery of unreacted additive (dark and faded bars, respectively, in Figure 3b), and the results highlight the merits of the Fe­(TPFPP)­Cl/NaOCl epoxidation method. The majority of reactions with this catalytic method (19 of the 26 reactions) proceeded in high yield with a high additive recovery. Problematic groups included free amines (H, I, and J), boronic acid derivatives (X and Y), and phenol (Z). The reactions with *m*CPBA showed mixed outcomes, with only 10 of the 26 reactions retaining a high yield. Aromatic nitrogen heterocycles were problematic, often resulting in diminished product formation and a poor additive recovery. Free amines (H, I, J), boronic acid derivatives (X, Y), and phenol (Z) were also problematic under the *m*CPBA conditions.^20,21^ These results show that Fe­(TPFPP)­Cl/NaOCl oxidation conditions offer synthetic advantages over *m*CPBA, in addition to overcoming the safety and waste complications of *m*CPBA relevant to larger-scale applications.

We then evaluated additional substrates with this catalytic method, using a catalyst loading of 0.05 mol %, 0.2 M substrate in MeCN, and 1.1 equiv of NaOCl (0.22 mmol of OCl^–^, titrated prior to use; see Section 2b of Supporting Information for details) (Figure 4). The reactions were performed in a capped vial under ambient conditions, with no need to exclude air. Reactions with vinyl (hetero)­arenes afforded excellent epoxide product yields, typically >90%, with negligible impact of electronically varied substituents (**2m**, **4a**-**4i**). Both 2- and 3-vinylpyridine underwent efficient epoxidation (75% and 78% yield of **2j** and **4j**, respectively), without any evidence of *N*-oxide side-product formation. β-Substituted styrene derivatives also furnished the corresponding epoxides in excellent yield (**2c**, **4k**-**4n**, 91–96%) with complete retention of the alkene stereochemistry in the product. Compatible β-substituents included aryl, methyl, chloromethyl, and acetoxymethyl groups.

**Figure 4:**
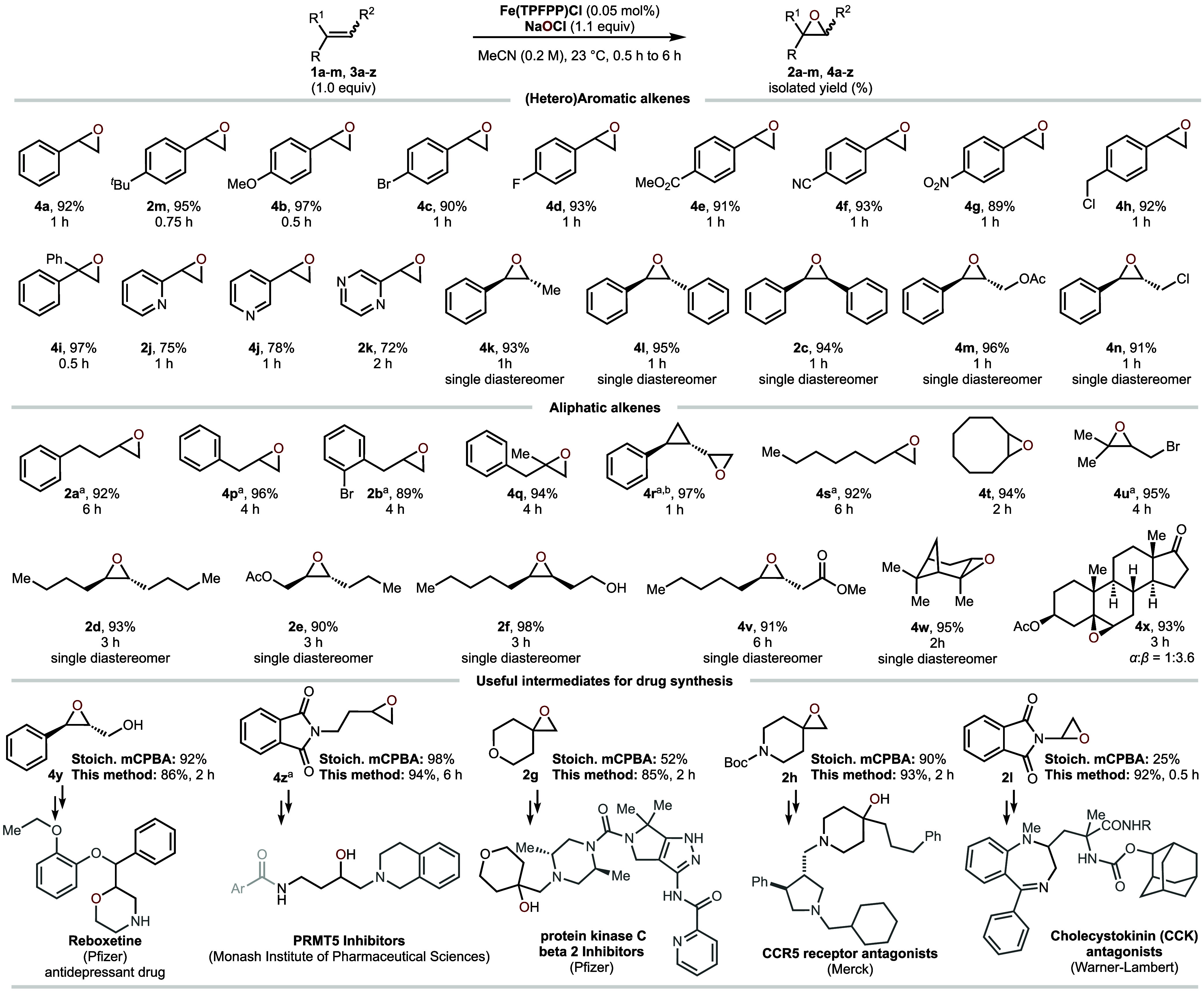
Scope of aromatic alkenes, aliphatic alkenes, and epoxide intermediates for pharmaceuticals. Reaction condition: Alkene (0.2 mmol), Fe­(TPFPP)­Cl (0.05 mol %), NaOCl (1.1 equiv) in MeCN (1 mL) at 23 °C for indicated time. Diastereoselectivity was determined by ^1^H NMR analysis of the crude reaction mixture. Where relevant, diastereomers are present as racemic mixtures. ^a^0.2 mol % of Fe­(TPFPP)Cl was used with higher concentration (0.4 M). ^b^1:1 disastereomer at the epoxide site (*trans* configuration retained at the cyclopropane).

Alkenes with aliphatic substituents beyond the initial test substrate **2a** undergo effective epoxidation. These substrates are less reactive than the styrene derivatives, and some benefit from the use of higher catalyst loading (0.2 mol %). Successful examples include allyl benzene derivatives (**2b**, **4p**–**q**: 89–96% yield). The cyclopropane-containing substrate **4r** is notable in that it forms the epoxide (97% yield) without any formation of ring-opened products. This observation implicates a concerted, rather than stepwise, OAT mechanism. The mono-, di-, and trisubstituted alkenes 1-octene, cyclooctene, and prenyl bromide afforded the corresponding epoxides, **4s**–**u**, in 92%, 94%, and 95% yield, respectively. Similar to the reactions with vinyl arenes, *cis*- and *trans*-disubstituted alkenes undergo epoxidation with no isomerization of the substituents. Examples include epoxides derived from *trans*-decene (**1d**, 93% yield) and *cis*- and *trans*-alkenes containing acetoxy (**1e**), free hydroxy (**1f**), and methyl ester substituents (**3v**) (90–98% yield). The protocol further proved applicable to more complex molecules, including the bicyclic monoterpene α-pinene (**4w**, 95% yield, single diastereomer) and dehydro­isoandrosterone 3-acetate (**4x**, 93% yield, 3.6:1 diastereomeric ratio). Among substrates tested, several alkenes with allylic substituents proved challenging (see Section 2h in the Supporting Information). Some substrates featured problematic functional groups (sulfide, 2° amine, 2° amide), but an allylic ether and carbonate also proved problematic.

Subsequently, we targeted epoxides that have been used to access drug molecules and related bioactive molecules, many of which have been prepared by reaction of an alkene with *m*CPBA. 3-Phenylglycidol (**4y**), an intermediate in the synthesis of the antidepressant reboxetine,^22^ was obtained in 86% yield, with no overoxidation of the hydroxymethyl group to the corresponding aldehyde or carboxylic acid. The reported yield with stoichiometric *m*CPBA is 92%.^23^ A phthalimido-substituted butene underwent epoxidation in 94% yield to afford epoxide **4z**, which has been used to access amino alcohol enzyme inhibitors.^24^ Epoxide **2g**, an intermediate toward protein kinase C beta 2 inhibitors, was obtained in 85% yield, substantially higher than the previously reported yield of 52%, obtained with stoichiometric *m*CPBA.^25^ Epoxide **2h**, which has been used to prepare CCR5 receptor antagonists,^26^ was prepared in 93% yield, comparable to the outcome with *m*CPBA (90%).^27^ Finally, the enimide-derived epoxide **2l** was obtained in 92% yield with the Fe­(TPFPP)­Cl/NaOCl catalyst system, significantly outperforming epoxidation with stoichiometric *m*CPBA (25% yield; note: this higher *m*CPBA yield relative to the 5% yield observed in Figure 3a was achieved through independent optimization of the *m*CPBA reaction). Epoxide **2l** has been used as an amino alcohol building block for cholecystokinin (CCK) antagonists.^28^

Evaluation of the epoxidation method with diene and polyene substrates showed that the reaction has considerable ability to undergo selective oxidation. For example, when using 0.05 mol % Fe­(TPFPP)Cl with 1.1 equiv of NaOCl, the monoepoxides **6a**–**6e** form with high-to-exclusive selectivity (6:1 – ≥20:1) and in good-to-excellent yields (71–90%, Figure 5a). Ethyl sorbate **5a** underwent exclusive monoepoxidation at the internal alkene distal from the electron-withdrawing ester fragment (**6a**, 86% yield). The two alkenes in 1,5-cyclooctadiene are separated by two methylene units, yet epoxidation could still proced in 71% yield with 8:1 selectivity for monoepoxide **6b**. This compound has been widely used in the synthesis of bioactive molecule, such as acetogenins,^29^ and in polymer chemistry.^30–32^ The symmetrical conjugated diene **5c** underwent monoepoxidation to **6c** in 90% yield with 20:1 selectivity, leveraging electronic differentiation of the alkenes upon installation of the first epoxide. Linalyl propionate **5d** underwent exclusive epoxidation at the trisubstituted alkene, without any competing reaction at the terminal alkene, to afford **6d** in 85% yield. Finally, geranyl propionate **5e** showed a similar preference for reaction at the most electron-rich alkene, generating epoxide **6e** in 79% yield with 6:1 selectivity.

**Figure 5:**
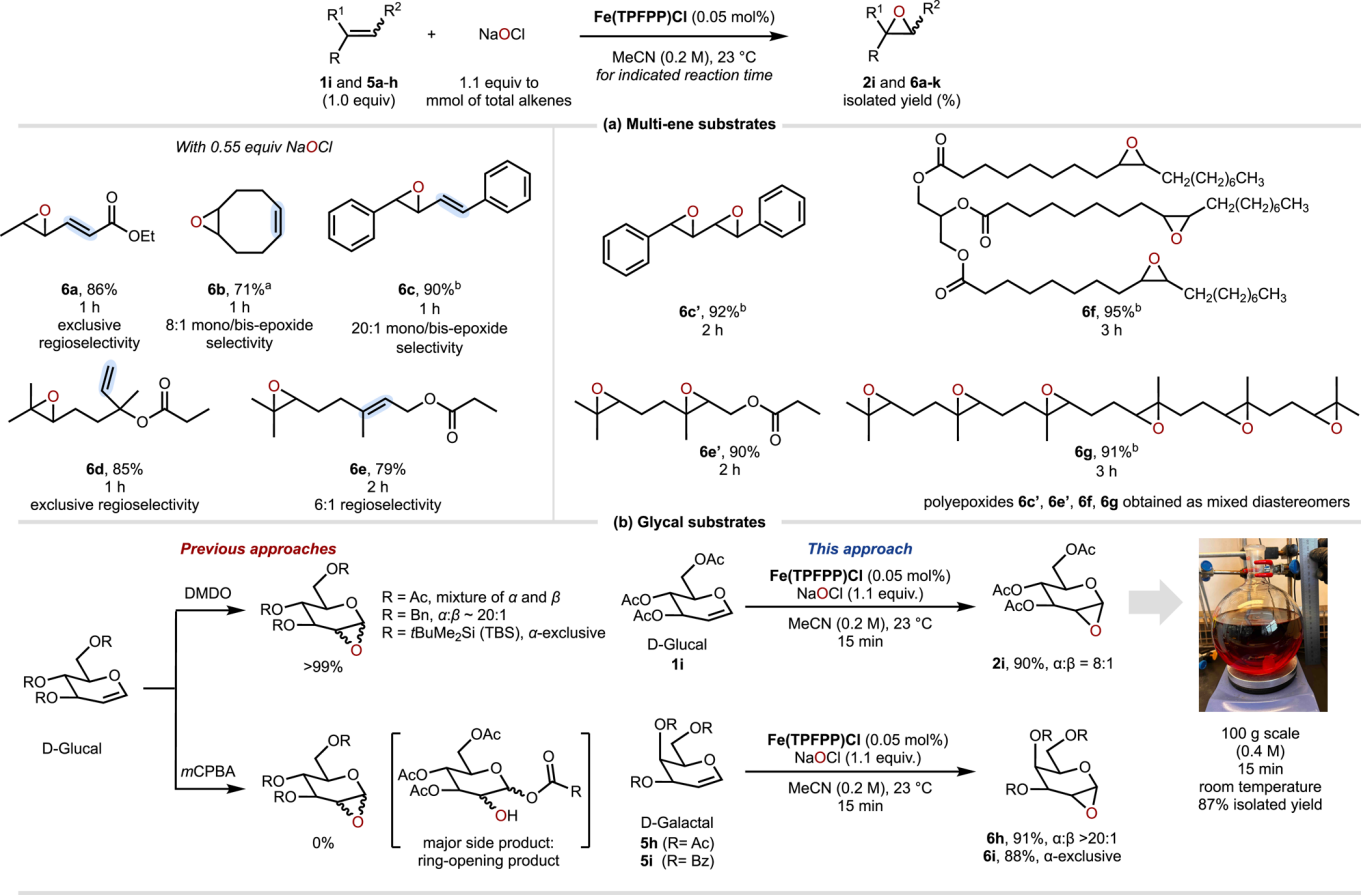
(a) Scope of regioselective epoxidation of multiene substrates. Reaction conditions: Alkene (0.2 mmol), Fe­(TPFPP)Cl (0.05 mol %), NaOCl (0.55 or 1.1 equiv) in MeCN (1 mL) at 23 °C for the indicated time. *mono-*:*di-* epoxidation selectivity was determined by ^1^H NMR analysis of the crude reaction mixture. ^a^2.0 mmol of substrate was used. ^b^A mixture of MeCN and CH_2_Cl_2_ (2:1) was used as the solvent. (b) Diastereoselective epoxidation of glycal substrates. Reaction conditions: Alkene (0.2 mmol), Fe­(TPFPP)Cl (0.05 mol %), NaOCl (1.1 equiv) in MeCN (1 mL) at 23 °C for 15 min. Diastereoselectivity was determined by ^1^H NMR analysis of the isolated products, and the orientation of the major product was assigned after methanolysis of the epoxide.

Increasing the stoichiometry of the NaOCl oxidant enabled di-, tri-, and polyepoxidation of substrates with more than one alkene under similarly mild conditions. Diene **5c** afforded the bis-epoxide **6c’** in 92% yield, and geranyl propionate **5e** furnished bis-epoxide **6e’** in 90% yield under these conditions. Glyceryl trioleate, which contains three alkenes, generated the tris-epoxide **6f** in excellent yield (95%), and squalene **5g** progressed to the hexakis-epoxide product **6g** in 91% yield. These compounds were isolated as a mixture of diastereomers.

Glycal epoxides are important intermediates for the stereospecific construction of β-linked oligosaccharides.^33^ DMDO is commonly used to access these epoxides^34,35^ because *m*CPBA often leads to undesirable ring-opening of the epoxide, unless an additive such as KF is used in the reaction (Figure 5b, left).^36^ Catalytic epoxidation of **1i** has been reported using a ruthenium porphyrin catalyst with 2,6-dichloropyridine *N*-oxide as the oxidant;^37^ however, the corresponding glycal epoxides were not isolated, and only methanolysis products were reported.

The present catalyst system promotes epoxidation of acylated glycals to afford the corresponding glycal epoxides in good yield and diastereoselectivity (Figure 5b, right). For example, tri-*O*-acetyl-*D*-glucal (**1i**) provided epoxide **2i** in 90% yield with an α:β ratio of 8:1. *D*-galactals bearing *O*-acetyl or *O*-benzoyl groups (**5h** and **5i**) exhibited excellent diastereoselectivity (20:1 and α-exclusive, respectively) and high yields of the corresponding epoxides (91% and 88% yield, respectively). The effectiveness of these reactions was demonstrated on a larger scale in the epoxidation of 100 g of **1i** (0.4 M acetonitrile). The reaction, which used 0.05 mol % of Fe­(TPFPP)Cl with 1.1 equiv NaOCl, progressed to completion in only 15 min at room temperature and afforded the desired epoxide in 87% yield. This result shows that the present catalytic method not only offers a practical alternative to *m*CPBA but also to DMDO-based epoxidation methods. Both of these methods present significant safety hazards on a large scale.

In summary, we have discovered and optimized a highly practical Fe­(TPFPP)­Cl-catalyzed alkene epoxidation method that uses NaOCl as an oxidant. The catalyst is commercially available and requires no additional ligands or cocatalysts. The protocol exhibits broad substrate scope and functional group tolerance, and it operates under ambient conditions in a nonchlorinated solvent (MeCN). The high reactivity and selectivity observed in this study suggest that this catalyst system is competitive or superior to *m*CPBA on a small scale and offers significant advantages in safety and atom economy on a large scale.

## Supplementary Material



## Notes

Experimental procedures, additional data, and characterization data (PDF)
