# Supplementary material for: Fe(porphyrin)-Catalyzed Alkene Epoxidation with NaOCl: A Practical Small- and Large-Scale Alternative to mCPBA
Source: J Am Chem Soc. 2026 May 26;148(22):22426–32. doi: 10.1021/jacs.6c06785 (PMC13266983; doi:10.1021/jacs.6c06785)
Supplement: Supplementary file 1 [file ja6c06785_si_001.pdf]

Supporting Information

Fe(porphyrin)-Catalyzed Alkene Epoxidation with NaOCl: A Practical Small- and Large-Scale Alternative to *m*CPBA

Sudip Maiti,[a,b,‡] Doohyun Baek,[a,b,‡] Shannon S. Stahl[a,b,]*

[a]Department of Chemistry, University of Wisconsin-Madison, Madison, WI, 53706, United States
[b]Wisconsin Energy Institute, University of Wisconsin−Madison, Madison, Wisconsin 53726, United States
*stahl@chem.wisc.edu

Table of Contents:

1. General Experimental Considerations S2
2. Experimental Procedures S2
   2a. Preparation of Metal Complexes S2
   2b. Iodometric Titration of Sodium Hypochlorite Solution S3
   2c. Reaction Optimization S3
   2d. General Experimental Procedures S7
   2e. Comparison of Different Epoxidation Methods with 12 Common Substrates S7
   2f. Robustness Screening with Various Additives S10
   2g. Scale-up process S13
   2h. Unsuccessful Substrates S13
3. Product Characterization Data S14
4. References S32
5. NMR Spectra S35

1. General Experimental Considerations

All reagents were purchased and used as received unless otherwise noted. Alkene substrates were purchased from Oakwood, Combi-Blocks, TCI America, Ambeed, Sigma-Aldrich. Sodium hypochlorite solution (available chlorine 4.00-4.99%) was purchased from Sigma-Aldrich and the concentration of hypochlorite (OCl^-) in the NaOCl solution was determined by iodometric titration (see Section 2b in this Supporting Information). 3-Chloroperbenzoic acid (*m*CPBA; 77%) was purchased from Sigma-Aldrich. Iodosylbenzene was prepared by the hydrolysis of the corresponding diacetate (purchased from Sigma-Aldrich) with aqueous sodium hydroxide.[1] Catalysts employed in this study were purchased from commercially available sources or prepared according to established literature protocols (Section 2a in this Supporting Information).[2]

Column chromatography was performed using an automated Combi-Flash with reusable 12 g, 25 g, or 40 g Silicycle cartridges or standard silica cartridges purchased from Teledyne Isco. Thin layer chromatographic (TLC) analysis was performed on Kieselgel 60 F254 aluminum backed silica plates, visualizing with UV light and/or staining with phosphomolybdic Acid (PMA) solution (10% in EtOH). NMR spectra (^1H and ^{13}C) were obtained with either a Bruker Avance III 400 MHz spectrometer or a Bruker Avance III 500 MHz spectrometer referenced against the residual solvent peaks: $CDCl_3$ peaks at 7.26 ppm (^1H) and 77.16 ppm (^{13}C); multiplicities are described using the following abbreviations: s = singlet, d = doublet, dd = doublet of doublet, m = multiplet. High-resolution mass spectra were obtained using a Thermo Q Exactive™ Plus via (ASAP-MS) by the mass spectrometry facility at the Univ. of Wisconsin-Madison.

2. Experimental Procedures

2a. Preparation of Metal Complexes

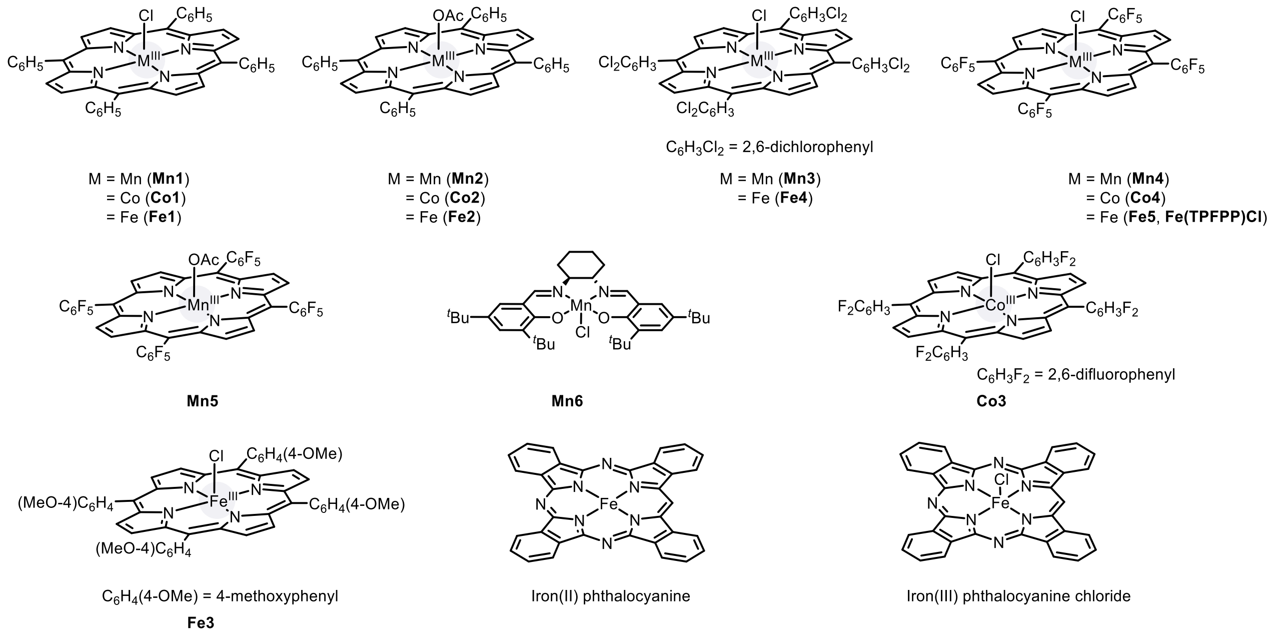

Figure S1. Structures of metal-complexes used in this study.

The catalysts **Fe1, Fe2, Fe3,** and **Fe4** were purchased from Frontier Scientific Inc. **Fe5 (Fe(TPFPP)Cl)** was purchased from Sigma-Aldrich. Iron(II) phthalocyanine was purchased from Strem Chemicals. Iron(III) phthalocyanine chloride was purchased from Sigma-Aldrich. Porphyrin ligands were purchased from Frontier Specialty Chemicals. **Mn1** was purchased from Sigma-Aldrich. **Mn2** was purchased from Strem Chemicals. **Mn3** was purchased from Frontier Specialty Chemicals. Mn6 was purchased from. All the Co-catalysts (**Co1**, **Co2**, **Co3**, and **Co4**) used in this study were purchased from Frontier Scientific Inc. **Mn4**,

and **Mn5** were prepared according to established literature protocols.

The complex **Mn4** was synthesized with slight modification of the known literature procedure.[2] In a 250 mL two-neck round-bottom flask, meso-tetra(pentafluorophenyl) porphyrin (H_2TPFPP) (487 mg, 0.5 mmol) and manganese (II) chloride tetrahydrate ($MnCl_2•4H_2O$) (1 g, 5 mmol) were dissolved in 100 mL of N,N-dimethylformamide (DMF) and refluxed for 12 h until all of the ligand was consumed as monitored by UV-vis spectroscopy. When the reaction was complete, the resulting mixture was allowed to rise to rt, and then the volume of the mixture was reduced to 20 mL in vacuo. Ice-cold water (100 mL) was added to the reaction mixture to obtain a green precipitate. The solid was filtered and washed with ice-cold water (20 × 3 mL) and dried under vacuum. Isolated yield: 495 mg (0.46 mmol, 93%).

The complex **Mn5** was synthesized by following a literature procedure.[2] In a 250 mL two-neck round bottom flask, meso-tetra(pentafluorophenyl) porphyrin (H_2TFPP) (487 mg, 0.500 mmol) and manganese(II) acetate tetrahydrate [$Mn(OAc)_2•4H_2O$] (1.2 g, 5.0 mmol) were dissolved in 50 mL of *N*,*N*-dimethylformamide (DMF) and refluxed for 12 h until all the ligand was consumed, as monitored by UVvis spectroscopy. When the reaction was complete, the resulting mixture was allowed to rise to rt, and then the volume of the mixture was reduced to 5 mL in vacuo. Ice-cold water (100 mL) was added to the mixture, forming a dark brown precipitate, which was then filtered, washed with ice-cold water (10 × 5 mL), and dried under vacuum. The solid was then extracted with acetone (5 × 5 mL), where the desired complex was soluble. The solvent was removed under reduced pressure to yield a dark brown solid. Isolated yield: 495 mg (0.460 mmol, 91%).

2b. Iodometric Titration of Sodium Hypochlorite Solution

A 25.0 mL aliquot of the NaOCl solution (Sigma-Aldrich, available chlorine 4.00-4.99%) was transferred to a 250 mL volumetric flask (10-fold dilution). The flask was filled to the mark with H_2O. A 25.0 mL aliquot of the diluted bleach solution was transferred to a 250 mL Erlenmeyer flask. KI (1.0 g) and 10% H_2SO_4 (v/v in H_2O, 10 mL) were added, resulting in the immediate formation of iodine, as indicated by the brown coloration. The formed iodine was titrated with standardized 0.1 M aqueous $Na_2S_2O_3$ solution using a burette. The reaction mixture was continuously swirled during titration, and the walls of the flask were rinsed periodically with H_2O. The titration was continued until the solution color faded to pale yellow. At this stage, 1% w/v aqueous starch indicator solution was added (4 drops), producing a dark blue color. The titration was then continued dropwise until the blue color disappeared, indicating the endpoint (Amount of the added $Na_2S_2O_3$ solution = 27.9 mL). By the equation $n_AM_BV_B = n_BM_AV_A$ (n = stoichiometric coefficient, M = molarity, V = volume; A = $S_2O_3^{2-}$, B = OCl^-), the molar concentration of OCl^- can be calculated (n_A = 2, M_A = 0.1 M, V_A = 27.9 mL; n_B = 1, M_B = x, V_B = 25 mL). The molarity of the hypochlorite anion (OCl^-, M_B) was calculated to be 0.0558 M. Accounting for the initial 10-fold dilution, the concentration of the OCl^- in the original NaOCl solution was determined to be 0.558 M. For example, a 395 μL aliquot of the NaOCl solution contains 0.22 mmol of OCl^-.

2c. Reaction Optimization

All reactions were carried out under ambient conditions (i.e., at room temperature with no intentional exclusion of air). To an oven-dried 4-dram vial (8 mL) equipped with a magnetic stir bar was added the metal catalyst and 4-phenyl-1-butene (0.2 mmol; 1 equiv). Subsequently, 1.0 mL of solvent was added by syringe. The reaction mixture was stirred for 2 min, after which NaOCl solution was added by syringe to the organic phase. The vial was then sealed, and the reaction was stirred for 6 h. The reaction mixture was diluted with DCM (5 mL), transferred to a separating funnel. The crude mixture was quenched with saturated aqueous $Na_2S_2O_3$ (1 mL), followed by the addition of saturated aqueous NaCl (5 mL). The solution was extracted with DCM (10 mL × 4), and the organic layers were combined and dried over anhydrous Na_2SO_4 and concentrated under reduced pressure. (Note: for many substrate EtOAc is equally effective to extract the product.) Then, 0.2 mmol of CH_2Br_2 (13.9 μL) was added to the crude mixture. The composition of the crude residue was determined by ^1H NMR spectroscopy in $CDCl_3$ using CH_2Br_2 as an external standard.

Table S1. Solvent Screening

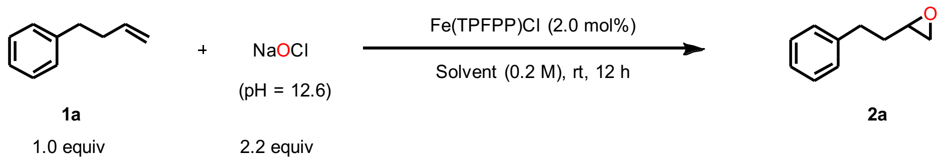

Entry	Solvent	Yield of **2** (%)
1	MeCN	92
2	EtOAc	0
3	EtOAc + BDTAC (5 mol%)	90
4	CH_2Cl_2	0
5	CH_2Cl_2 + BDTAC (5 mol%)	91
6	$ClCH_2CH_2Cl$	0
5	THF	0
6	Et_2O	0
7	Hexane	0

Table S2. Optimization of Reaction Time

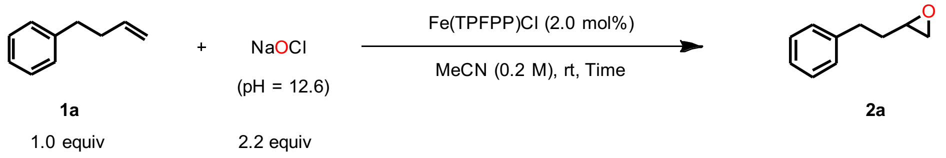

Entry	Time (h)	Yield of **2a** (%)
1	12	92
2	10	92
3	8	90
4	6	92
5	3	85

Table S3. Optimization of Reaction Temperature

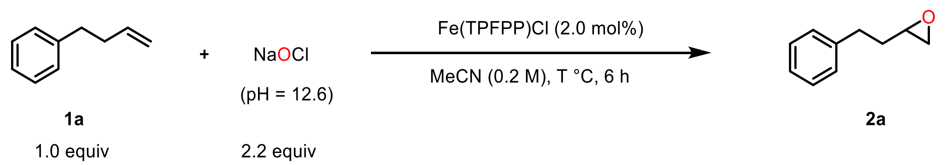

Entry	Temperature (°C)	Yield of **2a** (%)
1	23	92
2	35	75
3	50	42
4	0	70
5	-10	30
6	-20	10

Table S4. Optimization of Catalyst Loading

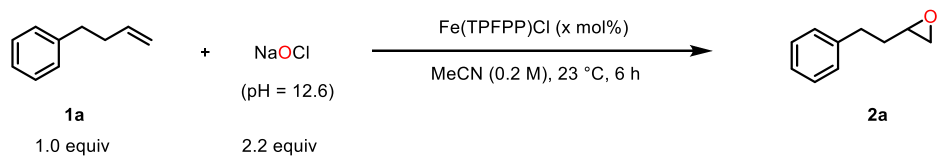

Entry	Fe(TPFPP)Cl (mol%)	Yield of **2a** (%)
1	2.0	92
2	1.0	92
3	0.5	93
4	0.2	92
5	0.1	84

Table S5. Optimization of NaOCl Loading

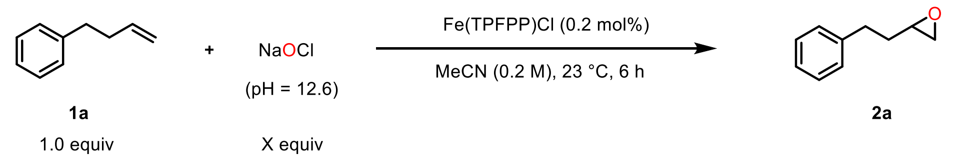

Entry	NaOCl (X equiv)	Yield of **2a** (%)
1	2.2	92
2	1.8	91
3	1.5	93
4	1.1	92

Table S6. Oxidant Screening

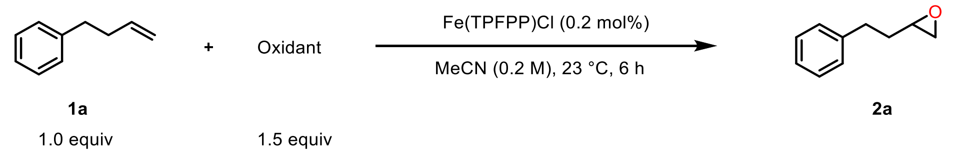

Entry	Oxidant (1.5 equiv)	Yield of **2a** (%)
1	PhIO	39
2	H_2O_2	0
3	H_2O_2 + butanedione (0.5 equiv)	0
4	H_2O_2 + AcOH (30 mol%)	0
5	32% CH_3CO_3H	0
6	NaOCl (1.1 equiv)	92

Table S7. Concentration Screening

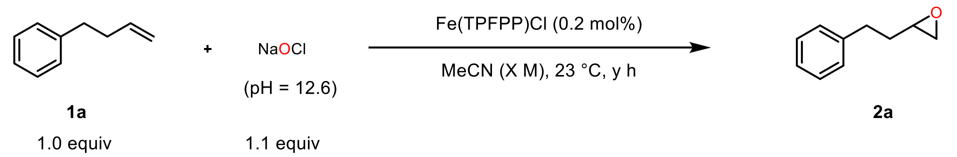

Entry	Concentration (x M)	time (y h)	Yield of **2a** (%)
1	0.2	3	84
2	0.2	6	92
3	0.4	3	94
4	0.4	6	97
5	0.8	3	97
6	0.8	6	99

2d. General Experimental Procedures

All reactions were carried out at room temperature under air atmosphere. To an oven-dried 4-dram vial (8 mL) equipped with a magnetic stir bar was added the corresponding alkene (0.2 mmol; 1 equiv). Subsequently, 1 mL of a Fe(TPFPP)Cl catalyst stock solution (0.1 mg of Fe(TPFPP)Cl in 1mL of MeCN; 0.05 mol%; See below note for the stock solution) was added. The reaction mixture was stirred for 2 min, after which NaOCl solution (0.22 mmol of OCl^-; 1.1 equiv) was added by syringe to the organic phase. The vial was then sealed, and the reaction was stirred at 200 rpm for the desired time periods, depending on the substrate (see individual entries in Figures 4 and 5 of the manuscript and section 3 of the Supporting Information for details). When the reaction was complete, as indicated by TLC with phosphomolybdic acid (PMA) stain, the reaction mixture was diluted with DCM (5 mL), transferred to a separating funnel. The crude mixture was quenched with saturated aqueous $Na_2S_2O_3$ (1 mL), followed by the addition of saturated aqueous NaCl (5 mL). The solution was extracted with DCM (10 mL × 4), and the organic layers were combined and dried over anhydrous Na_2SO_4 and concentrated under reduced pressure. In most cases, the product was obtained through a short celite plug using Et_2O as the eluent without further purification. Otherwise, the product was purified by flash column chromatography to afford the desired epoxide (See Section 3 in this Supporting Information).

Note on reaction time: The reaction time for each substrate is indicated in Figures 4 and 5, and also Section 3 in this Supporting Information. The reaction time is especially important for the glycal substrates (**1i**, **5g**, **5h**). The reaction is typically complete within 20 min, and a significantly decreased yield was observed when the reaction time exceeded 30 min. Decomposition was accompanied by the reaction mixture turning bright pink.

Note on catalyst loading: The catalyst loading can generally be reduced to 0.05 mol% for aromatic and electron-rich alkene substrates. Some aliphatic alkene substrates require a higher catalyst loading of up to 0.2 mol%, as noted in Figures 4 and 5 of the manuscript and Section 3 of this Supporting Information.

Note on catalyst stock solution: Catalyst loadings were controlled using stock solutions due to the difficulty of accurately weighing the very small amount of catalyst required. 1 mL of a catalyst stock solution (1.06 mg of Fe(TPFPP)Cl in 10 mL MeCN, prepared in a 10 mL volumetric flask) was used to deliver a 0.05 mol% catalyst loading to the 0.2 mmol of starting substrates. For a 0.2 mol% catalyst loading, 1 mL of a stock solution (4.24 mg of Fe(TPFPP)Cl in 10 mL MeCN, prepared in a 10 mL volumetric flask) was used to the 0.2 mmol of starting substrates.

Note on extraction: After the reaction, heteroaromatic substrates such as 2-(oxiran-2-yl)pyridine (**1j**), 3-(oxiran-2-yl)pyridine (**3j**), and 2-(oxiran-2-yl)pyrazine (**1k**) require thorough extraction. At least four extractions with 10 mL portions of DCM are necessary, and TLC monitoring (with PMA Staining) is recommended to ensure complete extraction of the products. For other substrates, EtOAc was equally effective as an extraction solvent.

Note on NMR Spectra containing CH_2Br_2 as standard: The CH_2Br_2 was added after determination of the mass-based yield. This standard was included early in our studies as a diagnostic step ensure that no NMR-silent impurities, such as inorganic salts, were retained in our products following purification.

2e. Comparison of Different Epoxidation Methods with 12 Common Substrates

General procedure for Method I (stoichiometric *m*CPBA epoxidation)

Reactions were carried out at room temperature under air atmosphere. To an oven-dried 4-dram vial (8 mL) equipped with a magnetic stir bar was added alkene (0.2 mmol, 1.0 equiv) and the vial was placed in an ice bath. Then *m*-chloroperoxybenzoic acid (1.2 equiv) in 1mL of CH_2Cl_2 was added slowly into the vial by syringe. The mixture was stirred at room temperature for an additional 12 h. At the end of the reaction, the reaction mixture was washed with saturated aqueous $NaHCO_3$ and extracted with CH_2Cl_2 (10 mL × 4). The organic layers were combined and dried over anhydrous Na_2SO_4. After solvent evaporation under reduced pressure, the composition of the crude residue was determined by ^{1}H NMR spectroscopy in $CDCl_3$ using CH_2Br_2 as an external standard (13.9 μL, 0.2 mmol).

General procedure for Method II (stoichiometric *in situ* generated DMDO epoxidation[3])

Reactions were carried out under an air atmosphere. To an oven-dried 4-dram vial (8 mL) equipped with a magnetic stir bar was added alkene (0.2 mmol, 1.0 equiv), CH_2Cl_2/acetone solution (10:1 ratio, 1.1 mL), and saturated aqueous $NaHCO_3$ (1.8 mL), and the vial was placed in an ice bath. Then, a solution of Oxone (0.4 mmol, 2.0 equiv) in H_2O (1.0 mL) was added slowly into the vial by syringe over 5 min. The mixture was stirred at 0 °C for 30 min and then at room temperature for an additional 6 h. At the end of the reaction, the organic phase was separated, and the aqueous phase was extracted with CH_2Cl_2 (10 mL × 4). The organic layers were combined and dried over anhydrous Na_2SO_4. After solvent evaporation under reduced pressure, the composition of the crude residue was determined by ^{1}H NMR spectroscopy in $CDCl_3$ using CH_2Br_2 as an external standard (13.9 µL, 0.2 mmol).

Note: For substrates **1a** and **1b**, 3 equiv of Oxone were used, and the reaction time was extended to 12 h.

General procedure for Method III (Reported by Meunier and co-workers[4])

In accordance with the literature procedure,[4] reactions were carried out at room temperature under nitrogen atmosphere. To an oven-dried 4-dram vial (8 mL) equipped with a magnetic stir bar was added Mn(TPP)OAc (2.5 mol%), benzyldimethyltetradecylammonium chloride (1 mol%), pyridine (0.15 equiv), and alkene (0.2 mmol; 1.0 equiv). Subsequently, 1.0 mL of N_2-purged dichloromethane (CH_2Cl_2) was added by syringe. The reaction mixture was stirred for 2 min, after which NaOCl solution (0.36 mmol of OCl^-;1.8 equiv) was added by syringe to the organic phase. The reaction mixture was stirred for 4 h at room temperature. At the end of the reaction, the reaction mixture was washed with saturated aqueous NaCl and extracted with CH_2Cl_2 (10 mL × 3). The organic layers were combined and dried over anhydrous Na_2SO_4. After solvent evaporation under reduced pressure, the composition of the crude residue was determined by ^{1}H NMR spectroscopy in $CDCl_3$ using CH_2Br_2 as an external standard (13.9 µL, 0.2 mmol).

General procedure for Method IV (Reported by Jacobsen and co-workers[5])

Between two publications by Jacobsen and co-workers in 1991[6] and 1994[5] on asymmetric epoxidation using NaOCl, the latter method was selected due to the beneficial effect of pyridine-*N*-oxide as an axial ligand. In accordance with the literature procedure,[5] reactions were carried out at room temperature under nitrogen atmosphere. To an oven-dried 4-dram vial (8 mL) equipped with a magnetic stir bar was added **Mn6** (3.0 mol%), 4-phenyl pyridine-*N*-oxide (0.2 equiv), and alkene (0.2 mmol; 1.0 equiv). Subsequently, 0.5 mL of CH_2Cl_2 was added by syringe. The reaction mixture was stirred for 2 min, after which NaOCl solution (0.3 mmol of OCl^-, 1.5 equiv) was added by syringe to the organic phase at 0 ºC. The reaction mixture was allowed to stir for 7 h at room temperature. At the end of the reaction, the reaction mixture was washed with saturated aqueous NaCl and extracted with CH_2Cl_2 (10 mL × 3). The organic layers were combined and dried over anhydrous Na_2SO_4. After solvent evaporation under reduced pressure, the composition of the crude residue was determined by ^{1}H NMR spectroscopy in $CDCl_3$ using CH_2Br_2 as an external standard (13.9 µL, 0.2 mmol).

General procedure for Method V (Reported by Noyori and co-workers[7])

In accordance with the literature procedure,[7] reactions were carried out under air atmosphere. To an oven-dried 4-dram vial (8 mL) equipped with a magnetic stir bar was added $Na_2WO_4{\cdot}2H_2O$ (2.0 mol%), $NH_2CH_2PO_3H_2$ (1.0 mol%), $[CH_3(n\text{-}C_8H_{17})_3N]HSO_4$ (1.0 mol%), and aqueous 30% H_2O_2 (1.5 equiv; 300 µL), and the mixture was vigorously stirred at room temperature for 15 min. To this was added alkene (2.0 mmol, 1.0 equiv), and the mixture was heated at 90 °C for 2 h with stirring at 1000 rpm before cooling to room temperature. The reaction was quenched with 5 mL of saturated aqueous $Na_2S_2O_3$. The solution was extracted with ethyl acetate (10 mL × 3) and the organic layers were combined and dried over anhydrous Na_2SO_4 and concentrated under reduced pressure. The composition of the crude residue was determined by ^{1}H NMR spectroscopy in $CDCl_3$ using CH_2Br_2 as an external standard (13.9 µL, 0.2 mmol).

General procedure for Method VI (Reported by Browne and co-workers[8])

In accordance with the literature procedure,[8] reactions were carried out under air atmosphere. First, two stock solutions were prepared. A catalyst stock solution containing 1.8 mg $Mn(ClO_4)_2 \cdot 6H_2O$ and 6.2 mg of 2-picolinic acid in MeCN was prepared in a 100 mL volumetric flask such that a 2 mL aliquot delivered the desired loadings for the reaction (0.0361 mg of $Mn(ClO_4)_2 \cdot 6H_2O$ (0.01 mol%) and 0.123 mg of 2-picolinic acid (0.5 mol%) to 1.0 mmol alkene). Separately, a 10 mL of 0.6 M aqueous NaOAc stock solution was prepared in a 10 mL of volumetric flask.

To an oven-dried 4-dram vial (8 mL) equipped with a magnetic stir bar were added an aliquot (2 mL) of the $Mn(ClO_4)_2 \cdot 6H_2O$/2-picolinic acid stock solution (0.01 mol%/0.5 mol%), alkene (1.0 mmol, 1.0 equiv), NaOAc (1 mol%, 16.7 µL of 0.6 M aqueous stock solution), and 2,3-butanedione (0.5 equiv, 43.5 µL). The reaction mixture was cooled down to 0 °C. Aqueous H_2O_2 (50 wt%, 1.5 equiv, 85 µL) was then added, and the reaction mixture was stirred for 1 h. The crude mixture was quenched with saturated aqueous $Na_2S_2O_3$ (1 mL), followed by the addition of saturated aqueous NaCl (5 mL). Then the mixture was extracted with CH_2Cl_2. The combined organic layers were washed with brine, dried over anhydrous Na_2SO_4, filtered, and concentrated under reduced pressure., the composition of the crude residue was determined by ^1H NMR spectroscopy in $CDCl_3$ using CH_2Br_2 as an external standard (13.9 µL, 0.2 mmol).

General procedure for Method VII (Reported by Stack and co-workers[9])

Preparation of CH_3CO_3H Oxidant Solution[9]

An aliquot (0.5 mL) of commercial 32% peracetic acid (PAA) was neutralized with 10% aqueous KOH (0.15 mL), then diluted to a 1:1 v/v mixture with AcOH (0.65 mL). The pH of the resulting solution was 1.9. The final concentration of CH_3CO_3H oxidant solution was 2.0 M. This mixture was freshly made before each reaction. *Note:* The catalytic system was not compatible with the direct use of the commercial 32% peracetic acid solution.

In accordance with the literature procedure,[9] reactions were carried out under air atmosphere. To an oven-dried 4-dram vial (8 mL) equipped with a magnetic stir bar was added $Mn(OAc)_2$ (0.4 mol%), 2-picolinic acid (2.0 mol%), and alkene (0.2 mmol, 1.0 equiv). Subsequently, 0.5 mL of MeCN was added by syringe, and the resulting mixture was brought to 0 °C. The freshly prepared CH_3CO_3H (1.1 equiv) was added slowly to the stirring reaction mixture. The reaction was stirred at 0 °C for 5 min. At the end of the reaction, the reaction mixture was washed with saturated aqueous NaCl and extracted with CH_2Cl_2 (10 mL × 3). The organic layers were combined and dried over anhydrous Na_2SO_4. After solvent evaporation under reduced pressure, the composition of the crude residue was determined by ^1H NMR spectroscopy in $CDCl_3$ using CH_2Br_2 as an external standard (13.9 µL, 0.2 mmol).

General procedure for Method VIII (This work)

Follow the reaction details in this Supporting Information Section 2d.

Summary of the substrate screening with literature protocol

Table S8. Obtained yield from the generality test with various alkenes

(a)

Method I
[O]: *m*CPBA
CH_2Cl_2, 0 °C to 23 °C

Method II
[O]: **DMDO** (acetone/oxone)
CH_2Cl_2, $NaHCO_3$, 0 °C to 23 °C

Method III
[Cat]: Mn(TPP)OAc; pyridine
[O]: NaOCl; BDTAC
CH_2Cl_2, 23 °C

Method IV
[Cat]: Mn-Salen; 4-PhPy-*N*-Oxide
[O]: NaOCl
CH_2Cl_2, 0 °C

Method V
[Cat]: Na_2WO_4; $[CH_3(n\text{-}C_8H_{17})_3N]HSO_4$
[O]: H_2O_2; $NH_2CH_2PO_3H_2$
No solvent, 90 °C

Method VI
[Cat]: $Mn(ClO_4)_2$; 2-picolinic acid
[O]: H_2O_2; NaOAc; butanedione
MeCN, 23 °C

Method VII
[Cat]: $Mn(OAc)_2$; 2-picolinic acid
[O]: CH_3CO_3H
MeCN, 0 °C

Method VIII (This work)
[Cat]: Fe(TPFPP)Cl
[O]: NaOCl
MeCN, 23 °C

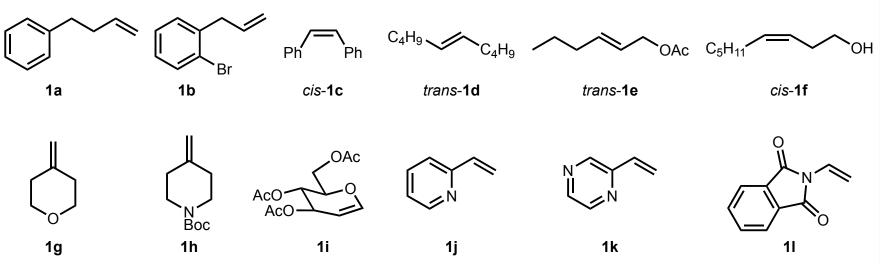

	1a	1b	1c	1d	1e	1f	1g	1h	1i	1j	1k	1l
**Method I**	99	83	99	99	98	98	92	93	0	2	0	5(25)
**Method II**	66	58	85	94	82	92	99	86	86	1	1	67
**Method III**	0	1	72	16	26	94	23	23	29	32	10	18
**Method IV**	0	2	86	0	0	69	13	21	17	86	34	35
**Method V**	68	3	94	41	61	60	1	89	3	3	0	0
**Method VI**	35	3	31	67	88	50	1	12	0	0	3	0
**Method VII**	82	76	13	71	86	77	80	91	0	98	91	0
**Method VIII**	99	89	94	93	90	98	85	93	90	75	72	92

Note: The epoxidation of compound **1l** using Method I afforded a higher yield (25%) under a shorter reaction time (1 h) compared to the general reaction time (12 h).

2f. Robustness Screening with Various Additives

Robustness Screening under *m*CPBA Epoxidation

Reactions were carried out at room temperature under air atmosphere. To an oven-dried 4-dram vial (8 mL) equipped with a magnetic stir bar was added 4-*tert*-butylstyrene (0.2 mmol; 1 equiv) and various additives (0.2 mmol, 1.0 equiv). Subsequently, 1.0 mL of CH_2Cl_2 was added by syringe. The reaction vial was placed in an ice bath and stirred for 5 min. Then *m*CPBA (77%, 1.2 equiv) was added slowly to the stirred solution of the reaction mixture at 0 ºC. The reaction mixture was allowed to stir for 1 h at room temperature. At the end of the reaction, the reaction mixture was washed with saturated aqueous $NaHCO_3$ and extracted with CH_2Cl_2 (10 mL × 3). The organic layers were combined and dried over anhydrous Na_2SO_4. After solvent evaporation under reduced pressure, the composition of the crude residue was determined by ^1H NMR spectroscopy in $CDCl_3$ using CH_2Br_2 as an external standard.

Table S9. *m*CPBA epoxidation with various additives

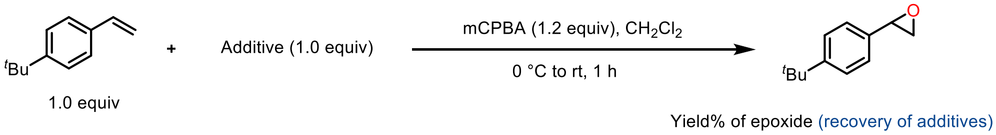

Yield% of epoxide (recovery of additives)

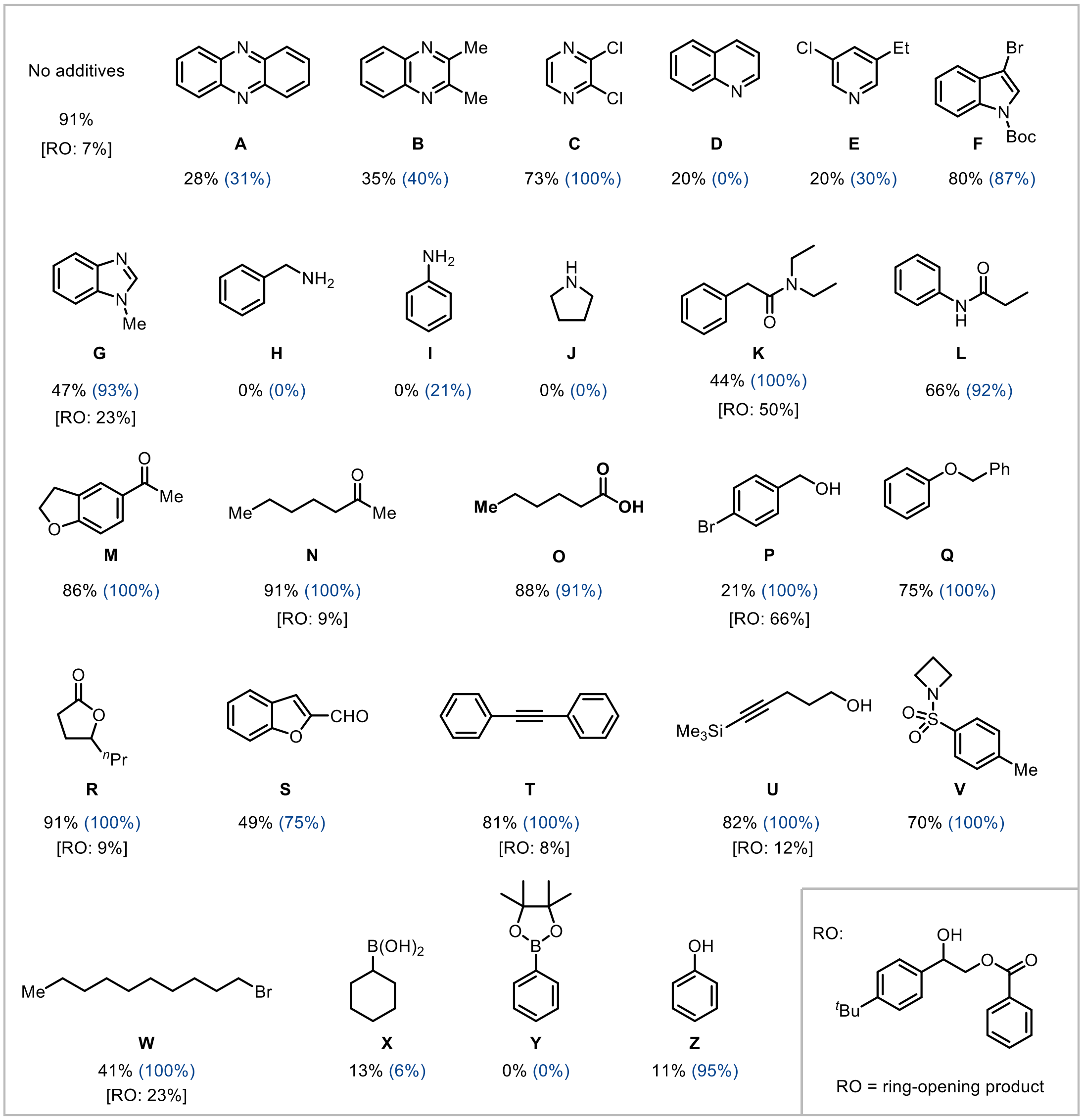

Additive	Yield% of epoxide	(recovery of additives)	RO
No additives	91%		[RO: 7%]
A	28%	(31%)	
B	35%	(40%)	
C	73%	(100%)	
D	20%	(0%)	
E	20%	(30%)	
F	80%	(87%)	
G	47%	(93%)	[RO: 23%]
H	0%	(0%)	
I	0%	(21%)	
J	0%	(0%)	
K	44%	(100%)	[RO: 50%]
L	66%	(92%)	
M	86%	(100%)	
N	91%	(100%)	[RO: 9%]
O	88%	(91%)	
P	21%	(100%)	[RO: 66%]
Q	75%	(100%)	
R	91%	(100%)	[RO: 9%]
S	49%	(75%)	
T	81%	(100%)	[RO: 8%]
U	82%	(100%)	[RO: 12%]
V	70%	(100%)	
W	41%	(100%)	[RO: 23%]
X	13%	(6%)	
Y	0%	(0%)	
Z	11%	(95%)	

Robustness Screening under Fe(TPFPP)Cl/NaOCl-Catalytic System

Reactions were carried out at room temperature under air atmosphere. To an oven-dried 4-dram vial (8 mL) equipped with a magnetic stir bar was added the Fe(TPFPP)Cl catalyst (0.05 mol%), 4-*tert*-butylstyrene (0.2 mmol; 1.0 equiv), and various additives (0.2 mmol, 1.0 equiv). Subsequently, 1.0 mL of MeCN was added by syringe. The reaction mixture was stirred for 2 min., after which NaOCl solution (0.22 mmol of OCl^-; 1.1 equiv) was added by syringe. The vial was then sealed, and the reaction was stirred for 45 min. When the reaction was complete, the reaction mixtures were extracted with CH_2Cl_2 (10 mL × 3) and the organic layers were combined and dried over anhydrous Na_2SO_4. After solvent evaporation under reduced pressure, the composition of the crude residue was determined by ^{1}H NMR spectroscopy in $CDCl_3$ using CH_2Br_2 as an external standard.

Table S10. Fe5/NaOCl catalyzed epoxidation with various additives

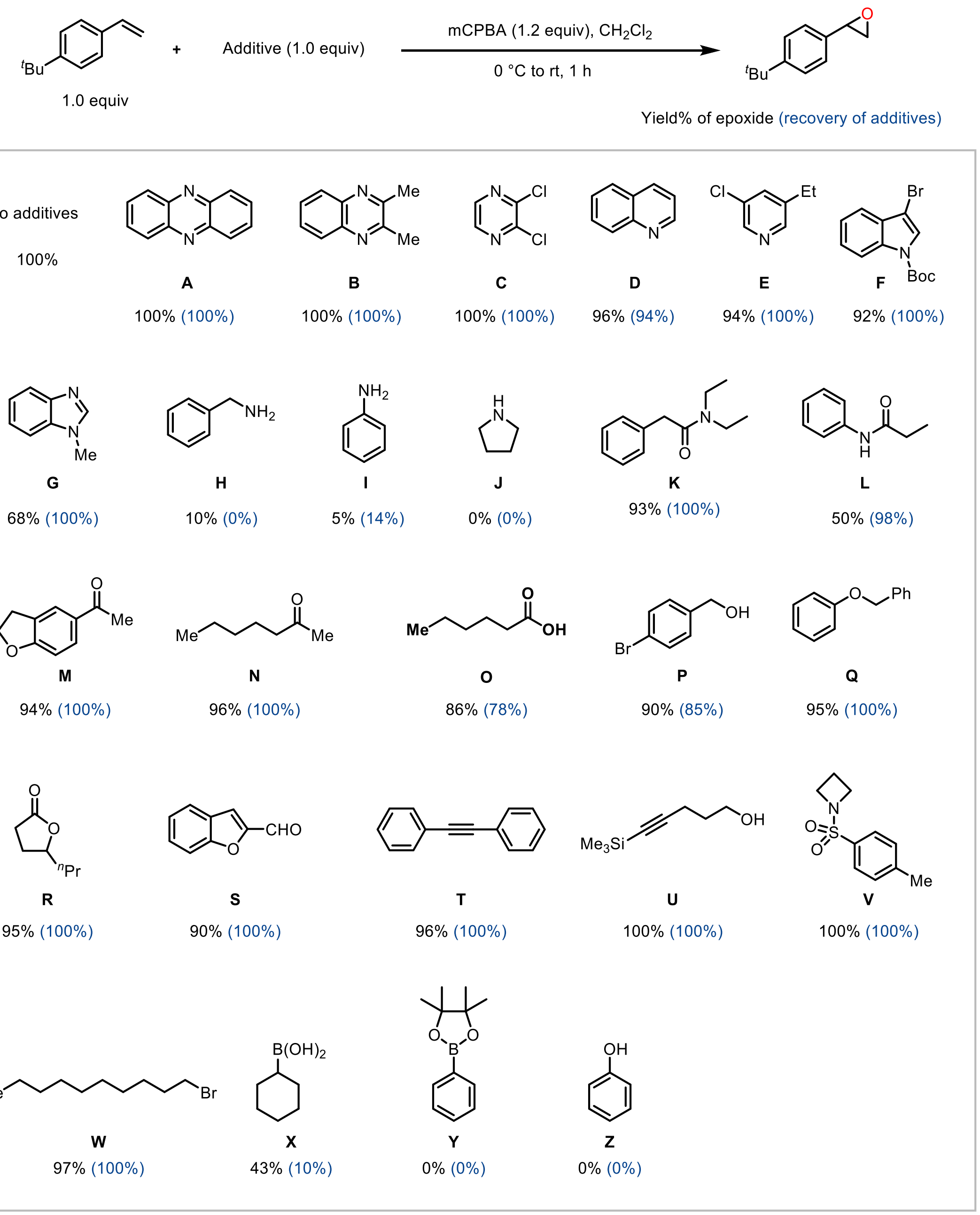

2g. Scale-up process

All reactions were carried out at room temperature under air atmosphere. To a 2000 mL flat-bottom round flask equipped with a magnetic stir bar was added the tri-*O*-acetyl-*D*-glucal (100 g, 367.3 mmol; 1 equiv). Subsequently, Fe(TPFPP)Cl catalyst (195 mg, 0.18 mmol; 0.05 mol%) was added to the flask. Then 900 mL of MeCN was transferred to the flask using a graduated cylinder. The reaction mixture was stirred for 5 min to dissolve the glucal and catalyst, after which an aqueous NaOCl solution (712 mL of 4.0% NaOCl solution, 404 mmol of OCl^-; 1.1 equiv) was poured into the flask using a graduated cylinder. The flask was sealed with septum, and the reaction was stirred vigorously for 15 min.

Upon completion, the reaction mixture was diluted with Et_2O (500 mL) and transferred to a 5L separating funnel. The crude mixture was quenched with saturated aqueous $Na_2S_2O_3$ (1 L) and followed by the addition of saturated aqueous NaCl (1 L). The aqueous layer was extracted with Et_2O (1 L × 4), and the organic layers were dried over anhydrous Na_2SO_4 and concentrated under reduced pressure. The resulting crude was passed through a short plug of Celite and dried under high vacuum to afford the desired product without further purification (93.6 g, 87% of isolated yield).

Note: The selection of the stir bar is crucial to support vigorous mixing. At this scale, a cross-shaped stir bar (50 mm × 50 mm) provided efficient mixing of acetonitrile and the aqueous NaOCl solution.

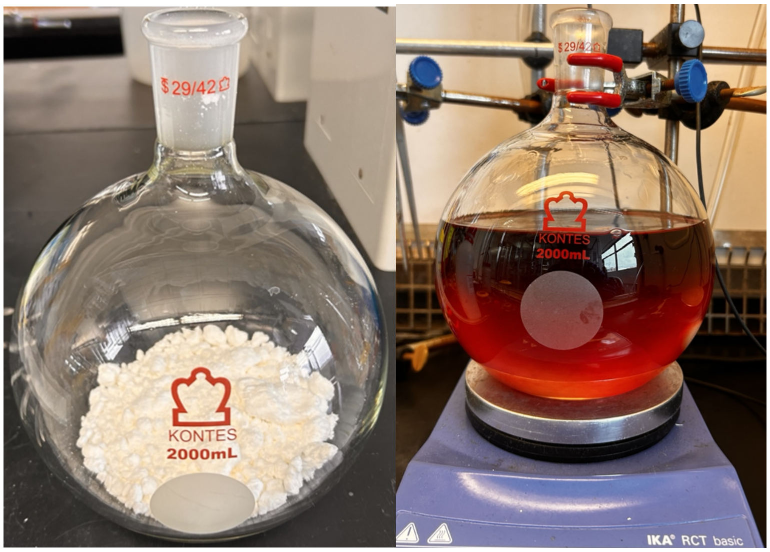

Figure S2. Reaction set up on 100 g scale with 2000 mL flat-bottom round flask under ambient conditions.

2h. Unsuccessful Substrates

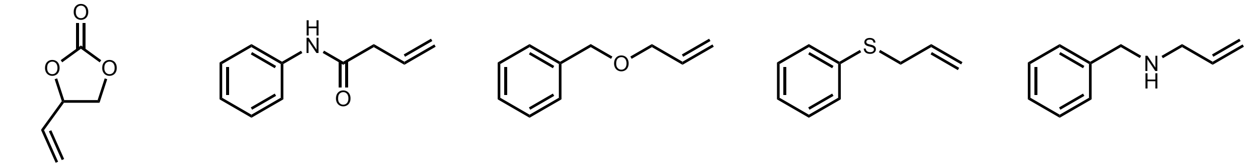

>90% remaining starting material	>90% remaining starting material	17% yield 70% remaining starting material	sulfone/sulfoxide formation	0% yield, no remaining starting material, complex product mixture

3. Product Characterization Data

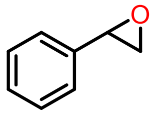

4a

2-phenyloxirane, **4a**:

The reaction was conducted at room temperature for 1 h following general experimental procedure (section 2d). After the extraction, the product was obtained through a short celite plug using Et_2O as the eluent without further purification (22.1 mg, 0.184 mmol, 92% yield). The spectral data are in agreement with reported literature values.[10]

^{1}H NMR (500 MHz, $CDCl_3$) δ 7.39 – 7.27 (m, 5H), 3.87 (dd, J = 4.1, 2.6 Hz, 1H), 3.16 (dd, J = 5.5, 4.1 Hz, 1H), 2.82 (dd, J = 5.5, 2.6 Hz, 1H).

^{13}C NMR (126 MHz, $CDCl_3$) δ 137.6, 128.6, 128.3, 125.5, 52.4, 51.3.

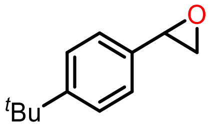

2m

2-(4-(tert-butyl)phenyl)oxirane, **2m**:

The reaction was conducted at room temperature for 0.75 h following general experimental procedure (section 2d). After the extraction, the product was obtained through a short celite plug using Et_2O as the eluent without further purification (33.5 mg, 0.19 mmol, 95% yield). The spectral data are in agreement with reported literature values.[11]

^{1}H NMR (500 MHz, $CDCl_3$) δ 7.39 (d, J = 8.4 Hz, 2H), 7.23 (d, J = 8.4 Hz, 2H), 3.86 – 3.85 (m, 1H), 3.15 (dd, J = 5.5, 4.0 Hz, 1H), 2.83 (dd, J = 5.4, 2.6 Hz, 1H), 1.32 (s, 9H).

^{13}C NMR (126 MHz, $CDCl_3$) δ 151.4, 134.6, 125.5, 125.4, 52.4, 51.2, 34.7, 31.4.

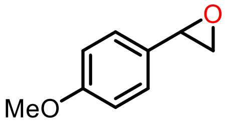

4b

2-(4-methoxyphenyl)oxirane, **4b**:

The reaction was conducted at room temperature for 0.5 h following general experimental procedure (section 2d). After the extraction, the product was obtained through a short celite plug using Et_2O as the eluent without further purification (29.1 mg, 0.194 mmol, 97% yield). The spectral data are in agreement with reported literature values.[11]

^{1}H NMR (500 MHz, $CDCl_3$) δ 7.20 (d, J = 8.8 Hz, 2H), 6.88 (d, J = 8.7 Hz, 2H), 3.82 (dd, J = 4.2, 2.7 Hz, 1H), 3.80 (s, 3H), 3.12 (dd, J = 5.3, 4.0 Hz, 1H), 2.81 (dd, J = 5.3, 2.6 Hz, 1H).

^{13}C NMR (126 MHz, $CDCl_3$) δ 159.7, 129.4, 126.9, 114.0, 55.3, 52.3, 51.1.

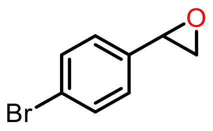

4c

2-(4-bromophenyl)oxirane, **4c**:

The reaction was conducted at room temperature for 1 h following general experimental procedure (section 2d). After the extraction, the product was obtained through a short celite plug using Et_2O as the eluent without further purification (35.8 mg, 0.18 mmol, 90% yield). The spectral data are in agreement with reported literature values.[11]

^{1}H NMR (500 MHz, $CDCl_3$) δ 7.46 (d, J = 8.5 Hz, 2H), 7.14 (d, J = 8.4 Hz, 2H), 3.82 (dd, J = 4.1, 2.5 Hz, 1H), 3.14 (dd, J = 5.5, 4.1 Hz, 1H), 2.75 (dd, J = 5.5, 2.6 Hz, 1H).

^{13}C NMR (126 MHz, $CDCl_3$) δ 136.7, 131.7, 127.2, 122.1, 51.9, 51.3.

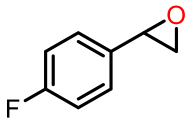

4d

2-(4-fluorophenyl)oxirane, **4d**:

The reaction was conducted at room temperature for 1 h following general experimental procedure (section 2d). After the extraction, the product was obtained through a short celite plug using Et_2O as the eluent without further purification (25.7 mg, 0.186 mmol, 93% yield). The spectral data are in agreement with reported literature values.[10]

^{1}H NMR (500 MHz, $CDCl_3$) δ 7.27 – 7.21 (m, 2H), 7.04 (t, J = 8.7 Hz, 2H), 3.85 (dd, J = 4.1, 2.6 Hz, 1H), 3.14 (dd, J = 5.4, 4.0 Hz, 1H), 2.77 (dd, J = 5.3, 2.5 Hz, 1H).

^{13}C NMR (126 MHz, $CDCl_3$) δ 163.7, 161.8, 133.3, 133.3, 127.3, 127.2, 115.6, 115.5, 51.9, 51.3.

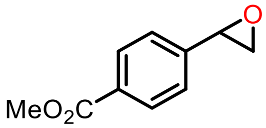

4e

methyl 4-(oxiran-2-yl)benzoate, **4e**:

The reaction was conducted at room temperature for 1 h following general experimental procedure (section 2d). After the extraction, the product was obtained through a short celite plug using Et_2O as the eluent without further purification (32.4 mg, 0.182 mmol, 91% yield). The spectral data are in agreement with reported literature values.[12]

^{1}H NMR (500 MHz, $CDCl_3$) δ 8.01 (d, J = 8.4 Hz, 2H), 7.34 (d, J = 8.4 Hz, 2H), 3.90 (s, 3H), 3.90 – 3.88 (m, 1H), 3.18 (dd, J = 5.6, 4.1 Hz, 1H), 2.78 (dd, J = 5.6, 2.5 Hz, 1H).

^{13}C NMR (126 MHz, $CDCl_3$) δ 166.8, 142.9, 130.0, 129.9, 125.5, 52.2, 52.0, 51.6.

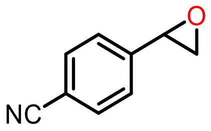

4f

4-(oxiran-2-yl)benzonitrile, **4f**:

The reaction was conducted at room temperature for 1 h following general experimental procedure (section 2d). After the extraction, the product was obtained through a short celite plug using Et_2O as the eluent without further purification (26.9 mg, 0.186 mmol, 93% yield). The spectral data are in agreement with reported literature values.[13]
^{1}H NMR (500 MHz, $CDCl_3$) δ 7.62 (d, J = 8.5 Hz, 2H), 7.38 (d, J = 8.4 Hz, 2H), 3.90 (dd, J = 4.0, 2.5 Hz, 1H), 3.19 (dd, J = 5.5, 4.1 Hz, 1H), 2.74 (dd, J = 5.5, 2.4 Hz, 1H).
^{13}C NMR (126 MHz, $CDCl_3$) δ 143.3, 132.4, 126.1, 118.7, 111.8, 51.7, 51.6.

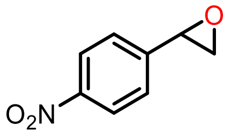

4g

2-(4-nitrophenyl)oxirane, **4g**:
The reaction was conducted at room temperature for 1 h following general experimental procedure (section 2d). After the extraction, the product was obtained through a short celite plug using Et_2O as the eluent without further purification (29.4 mg, 0.178 mmol, 89% yield). The spectral data are in agreement with reported literature values.[13]
^{1}H NMR (500 MHz, $CDCl_3$) δ 8.20 (d, J = 8.8 Hz, 1H), 7.44 (d, J = 8.8 Hz, 1H), 3.96 (dd, J = 4.2, 2.4 Hz, 1H), 3.23 (dd, J = 5.5, 4.1 Hz, 1H), 2.78 (dd, J = 5.4, 2.5 Hz, 1H).
^{13}C NMR (126 MHz, $CDCl_3$) δ 147.8, 145.3, 126.3, 123.9, 51.8, 51.5.

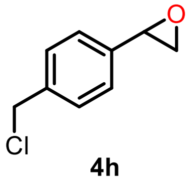

4h

2-(4-(chloromethyl)phenyl)oxirane, **4h**:
The reaction was conducted at room temperature for 1 h following general experimental procedure (section 2d). After the extraction, the product was obtained through a short celite plug using Et_2O as the eluent without further purification (31.0 mg, 0.184 mmol, 92% yield).

^{1}H NMR (500 MHz, $CDCl_3$) δ 7.37 (d, J = 8.2 Hz, 2H), 7.28 (d, J = 8.1 Hz, 2H), 4.58 (s, 1H), 3.87 (dd, J = 4.1, 2.5 Hz, 1H), 3.16 (dd, J = 5.5, 4.0 Hz, 1H), 2.79 (dd, J = 5.5, 2.5 Hz, 1H).
^{13}C NMR (126 MHz, $CDCl_3$) δ 138.0, 137.5, 128.9, 125.9, 52.1, 51.4, 46.0.
HRMS (ESI): Calculated for C_9H_9ClO $[M+H]^+$: 169.0415; found: 169.0413.

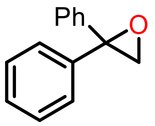

4i

2,2-diphenyloxirane, **4i**:
The reaction was conducted at room temperature for 0.5 h following general experimental procedure (section 2d). After the extraction, the product was obtained through a short celite plug using Et_2O as the eluent without further purification (38.1 mg, 0.194 mmol, 97% yield). The spectral data are in agreement with reported literature values.[14]
^{1}H NMR (500 MHz, $CDCl_3$) δ 7.41 – 7.32 (m, 10H), 3.31 (s, 2H).
^{13}C NMR (126 MHz, $CDCl_3$) δ 139.6, 128.3, 128.0, 127.5, 61.8, 56.9.

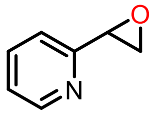

2j

2-(oxiran-2-yl)pyridine, **2j**:

The reaction was conducted at room temperature for 1 h following general experimental procedure (section 2d). After the extraction, the product was purified by silica-gel column chromatography using (DCM:MeOH 98:2) as an eluent (18.1 mg, 0.150 mmol, 75% yield). The spectral data are in agreement with reported literature values.[15]

^{1}H NMR (500 MHz, $CDCl_3$) δ 8.57 – 8.54 (m, 1H), 7.67 (td, J = 7.7, 1.8 Hz, 1H), 7.24 – 7.20 (m, 2H), 4.00 (dd, J = 4.2, 2.5 Hz, 1H), 3.17 (dd, J = 5.8, 4.1 Hz, 1H), 2.93 (dd, J = 5.8, 2.5 Hz, 1H).

^{13}C NMR (126 MHz, $CDCl_3$) δ 157.2, 149.5, 136.9, 123.2, 119.7, 52.9, 50.5.

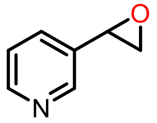

4j

3-(oxiran-2-yl)pyridine, **4j**:

The reaction was conducted at room temperature for 1 h following general experimental procedure (section 2d). After the extraction, the product was purified by silica-gel column chromatography using (DCM:MeOH 98:2) as an eluent (19.0 mg, 0.157 mmol, 78% yield). The spectral data are in agreement with reported literature values.[16]

^{1}H NMR (500 MHz, $CDCl_3$) δ 8.59 (d, J = 1.7 Hz, 1H), 8.56 (dd, J = 4.9, 1.7 Hz, 1H), 7.55 (dt, J = 7.9, 1.8 Hz, 1H), 7.29 (dd, J = 7.9, 3.7 Hz, 1H), 3.91 (dd, J = 4.1, 2.5 Hz, 1H), 3.21 (dd, J = 5.3, 4.1 Hz, 1H), 2.83 (dd, J = 5.3, 2.5 Hz, 1H).

^{13}C NMR (126 MHz, $CDCl_3$) δ 149.7, 147.9, 133.4, 132.8, 123.6, 51.2, 50.5.

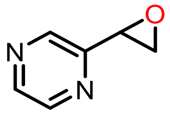

2k

2-(oxiran-2-yl)pyrazine, **2k**:

The reaction was conducted at room temperature for 2 h following general experimental procedure (section 2d). After the extraction, the product was purified by silica-gel column chromatography using (DCM:MeOH 98:2) as an eluent (17.6 mg, 0.144 mmol, 72% yield).

^{1}H NMR (500 MHz, $CDCl_3$) δ 8.57 (s, 1H), 8.54 (brs, 2H), 4.05 (dd, J = 4.1, 2.5 Hz, 1H), 3.26 (dd, J = 5.7, 4.1 Hz, 1H), 3.08 (dd, J = 5.7, 2.5 Hz, 1H).

^{13}C NMR (126 MHz, $CDCl_3$) δ 152.6, 144.4, 144.2, 142.6, 51.2, 50.4.

HRMS (ESI): Calculated for $C_6H_6N_2O$ $[M+H]^+$: 122.0600; found:122.0602.

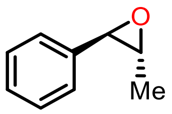

4k

rel-(2*R*,3*R*)-2-methyl-3-phenyloxirane, **4k**:
The reaction was conducted at room temperature for 1 h following general experimental procedure (section 2d). After the extraction, the product was obtained through a short celite plug using Et_2O as the eluent without further purification (24.9 mg, 0.186 mmol, 93% yield). The spectral data are in agreement with reported literature values.[10]
^{1}H NMR (500 MHz, $CDCl_3$) δ 7.37 – 7.22 (m, 5H), 3.58 (d, J = 2.1 Hz, 1H), 3.05 (qd, J = 5.2, 2.1 Hz, 1H), 1.46 (d, J = 5.2 Hz, 3H).
^{13}C NMR (126 MHz, $CDCl_3$) δ 137.8, 128.5, 128.1, 125.6, 59.6, 59.2, 18.0.

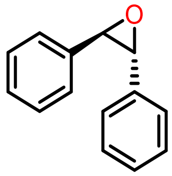

4l

rel-(2*R*,3*R*)-2,3-diphenyloxirane, **4l**:
The reaction was conducted at room temperature for 1 h following general experimental procedure (section 2d). After the extraction, the product was obtained through a short celite plug using Et_2O as the eluent without further purification (37.3 mg, 0.19 mmol, 95% yield). The spectral data are in agreement with reported literature values.[17]
^{1}H NMR (400 MHz, $CDCl_3$) δ 7.43 – 7.32 (m, 10H), 3.89 (s, 2H).
^{13}C NMR (101 MHz, CDCl3) δ 137.2, 128.7, 128.4, 125.6, 62.9.

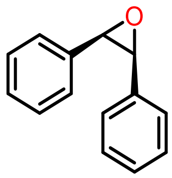

2c

rel-(2*R*,3*S*)-2,3-diphenyloxirane, **2c**:
The reaction was conducted at room temperature for 1 h following general experimental procedure (section 2d). After the extraction, the product was obtained through a short celite plug using Et_2O as the eluent without further purification (36.9 mg, 0.188 mmol, 94% yield). The spectral data are in agreement with reported literature values.[17]
^{1}H NMR (400 MHz, $CDCl_3$) δ 7.22 – 7.14 (m, 10H), 4.38 (s, 2H).
^{13}C NMR (101 MHz, $CDCl_3$) δ 134.4, 127.9, 127.6, 126.9, 59.8.

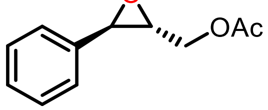

4m

rel-((2*R*,3*R*)-3-phenyloxiran-2-yl)methyl acetate, **4m**:
The reaction was conducted at room temperature for 1 h following general experimental procedure (section 2d). After the extraction, the product was obtained through a short celite plug using Et_2O as the eluent without further purification (36.9 mg, 0.192 mmol, 96% yield). The spectral data are in agreement with reported literature values.[10]
^{1}H NMR (500 MHz, $CDCl_3$) δ 7.37 – 7.30 (m, 3H), 7.28 – 7.26 (m, 2H), 4.49 (dd, J = 12.3, 3.3 Hz, 1H), 4.09 (dd, J = 12.3, 5.9 Hz, 1H), 3.81 (d, J = 2.1 Hz, 1H), 3.27 (ddd, J = 5.5, 3.3, 2.0 Hz, 1H), 2.12 (s, 3H).
^{13}C NMR (126 MHz, $CDCl_3$) δ 170.8, 136.2, 128.6, 128.5, 125.7, 64.2, 59.3, 56.5, 20.8.

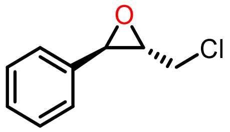

4n

rel-(2*S*,3*R*)-2-(chloromethyl)-3-phenyloxirane, **4n**:
The reaction was conducted at room temperature for 1 h following general experimental procedure (section 2d). After the extraction, the product was obtained through a short celite plug using Et_2O as the eluent without further purification (30.7 mg, 0.182 mmol, 91% yield). The spectral data are in agreement with reported literature values.[10]
^{1}H NMR (500 MHz, $CDCl_3$) δ 7.39 – 7.33 (m, 3H), 7.30 – 7.27 (m, 2H), 3.84 (d, J = 1.9 Hz, 1H), 3.74 (dd, J = 11.8, 4.7 Hz, 1H), 3.68 (dd, J = 11.8, 5.9 Hz, 1H), 3.31 (ddd, J = 5.8, 4.8, 1.9 Hz, 1H).
^{13}C NMR (126 MHz, $CDCl_3$) δ 136.0, 128.7, 128.7, 125.7, 61.1, 58.7, 44.5.

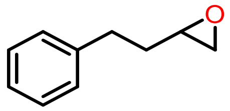

2a

2-phenethyloxirane, **2a**:
Compound **2a** was prepared using 4-phenyl-1-butene (26.4 mg, 0.2 mmol), Fe(TPFPP)Cl (0.2 mol%), NaOCl (0.22 mmol of OCl^-;1.1 equiv), and MeCN (0.5 mL). The reaction was conducted at room temperature for 6 h. After the extraction, the product was obtained through a short celite plug using Et_2O as the eluent without further purification (27.3 mg, 0.184 mmol, 92% yield). The spectral data are in agreement with reported literature values.[13]
^{1}H NMR (400 MHz, $CDCl_3$) δ 7.36 – 7.28 (m, 2H), 7.25 – 7.20 (m, 3H), 3.01 – 2.93 (m, 1H), 2.90 – 2.73 (m, 3H), 2.49 (dd, J = 5.0, 2.7 Hz, 1H), 1.94 – 1.80 (m, 2H).
^{13}C NMR (126 MHz, $CDCl_3$) δ 141.3, 128.5, 128.4, 126.0, 51.8, 47.3, 34.3, 32.3.

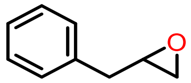

4p

2-benzyloxirane, **4p**:
Compound **4p** was prepared using allylbenzene (26.5 μL, 23.6 mg, 0.2 mmol), Fe(TPFPP)Cl (0.2 mol%), NaOCl (0.22 mmol of OCl^-;1.1 equiv), and MeCN (0.5 mL). The reaction was conducted at room temperature for 4 h. After the extraction, the product was obtained through a short celite plug using Et_2O as the eluent without further purification (25.7 mg, 0.192 mmol, 96% yield). The spectral data are in agreement with reported literature values.[18]

^{1}H NMR (500 MHz, $CDCl_3$) δ 7.34 – 7.30 (m, 2H), 7.27 – 7.24 (m, 3H), 3.19 – 3.14 (m, 1H), 2.93 (dd, J = 14.5, 5.7 Hz, 1H), 2.85 – 2.79 (m, 2H), 2.56 (dd, J = 5.0, 2.7 Hz, 1H).
^{13}C NMR (126 MHz, $CDCl_3$) δ 137.2, 129.1, 128.6, 126.7, 52.6, 47.0, 38.8.

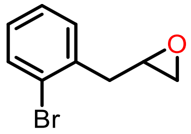

2b

2-(2-bromobenzyl)oxirane, **2b**:
Compound **2b** was prepared using 1-allyl-2-bromobenzene (39.4 mg, 0.2 mmol), Fe(TPFPP)Cl (0.2 mol%), NaOCl (0.22 mmol of OCl^-;1.1 equiv), and MeCN (0.5 mL). The reaction was conducted at room temperature for 4 h. After the extraction, the product was obtained through a short celite plug using Et_2O as the eluent without further purification (37.9 mg, 0.178 mmol, 89% yield). The spectral data are in agreement with reported literature values.[16]
^{1}H NMR (500 MHz, $CDCl_3$) δ 7.56 (dd, J = 8.0, 1.3 Hz, 1H), 7.32 (dd, J = 7.7, 1.9 Hz, 1H), 7.27 (td, J = 7.4, 1.3 Hz, 1H), 7.12 (td, J = 7.5, 1.8 Hz, 1H), 3.26 – 3.23 (m, 1H), 3.04 – 3.03 (m, 2H), 2.81 (dd, J = 5.0, 3.8 Hz, 1H), 2.57 (dd, J = 4.9, 2.7 Hz, 1H).
^{13}C NMR (126 MHz, $CDCl_3$) δ 136.8, 132.8, 131.2, 128.5, 127.6, 124.7, 51.3, 47.1, 38.7.

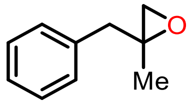

4q

2-benzyl-2-methyloxirane, **4q**:
Compound **4q** was prepared using (2-methylallyl)benzene (30 µL, 26.4 mg, 0.2 mmol), Fe(TPFPP)Cl (0.05 mol%), NaOCl (0.22 mmol of OCl^-;1.1 equiv), and MeCN (1.0 mL). The reaction was conducted at room temperature for 4 h. After the extraction, the product was obtained through a short celite plug using Et_2O as the eluent without further purification (27.8 mg, 0.188 mmol, 94% yield). The spectral data are in agreement with reported literature values.[19]
^{1}H NMR (500 MHz, $CDCl_3$) δ 7.33 – 7.28 (m, 2H), 7.27 – 7.21 (m, 3H), 2.95 – 2.77 (m, 2H), 2.69 – 2.59 (m, 2H), 1.29 (s, 3H).
^{13}C NMR (126 MHz, $CDCl_3$) δ 137.3, 129.6, 128.4, 126.6, 57.4, 53.4, 43.1, 20.9.

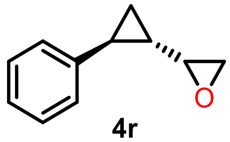

4r

rel-2-((1*S*,2*S*)-2-phenylcyclopropyl)oxirane, **4r**:
trans-2-phenyl-1-vinylcyclopropane was synthesized by following previously reported procedure.[20]
Compound **4r** was prepared using *trans*-2-phenyl-1-vinylcyclopropane (28.8 mg, 0.2 mmol), Fe(TPFPP)Cl (0.2 mol%), NaOCl (0.22 mmol of OCl^-;1.1 equiv), and MeCN (0.5 mL). The reaction was conducted at room temperature for 1 h. After the extraction, the product was obtained through a short celite plug using Et_2O as the eluent without further purification (31.1 mg, 0.194 mmol, 97% yield). The spectral data are in agreement with reported literature values.[21,22] The diastereomeric ratio was determined by ^{13}C NMR by comparing the equal intensities of the signals corresponding to the two isomers.

^{1}H NMR (500 MHz, $CDCl_3$) δ 7.30 – 7.24 (m, 2H), 7.20 – 7.14 (m, 1H), 7.07 (dt, J = 8.0, 1.6 Hz, 2H), 3.01 – 2.93 (m, 1H), 2.81 (dt, J = 5.0, 3.6 Hz, 1H), 2.61 (dt, J = 5.0, 2.8 Hz, 1H), 2.01 – 1.89 (m, 1H), 1.34 – 1.21 (m, 1H), 1.10 – 0.91 (m, 2H).
^{13}C NMR (126 MHz, $CDCl_3$) δ 142.18, 142.04, 128.50, 128.45, 126.06, 125.87, 53.26, 53.00, 47.10, 46.73, 23.52, 23.23, 20.37, 19.91, 12.54, 12.08.

Note: The 1:1 diastereoselectivity was determined by comparing the intensities of the ^{13}C NMR peaks of the diastereomers.

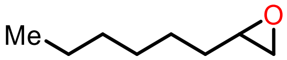

4s

2-hexyloxirane, **4s**:
Compound **4r** was prepared using 1-octene (22.4 mg, 0.2 mmol), Fe(TPFPP)Cl (0.2 mol%), NaOCl (0.22 mmol of OCl^-;1.1 equiv), and CH_3CN (0.5 mL). The reaction was conducted at room temperature for 6 h. After the extraction, the product was obtained through a short celite plug using Et_2O as the eluent without further purification (23.5 mg, 0.184 mmol, 92% yield). The spectral data are in agreement with reported literature values.[23]
^{1}H NMR (500 MHz, $CDCl_3$) δ 2.92 – 2.88 (m, 1H), 2.74 (dd, J = 5.1, 4.0 Hz, 1H), 2.46 (dd, J = 5.0, 2.8 Hz, 1H), 1.54 – 1.49 (m, 2H), 1.48 – 1.39 (m, 2H), 1.35 – 1.27 (m, 6H), 0.87 (t, J = 7.0 Hz, 3H).
^{13}C NMR (126 MHz, $CDCl_3$) δ 52.5, 47.3, 32.6, 31.8, 29.2, 26.0, 22.6, 14.2.

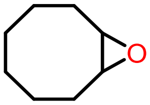

4t

9-oxabicyclo[6.1.0]nonane, **4t**:
Compound **4s** was prepared using *cis*-cyclooctene (26 μL, 0.2 mmol), Fe(TPFPP)Cl (0.05 mol%), NaOCl (0.22 mmol of OCl^-;1.1 equiv), and MeCN (1.0 mL). The reaction was conducted at room temperature for 2 h. After the extraction, the product was obtained through a short celite plug using Et_2O as the eluent without further purification (23.7 mg, 0.188 mmol, 94% yield). The spectral data are in agreement with reported literature values.[23]
^{1}H NMR (500 MHz, CDCl3) δ 2.93 – 2.85 (m, 2H), 2.18 – 2.07 (m, 2H), 1.65 – 1.57 (m, 2H), 1.56 – 1.49 (m, 2H), 1.49 – 1.38 (m, 4H), 1.32 – 1.20 (m, 2H).
^{13}C NMR (126 MHz, CDCl3) δ 55.7, 26.6, 26.3, 25.7.

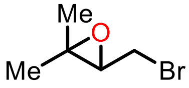

4u

3-(bromomethyl)-2,2-dimethyloxirane, **4u**:
Compound **4t** was prepared using 1-bromo-3-methylbut-2-ene (29.8 mg, 0.2 mmol), Fe(TPFPP)Cl (0.2 mol%), NaOCl (0.22 mmol of OCl^-;1.1 equiv), and MeCN (0.5 mL). The reaction was conducted at room temperature for 4 h. After the extraction, the product was obtained through a short celite plug using Et_2O as the eluent without further purification. Because of the compound's volatility, the yield was determined

by ^{1}H NMR spectroscopy of the crude reaction mixture using CH_2Br_2 as an internal standard (NMR yield: 95%).
^{1}H NMR (500 MHz, $CDCl_3$) δ 3.45 (dd, J = 10.6, 6.4 Hz, 1H), 3.25 (dd, J = 10.5, 7.2 Hz, 1H), 3.04 (t, J = 6.8 Hz, 1H), 1.31 (s, 3H), 1.27 (s, 3H).
^{13}C NMR (126 MHz, $CDCl_3$) δ 62.2, 60.7, 30.0, 24.3, 18.1.
HRMS (ESI): Calculated for $C_5H_{10}BrO$ $[M+H]^+$: 164.9910; found: 164.9910.

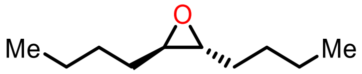

2d

rel-(2*R*,3*R*)-2,3-dibutyloxirane, **2d**:
Compound **2d** was prepared following using *trans*-5-decene (28.0 mg, 0.2 mmol), Fe(TPFPP)Cl (0.05 mol%), NaOCl (0.22 mmol of OCl^-;1.1 equiv), and MeCN (1.0 mL). The reaction was conducted at room temperature for 3 h. After the extraction, the product was obtained through a short celite plug using Et_2O as the eluent without further purification (29.1 mg, 0.186 mmol, 93% yield). The spectral data are in agreement with reported literature values.[24]
^{1}H NMR (500 MHz, $CDCl_3$) δ 2.66 – 2.61 (m, 2H), 1.54 – 1.30 (m, 12H), 0.89 (t, J = 7.1 Hz, 6H).
^{13}C NMR (126 MHz, $CDCl_3$) δ 59.0, 31.9, 28.2, 22.6, 14.1.

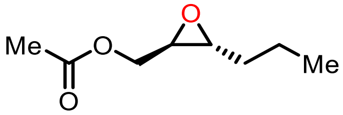

2e

rel-((2*R*,3*R*)-3-propyloxiran-2-yl)methyl acetate, **2e**:
Compound **2e** was prepared using *trans*-2-hexenyl acetate (28.4 mg, 0.2 mmol), Fe(TPFPP)Cl (0.05 mol%), NaOCl (0.22 mmol of OCl^-;1.1 equiv), and MeCN (1.0 mL). The reaction was conducted at room temperature for 3 h. After the extraction, the product was obtained through a short celite plug using Et_2O as the eluent without further purification (28.5 mg, 0.18 mmol, 90% yield). The spectral data are in agreement with reported literature values.[25]
^{1}H NMR (500 MHz, $CDCl_3$) δ 4.25 – 4.17 (m, 2H), 3.02 – 2.99 (m, 1H), 2.91 – 2.88 (m, 1H), 2.04 (s, 3H), 1.92 – 1.85 (m, 1H), 1.83 – 1.76 (m, 1H), 1.59 – 1.45 (m, 2H), 1.03 (t, J = 7.5 Hz, 3H).
^{13}C NMR (126 MHz, $CDCl_3$) δ 171.1, 61.9, 58.1, 54.4, 27.4, 21.2, 21.0, 10.6.

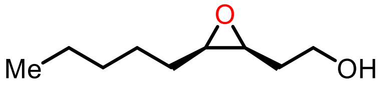

2f

rel-2-((2*S*,3*R*)-3-pentyloxiran-2-yl)ethan-1-ol, **2f**:
Compound **2f** was prepared using *cis*-3-nonen-1-ol (33.8 μL, 0.2 mmol), Fe(TPFPP)Cl (0.05 mol%), NaOCl (0.22 mmol of OCl^-;1.1 equiv), and MeCN (1.0 mL). The reaction was conducted at room temperature for 3 h. After the extraction, the product was obtained through a short celite plug using Et_2O as the eluent without further purification (31.0 mg, 0.196 mmol, 98% yield).
^{1}H NMR (500 MHz, $CDCl_3$) δ 3.89 – 3.73 (m, 2H), 3.13 – 3.04 (m, 1H), 3.00 – 2.88 (m, 1H), 1.93 – 1.80 (m, 1H), 1.72 – 1.61 (m, 1H), 1.55 – 1.46 (m, 3H), 1.34 – 1.26 (m, 5H), 0.90 – 0.85 (m, 3H).
^{13}C NMR (126 MHz, $CDCl_3$) δ 60.7, 56.9, 55.1, 31.7, 30.6, 27.9, 26.2, 22.6, 14.0.
HRMS (ESI): Calculated for $C_9H_{19}O_2$ $[M+H]^+$: 159.1380; found: 159.1379.

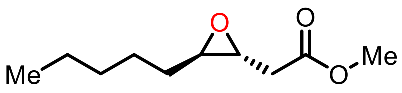

4v

rel-methyl 2-((2R,3R)-3-pentyloxiran-2-yl)acetate, **4v**:
Compound **4u** was prepared using methyl *trans*-3-nonenoate (34.05 mg, 0.2 mmol), Fe(TPFPP)Cl (0.05 mol%), NaOCl (0.22 mmol of OCl^-;1.1 equiv), and MeCN (1.0 mL). The reaction was conducted at room temperature for 6 h. After the extraction, the product was obtained through a short celite plug using Et_2O as the eluent without further purification (33.9 mg, 0.182 mmol, 91% yield). The spectral data are in agreement with reported literature values.[26]
^{1}H NMR (500 MHz, $CDCl_3$) δ 3.70 (s, 3H), 3.02 (td, J = 5.9, 2.1 Hz, 1H), 2.73 (td, J = 5.7, 2.1 Hz, 1H), 2.55 (dd, J = 8.6, 6.0 Hz, 2H), 1.58 – 1.50 (m, 2H), 1.47 – 1.39 (m, 2H), 1.32 – 1.27 (m, 4H), 0.86 (t, J = 5.0 Hz, 3H).
^{13}C NMR (101 MHz, $CDCl_3$) δ 170.99, 58.74, 53.99, 51.98, 37.68, 31.73, 31.60, 25.57, 22.61, 14.05.

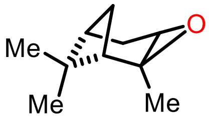

4w

rel-(1*S*,2*S*,4*S*,6*S*)-2,7,7-trimethyl-3-oxatricyclo[4.1.1.02,4]octane, **4w**:

Compound **4v** was prepared using (1*R*)-(+)-α-pinene (27.2 mg, 0.2 mmol), Fe(TPFPP)Cl (0.05 mol%), NaOCl (0.22 mmol of OCl^-; 1.1 equiv), and MeCN (0.5 mL). The reaction was conducted at room temperature for 2 h. After the extraction, the product was obtained through a short celite plug using Et_2O as the eluent without further purification (28.9 mg, 0.19 mmol, 95% yield). The spectral data are in agreement with reported literature values.[8]

^{1}H NMR (500 MHz, $CDCl_3$) δ 3.07 (d, J = 4.3 Hz, 1H), 2.03 – 1.95 (m, 2H), 1.95 – 1.86 (m, 2H), 1.73 – 1.68 (m, 1H), 1.59 (d, J = 9.6 Hz, 1H), 1.33 (s, 3H), 1.27 (s, 3H), 0.92 (s, 3H).
^{13}C NMR (126 MHz, $CDCl_3$) δ 60.5, 57.0, 45.1, 40.6, 39.7, 27.7, 26.8, 25.9, 22.5, 20.2.

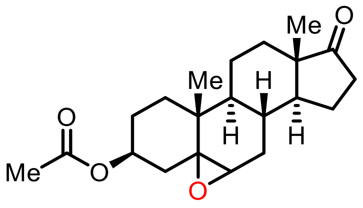

4x

rel-(3*S*,6a*R*,6b*S*,9a*S*,11a*S*,11b*R*)-9a,11b-dimethyl-9-oxohexadecahydrocyclopenta[1,2]phenanthro[8a,9-b]oxiren-3-yl acetate, **4x**:
Compound **4w** was prepared using dehydroisoandrosterone 3-acetate (66.0 mg, 0.2 mmol), Fe(TPFPP)Cl (0.05 mol%), NaOCl (0.22 mmol of OCl^-; 1.1 equiv), and MeCN (1.0 mL). The reaction was conducted at room temperature for 3 h. After the extraction, the product was obtained through a short celite plug using Et_2O as the eluent without further purification (64.4 mg, 0.186 mmol, 93% yield; d.r. = *α:β* = 1:3.6, *β*-epoxide is the major isomer). The diastereomeric ratio (d.r.) was determined using the signal at 4.78–4.63

ppm (m, 1H) in the ^{1}H NMR spectrum in $CDCl_3$. The spectral data are in agreement with reported literature values.[27]
^{1}H NMR (500 MHz, $CDCl_3$) δ 4.78 – 4.63 (m, 1H), 3.10 (d, J = 2.7 Hz, 1H), 2.40 (dd, J = 18.9, 8.6 Hz, 1H), 2.18 – 2.00 (m, 4H), 1.98 (s, 3H), 1.96 – 1.85 (m, 3H), 1.83 – 1.07 (m, 19H), 0.98 (s, 3H), 0.79 (s, 3H), 0.64 (td, J = 11.6, 11.0, 4.3 Hz, 1H).

Note: In the regions 2.18 – 2.00, 1.96 – 1.85, and 1.83 – 1.07 ppm, the proton integration appears higher because peaks from a minor isomer merge in these areas.

^{13}C NMR (126 MHz, $CDCl_3$) δ 220.7, 170.5, 71.1, 63.2, 62.6, 51.1, 51.1, 47.4, 37.9, 36.7, 35.7, 35.3, 31.4, 31.4, 29.4, 27.1, 21.7, 21.3, 21.2, 17.1, 13.5.

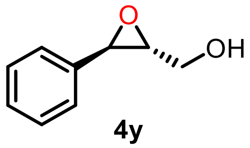

4y

((2*R*,3*R*)-3-phenyloxiran-2-yl)methanol, **4y**:
Compound **4x** was prepared using *trans*-cinnamyl alcohol (26.8 mg, 0.2 mmol), Fe(TPFPP)Cl (0.05 mol%), NaOCl (0.22 mmol of OCl^-; 1.1 equiv), and MeCN (1.0 mL). The reaction was conducted at room temperature for 2 h. After the extraction, the product was purified by silica-gel column chromatography using (Hexane:EtOAc 3:1) as an eluent (25.8 mg, 0.172 mmol, 86% yield). The spectral data are in agreement with reported literature value.[10]
^{1}H NMR (500 MHz, $CDCl_3$) δ 7.40 – 7.25 (m, 1H), 4.06 (ddd, J = 12.9, 5.0, 2.4 Hz, 0H), 3.94 (d, J = 2.3 Hz, 0H), 3.85 – 3.76 (m, 0H), 3.24 (dt, J = 3.9, 2.3 Hz, 0H).
^{13}C NMR (126 MHz, CDCl3) δ 136.7, 128.7, 128.5, 125.8, 62.6, 61.3, 55.7.

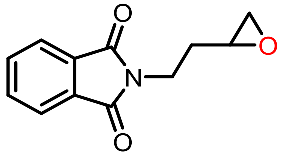

4z

2-(2-(oxiran-2-yl)ethyl)isoindoline-1,3-dione, **4z**:
Compound **4y** was prepared using 2-(but-3-en-1-yl)isoindoline-1,3-dione (40.2 mg, 0.2 mmol), Fe(TPFPP)Cl (0.2 mol%), NaOCl (0.22 mmol of OCl^-;1.1 equiv), and MeCN (0.5 mL). The reaction was conducted at room temperature for 6 h. After the extraction, the product was obtained through a short celite plug using Et_2O as the eluent without further purification (40.8 mg, 0.188 mmol, 94% yield).
^{1}H NMR (500 MHz, $CDCl_3$) δ 7.82 (dd, J = 5.5, 3.0 Hz, 2H), 7.69 (dd, J = 5.4, 3.1 Hz, 2H), 3.86 (qt, J = 13.7, 6.8 Hz, 1H), 3.00 – 2.94 (m, 1H), 2.71 – 2.67 (m, 1H), 2.42 (dd, J = 4.9, 2.6 Hz, 1H), 2.01 – 1.93 (m, 1H), 1.85 – 1.77 (m, 1H).
^{13}C NMR (126 MHz, $CDCl_3$) δ 168.3, 134.0, 132.0, 123.3, 50.2, 46.4, 35.1, 31.6.
HRMS (ESI): Calculated for $C_{12}H_{12}NO_3$ $[M+H]^+$: 218.0812; found: 218.0811.

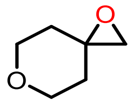

2g
1,6-dioxaspiro[2.5]octane, **2g**:

Compound **2g** was prepared using 4-methylenetetrahydro-2*H*-pyran (19.6 mg, 0.2 mmol), Fe(TPFPP)Cl (0.05 mol%), NaOCl (0.22 mmol of OCl^-;1.1 equiv), and MeCN (1.0 mL). The reaction was conducted at room temperature for 2 h. After the extraction, the product was obtained through a short celite plug using Et_2O as the eluent without further purification (19.4 mg, 0.17 mmol, 85% yield).
^{1}H NMR (500 MHz, $CDCl_3$) δ 3.90 – 3.83 (m, 2H), 3.83 – 3.78 (m, 2H), 2.69 (s, 2H), 1.90 – 1.82 (m, 2H), 1.56 – 1.48 (m, 2H).
^{13}C NMR (126 MHz, $CDCl_3$) δ 66.6, 56.6, 54.0, 33.9.
HRMS (ESI): Calculated for $C_6H_{11}O_2$ $[M+H]^+$: 115.0754; found: 115.0755.

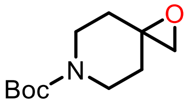

2h

tert-butyl 1-oxa-6-azaspiro[2.5]octane-6-carboxylate, **2h**:
Compound **2h** was prepared using *tert*-butyl 4-methylenepiperidine-1-carboxylate (41 µL, 39.4 mg, 0.2 mmol), Fe(TPFPP)Cl (0.05 mol%), NaOCl (0.22 mmol of OCl^-;1.1 equiv), and MeCN (1.0 mL). The reaction was conducted at room temperature for 2 h. After the extraction, the product was obtained through a short celite plug using Et_2O as the eluent without further purification (39.7 mg, 0.186 mmol, 93% yield).
^{1}H NMR (500 MHz, $CDCl_3$) δ 3.75 – 3.59 (m, 2H), 3.43 – 3.34 (m, 2H), 2.66 (s, 2H), 1.81 – 1.71 (m, 2H), 1.43 (s, 9H), 1.42 – 1.37 (m, 2H).
^{13}C NMR (126 MHz, $CDCl_3$) δ 154.8, 79.8, 57.2, 53.8, 42.8, 42.1, 32.9, 28.4.
HRMS (ESI): Calculated for $C_{11}H_{19}NNaO_3$ $[M+Na]^+$: 236.1257; found: 236.1256.

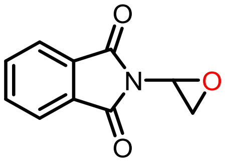

2l

2-(oxiran-2-yl)isoindoline-1,3-dione, **2l**:
Compound **2l** was prepared using 2-vinylisoindoline-1,3-dione (34.6 mg, 0.2 mmol), Fe(TPFPP)Cl (0.05 mol%), NaOCl (0.22 mmol of OCl^-;1.1 equiv), and MeCN (1.0 mL). The reaction was conducted at room temperature for 0.5 h. After the extraction, the product was obtained through a short celite plug using Et_2O as the eluent without further purification (34.8 mg, 0.184 mmol, 92% yield).
^{1}H NMR (500 MHz, $CDCl_3$) δ 7.87 – 7.83 (m, 2H), 7.76 – 7.73 (m, 2H), 4.88 (dd, J = 3.8, 2.1 Hz, 1H), 4.09 (dd, J = 4.6, 2.1 Hz, 1H), 3.09 – 3.06 (m, 1H).
^{13}C NMR (126 MHz, $CDCl_3$) δ 167.5, 134.7, 131.4, 123.7, 54.3, 44.5.
HRMS (ESI): Calculated for $C_{10}H_8NO_3$ $[M+H]^+$: 190.0499; found: 190.0496.

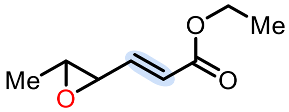

6a

ethyl (*E*)-3-(3-methyloxiran-2-yl)acrylate, **6a**:
Compound **6a** was prepared using ethyl sorbate (ethyl *trans*,*trans*-2,4-hexadienoate) (29.3 µL, 28 mg, 0.2 mmol), Fe(TPFPP)Cl (0.05 mol%), NaOCl (0.22 mmol of OCl^-;1.1 equiv), and MeCN (1.0 mL). The reaction was conducted at room temperature for 1 h. After the extraction, the product was obtained through

a short celite plug using Et_2O as the eluent without further purification (26.9 mg, 0.172 mmol, 86% yield; exclusive mono-epoxide selectivity). The spectral data are in agreement with reported literature values.[23]
^{1}H NMR (500 MHz, $CDCl_3$) δ 6.66 (dd, J = 15.7, 7.2 Hz, 1H), 6.11 (d, J = 15.7 Hz, 1H), 4.19 (q, J = 7.1 Hz, 2H), 3.17 (dd, J = 7.2, 2.3 Hz, 1H), 2.96 (qd, J = 5.2, 2.0 Hz, 1H), 1.37 (d, J = 5.2 Hz, 3H), 1.27 (t, J = 7.1 Hz, 3H).
^{13}C NMR (126 MHz, $CDCl_3$) δ 165.8, 144.7, 123.8, 60.7, 57.5, 57.3, 17.6, 14.3.

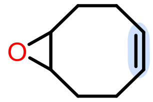

6b

(*Z*)-9-oxabicyclo[6.1.0]non-4-ene, **6b**:
Compound **6b** was prepared using 1,5-cyclooctadiene (216 mg, 2.0 mmol), Fe(TPFPP)Cl (0.05 mol%), NaOCl (0.22 mmol of OCl^-;1.1 equiv), and MeCN (10 mL). The reaction was conducted at room temperature for 1 h. After the extraction, the product was purified by silica-gel column chromatography using (Pentane:Et_2O 9:1) as an eluent. The TLC was visualized using a PMA stain (176.3 mg, 1.42 mmol, 71% yield). The spectral data are in agreement with reported literature values.[28]
^{1}H NMR (500 MHz, $CDCl_3$) δ 5.63 – 5.50 (m, 2H), 3.02 (q, J = 4.3 Hz, 2H), 2.51 – 2.39 (m, 2H), 2.19 – 1.96 (m, 6H).
^{13}C NMR (126 MHz, $CDCl_3$) δ 128.93, 56.84, 28.19, 23.79.
Note: Because the product is highly volatile, 2.0 mmol of substrate was used to obtain isolated yield. it is crucial to evaporate the solvent cautiously using a rotary evaporator (limit: 600 mbar at 23 °C). The distillate from the rotary evaporator was checked by TLC; if any product was detected, the solvent was re-concentrated to recover the lost material.

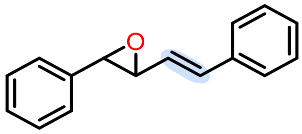

6c

(*E*)-2-phenyl-3-styryloxirane, **6c**:
Compound **6c** was prepared using *trans*,*trans*-1,4-diphenyl-1,3-butadiene (41.2 mg, 0.2 mmol), Fe(TPFPP)Cl (0.05 mol%), NaOCl (0.22 mmol of OCl^-;0.55 equiv to the mmol of total alkene), and a mixture of MeCN and CH_2Cl_2 (1:0.5 mL). The reaction was conducted at room temperature for 1 h. After the extraction, the product was obtained through a short celite plug using CH_2Cl_2 as the eluent without further purification (40.0 mg, 0.18 mmol, 90% yield; mono-epoxide:di-epoxide selectivity = 20:1).
^{1}H NMR (500 MHz, $CDCl_3$) δ 7.45 – 7.38 (m, 3H), 7.40 – 7.32 (m, 6H), 7.33 – 7.26 (m, 1H), 6.83 (d, J = 16.0 Hz, 1H), 6.09 (dd, J = 16.0, 7.7 Hz, 1H), 3.91 (d, J = 2.0 Hz, 1H), 3.55 (dd, J = 7.7, 1.9 Hz, 1H).
^{13}C NMR (126 MHz, $CDCl_3$) δ 137.0, 136.0, 134.5, 128.7, 128.6, 128.3, 128.2, 126.6, 126.2, 125.5, 63.2, 60.8.
HRMS (ESI): Calculated for $C_{16}H_{15}O$ $[M+H]^+$: 223.1117; found: 223.1117.

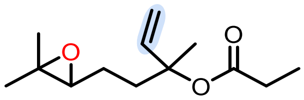

6d

5-(3,3-dimethyloxiran-2-yl)-3-methylpent-1-en-3-yl propionate, **6d**:
Compound **6d** was prepared using linalyl propionate (47 µL, 42 mg, 0.2 mmol), Fe(TPFPP)Cl (0.05 mol%), NaOCl (0.22 mmol of OCl^-;0.55 equiv to the mmol of total alkene), and MeCN (1.0 mL). After the

extraction, the product was obtained through a short celite plug using Et_2O as the eluent without further purification (38.5 mg, 0.17 mmol, 85% yield; exclusive mono-epoxide selectivity).

^{1}H NMR (500 MHz, $CDCl_3$) δ 5.92 (ddd, J = 23.2, 17.5, 11.0 Hz, 1H), 5.18 – 5.08 (m, 2H), 2.69 (td, J = 6.4, 2.7 Hz, 1H), 2.26 (qd, J = 7.6, 2.6 Hz, 2H), 1.98 (dddd, J = 13.4, 12.0, 4.9, 1.2 Hz, 1H), 1.85 (ddt, J = 13.7, 11.8, 4.4 Hz, 1H), 1.53 (d, J = 11.4 Hz, 3H), 1.28 (s, 3H), 1.23 (d, J = 1.6 Hz, 3H), 1.08 (td, J = 7.6, 1.5 Hz, 3H).

^{13}C NMR (126 MHz, $CDCl_3$) δ 173.3, 141.6, 141.4, 113.5, 113.5, 82.2, 82.1, 64.0, 58.5, 36.3, 36.3, 28.6, 28.5, 24.9, 23.8, 23.7, 23.5, 23.4, 18.6, 18.6, 9.2, 9.2.

HRMS (ESI): Calculated for $C_{13}H_{23}O_3$ $[M+H]^+$: 227.1642; found: 227.1638.

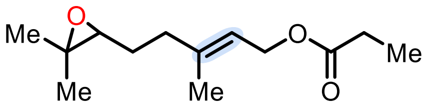

6e

(*E*)-5-(3,3-dimethyloxiran-2-yl)-3-methylpent-2-en-1-yl propionate, **6e**:

Compound **6e** was prepared using geranyl propionate (46.7 µL, 42 mg, 0.2 mmol), Fe(TPFPP)Cl (0.05 mol%), NaOCl (0.22 mmol of OCl^-;0.55 equiv to the mmol of total alkene), and MeCN (1.0 mL). The reaction was conducted at room temperature for 2 h. After the extraction, the product was obtained through a short celite plug using Et_2O as the eluent without further purification (35.8 mg, 0.158 mmol, 79% yield; mono-epoxide:di-epoxide selectivity = 6:1). The spectral data are in agreement with reported literature values.[29]

^{1}H NMR (500 MHz, $CDCl_3$) δ 5.35 (tq, J = 7.1, 1.3 Hz, 1H), 4.57 (d, J = 7.0 Hz, 2H), 2.67 (t, J = 6.2 Hz, 1H), 2.30 (q, J = 7.6 Hz, 2H), 2.25 – 2.07 (m, 2H), 1.70 (s, 3H), 1.67 – 1.60 (m, 2H), 1.27 (s, 3H), 1.23 (s, 3H), 1.11 (t, J = 7.6 Hz, 3H).

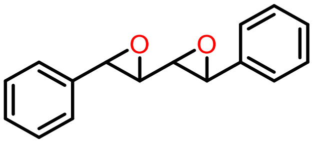

6c'

3,3'-diphenyl-2,2'-bioxirane, **6c'**:

Compound **6c'** was prepared using *trans*,*trans*-1,4-diphenyl-1,3-butadiene (41.2 mg, 0.2 mmol, 1.0 equiv), Fe5 (0.05 mol%), NaOCl (0.44 mmol of OCl^-; 1.1 equiv to the mmol of total alkene), and a mixture of MeCN and CH_2Cl_2 (1:0.5 mL). The reaction was conducted at room temperature for 2 h. After the extraction, the product was obtained through a short celite plug using CH_2Cl_2 as the eluent without further purification (43.8 mg, 0.184 mmol, 92% yield).

^{1}H NMR (400 MHz, $CDCl_3$) δ 7.41 – 7.34 (m, 7H), 7.34 – 7.29 (m, 3H), 3.96 (d, J = 11.2 Hz, 2H), 3.21 – 3.18 (m, 2H).

^{13}C NMR (101 MHz, CDCl3) δ 136.3, 136.1, 128.7, 128.6, 128.6, 128.6, 125.7, 125.7, 60.6, 60.2, 56.7, 56.0.

HRMS (ESI): Calculated for $C_{13}H_{23}O_3$ $[M+H]^+$: 239.1967; found: 239.1064.

Note: The 1:1 diastereoselectivity was determined by comparing the intensities of the ^{13}C NMR peaks of the diastereomers.

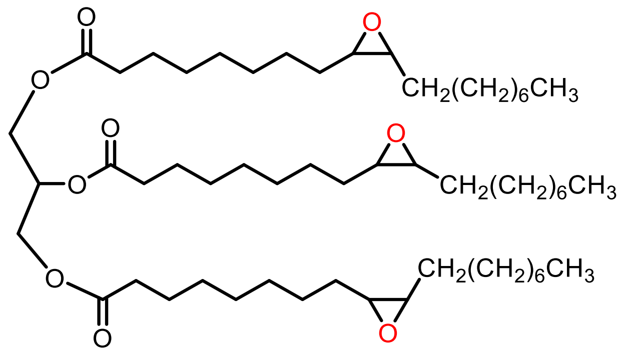

6f

propane-1,2,3-triyl tris(8-(3-octyloxiran-2-yl)octanoate), **6f**:
Compound **6f** was prepared using glyceryl trioleate (177.0 mg, 0.2 mmol), Fe(TPFPP)Cl (0.05 mol%), NaOCl (0.66 mmol of OCl^-; 1.1 equiv to the mmol of total alkene), and a mixture of MeCN and CH_2Cl_2 (1:0.5 mL). The reaction was conducted at room temperature for 3 h. After the extraction, the product was obtained through a short celite plug using CH_2Cl_2 as the eluent without further purification (177.4 mg, 0.19 mmol, 95% yield). The spectral data are in agreement with reported literature values.[30]
^{1}H NMR (500 MHz, $CDCl_3$) δ 5.24 – 5.14 (m, 1H), 4.23 (dd, J = 12.1, 5.0 Hz, 2H), 4.08 (dd, J = 12.2, 6.2 Hz, 2H), 2.87 (bs, 6H), 2.25 (t, J = 7.8 Hz, 6H), 1.58 – 1.16 (m, 78H), 0.81 (t, J = 6.9 Hz, 9H).
^{13}C NMR (126 MHz, $CDCl_3$) δ 173.1, 172.7, 68.8, 62.0, 57.0, 56.9, 34.0, 33.9, 31.8, 29.5, 29.4, 29.2, 29.1, 29.1, 28.9, 28.8, 27.8, 27.7, 26.5, 26.5, 24.7, 24.7, 22.6, 14.0.

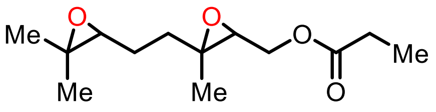

6e'

(3-(2-(3,3-dimethyloxiran-2-yl)ethyl)-3-methyloxiran-2-yl)methyl propionate, **6e'**:
Compound **6e'** was prepared using geranyl propionate (47 µL, 42 mg, 0.2 mmol), Fe5 (0.05 mol%), NaOCl (0.44 mmol of OCl^-; 1.1 equiv to the mmol of total alkene), and MeCN (1.0 mL). The reaction was conducted at room temperature for 2 h. After the extraction, the product was obtained through a short celite plug using CH_2Cl_2 as the eluent without further purification (43.6 mg, 0.18 mmol, 90% yield).
^{1}H NMR (500 MHz, $CDCl_3$) δ 4.30 (ddd, J = 12.1, 10.2, 4.3 Hz, 1H), 4.03 (ddd, J = 12.1, 6.8, 2.7 Hz, 1H), 2.99 (dd, J = 6.8, 4.3 Hz, 1H), 2.70 – 2.66 (m, 1H), 2.35 (q, J = 7.5 Hz, 2H), 1.82 – 1.53 (m, 4H), 1.30 (d, J = 6.1 Hz, 3H), 1.28 (s, 3H), 1.24 (d, J = 4.0 Hz, 3H), 1.12 (t, J = 7.2 Hz, 3H).
^{13}C NMR (126 MHz, $CDCl_3$) δ 174.4, 63.8, 63.6, 63.2, 63.1, 60.3, 60.2, 59.9, 59.4, 58.5, 58.45, 35.2, 34.8, 27.4, 24.8, 24.6, 24.4, 18.7, 18.7, 17.0, 16.8, 9.1.

Note: The 1:1 diastereoselectivity was determined by comparing the intensities of the ^{13}C NMR peaks of the diastereomers. The chemical shift values for all carbons corresponding to both diastereomers are reported. The spectral data are in agreement with reported literature values.[31]

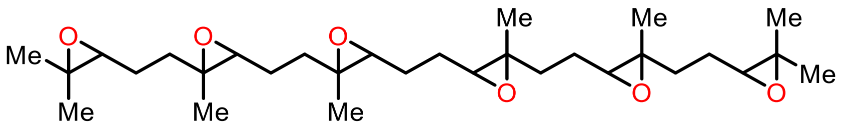

6g

1,2-bis(3-(2-(3-(2-(3,3-dimethyloxiran-2-yl)ethyl)-3-methyloxiran-2-yl)ethyl)-3 methyloxiran-2-yl)ethane, **6g**:

Compound **6g** was prepared using squalene (82.1 mg, 0.2 mmol), Fe(TPFPP)Cl (0.05 mol%), NaOCl (1.32 mmol of OCl^-; 1.1 equiv to the mmol of total alkene), and a mixture of MeCN and CH_2Cl_2 (1:0.5 mL). The reaction was conducted at room temperature for 3 h. The product was obtained after the extractions as an oil and as a mixture of diastereomeric hexaepoxides (92.2 mg, 0.182 mmol, 91% yield).

^{1}H NMR (500 MHz, $CDCl_3$) δ 2.78 – 2.58 (m, 6H), 1.76 – 1.46 (m, 20H), 1.26 – 1.18 (m, 24H).

^{13}C NMR (126 MHz, $CDCl_3$) δ 63.9, 63.7, 63.3, 63.3, 63.2, 63.2, 63.2, 63.1, 62.9, 62.9, 62.9, 62.8, 62.7, 62.7, 62.6, 62.4, 62.4, 62.3, 60.5, 60.5, 60.4, 60.4, 60.3, 60.3, 58.4, 58.4, 35.5, 35.5, 35.5, 35.4, 35.1, 35.1, 35.1, 35.0, 25.9, 25.7, 24.8, 24.7, 24.5, 24.5, 24.5, 24.4, 24.3, 24.3, 24.3, 24.3, 18.6, 18.6, 16.6, 16.6, 16.6, 16.5, 16.4, 16.3, 16.3.

Note: Global epoxidation of all the alkene bonds of squalene was confirmed by ^{1}H NMR analysis that revealed the new arising epoxide signal at 2.78 – 2.58 ppm (m, 6H) as a broad multiplet (multiple hexaepoxy diastereomers) and the complete disappearance of the alkene signal at 5.20 – 5.05 ppm (m, 6H).[32,33]

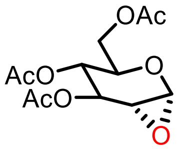

2i

1,2-Anhydro-3,4,6-tri-*O*-acetyl-*α*-D-glucopyranose, **2i:**

Compound **2i** was prepared using tri-*O*-acetyl-D-glucal (54.4 mg, 0.2 mmol), Fe(TPFPP)Cl (0.05 mol%), NaOCl (0.22 mmol of OCl^-; 1.1 equiv), and MeCN (1.0 mL). The reaction was conducted at room temperature for 15 min. The product was obtained after the extractions as an oil (51.8 mg, 0.18 mmol, 90% yield; α:β = 8:1). The diastereomeric ratio (d.r.) was determined using the signal at 2.99 ppm (d, J = 2.2 Hz, 1H) in the ^{1}H NMR spectrum in $CDCl_3$.

^{1}H NMR (500 MHz, $CDCl_3$) δ 5.19 (dd, J = 8.2, 1.1 Hz, 1H), 5.03 – 4.96 (m, 2H), 4.30 (dd, J = 12.4, 4.0 Hz, 1H), 4.03 (dd, J = 12.4, 2.2 Hz, 1H), 3.95 (ddd, J = 10.5, 4.1, 2.2 Hz, 1H), 2.99 (d, J = 2.2 Hz, 1H), 2.06 (s, 3H), 2.04 (s, 3H), 2.00 (s, 3H).

^{13}C NMR (126 MHz, $CDCl_3$) δ 170.7, 170.0, 169.7, 77.1, 70.0, 66.5, 65.9, 61.2, 52.4, 20.8, 20.7, 20.7.

HRMS (ESI): Calculated for $C_{12}H_{17}O_8$ $[M+H]^+$: 289.0918; found: 289.0914.

Note: The stereochemical orientation of epoxide was established by methanolysis.

General procedure for methanolysis of **2i**: The 1,2-anhydro sugar (0.1 mmol) was dissolved in 1.0 mL of anhydrous MeOH, and the solution was stirred at room temperature for 2 h. The MeOH was removed in vacuo to afford a quantitative yield of methyl glycoside(s).

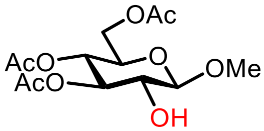

Methyl 2-hydroxy-3,4,6-tri-*O*-acetyl-*β*-D-glucopyranoside:

^{1}H NMR (500 MHz, $CDCl_3$) δ 5.08 (t, J = 9.5 Hz, 1H), 4.99 (t, J = 9.7 Hz, 1H), 4.28 – 4.23 (m, 2H), 4.08 (dd, J = 12.3, 2.4 Hz, 1H), 3.67 (ddd, J = 9.9, 4.7, 2.3 Hz, 1H), 3.54 (s, 3H), 3.52 (dd, J = 7.2, 2.2 Hz, 1H), 2.05 (s, 3H), 2.04 (s, 3H), 2.00 (s, 3H).

^{13}C NMR (126 MHz, $CDCl_3$) δ 170.92, 169.82, 103.82, 74.53, 72.08, 71.78, 68.41, 62.07, 57.53, 20.88, 20.82, 20.71.

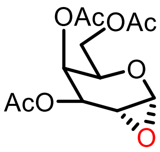

6h

1,2-Anhydro-3,4,6-tri-*O*-acetyl-*α*-D-galactopyranose, **6h:**

Compound **6h** was prepared using tri-O-acetyl-D-galactal (54.4 mg, 0.2 mmol), Fe(TPFPP)Cl (0.05 mol%), NaOCl (0.22 mmol of OCl^-; 1.1 equiv), and MeCN (1.0 mL). The reaction was conducted at room temperature for 15 min. The product was obtained after the extractions as an oil. The diastereomeric ratio (d.r.) was determined using the signal at 2.97 ppm (52.5 mg, 0.182 mmol, 91% yield; α:β >20:1).

^{1}H NMR (500 MHz, $CDCl_3$) δ 5.21 – 5.18 (m, 1H), 5.14 (d, J = 5.3 Hz, 1H), 5.03 (d, J = 3.0 Hz, 1H), 4.09 – 4.06 (m, 2H), 3.99 (t, J = 6.6 Hz, 1H), 2.98 – 2.95 (m, 1H), 2.10 (s, 3H), 2.04 (s, 3H), 2.03 (s, 3H).

^{13}C NMR (126 MHz, $CDCl_3$) δ 170.5, 170.2, 169.4, 76.8, 67.0, 66.5, 62.0, 61.6, 50.1, 20.7, 20.6, 20.6.

HRMS (ESI): Calculated for $C_{12}H_{17}O_8$ $[M+H]^+$: 289.0918; found: 289.0913.

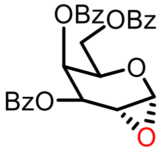

6i

1,2-Anhydro-3,4,6-tri-*O*-benzoyl-*a*-D-galactopyranose, **6i**:

Compound **6i** was prepared using tri-*O*-benzoyl-D-galactal (91.6 mg, 0.2 mmol), Fe(TPFPP)Cl (0.05 mol%), NaOCl (0.22 mmol of OCl^-; 1.1 equiv), and MeCN (1.0 mL). The reaction was conducted at room temperature for 15 min. The product was obtained after the extractions as an oil (83.5 mg, 0.176 mmol, 88% yield; α–exclusive).

^{1}H NMR (500 MHz, $CDCl_3$) δ 8.07 – 7.98 (m, 4H), 7.88 – 7.84 (m, 2H), 7.61 (t, J = 7.5 Hz, 1H), 7.56 – 7.49 (m, 2H), 7.46 (t, J = 7.8 Hz, 2H), 7.40 (t, J = 7.8 Hz, 2H), 7.31 (t, J = 7.8 Hz, 2H), 5.78 (dd, J = 4.4, 1.8 Hz, 1H), 5.59 (d, J = 4.3 Hz, 1H), 5.26 (d, J = 2.3 Hz, 1H), 4.64 – 4.57 (m, 1H), 4.44 – 4.38 (m, 2H), 3.23 – 3.21 (m, 1H).

^{13}C NMR (126 MHz, $CDCl_3$) δ 166.0, 165.5, 165.0, 133.6, 133.5, 133.3, 129.9, 129.8, 129.3, 129.1, 128.8, 128.6, 128.4, 76.9, 67.4, 67.3, 62.6, 62.5, 50.3.

HRMS (ESI): Calculated for $C_{27}H_{23}O_8$ $[M+H]^+$: 475.1387; found: 475.1385.

Note: The stereochemical orientation of epoxide was established by methanolysis.

General procedure for methanolysis of **6i**: The 1,2-anhydro sugar (0.1 mmol) was dissolved in 1.0 mL of anhydrous MeOH, and the solution was stirred at room temperature for 2 h. The MeOH was removed in vacuo to afford a quantitative yield of methyl glycoside(s).

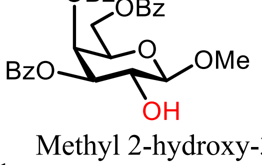

Methyl 2-hydroxy-3,4,6-tri-*O*-benzoyl-*β*-D-galactopyranoside:

^{1}H NMR (500 MHz, $CDCl_3$) δ 8.07 (d, J = 6.8 Hz, 2H), 8.02 (d, J = 6.9 Hz, 2H), 7.85 (d, J = 6.9 Hz, 2H), 7.63 – 7.57 (m, 1H), 7.54 (t, J = 7.4 Hz, 1H), 7.49 – 7.44 (m, 3H), 7.41 (t, J = 7.8 Hz, 2H), 7.30 – 7.24 (m, 2H), 5.92 (d, J = 3.6 Hz, 1H), 5.42 (ddd, J = 10.2, 3.6, 1.7 Hz, 1H), 4.66 (dd, J = 11.3, 6.6 Hz, 1H), 4.48 (d, J = 7.7 Hz, 1H), 4.38 (dd, J = 11.3, 6.7 Hz, 1H), 4.26 (t, J = 6.7 Hz, 1H), 4.12 (dd, J = 10.2, 7.6 Hz, 1H), 3.64 (s, 3H).

^{13}**C NMR** (126 MHz, $CDCl_3$) δ 166.11, 166.01, 165.55, 133.60, 133.34, 133.29, 129.99, 129.83, 129.78, 129.41, 129.23, 129.19, 128.61, 128.51, 128.31, 104.33, 73.35, 71.20, 69.88, 68.22, 62.07, 57.61.

4. References

(1) IODOSOBENZENE. *Org. Synth.* **1963**, *43*, 60. https://doi.org/10.15227/orgsyn.043.0060.

(2) Hoffman, B. M.; Szymanski, T.; Brown, T. G.; Basolo, F. The Dioxygen Adducts of Several Manganese(II) Porphyrins. Electron Paramagnetic Resonance Studies. *J. Am. Chem. Soc.* **1978**, *100*, 7253–7259. https://doi.org/10.1021/ja00491a022.

(3) Cheshev, P.; Marra, A.; Dondoni, A. Direct Epoxidation of D-Glucal and d-Galactal Derivatives with in Situ Generated DMDO. *Carbohydrate Research* **2006**, *341*, 2714–2716. https://doi.org/10.1016/j.carres.2006.09.003.

(4) Meunier, B.; Guilmet, E.; De Carvalho, M. E.; Poilblanc, R. Sodium Hypochlorite: A Convenient Oxygen Source for Olefin Epoxidation Catalyzed by (Porphyrinato)Manganese Complexes. *J. Am. Chem. Soc.* **1984**, *106*, 6668–6676. https://doi.org/10.1021/ja00334a035.

(5) Brandes, B. D.; Jacobsen, E. N. Highly Enantioselective, Catalytic Epoxidation of Trisubstituted Olefins. *J. Org. Chem.* **1994**, *59*, 4378–4380. https://doi.org/10.1021/jo00095a009.

(6) Jacobsen, E. N.; Zhang, W.; Muci, A. R.; Ecker, J. R.; Deng, L. Highly Enantioselective Epoxidation Catalysts Derived from 1,2-Diaminocyclohexane. *J. Am. Chem. Soc.* **1991**, *113*, 7063–7064. https://doi.org/10.1021/ja00018a068.

(7) Sato, K.; Aoki, M.; Ogawa, M.; Hashimoto, T.; Noyori, R. A Practical Method for Epoxidation of Terminal Olefins with 30% Hydrogen Peroxide under Halide-Free Conditions. *J. Org. Chem.* **1996**, *61*, 8310–8311. https://doi.org/10.1021/jo961287e.

(8) Dong, J. J.; Saisaha, P.; Meinds, T. G.; Alsters, P. L.; Ijpeij, E. G.; van Summeren, R. P.; Mao, B.; Fañanás-Mastral, M.; de Boer, J. W.; Hage, R.; Feringa, B. L.; Browne, W. R. Oxidation of Alkenes with H_2O_2 by an in-Situ Prepared Mn(II)/Pyridine-2-Carboxylic Acid Catalyst and the Role of Ketones in Activating H_2O_2. *ACS Catal.* **2012**, *2*, 1087–1096. https://doi.org/10.1021/cs3002226.

(9) Moretti, R. A.; Du Bois, J.; Stack, T. D. P. Manganese(II)/Picolinic Acid Catalyst System for Epoxidation of Olefins. *Org. Lett.* **2016**, *18*, 2528–2531. https://doi.org/10.1021/acs.orglett.6b00518.

(10) Tse, M. K.; Klawonn, M.; Bhor, S.; Döbler, C.; Anilkumar, G.; Hugl, H.; Mägerlein, W.; Beller, M. Convenient Method for Epoxidation of Alkenes Using Aqueous Hydrogen Peroxide. *Org. Lett.* **2005**, *7*, 987–990. https://doi.org/10.1021/ol047604i.

(11) Li, J.; Li, S.; Cui, G.-H.; Yuan, K.; Wang, C.; Yang, C.; Zhang, G.; Guo, R. Photoinduced Copper-Catalyzed Regio- and Enantioselective Alkynylation of Epoxides. *ACS Catal.* **2024**, *14*, 7553–7561. https://doi.org/10.1021/acscatal.4c00818.

(12) Lv, S.; Zhang, Q.; Zhou, H. NaBr-Mediated Electrochemical Hydration of Alkenes: A Minimalist Synthesis of Anti-Markovnikov Alcohol. *Org. Lett.* **2025**, *27*, 10502–10506. https://doi.org/10.1021/acs.orglett.5c03365.

(13) Huang, C.; Ma, W.; Zheng, X.; Xu, M.; Qi, X.; Lu, Q. Epoxide Electroreduction. *J. Am. Chem. Soc.* **2022**, *144*, 1389–1395. https://doi.org/10.1021/jacs.1c11791.

(14) Vyas, D. J.; Larionov, E.; Besnard, C.; Guénée, L.; Mazet, C. Isomerization of Terminal Epoxides by a [Pd–H] Catalyst: A Combined Experimental and Theoretical Mechanistic Study. *J. Am. Chem. Soc.* **2013**, *135*, 6177–6183. https://doi.org/10.1021/ja400325w.

(15) Mikleušević, A.; Primožič, I.; Hrenar, T.; Salopek-Sondi, B.; Tang, L.; Elenkov, M. M. Azidolysis of Epoxides Catalysed by the Halohydrin Dehalogenase from *Arthrobacter* Sp. AD2 and a Mutant with Enhanced Enantioselectivity: An (*S*)-Selective HHDH. *Tetrahedron: Asymmetry* **2016**, *27*, 930–935. https://doi.org/10.1016/j.tetasy.2016.08.003.

(16) Piccinini, A.; Kavanagh, S. A.; Connon, S. J. Highly Enantioselective Ylide-Mediated Synthesis of Terminal Epoxides. *Chem. Commun.* **2012**, *48*, 7814–7816. https://doi.org/10.1039/C2CC32101G.
(17) Yamada, K.; Igarashi, Y.; Betsuyaku, T.; Kitamura, M.; Hirata, K.; Hioki, K.; Kunishima, M. An Isolable and Bench-Stable Epoxidizing Reagent Based on Triazine: Triazox. *Org. Lett.* **2018**, *20*, 2015–2019. https://doi.org/10.1021/acs.orglett.8b00560.
(18) Zhang, J. B., Vipul V. ;. Suzuki, Keisuke; Ohmori, Ken. Diastereoselective Access to Anti-β-Hydroxy Sulfoxides from Chiral Epoxides and Prochiral Sulfenate Anions: Mechanistic Insights, Scope, and Limitation. *Synlett* **2024**, *35*, 1458–1464. https://doi.org/10.1055/a-2196-5592.
(19) Li, A.; Liu, J.; Pham, S. Q.; Li, Z. Engineered P450pyr Monooxygenase for Asymmetric Epoxidation of Alkenes with Unique and High Enantioselectivity. *Chem. Commun.* **2013**, *49*, 11572–11574. https://doi.org/10.1039/C3CC46675B.
(20) Meng, Q.-Y.; Döben, N.; Studer, A. Cooperative NHC and Photoredox Catalysis for the Synthesis of β-Trifluoromethylated Alkyl Aryl Ketones. *Angewandte Chemie International Edition* **2020**, *59*, 19956–19960. https://doi.org/10.1002/anie.202008040.
(21) Fu, H.; Look, G. C.; Zhang, W.; Jacobsen, E. N.; Wong, C. H. Mechanistic Study of a Synthetically Useful Monooxygenase Model Using the Hypersensitive Probe Trans-2-Phenyl-1-Vinylcyclopropane. *J. Org. Chem.* **1991**, *56*, 6497–6500. https://doi.org/10.1021/jo00023a008.
(22) Dollet, R.; Villada, J. D.; Poisson, T.; Fasan, R.; Jubault, P. Chemoenzymatic Synthesis of Optically Active α-Cyclopropyl-Pyruvates and Cyclobutenoates *via* Enzyme-Catalyzed Carbene Transfer with Diazopyruvate. *Org. Chem. Front.* **2024**, *11*, 2008–2014. https://doi.org/10.1039/D3QO01987J.
(23) Dubois, G.; Murphy, A.; Stack, T. D. P. Simple Iron Catalyst for Terminal Alkene Epoxidation. *Org. Lett.* **2003**, *5*, 2469–2472. https://doi.org/10.1021/ol0347085.
(24) Taguchi, M.; Nagasawa, Y.; Yamaguchi, E.; Tada, N.; Miura, T.; Itoh, A. One-Pot Epoxidation of Alkenes Using Aerobic Photoperoxidation of Toluenes. *Tetrahedron Letters* **2016**, *57*, 230–232. https://doi.org/10.1016/j.tetlet.2015.12.027.
(25) Mello, R.; Alcalde-Aragonés, A.; Olmos, A.; González-Núñez, M. E.; Asensio, G. Epoxidation of Olefins with a Silica-Supported Peracid in Supercritical Carbon Dioxide under Flow Conditions. *J. Org. Chem.* **2012**, *77*, 4706–4710. https://doi.org/10.1021/jo300532f.
(26) Fujisawa, T.; Takeuchi, M.; Sato, T. A Short-Step Synthesis of (±)-Pyrenophorin Utilizing 3-Alkenoate as a Masked Synthon of 4-Oxo-2-Alkenoate. *Chem. Lett.* **1982**, *11*, 1795–1798. https://doi.org/10.1246/cl.1982.1795.
(27) Cussó, O.; Garcia-Bosch, I.; Font, D.; Ribas, X.; Lloret-Fillol, J.; Costas, M. Highly Stereoselective Epoxidation with H_2O_2 Catalyzed by Electron-Rich Aminopyridine Manganese Catalysts. *Org. Lett.* **2013**, *15*, 6158–6161. https://doi.org/10.1021/ol403018x.
(28) Clark, P. G.; Guidry, E. N.; Chan, W. Y.; Steinmetz, W. E.; Grubbs, R. H. Synthesis of a Molecular Charm Bracelet via Click Cyclization and Olefin Metathesis Clipping. *J. Am. Chem. Soc.* **2010**, *132*, 3405–3412. https://doi.org/10.1021/ja9090337.
(29) Uyanik, M.; Ishihara, K.; Yamamoto, H. Catalytic Diastereoselective Polycyclization of Homo(Polyprenyl)Arene Analogues Bearing Terminal Siloxyvinyl Groups. *Org. Lett.* **2006**, *8*, 5649–5652. https://doi.org/10.1021/ol062378t.

(30) Crivello, J. V.; Narayan, R. Epoxidized Triglycerides as Renewable Monomers in Photoinitiated Cationic Polymerization. *Chem. Mater.* **1992**, *4*, 692–699. https://doi.org/10.1021/cm00021a036.
(31) Watanabe, Y.; Laschat, S.; Budde, M.; Affolter, O.; Shimada, Y.; Urlacher, V. B. Oxidation of Acyclic Monoterpenes by P450 BM-3 Monooxygenase: Influence of the Substrate *E*/*Z*-Isomerism on Enzyme Chemo- and Regioselectivity. *Tetrahedron* **2007**, *63*, 9413–9422. https://doi.org/10.1016/j.tet.2007.06.104.
(32) Kawashima, H.; Okuda, Y.; Kijima, M.; Fujitani, T.; Choi, J.-C. Epoxidation of Microalgal Biomass-Derived Squalene with Hydrogen Peroxide Using Solid Heterogeneous Tungsten-Based Catalyst. *Tetrahedron* **2020**, *76*, 131109. https://doi.org/10.1016/j.tet.2020.131109.
(33) Cunningham, W. B.; Tibbetts, J. D.; Hutchby, M.; Maltby, K. A.; Davidson, M. G.; Hintermair, U.; Plucinski, P.; Bull, S. D. Sustainable Catalytic Protocols for the Solvent Free Epoxidation and Anti-Dihydroxylation of the Alkene Bonds of Biorenewable Terpene Feedstocks Using H_2O_2 as Oxidant. *Green Chem.* **2020**, *22*, 513–524. https://doi.org/10.1039/C9GC03208H.

5. NMR Spectra

2-phenyloxirane, **4a**:

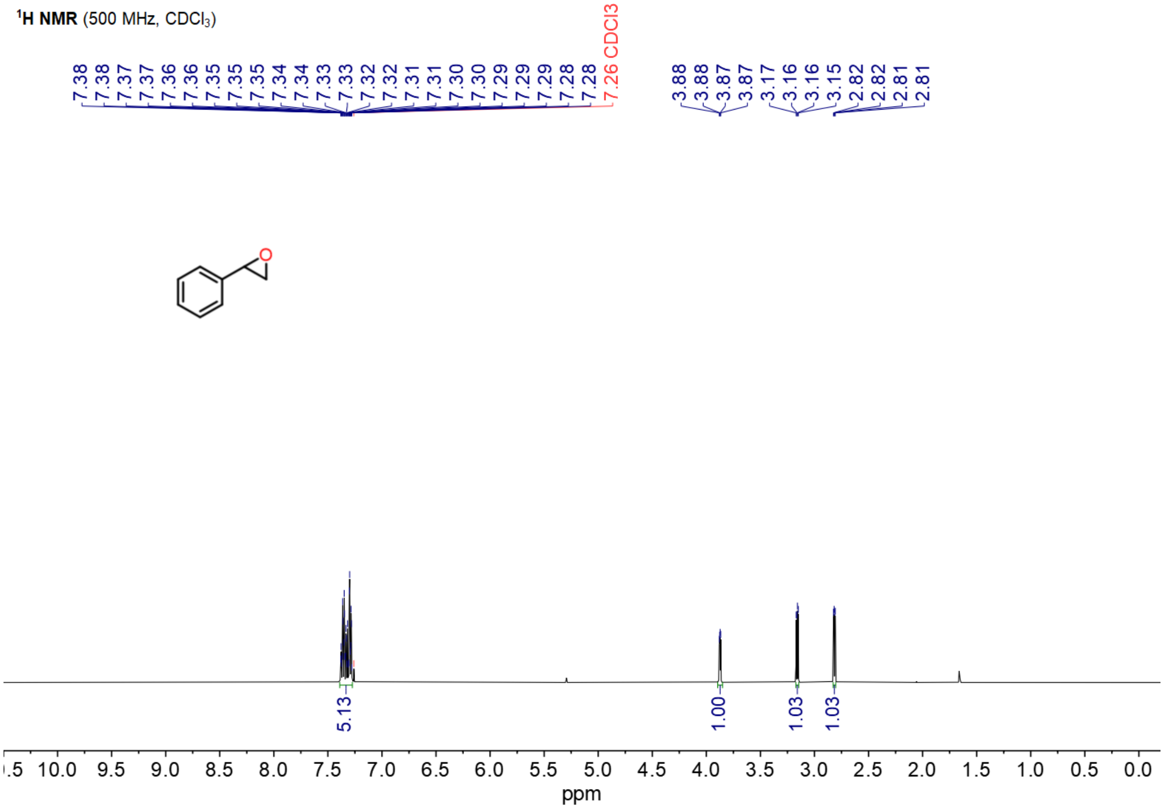

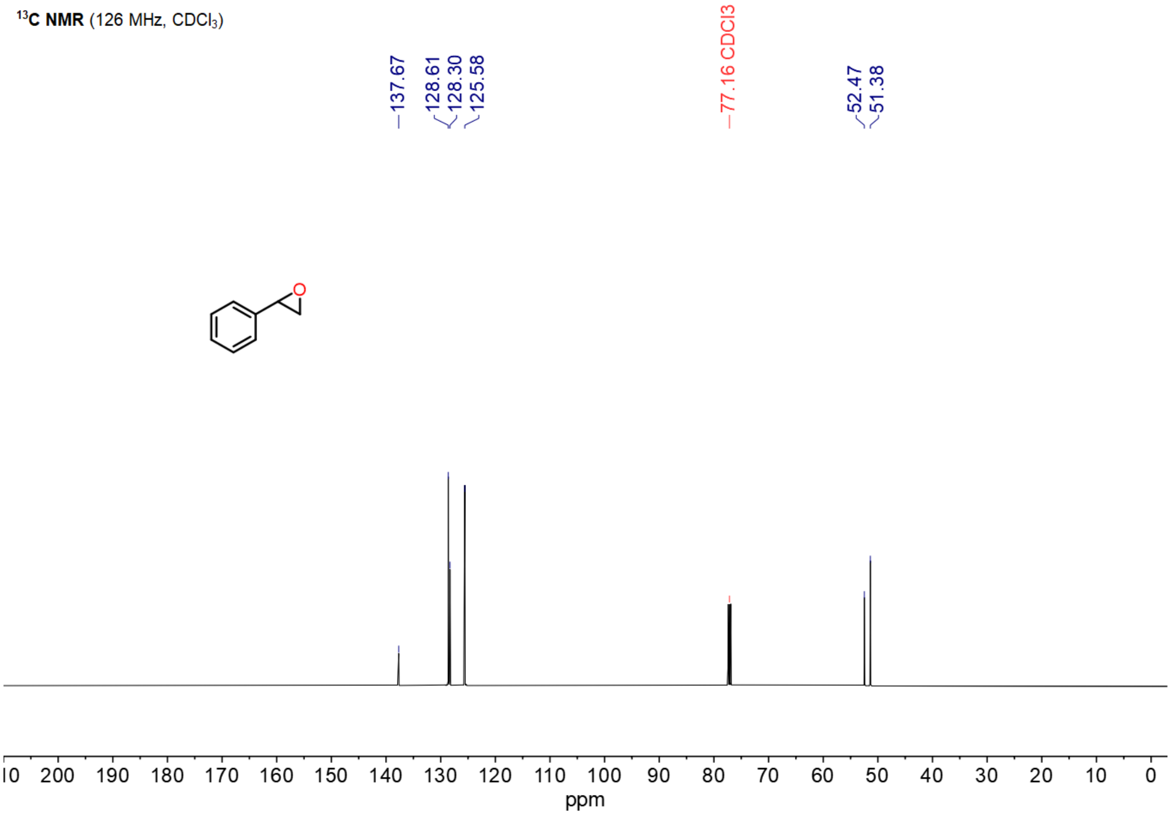

2-(4-(tert-butyl)phenyl)oxirane, **2m**:

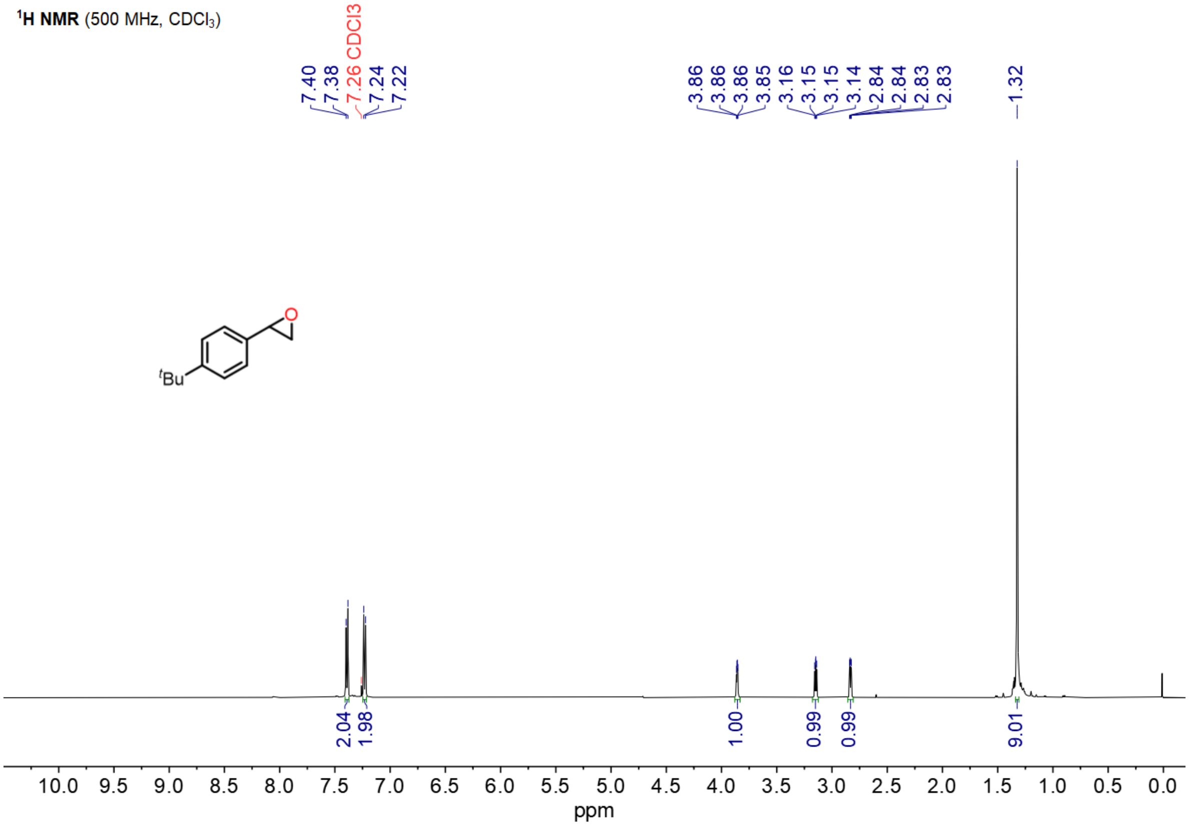

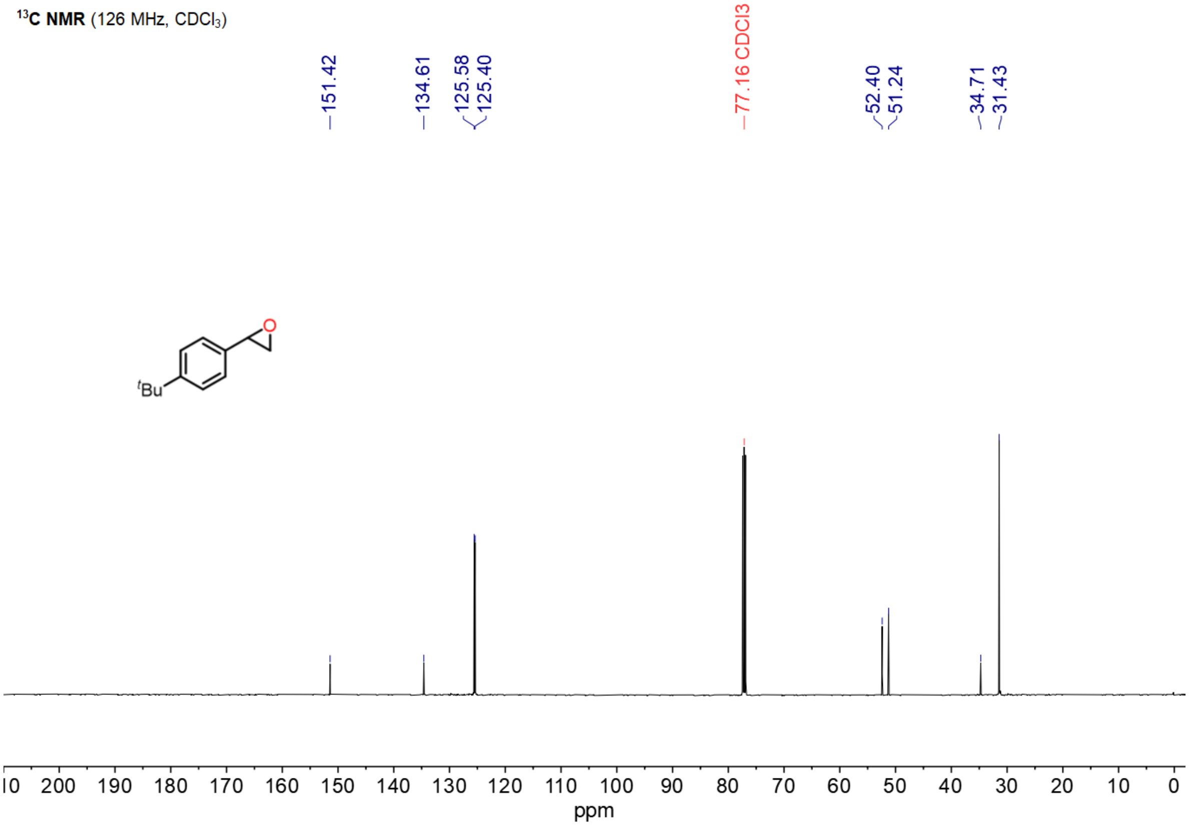

2-(4-methoxyphenyl)oxirane, **4b**:

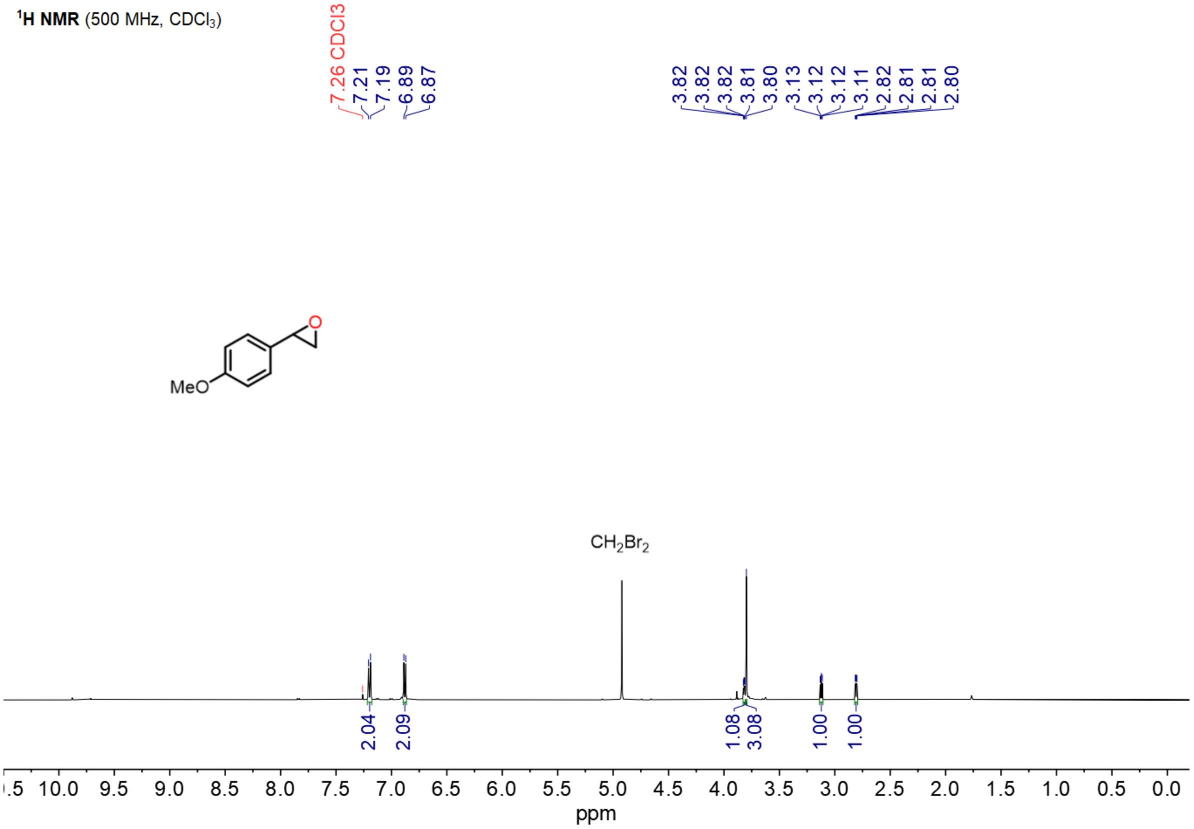

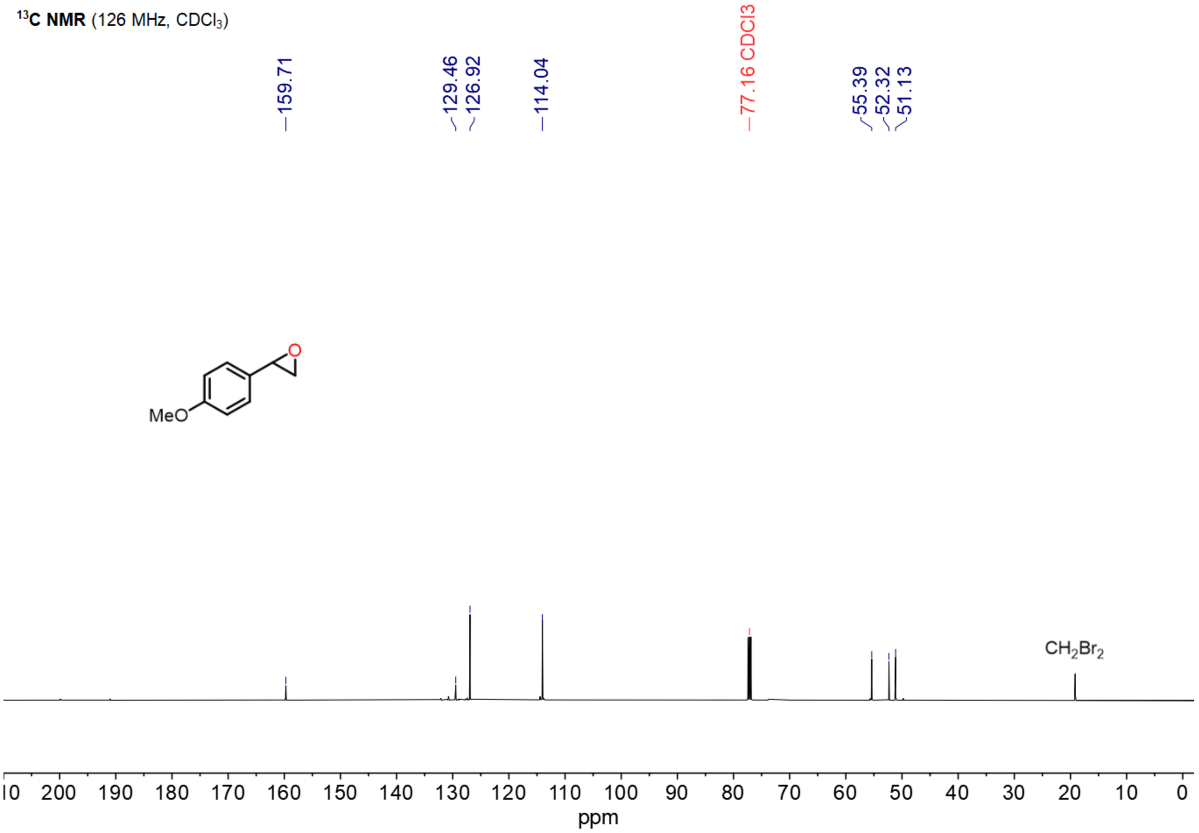

2-(4-bromophenyl)oxirane, **4c**:

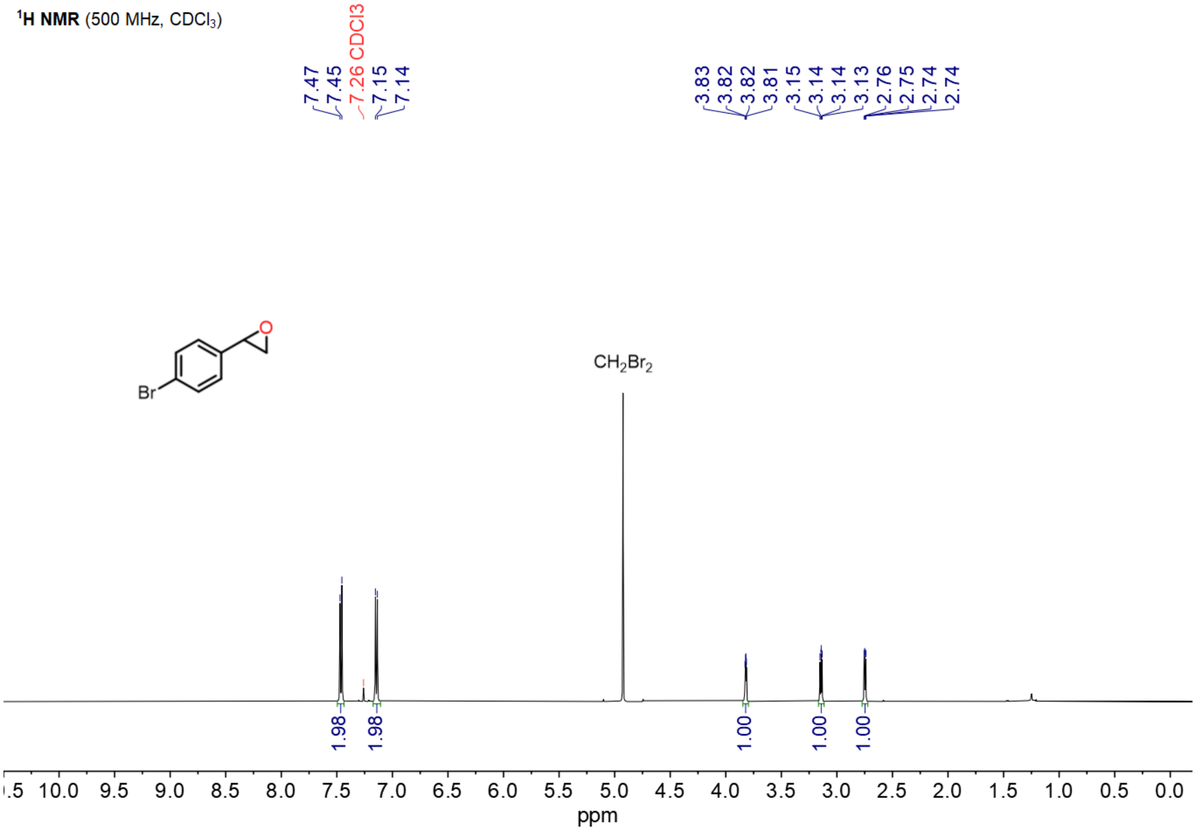

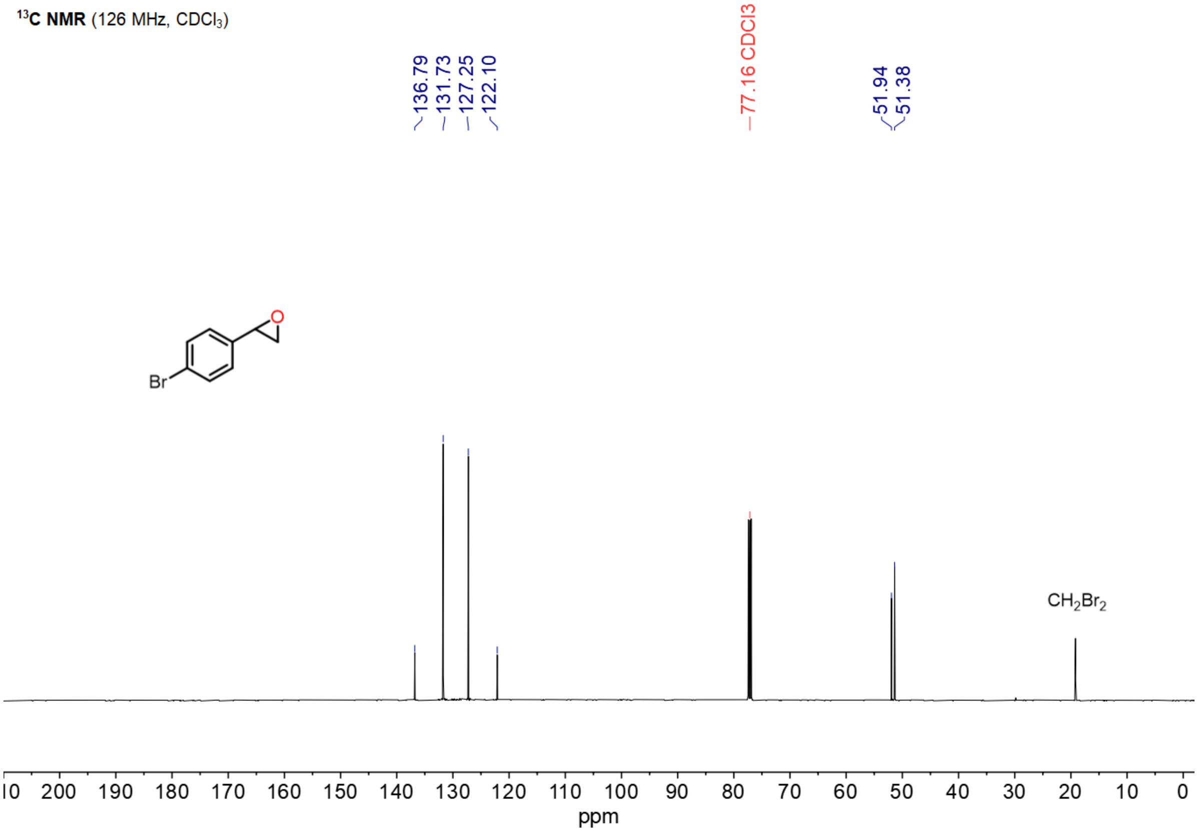

2-(4-fluorophenyl)oxirane, **4d**:

^{1}**H NMR** (500 MHz, $CDCl_3$)

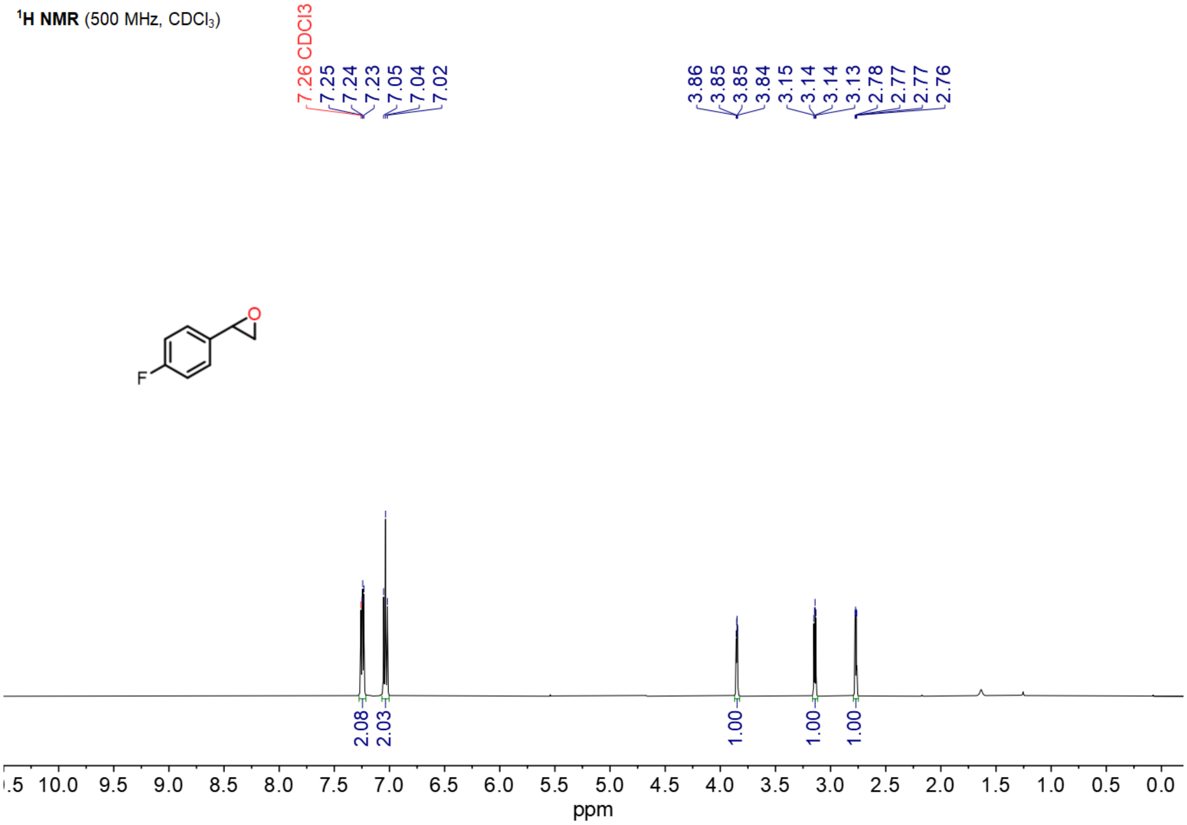

^{13}**C NMR** (126 MHz, $CDCl_3$)

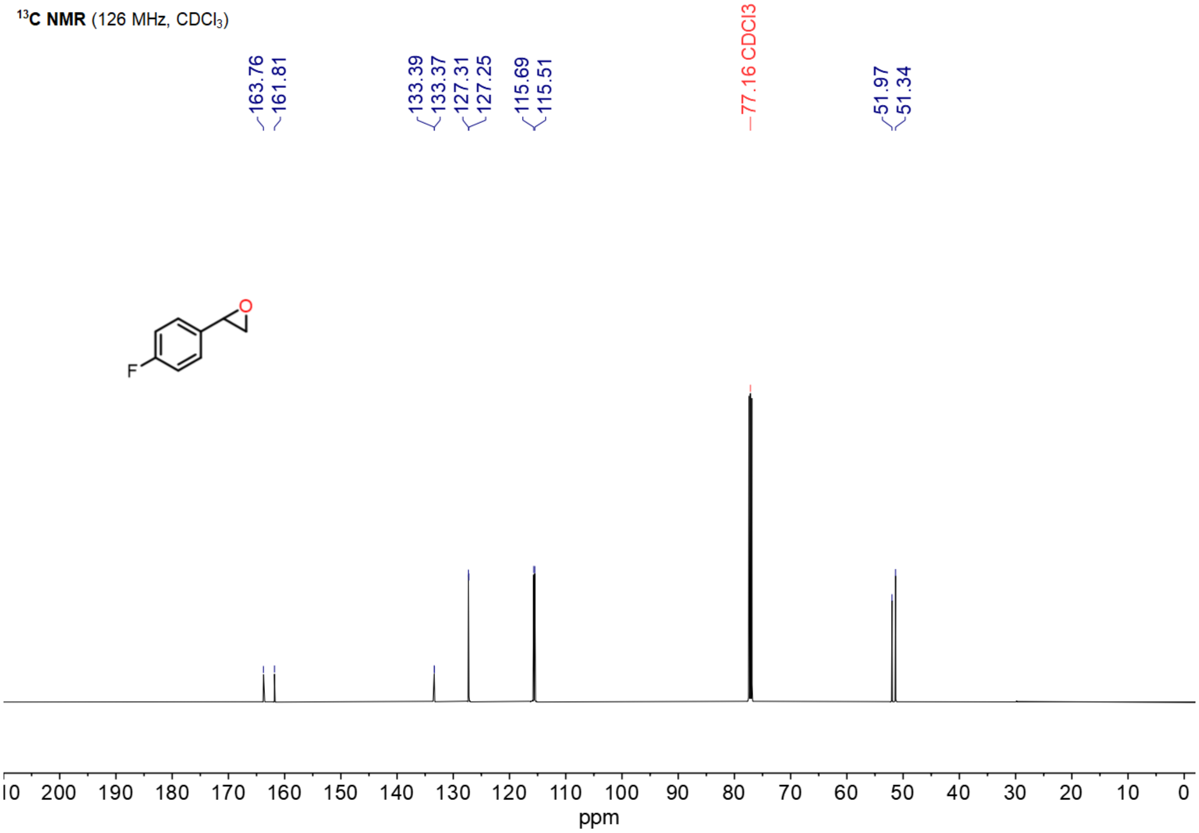

methyl 4-(oxiran-2-yl)benzoate, **4e**:

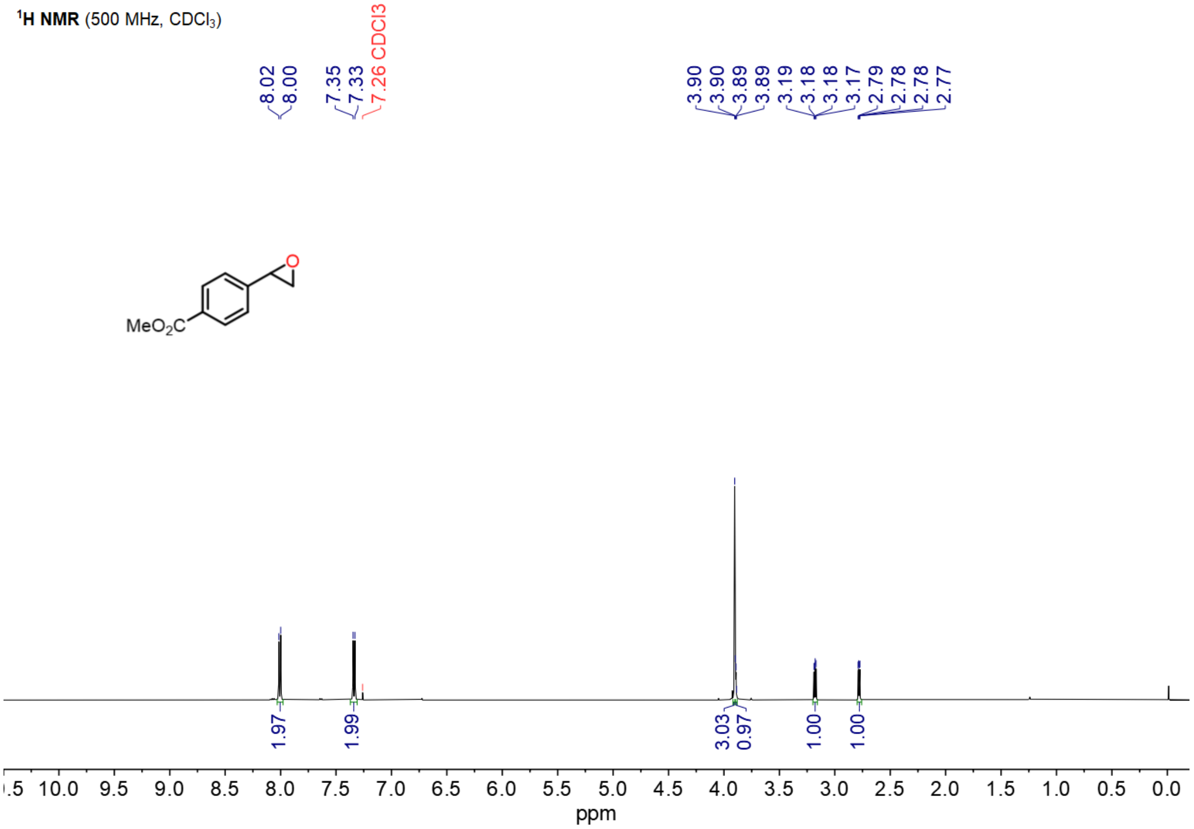

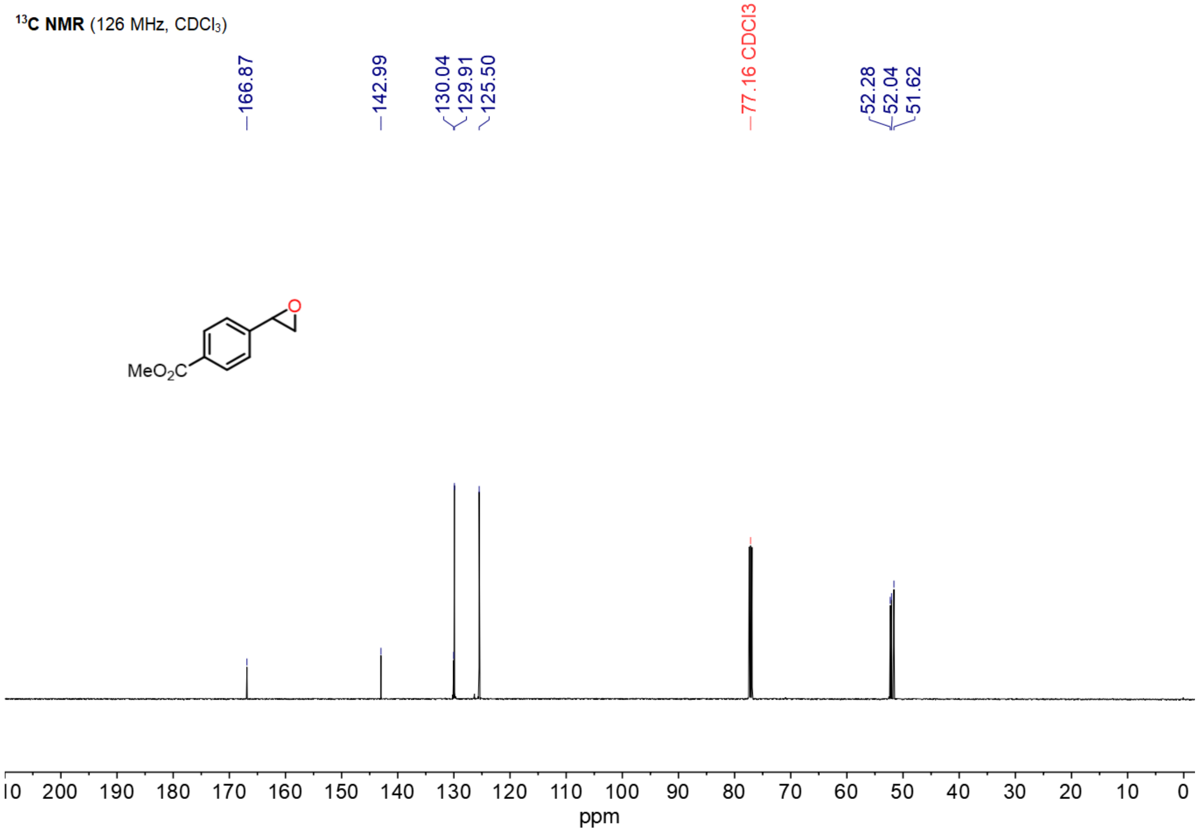

4-(oxiran-2-yl)benzonitrile, **4f**:

^{1}H NMR (500 MHz, $CDCl_3$)

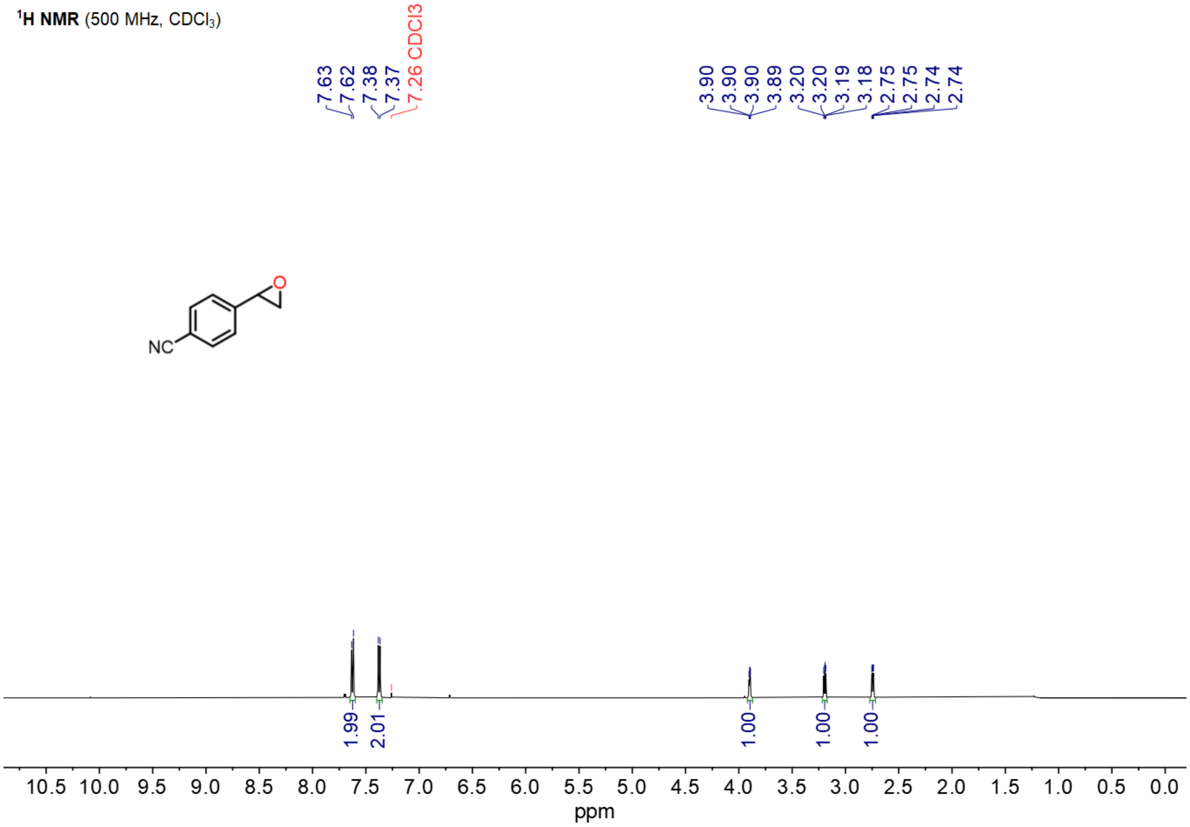

^{13}C NMR (126 MHz, $CDCl_3$)

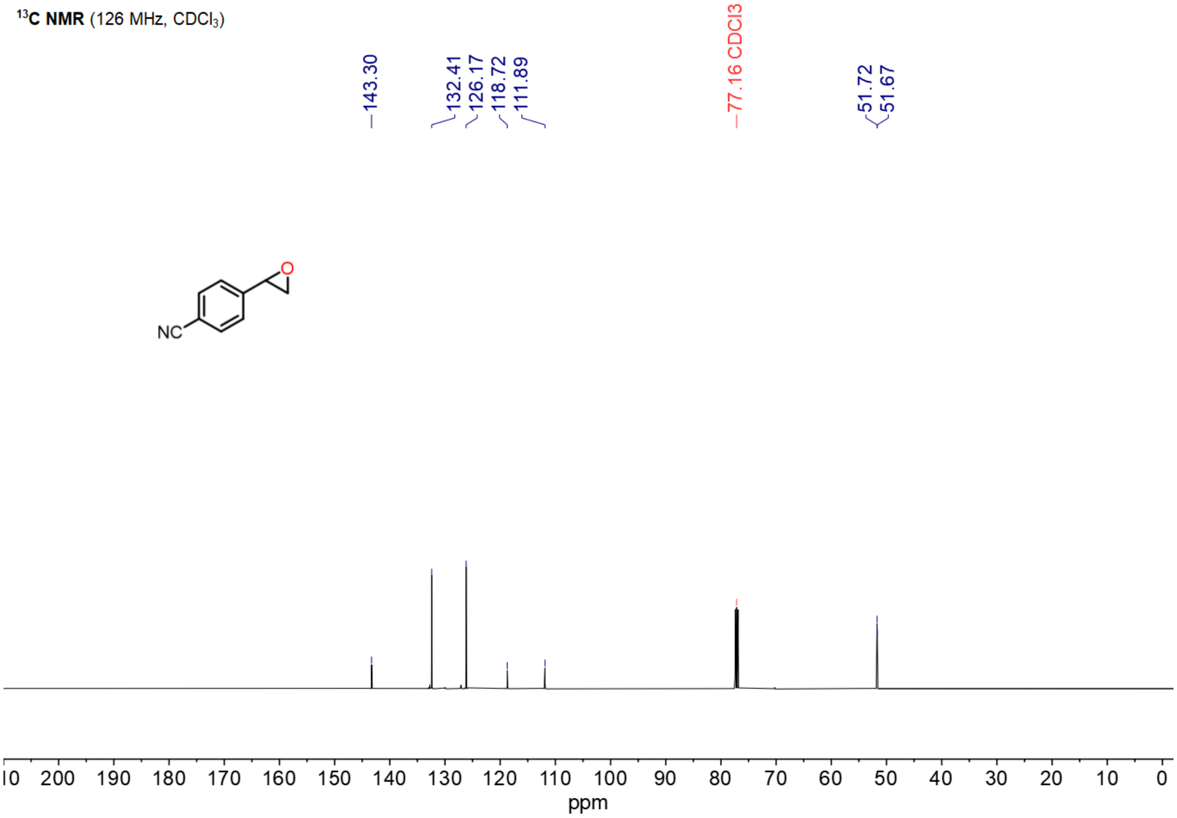

2-(4-nitrophenyl)oxirane, **4g**:

^{1}**H NMR** (500 MHz, $CDCl_3$)

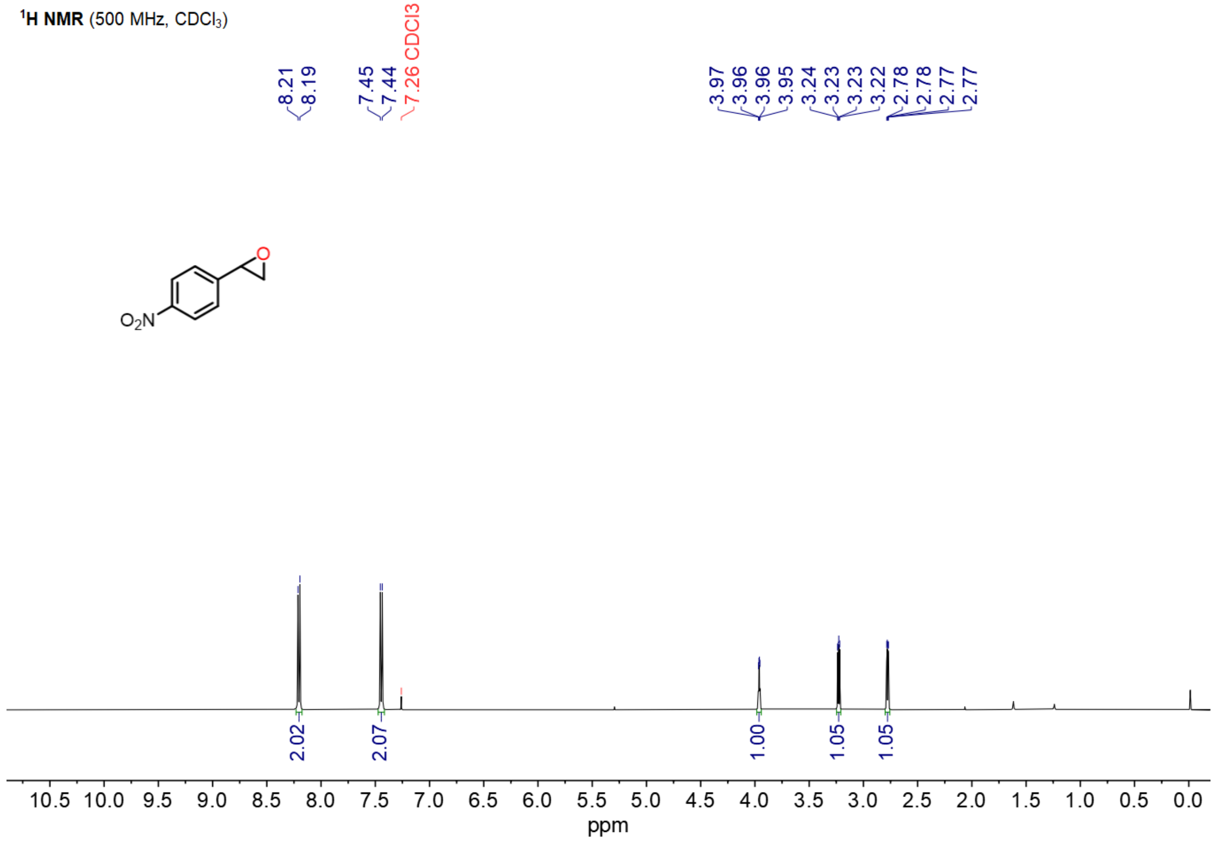

^{13}**C NMR** (126 MHz, $CDCl_3$)

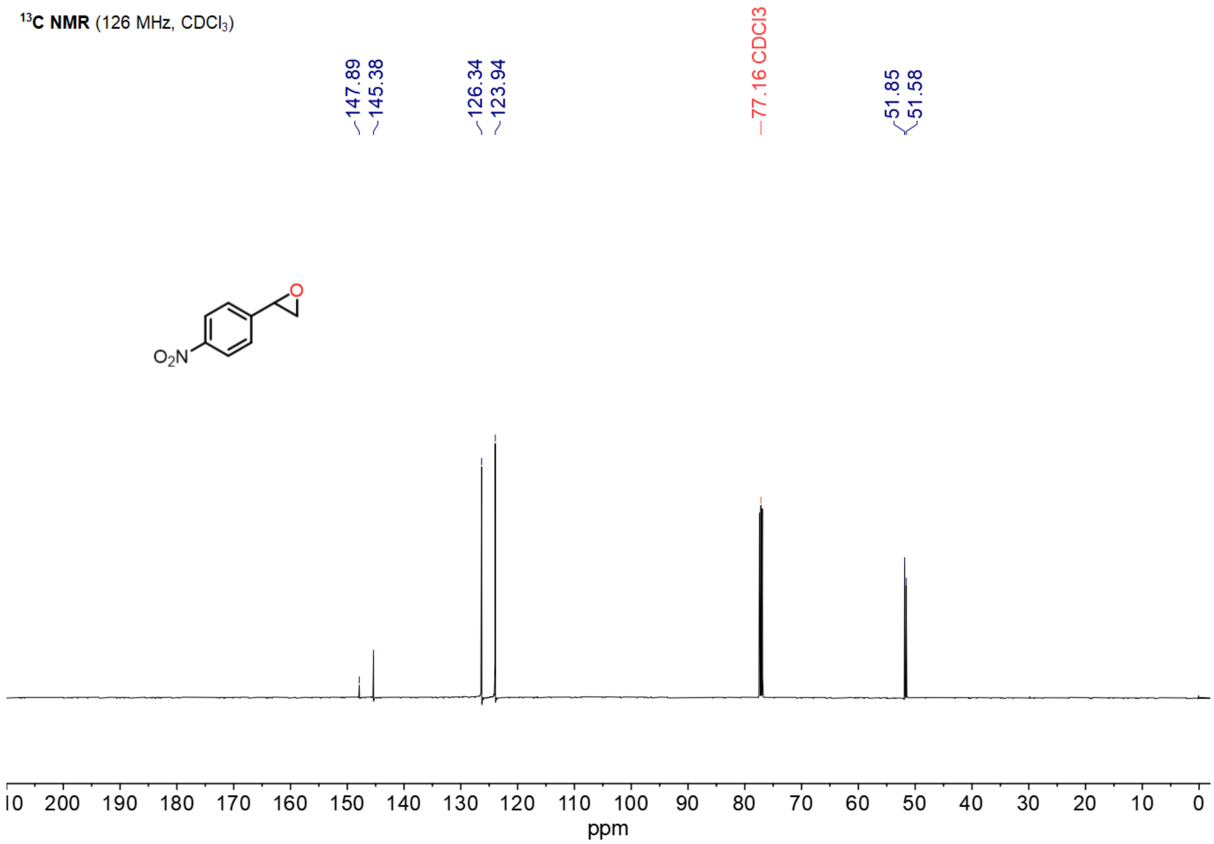

2-(4-(chloromethyl)phenyl)oxirane, **4h**:

^{1}H NMR (500 MHz, $CDCl_3$)

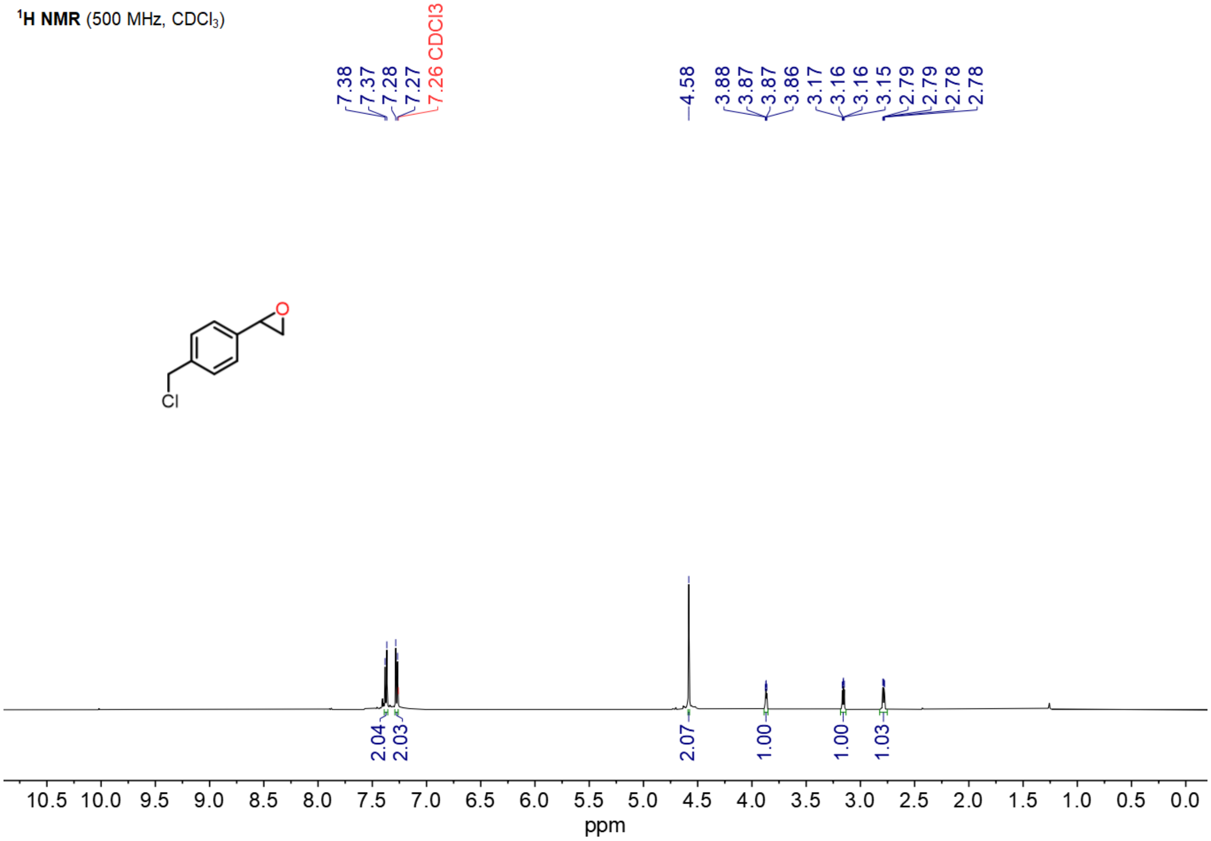

^{13}C NMR (126 MHz, $CDCl_3$)

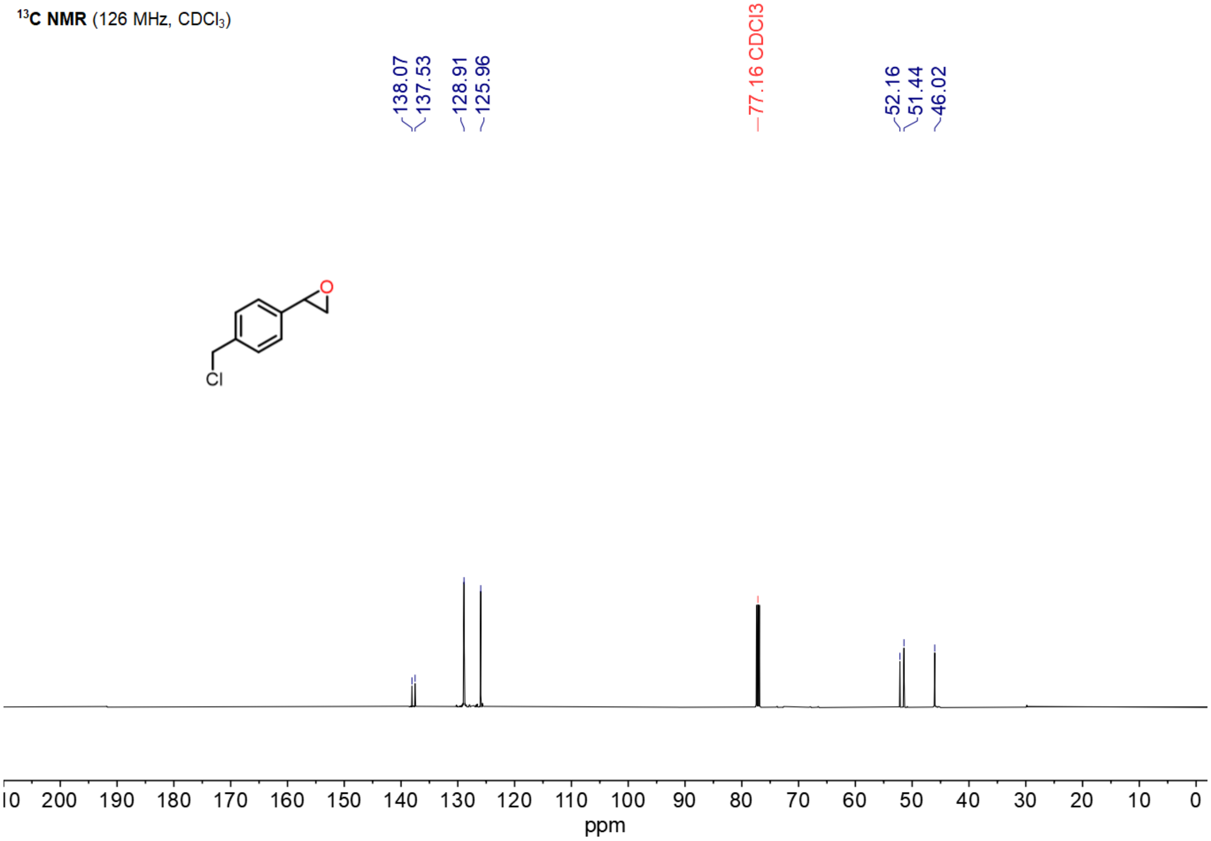

2,2-diphenyloxirane, **4i**:

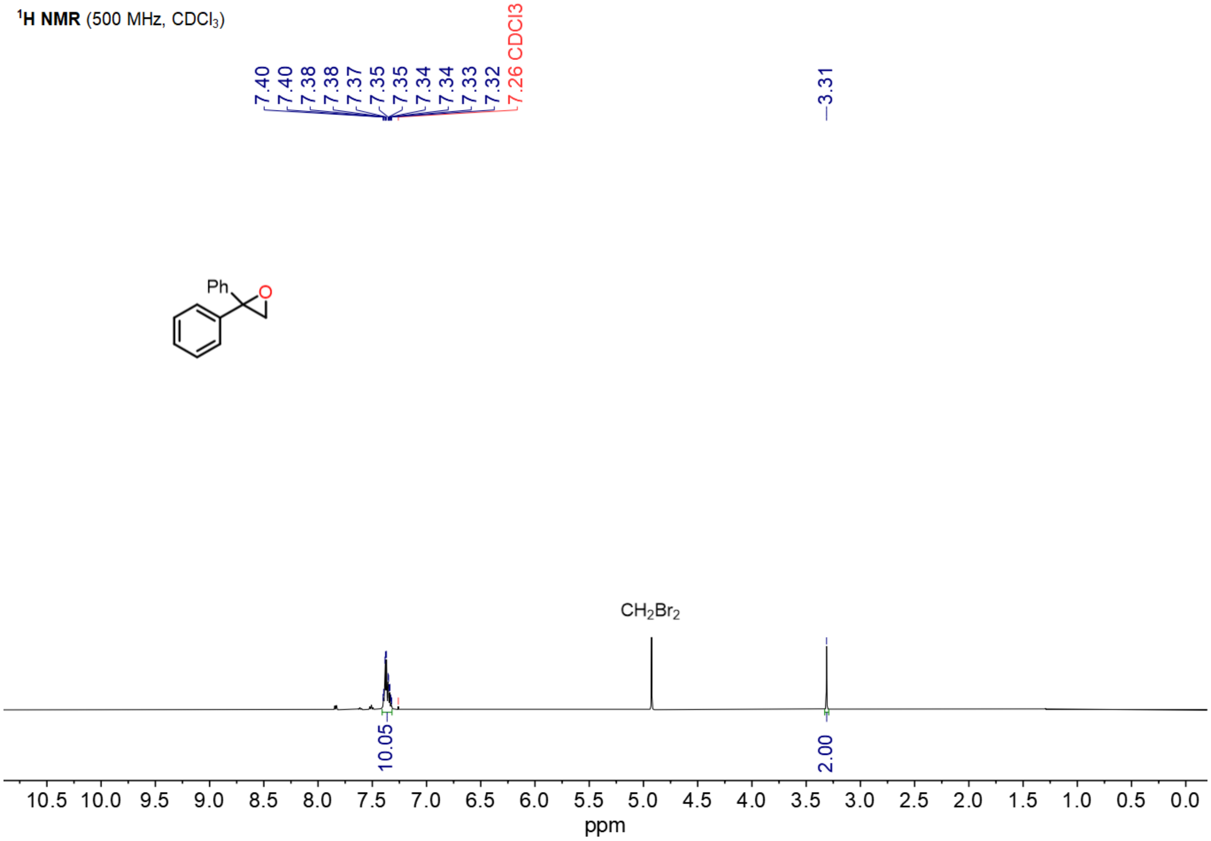

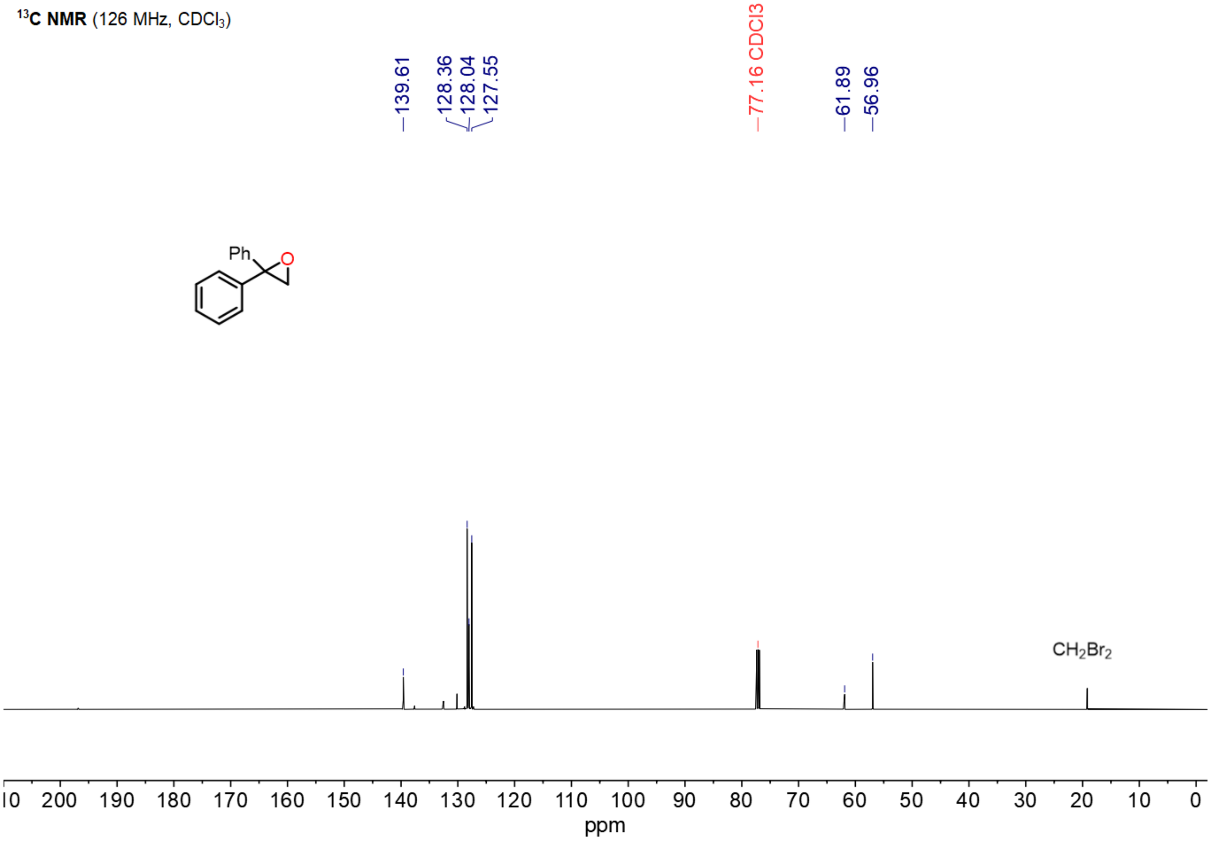

2-(oxiran-2-yl)pyridine, **2j**:

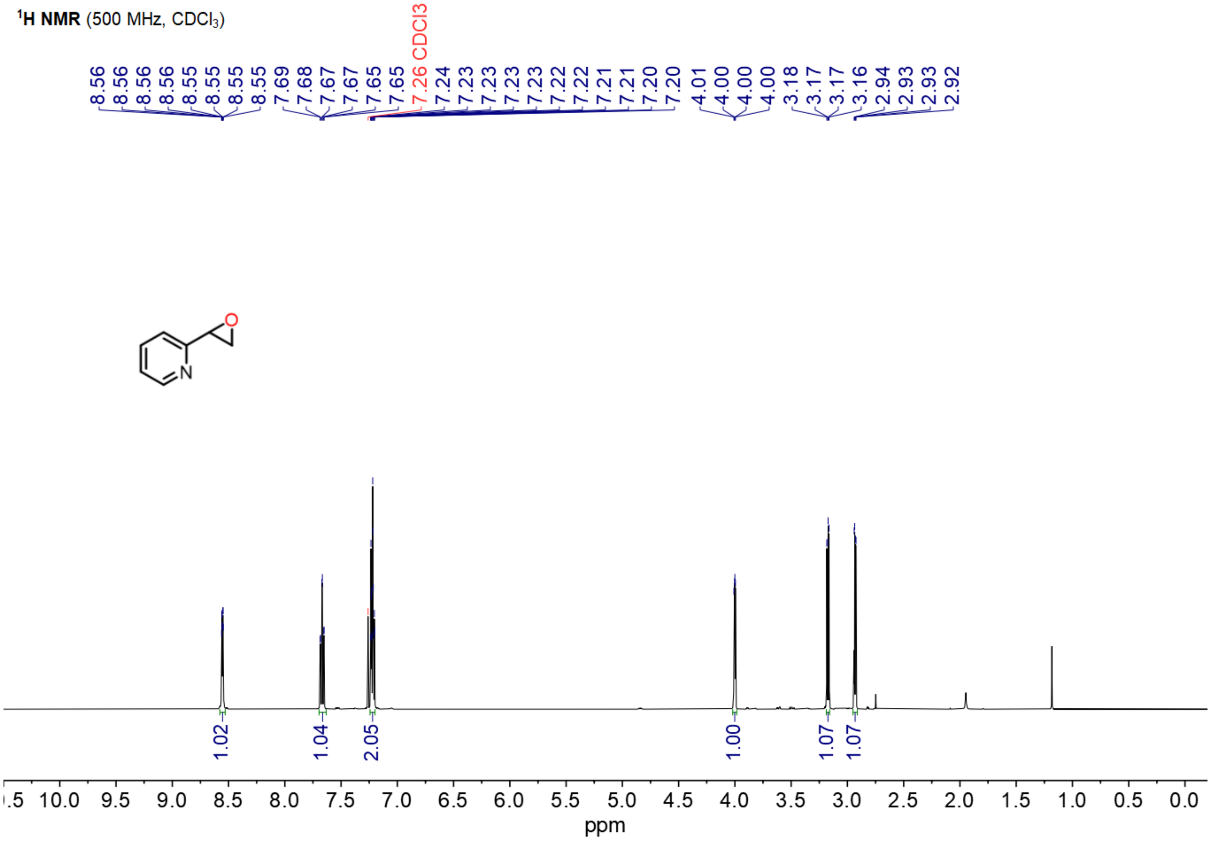

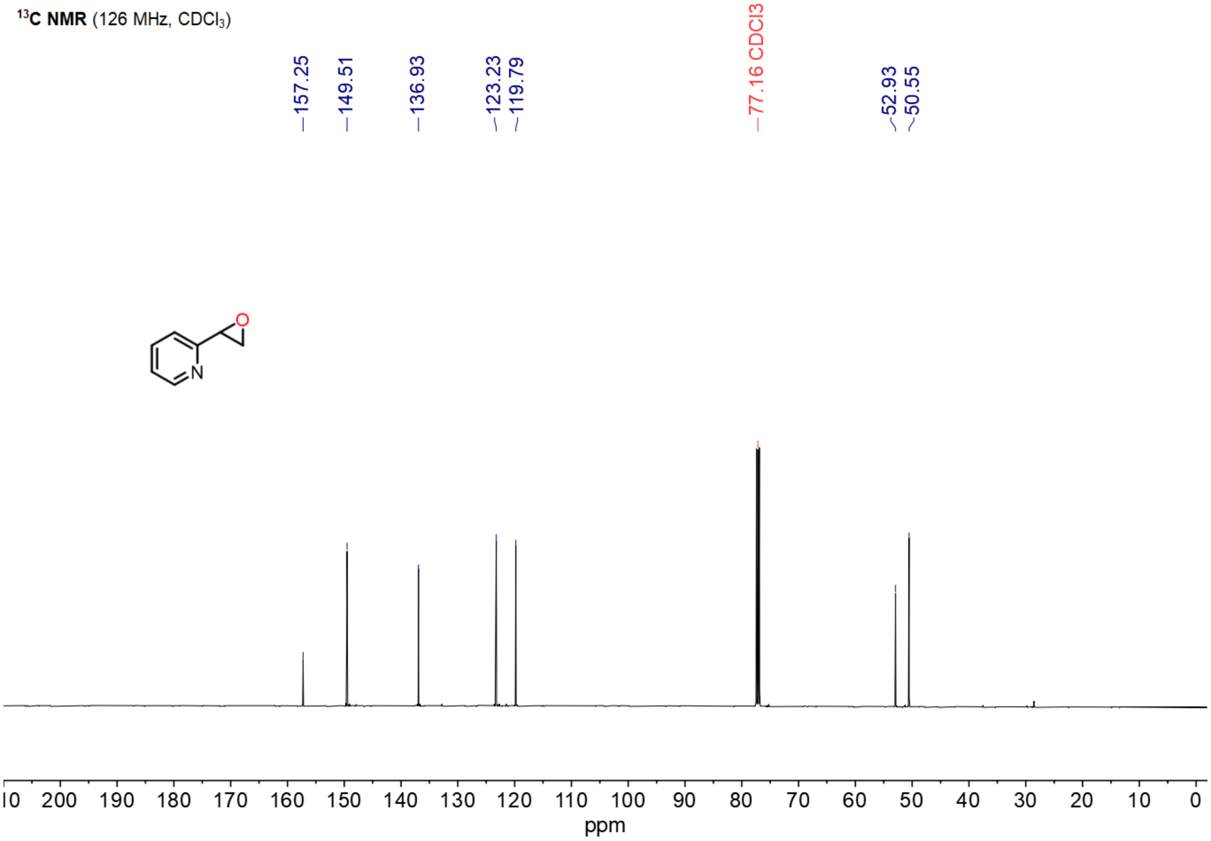

3-(oxiran-2-yl)pyridine, **4j**:

[1] **H NMR** (500 MHz, $CDCl_3$)

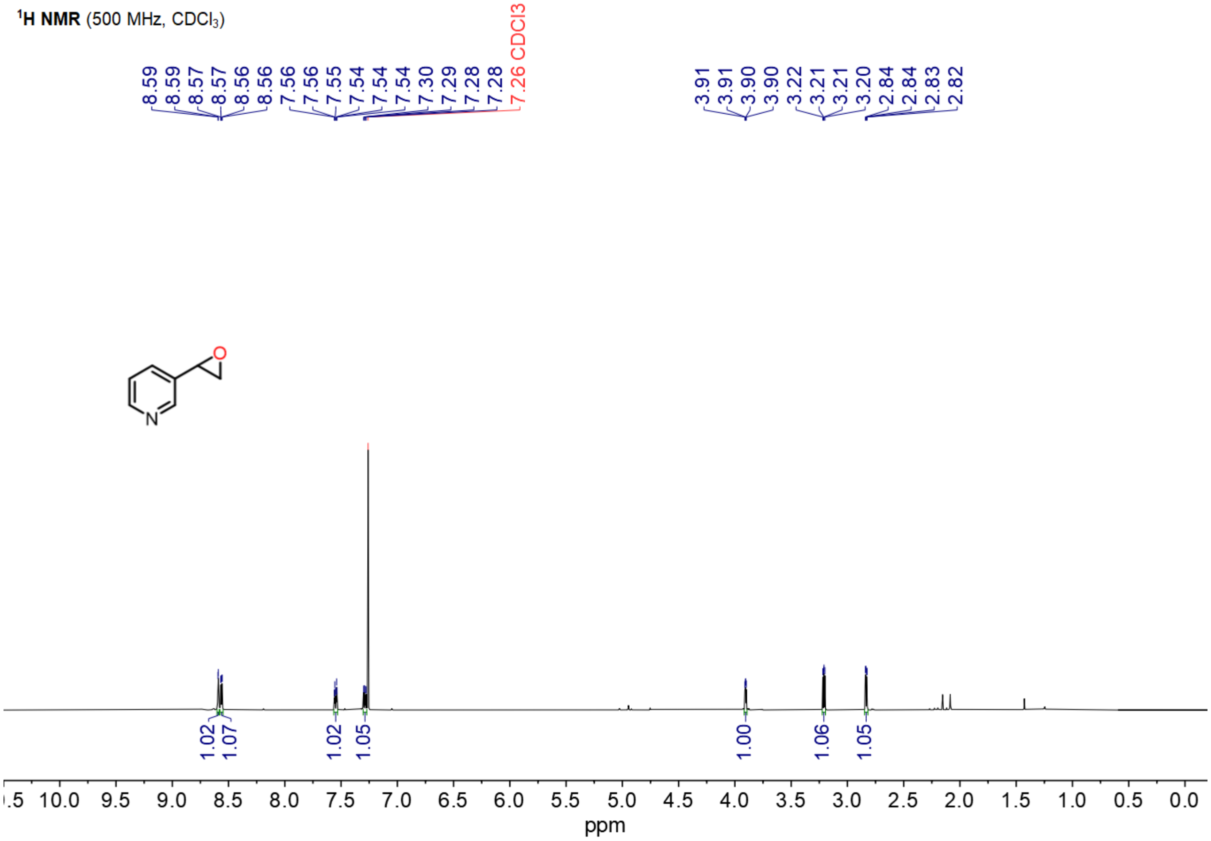

[13] **C NMR** (126 MHz, $CDCl_3$)

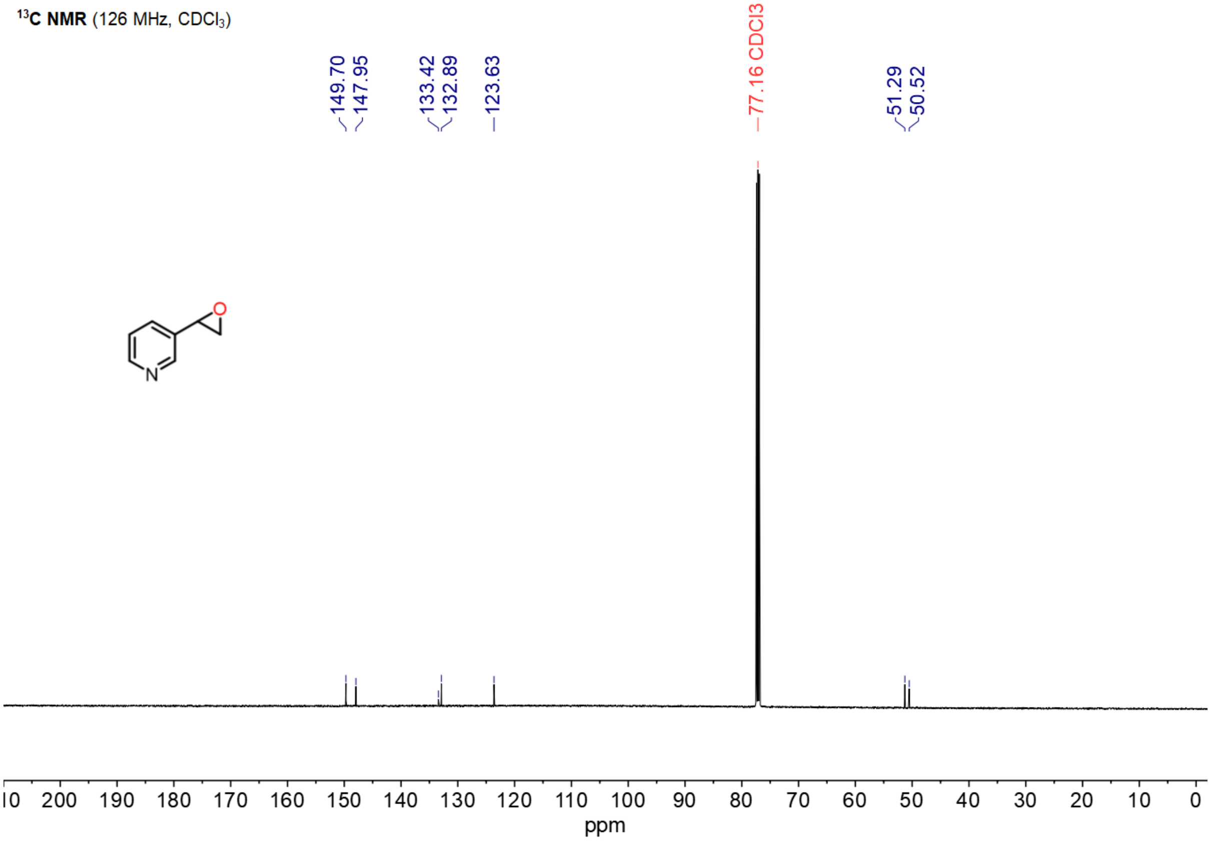

2-(oxiran-2-yl)pyrazine, **2k**:

[1]**H NMR** (500 MHz, $CDCl_3$)

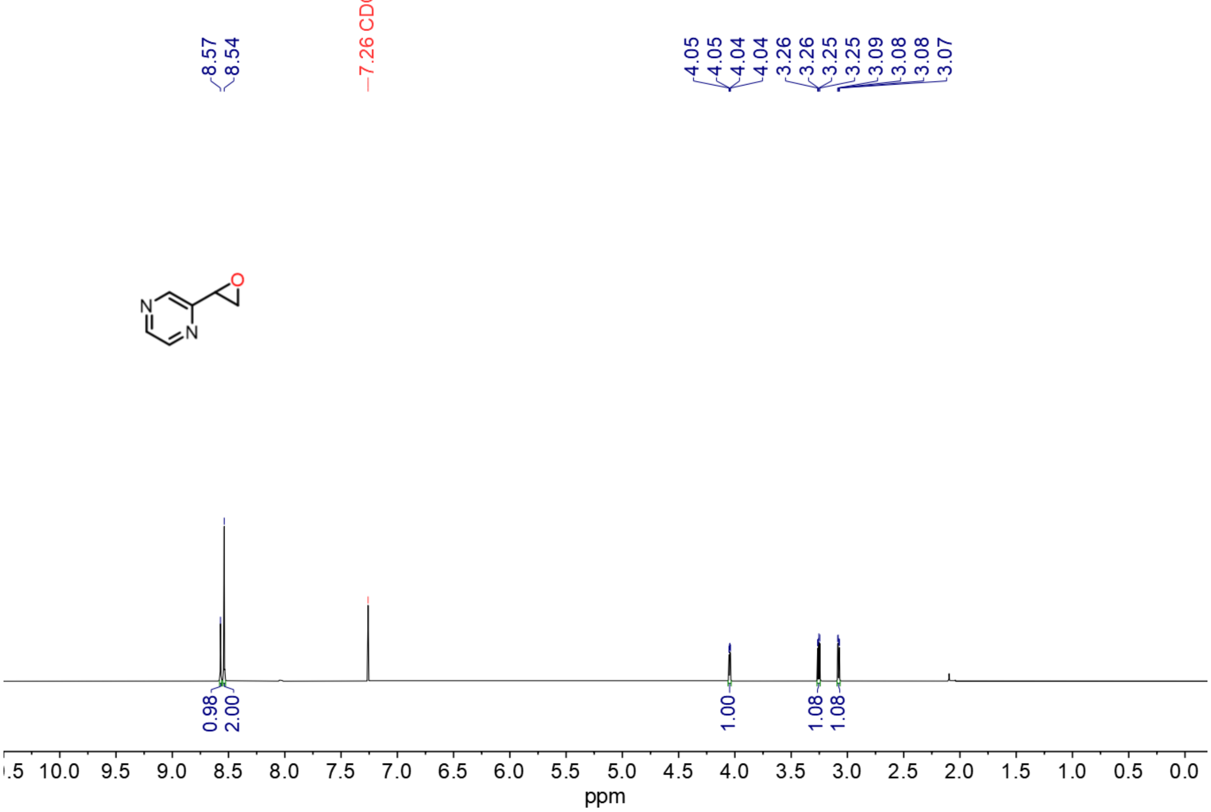

[13]**C NMR** (126 MHz, $CDCl_3$)

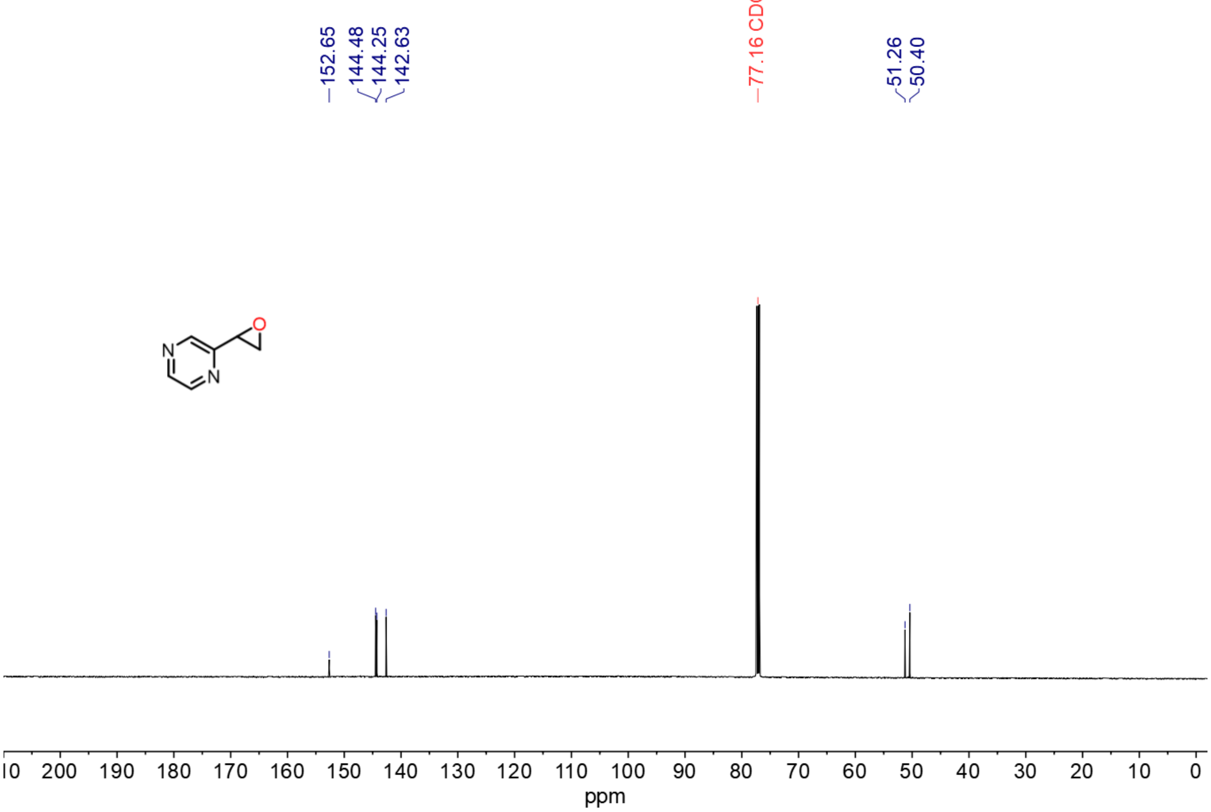

rel-(2*R*,3*R*)-2-methyl-3-phenyloxirane, **4k**:

^{1}H NMR (500 MHz, $CDCl_3$)

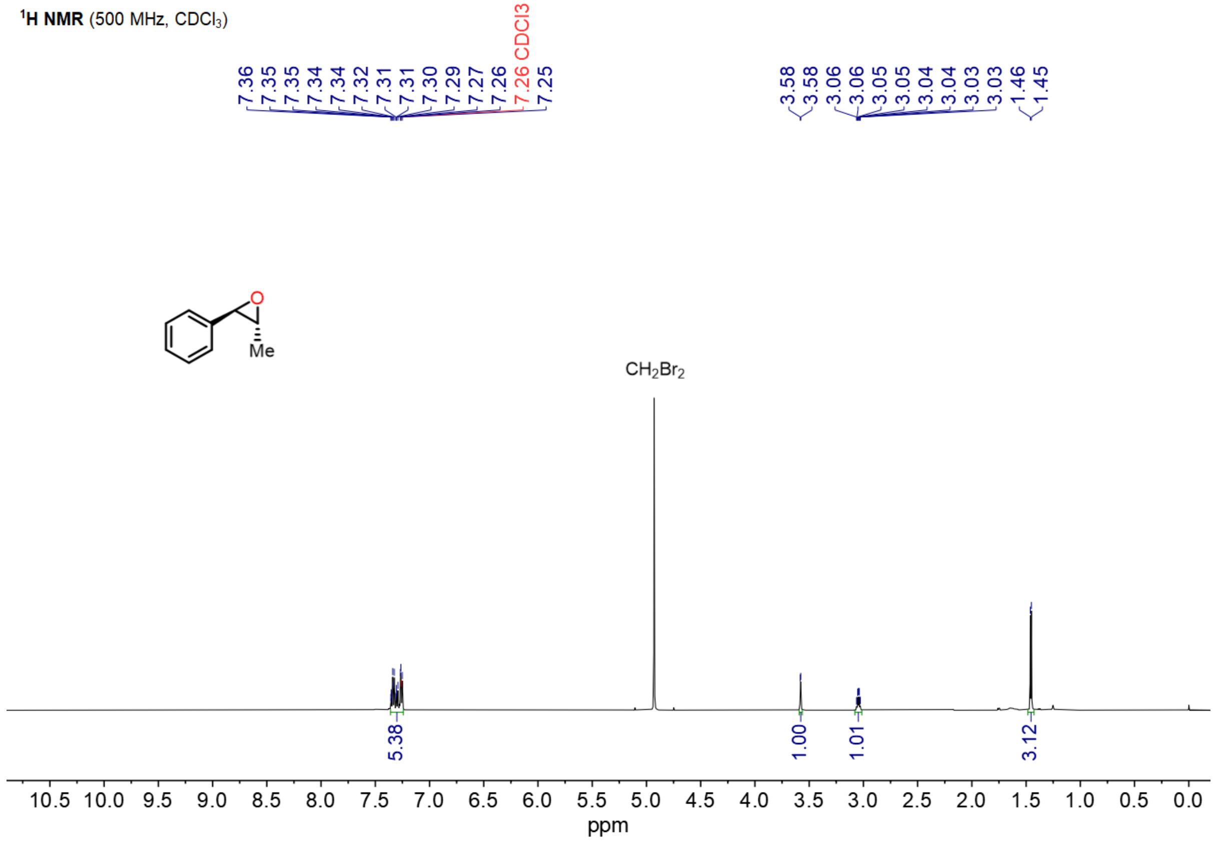

^{13}C NMR (126 MHz, $CDCl_3$)

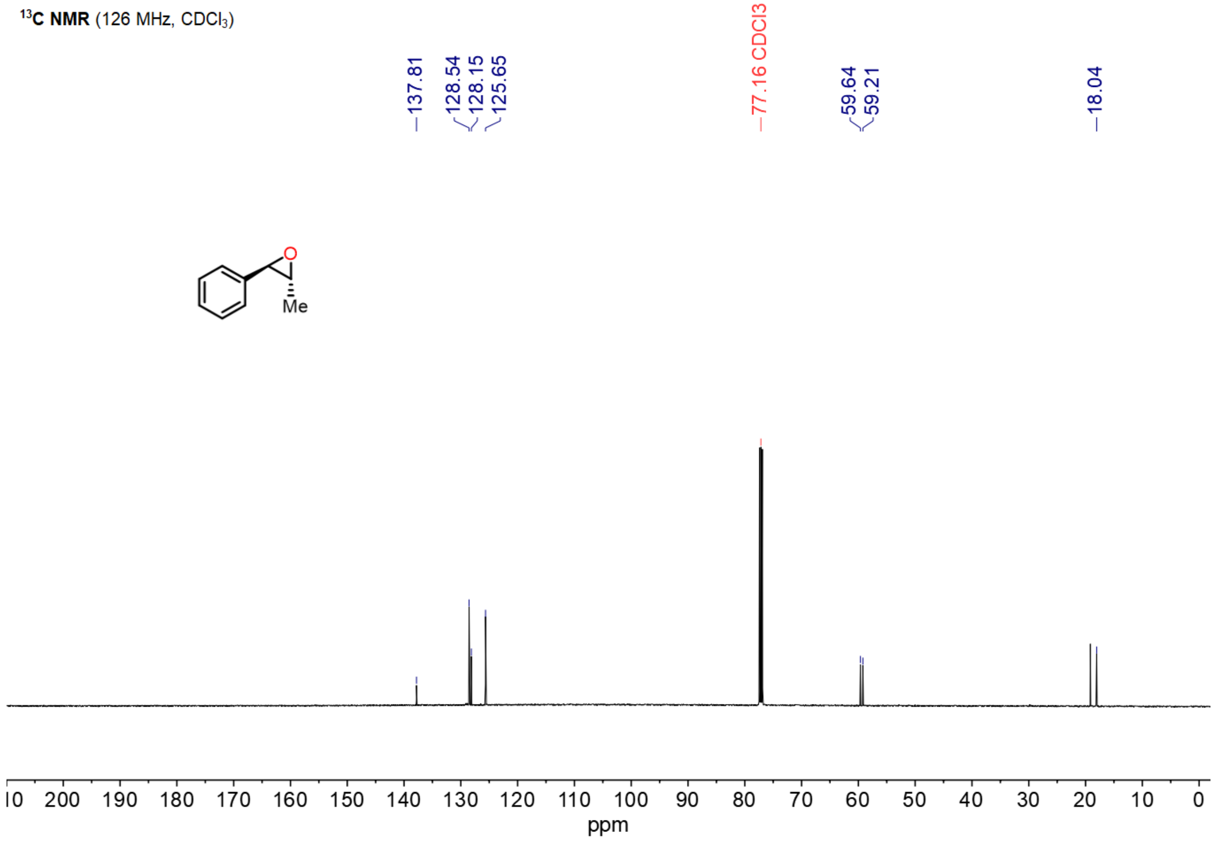

rel-(2*R*,3*R*)-2,3-diphenyloxirane, **4l**:

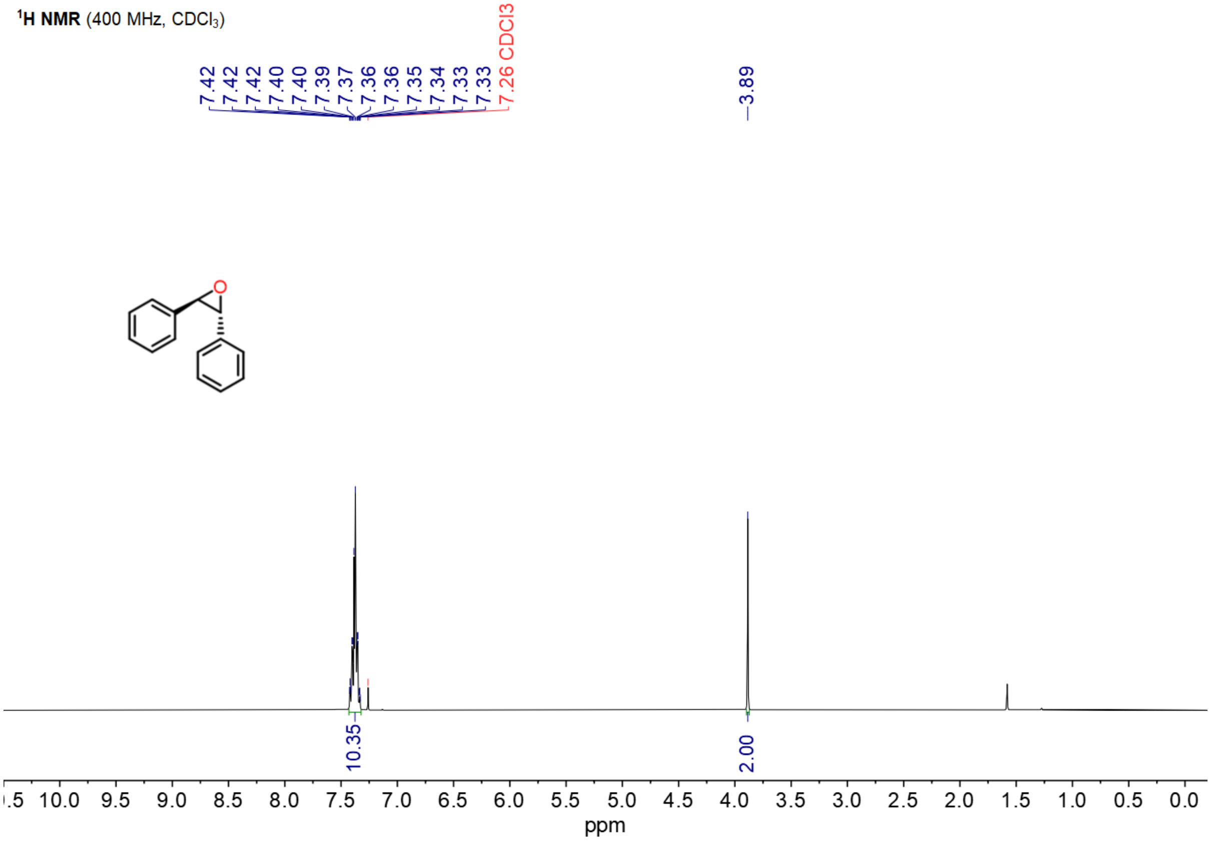

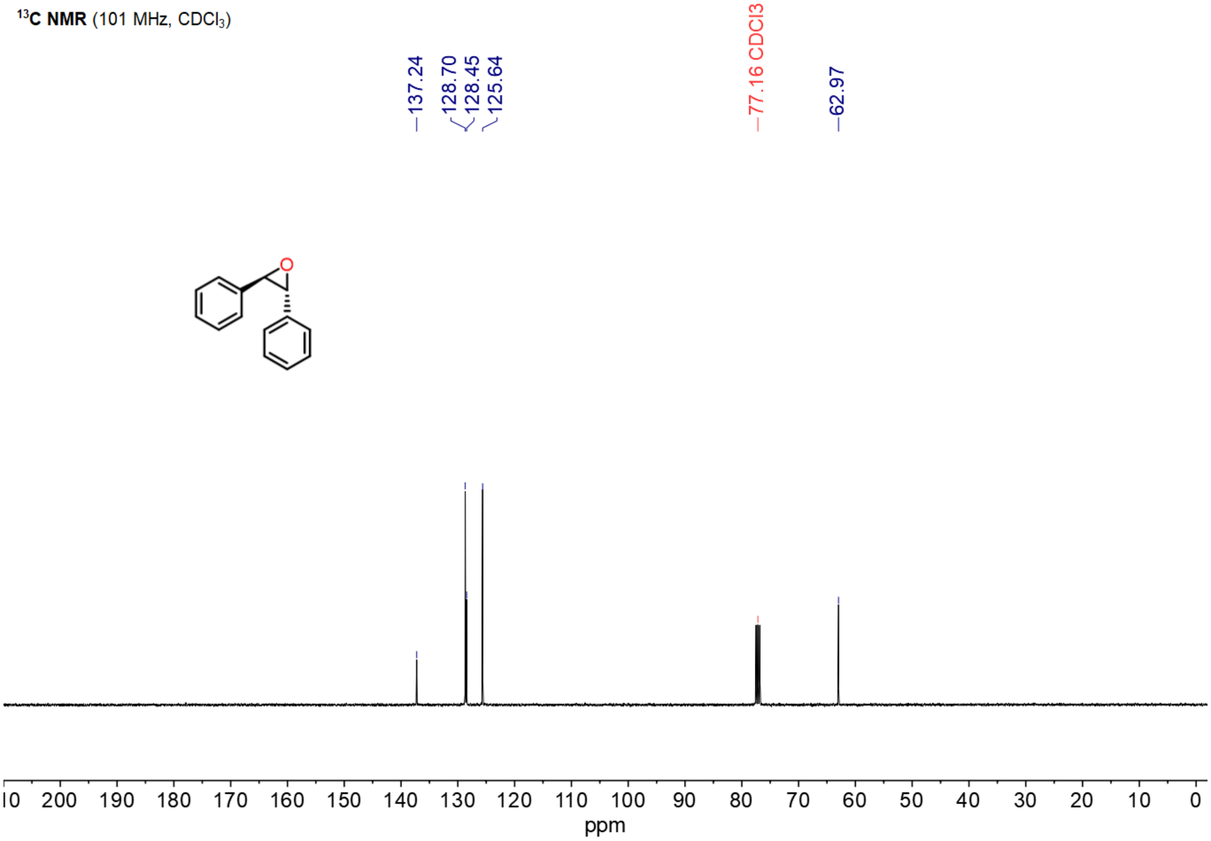

rel-(2*R*,3*S*)-2,3-diphenyloxirane, **2c**:

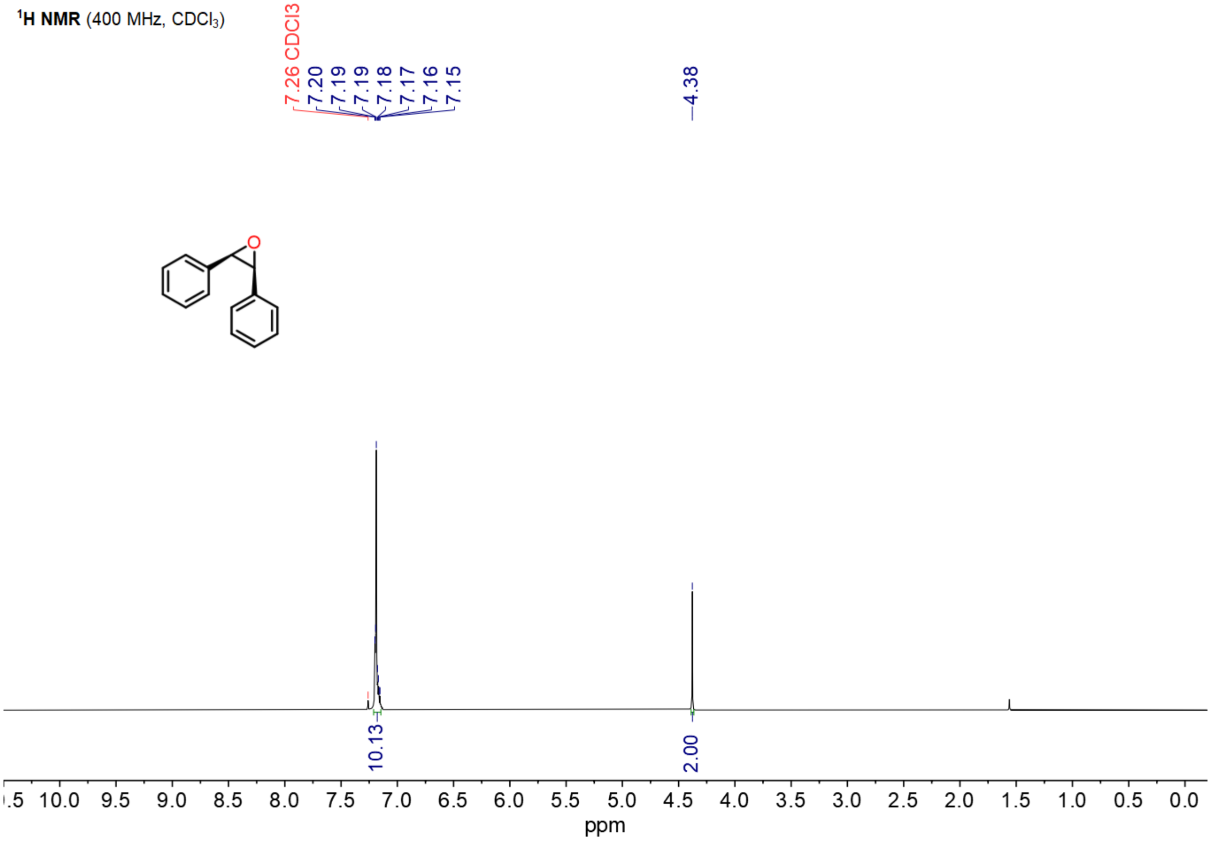

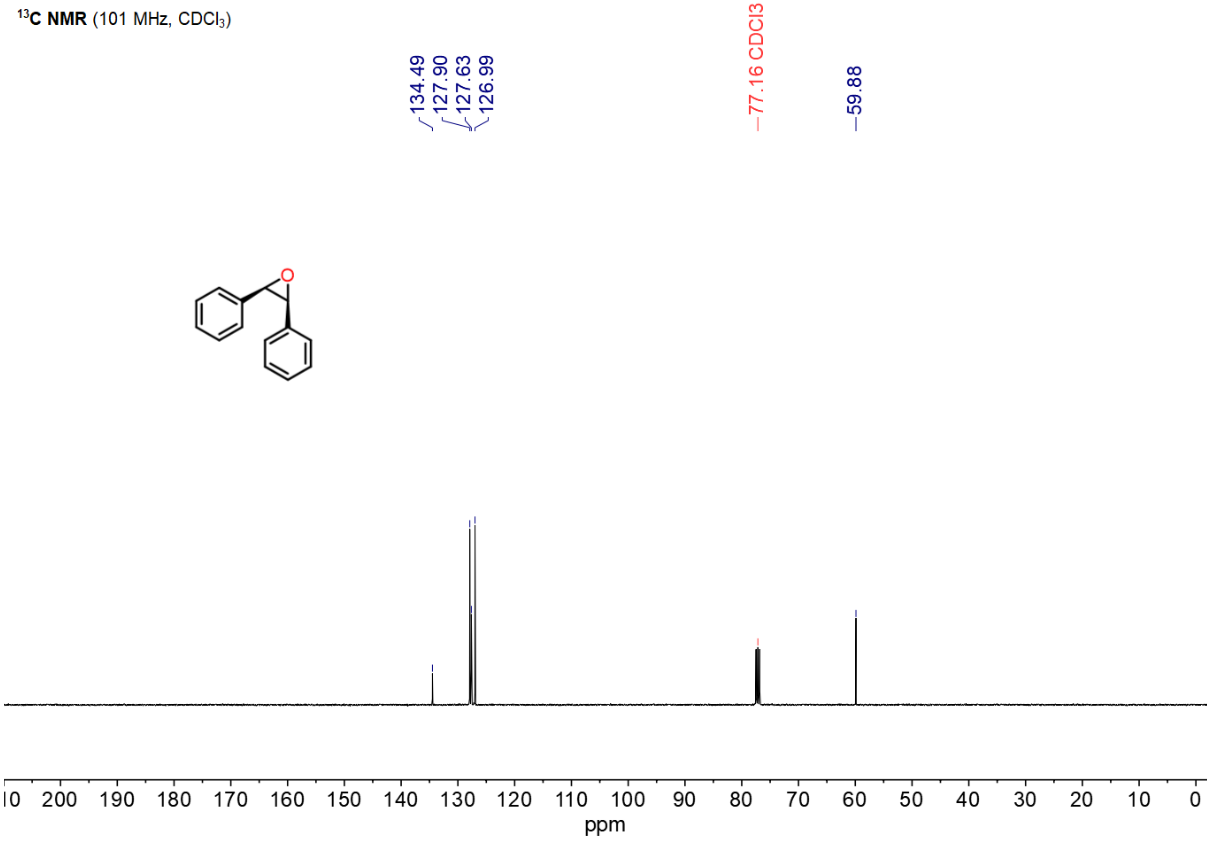

rel-((2*R*,3*R*)-3-phenyloxiran-2-yl)methyl acetate, **4m**:

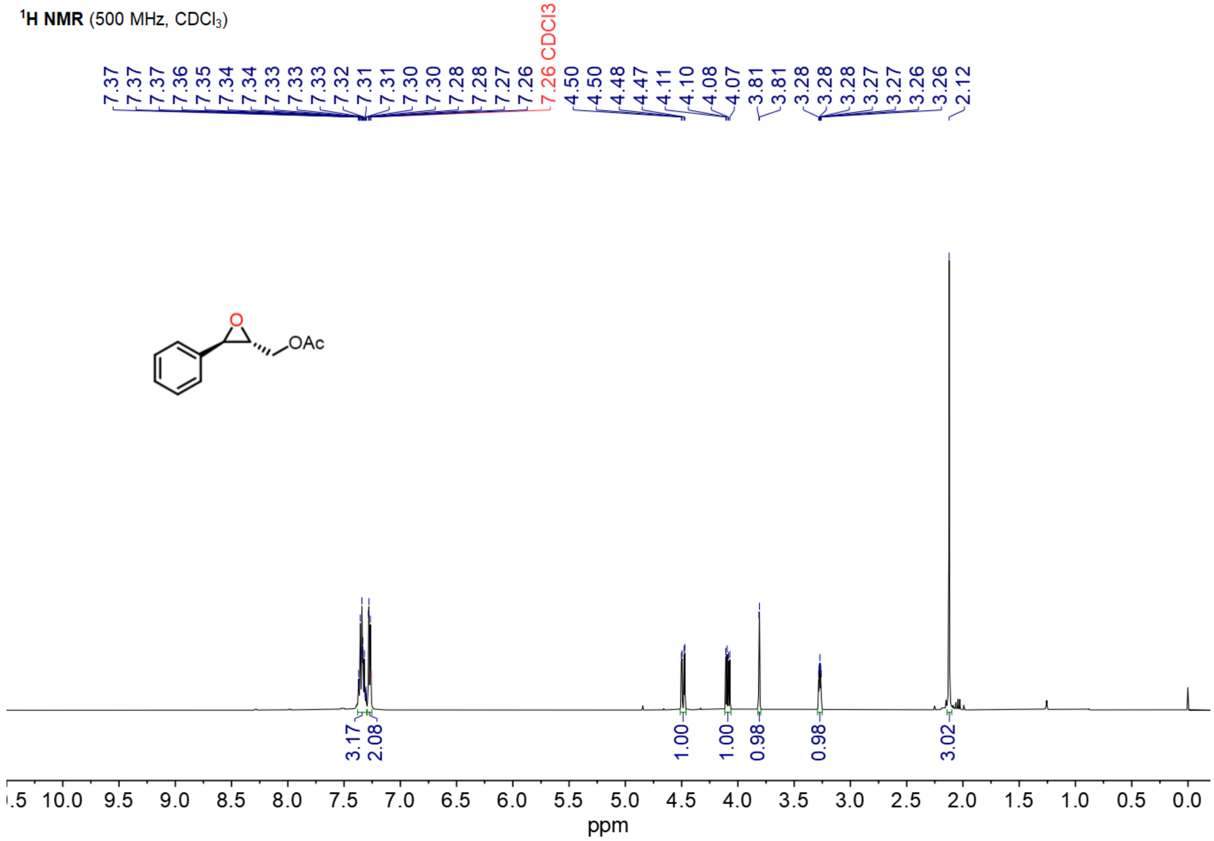

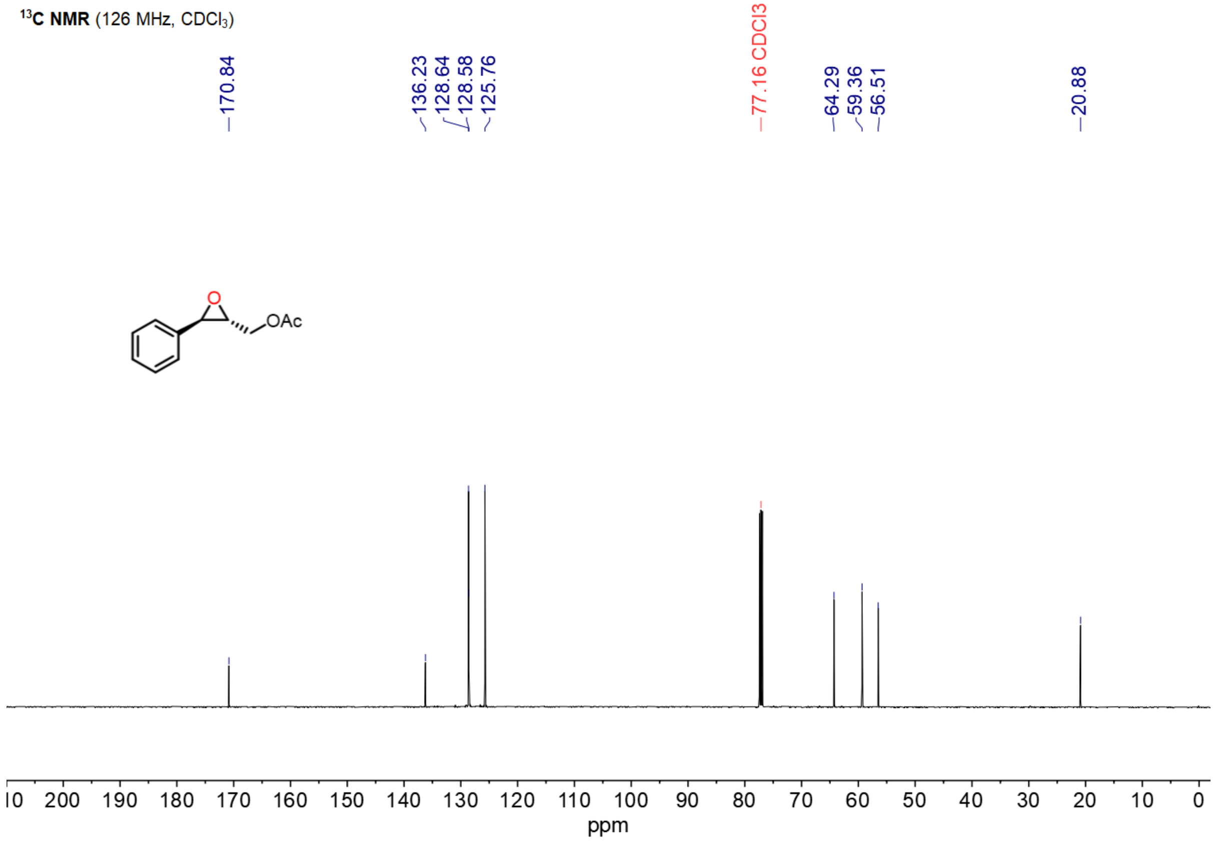

rel-(2*S*,3*R*)-2-(chloromethyl)-3-phenyloxirane, **4n**:

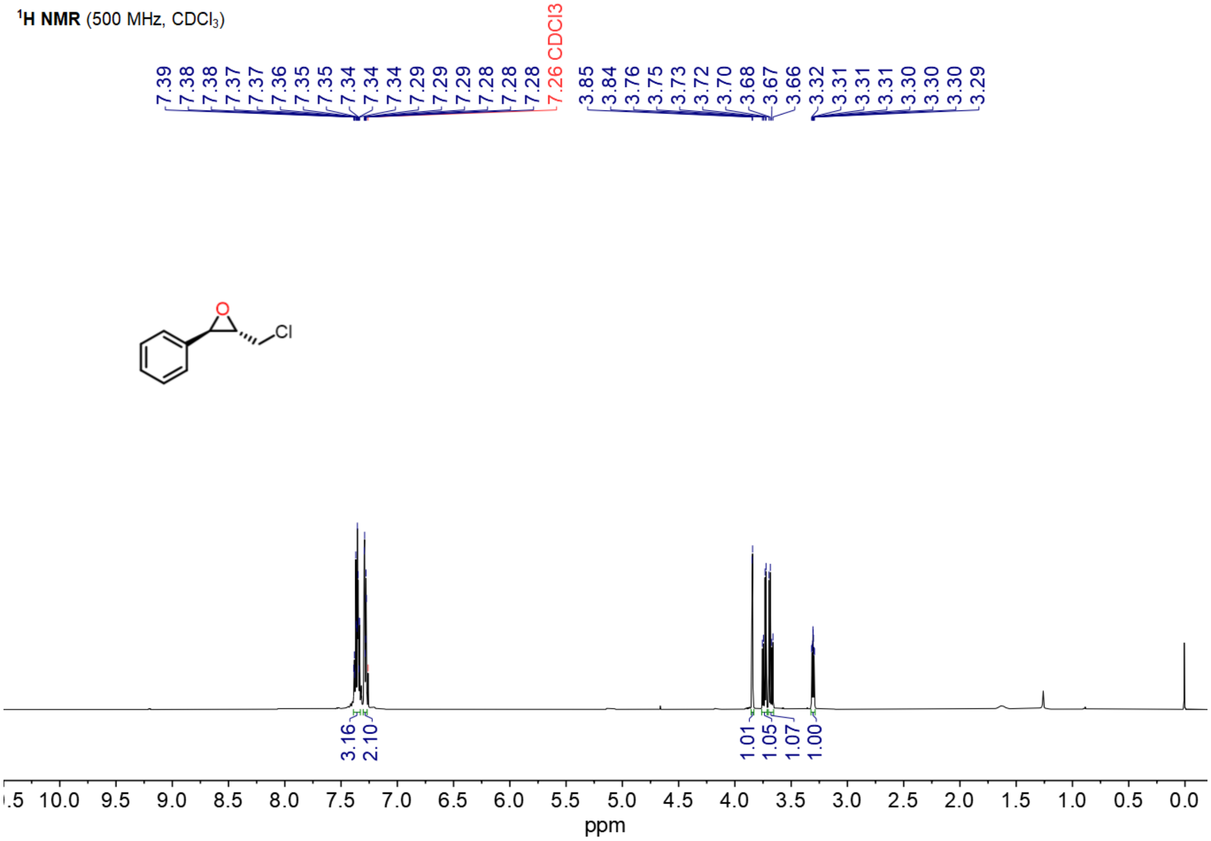

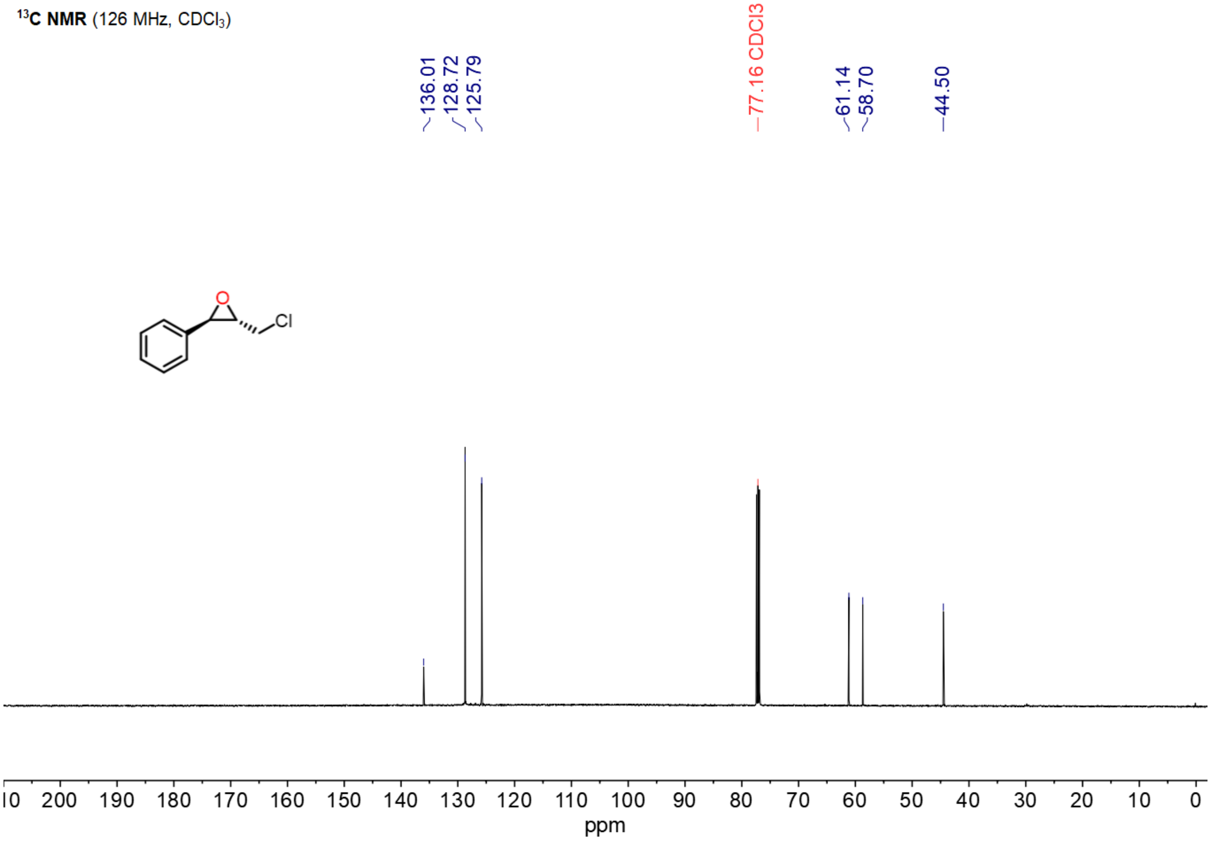

2-phenethyloxirane, **2a**:

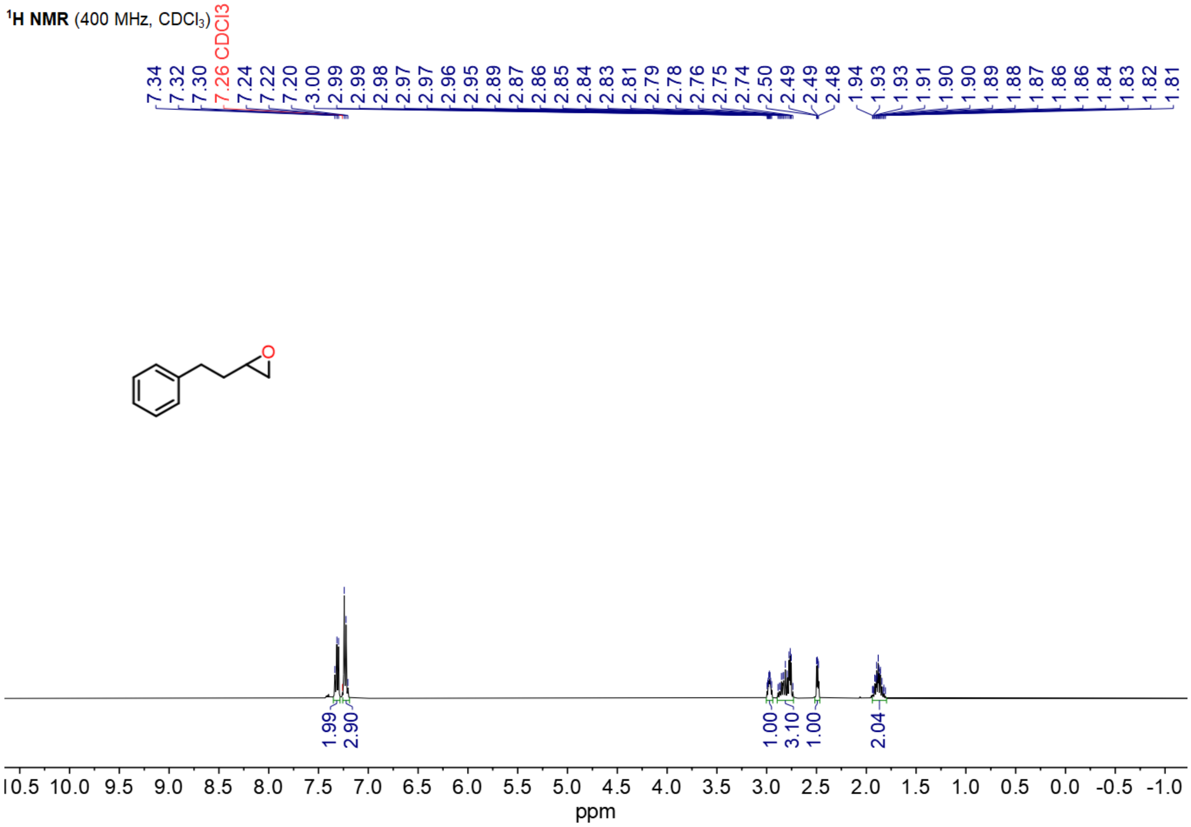

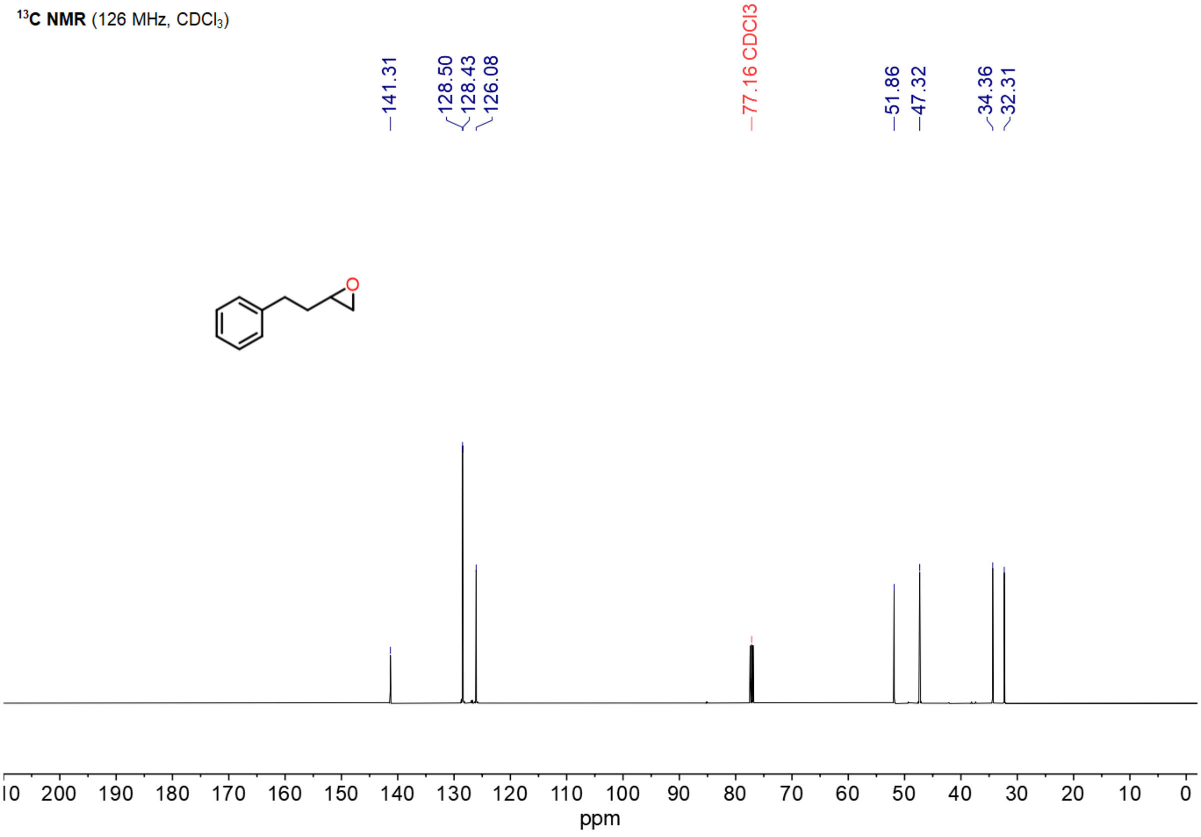

2-benzyloxirane, **4p**:

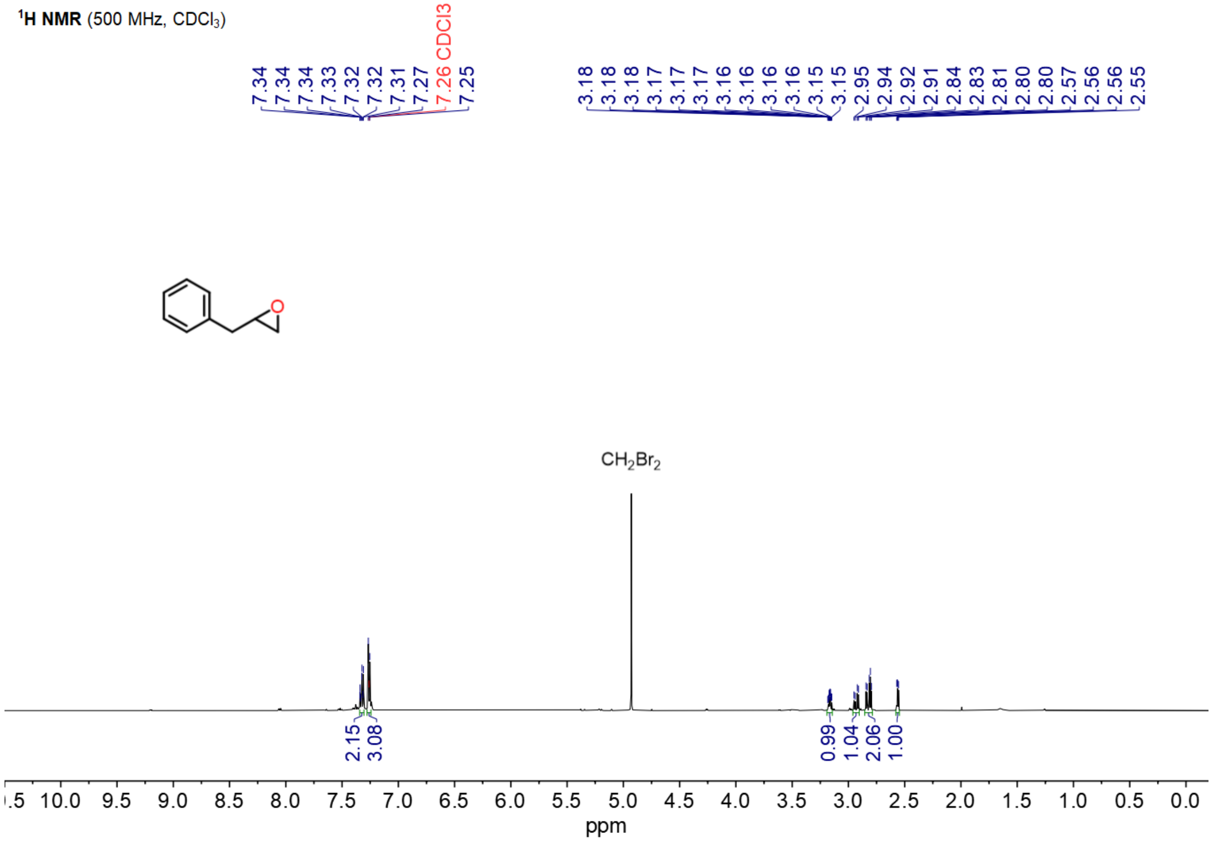

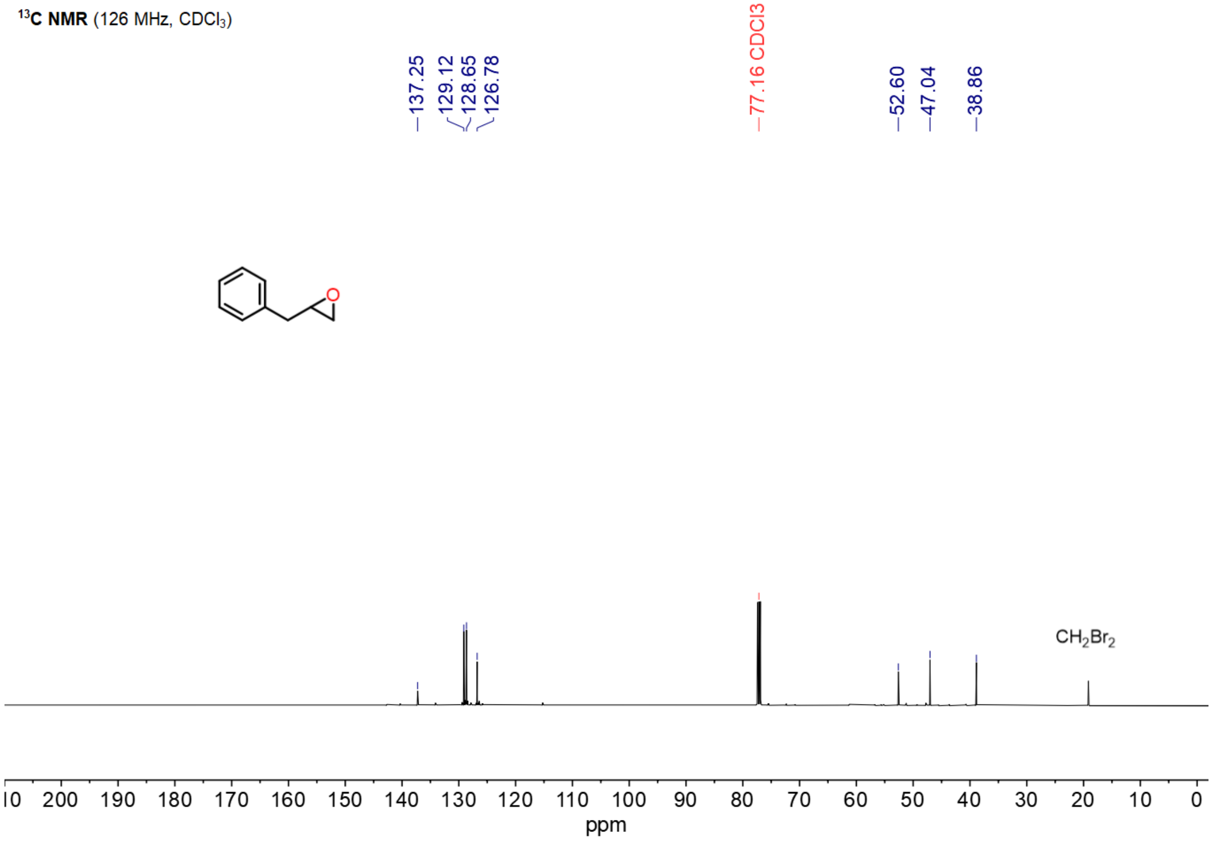

2-(2-bromobenzyl)oxirane, **2b**:

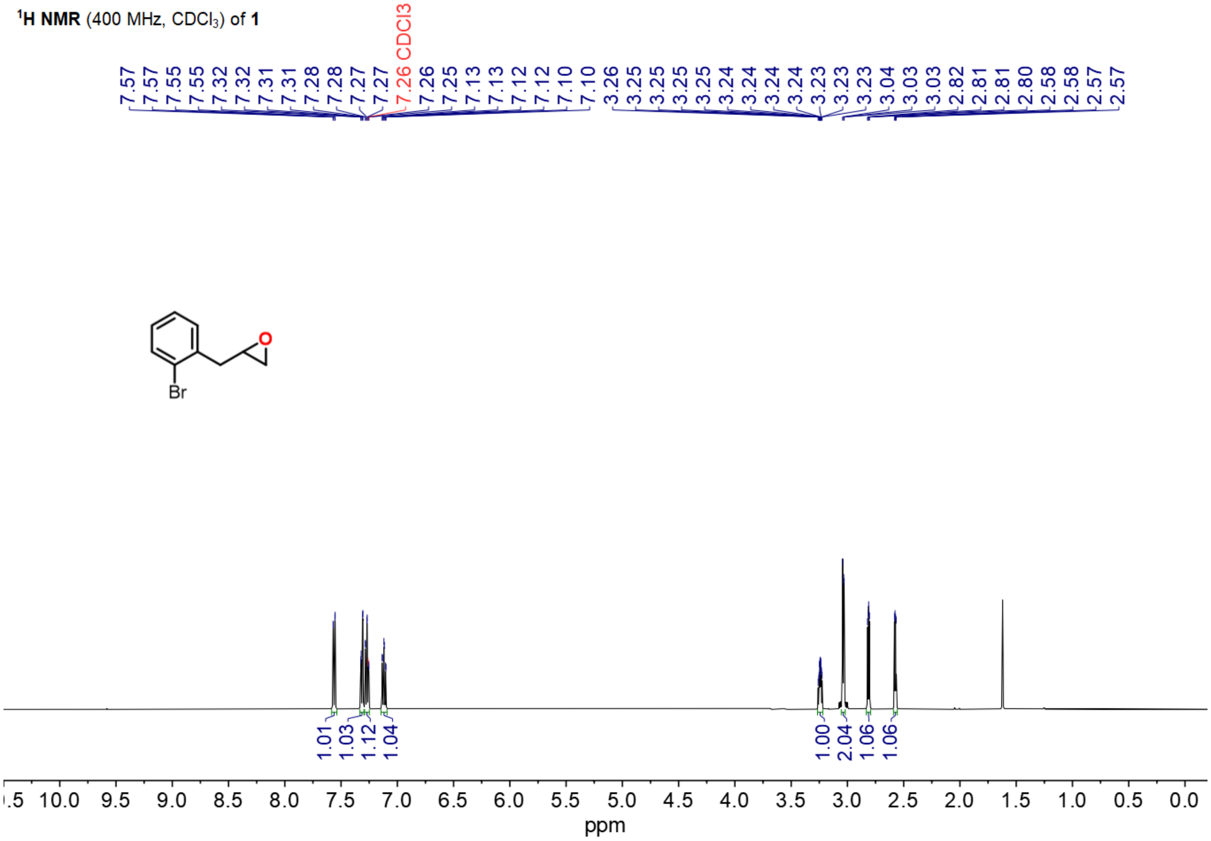

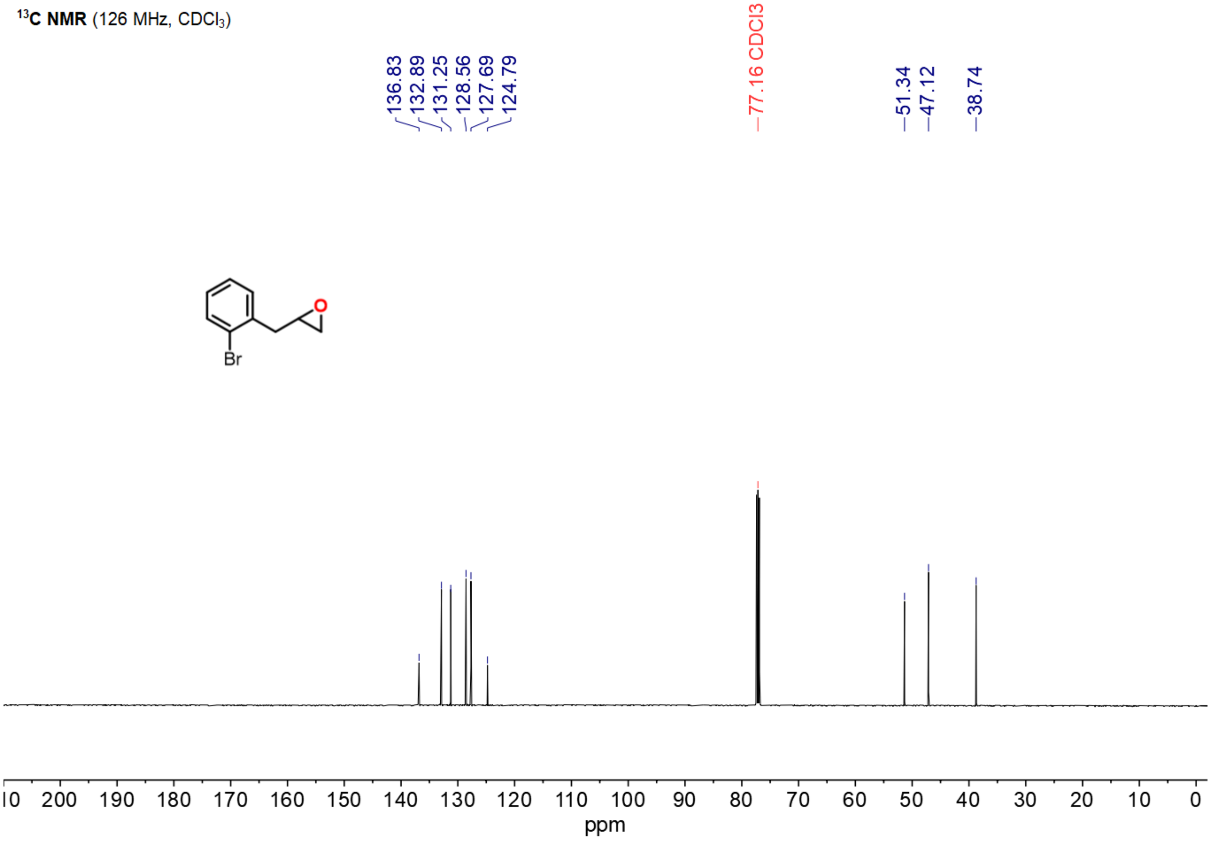

2-benzyl-2-methyloxirane, **4q**:

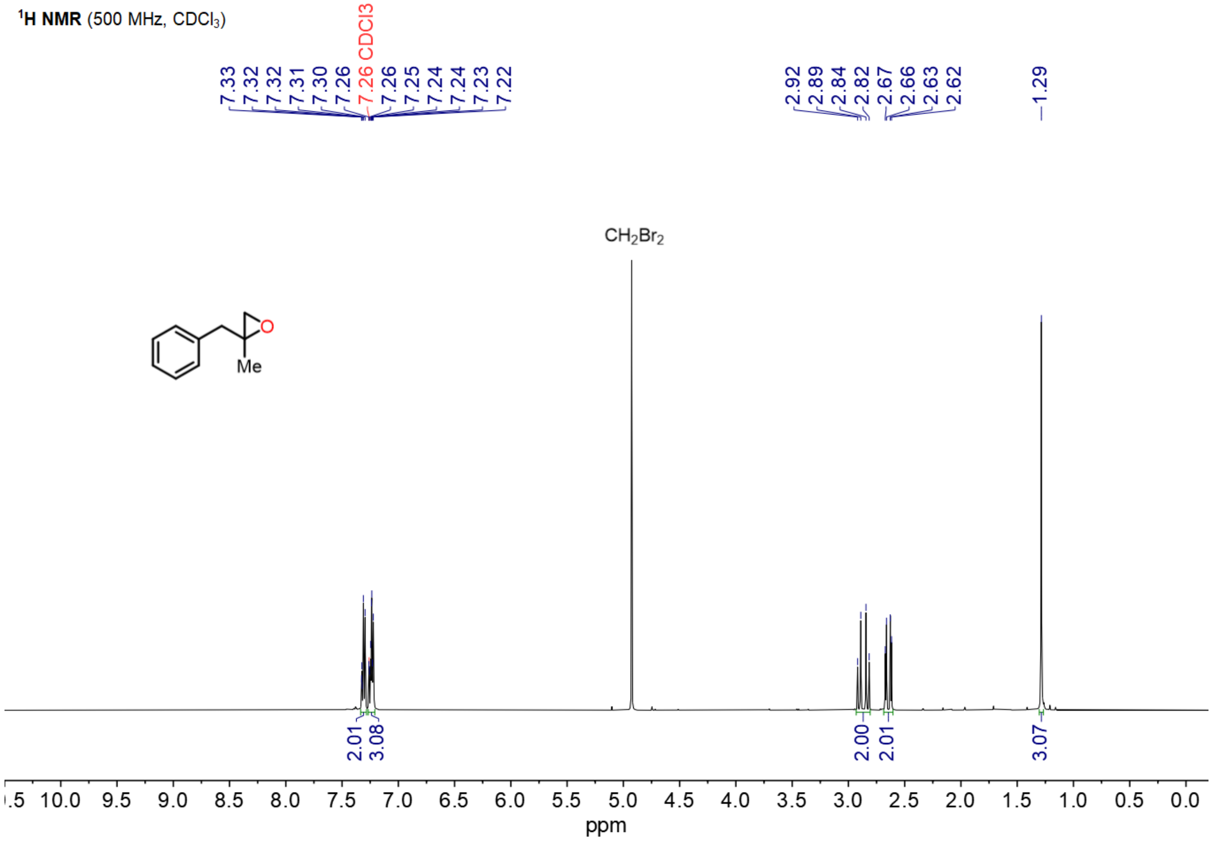

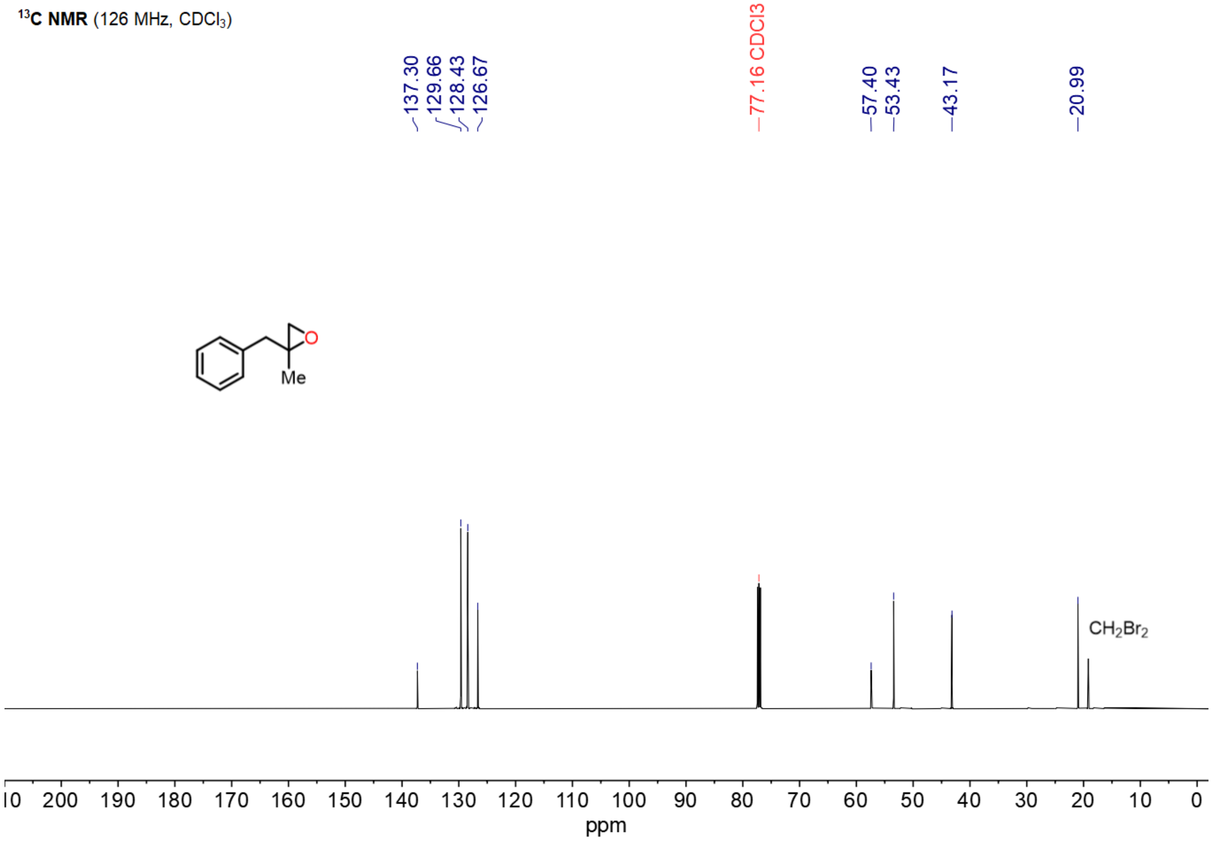

rel-2-((1*S*,2*S*)-2-phenylcyclopropyl)oxirane, **4r**:

^{1}H NMR (500 MHz, $CDCl_3$)

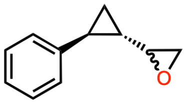

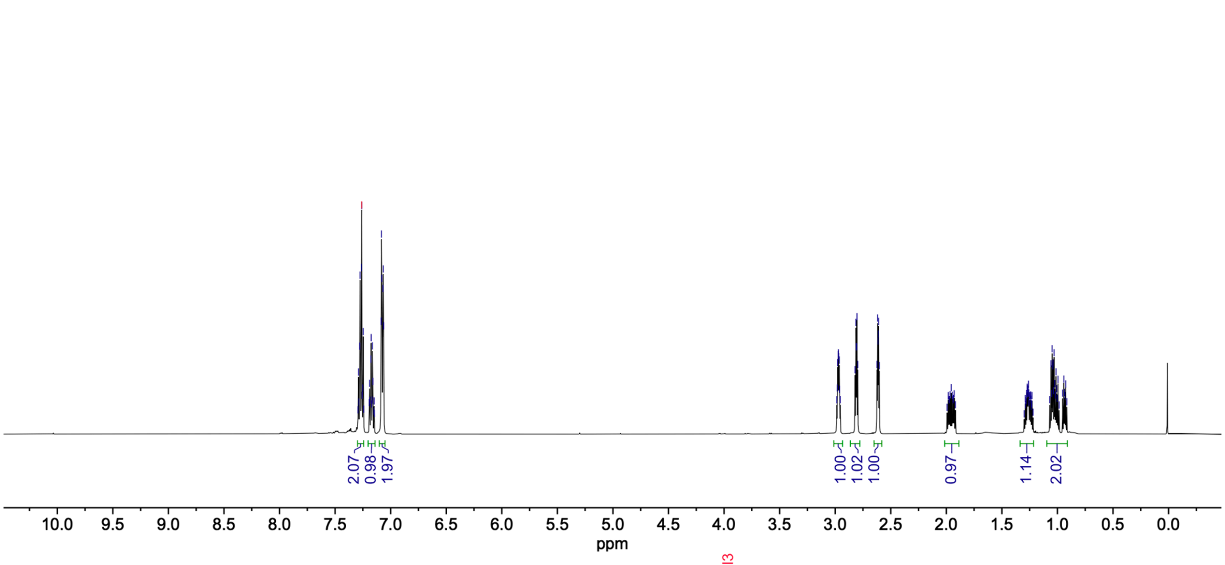

^{13}C NMR (126 MHz, $CDCl_3$)

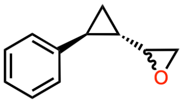

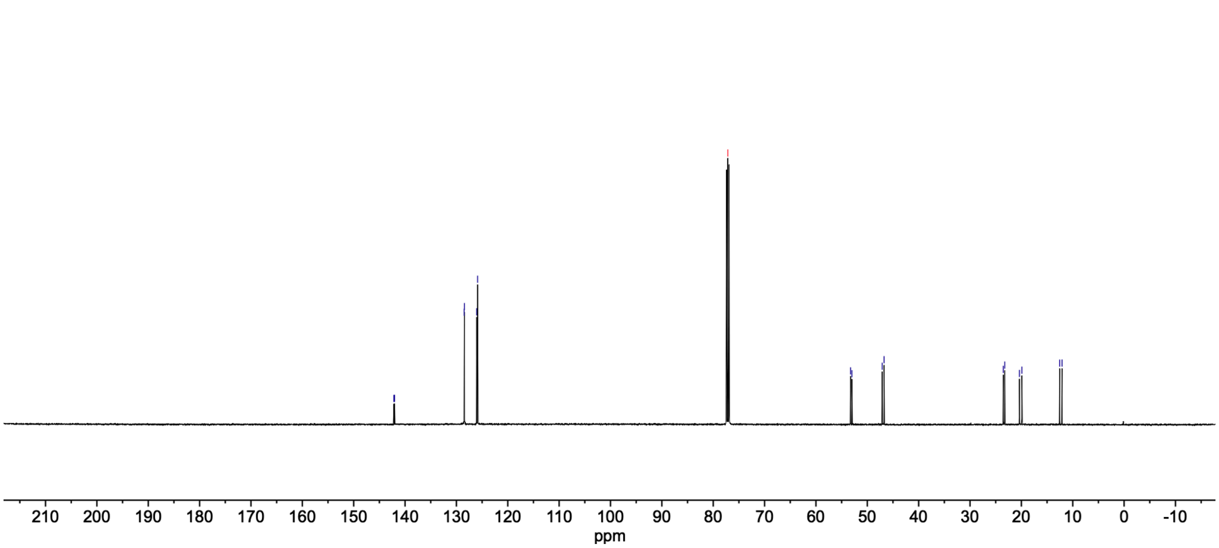

2-hexyloxirane, **4s**:

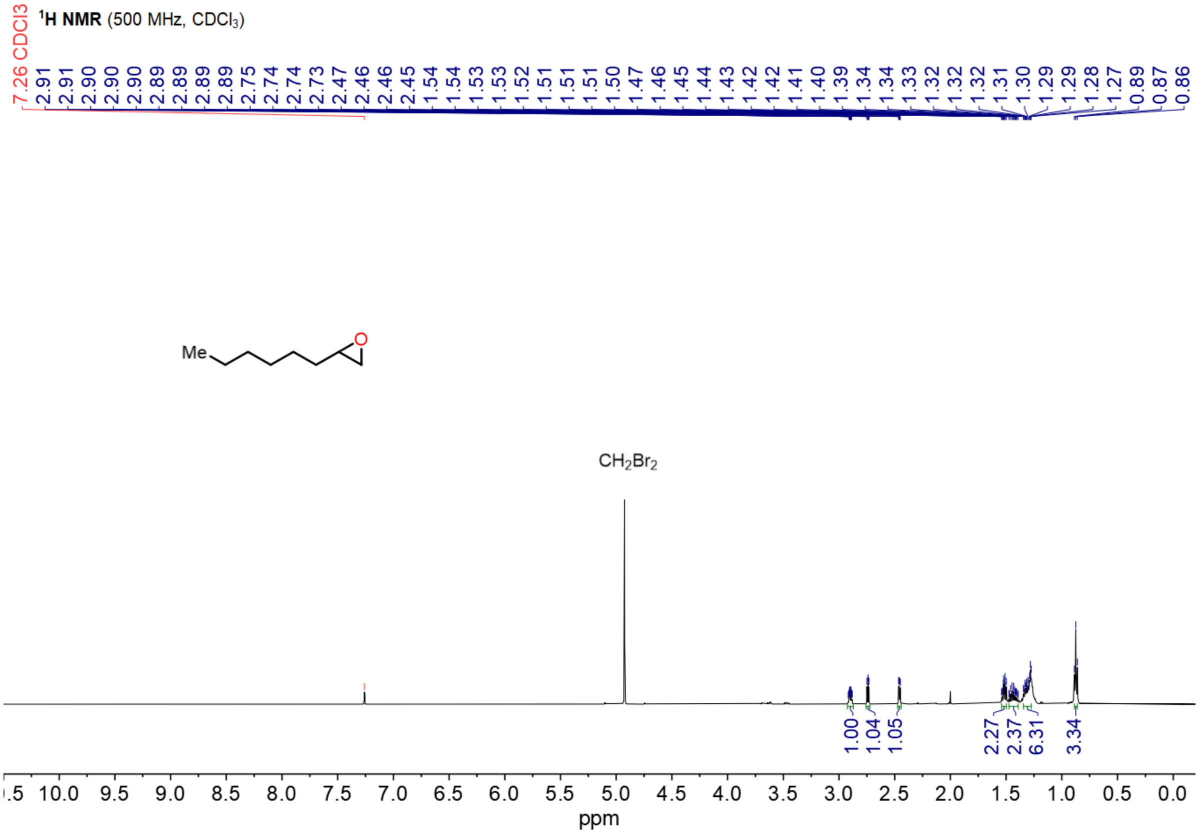

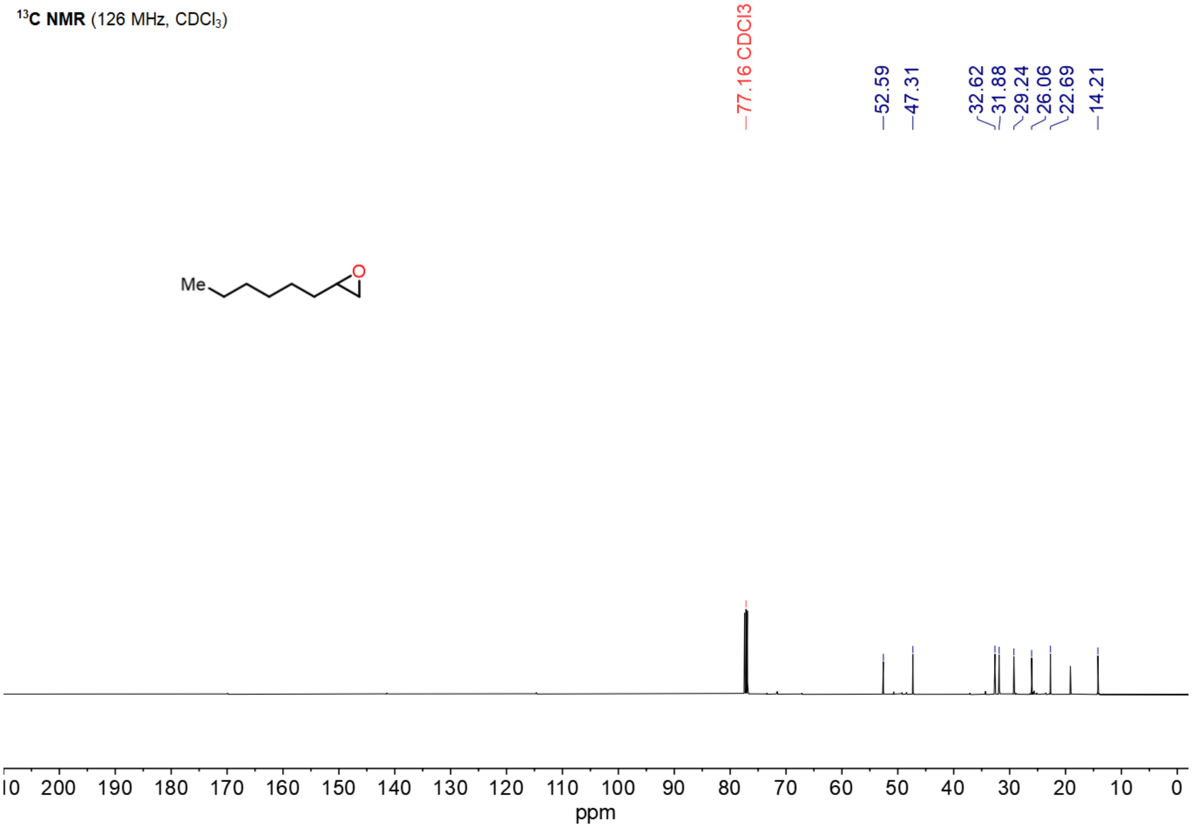

9-oxabicyclo[6.1.0]nonane, **4t**:

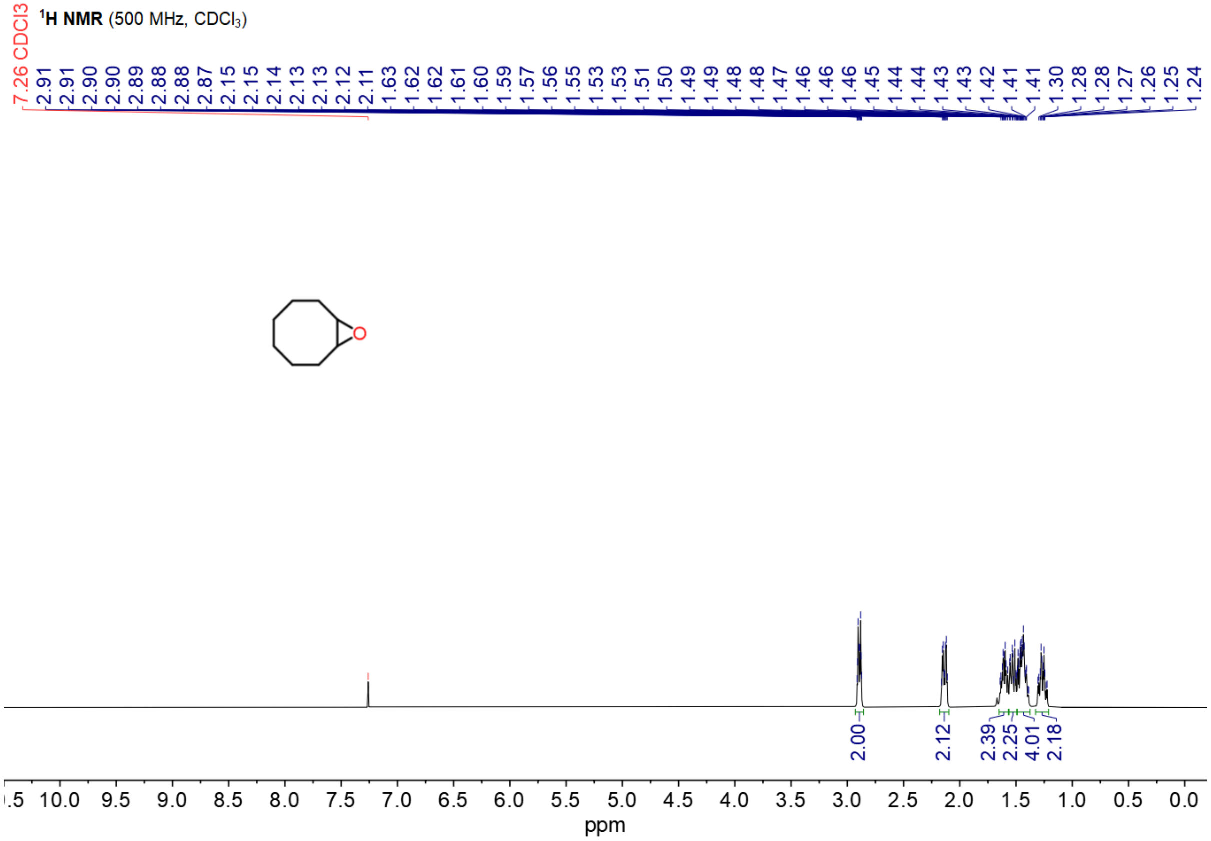

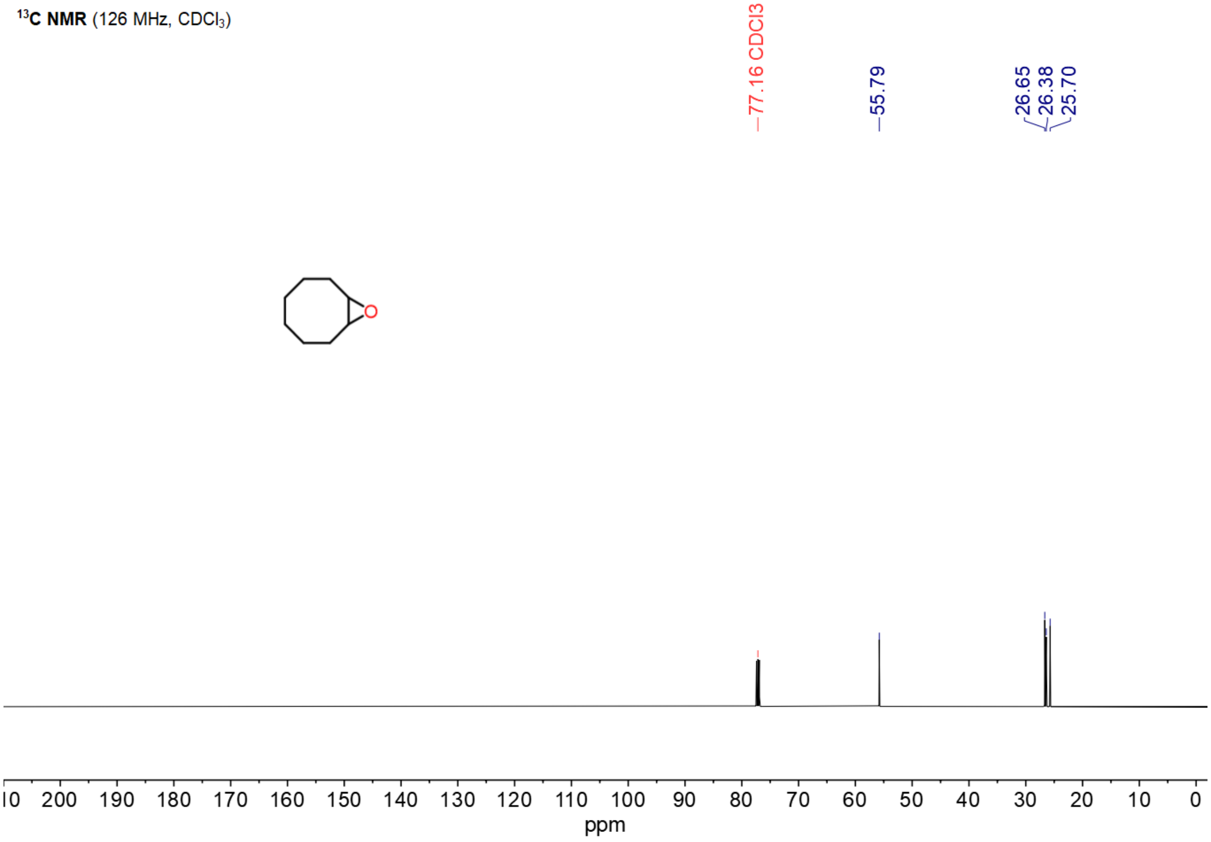

3-(bromomethyl)-2,2-dimethyloxirane, **4u**:

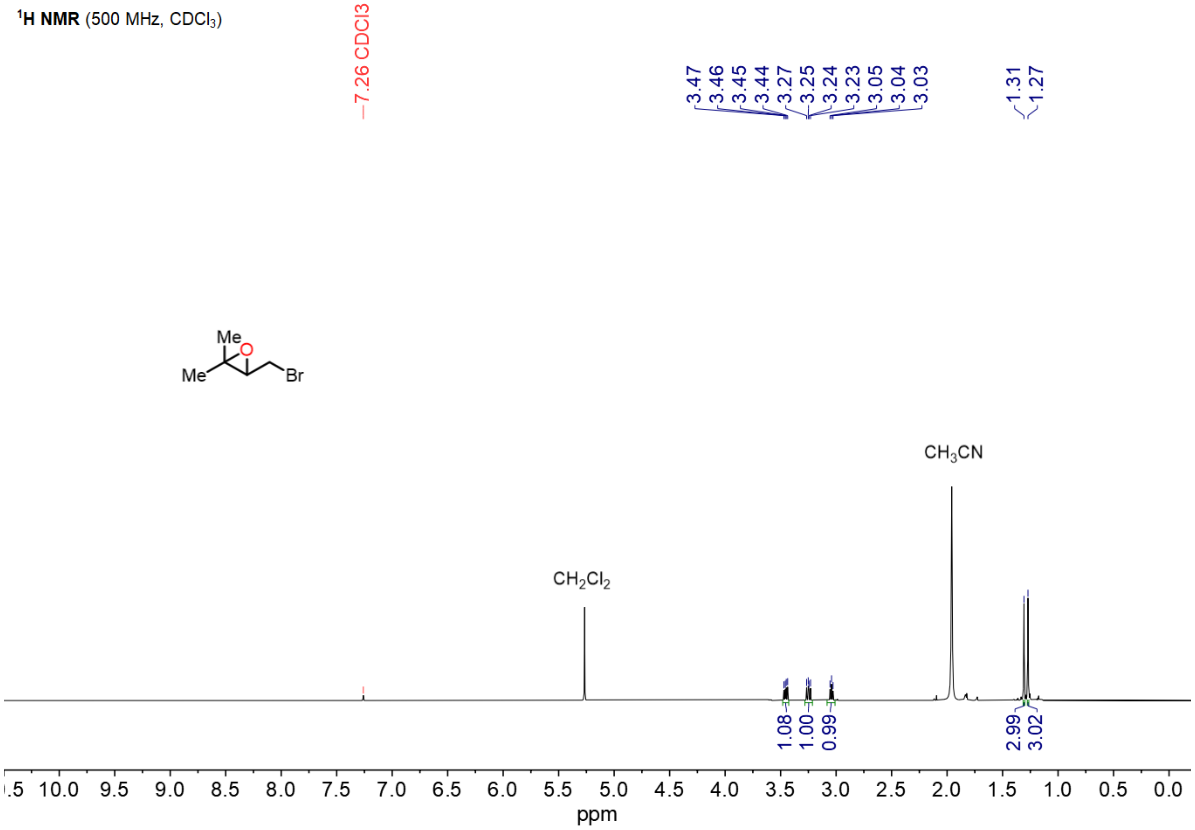

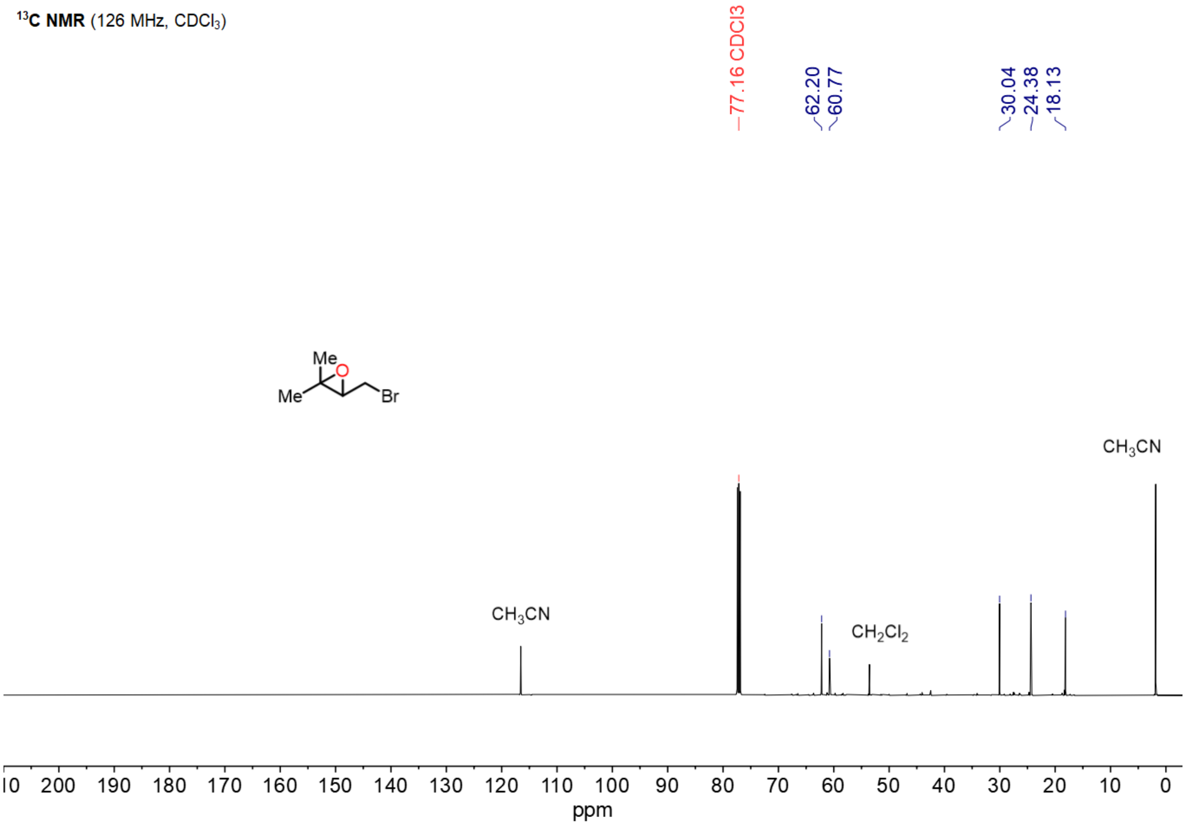

rel-(2*R*,3*R*)-2,3-dibutyloxirane, **2d**:

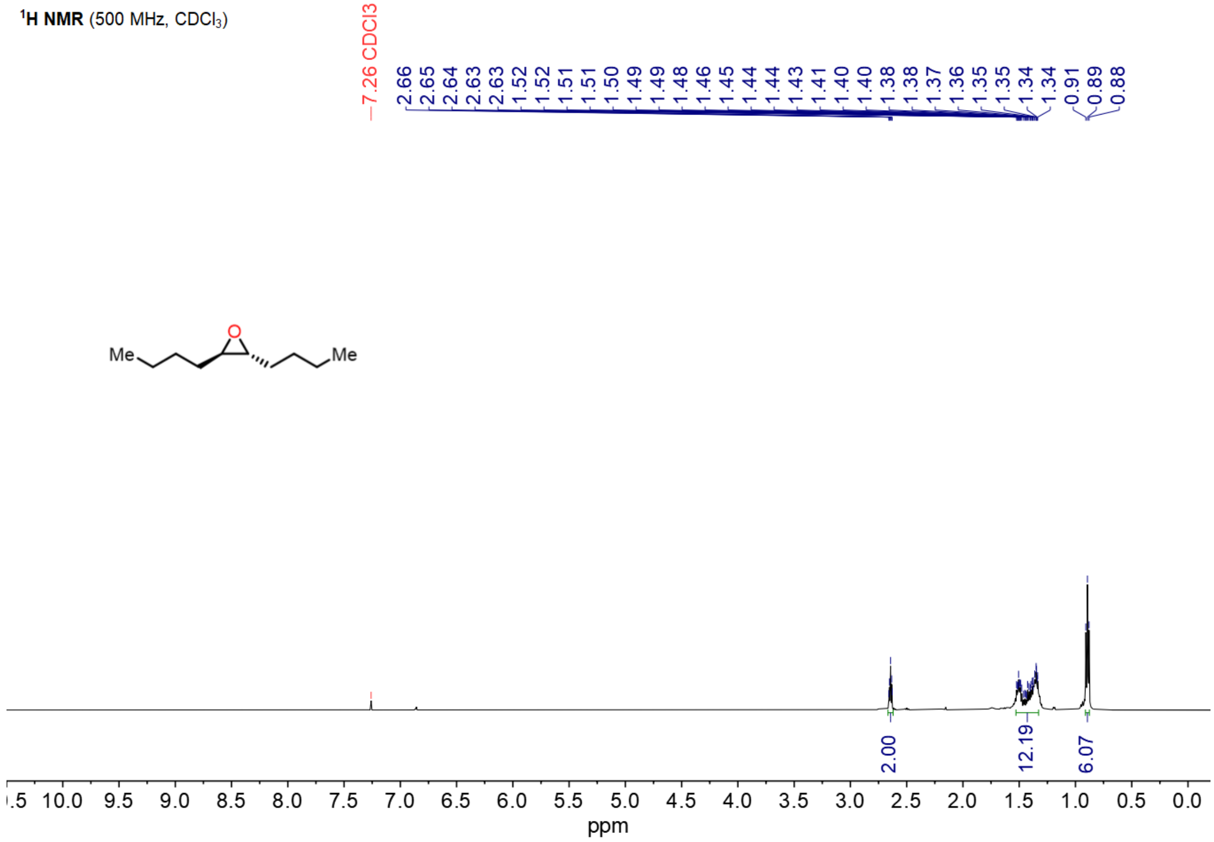

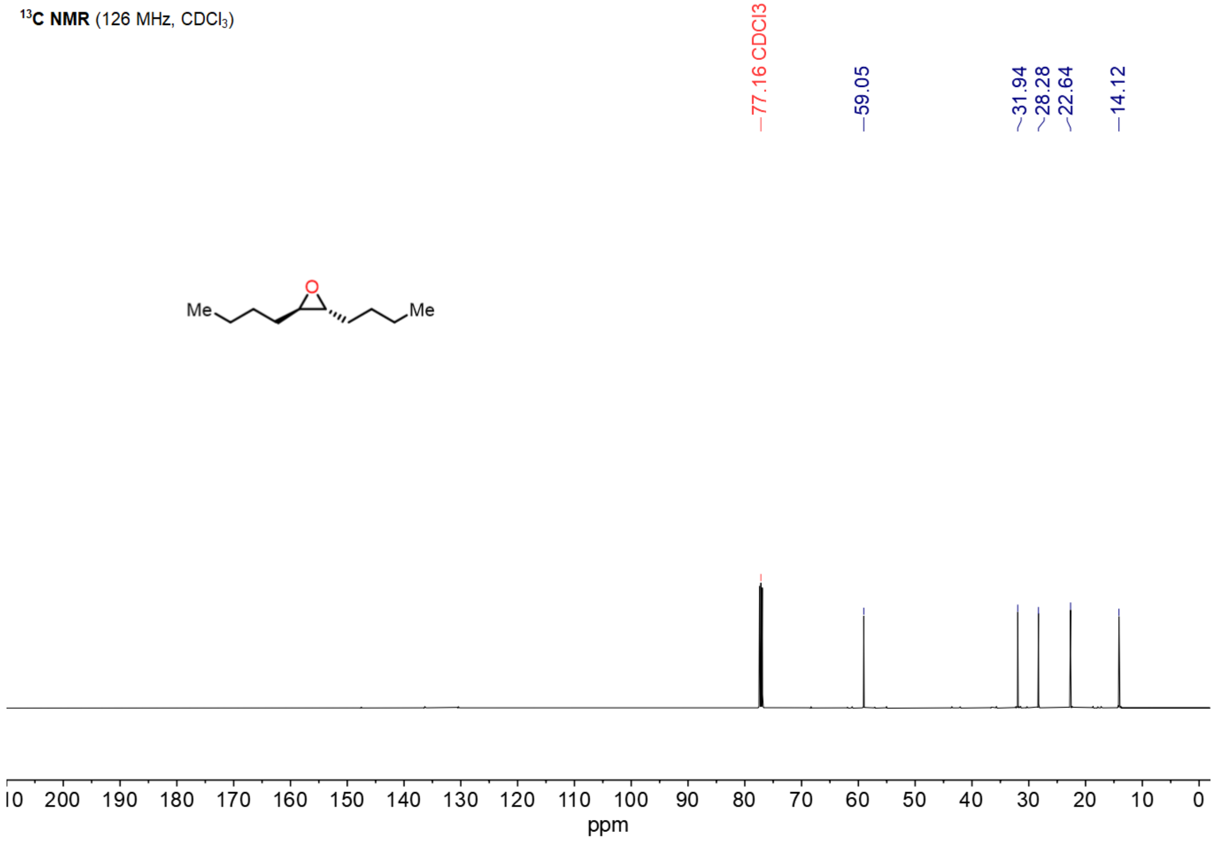

rel-((2*R*,3*R*)-3-propyloxiran-2-yl)methyl acetate, **2e**:

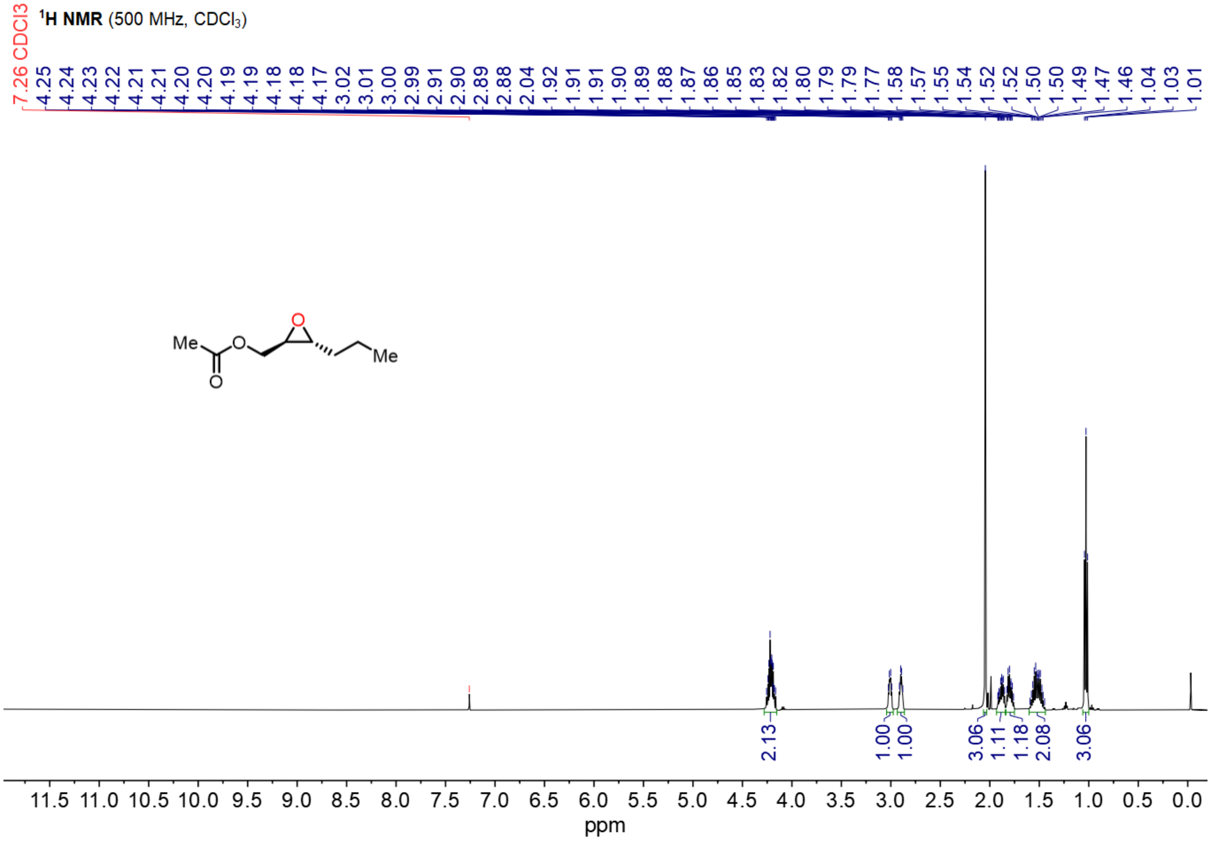

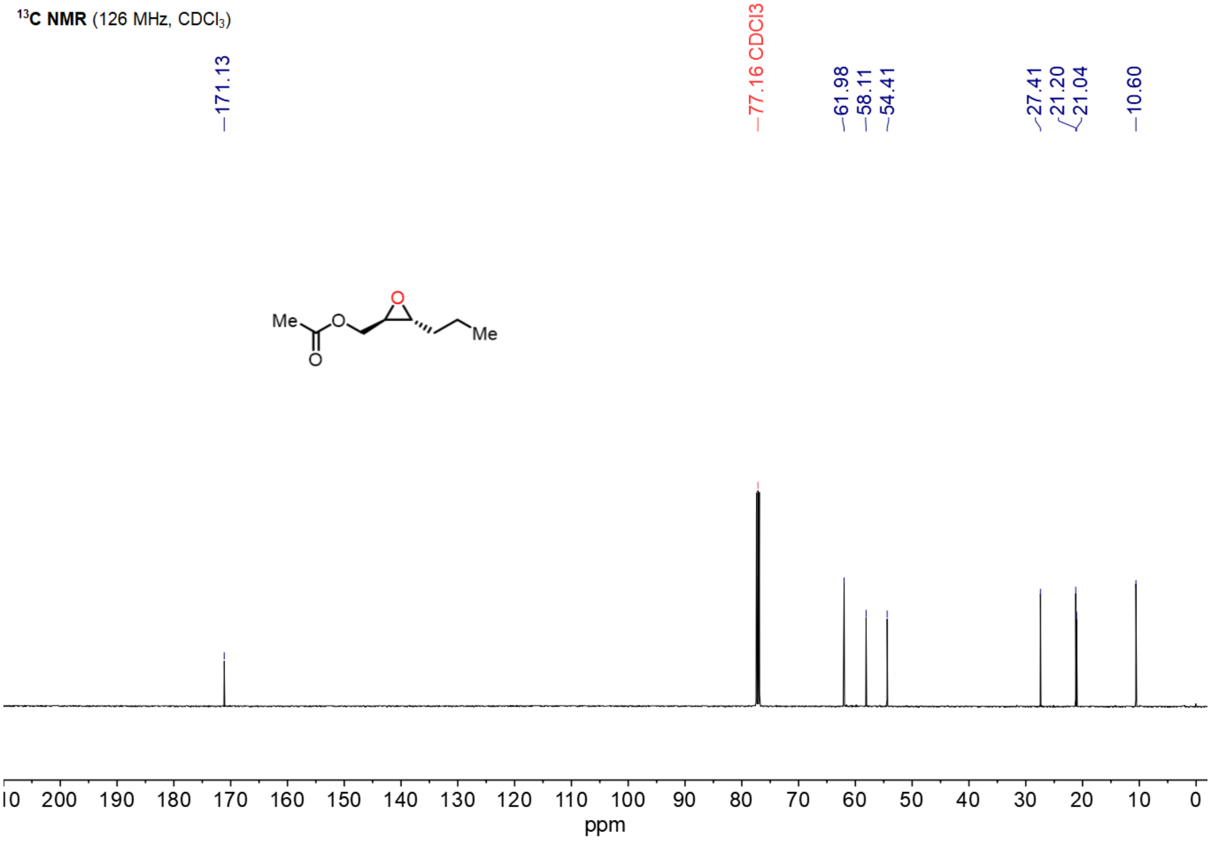

rel-methyl 2-((2*R*,3*R*)-3-pentyloxiran-2-yl)acetate, **4v**:

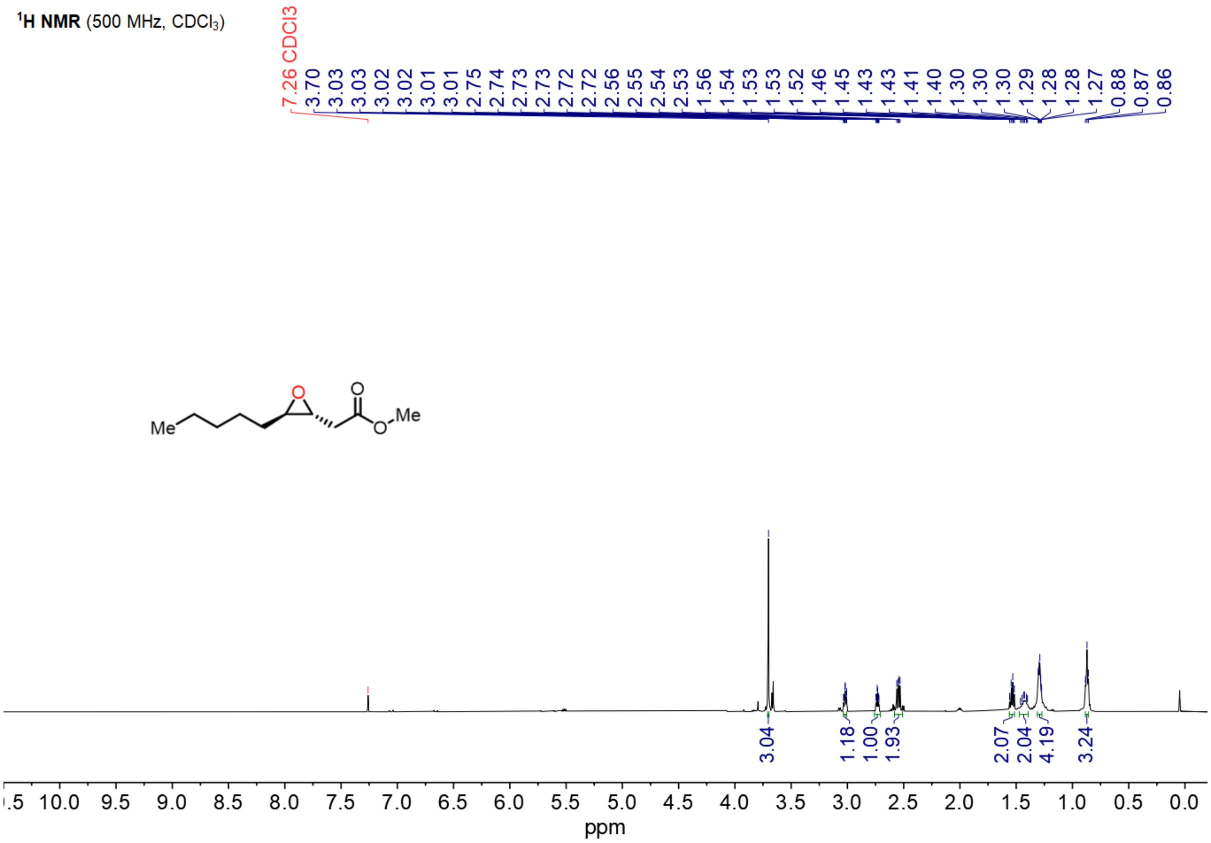

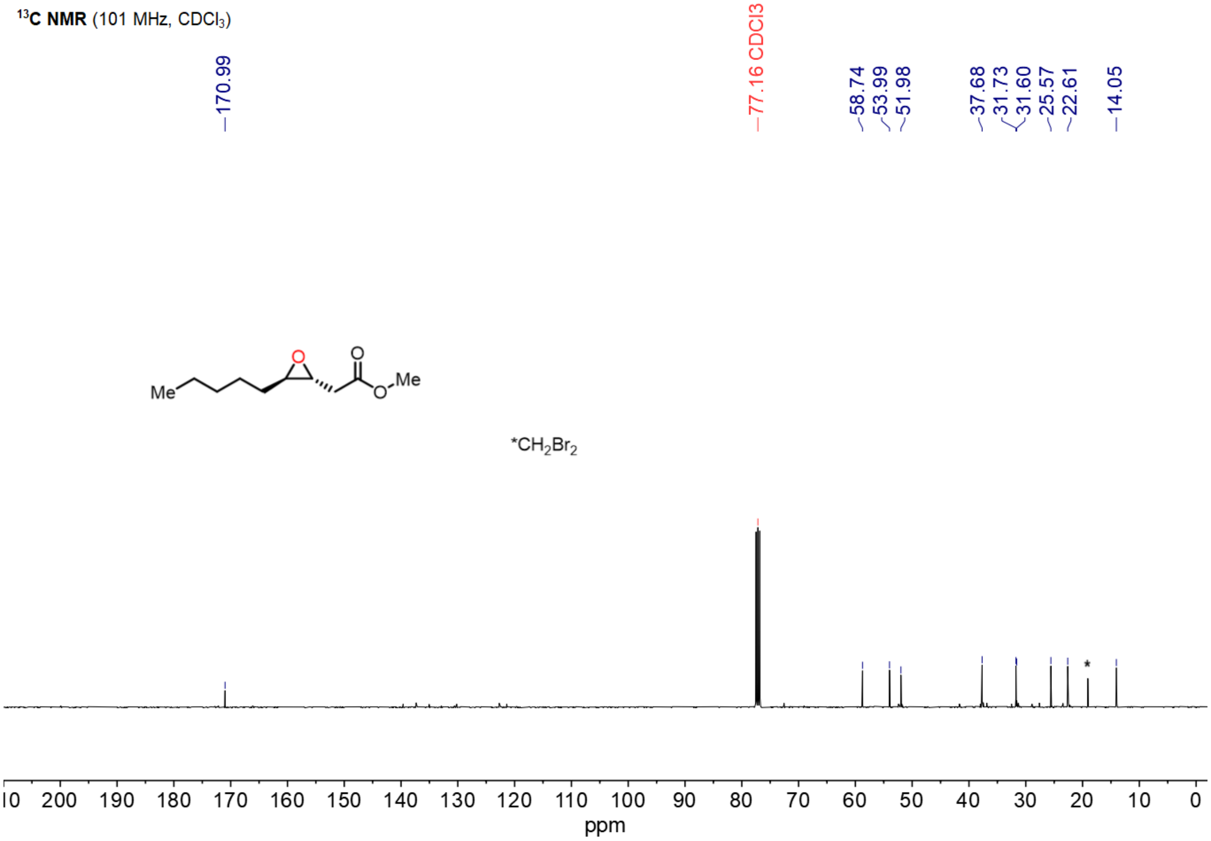

rel-2-((2*S*,3*R*)-3-pentyloxiran-2-yl)ethan-1-ol, **2f**:

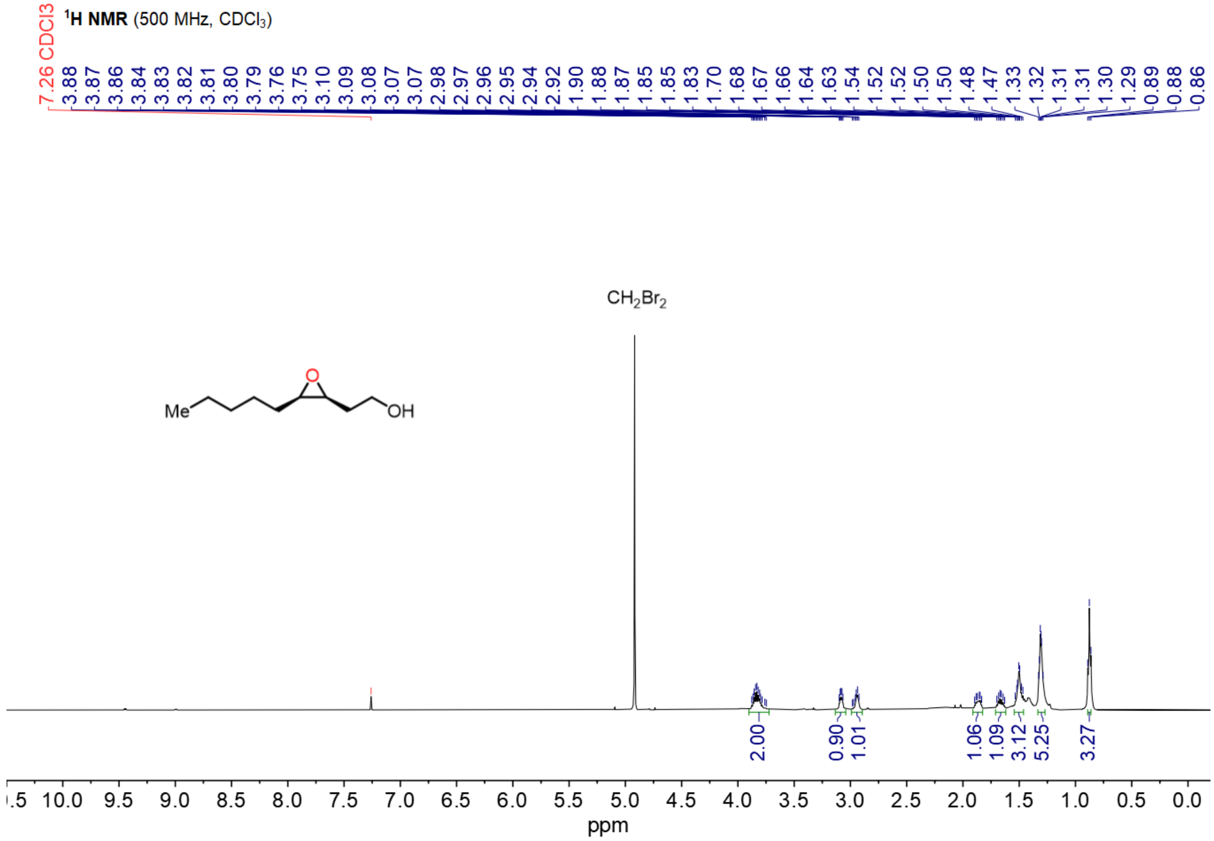

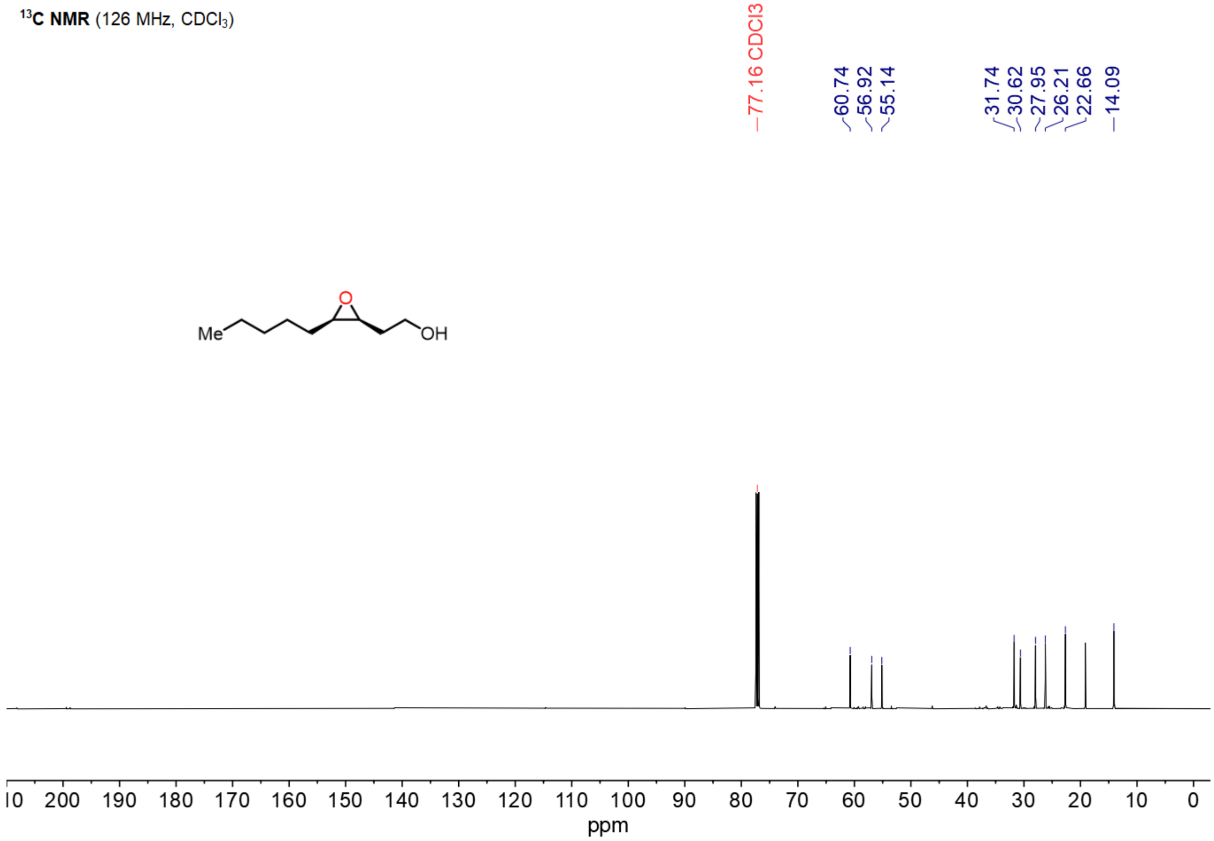

rel-(1*S*,2*S*,4*S*,6*S*)-2,7,7-trimethyl-3-oxatricyclo[4.1.1.02,4]octane, **4w**:

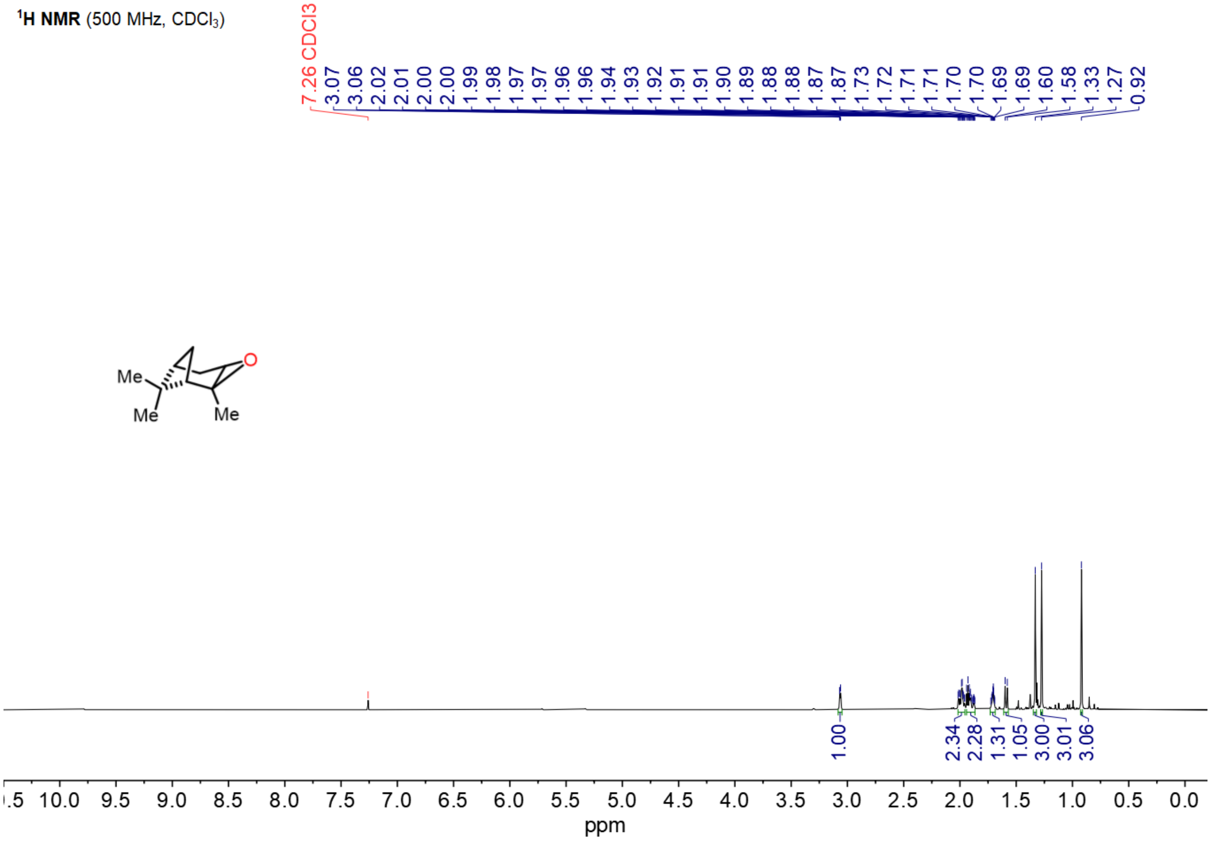

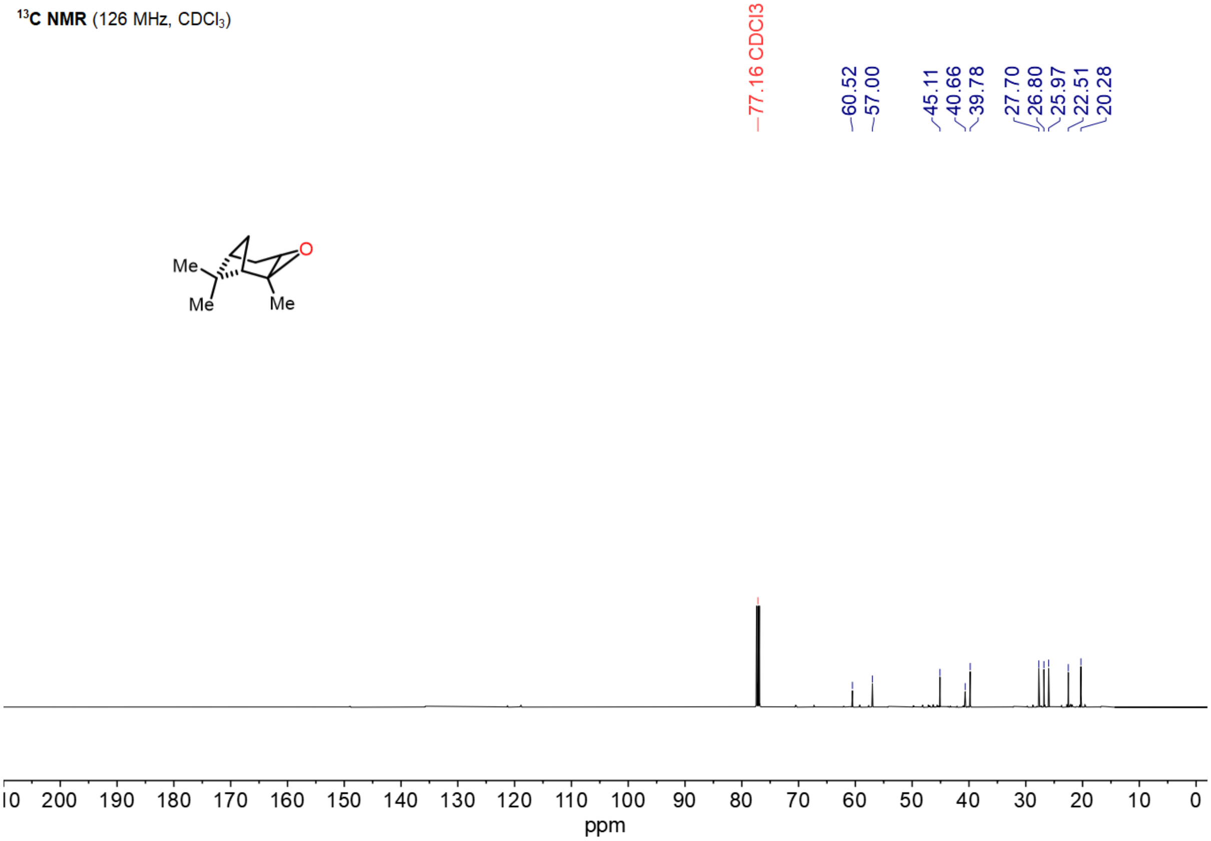

rel-(3*S*,6a*R*,6b*S*,9a*S*,11a*S*,11b*R*)-9a,11b-dimethyl-9-oxohexadecahydrocyclopenta[1,2]phenanthro[8a,9-b]oxiren-3-yl acetate, **4x**:

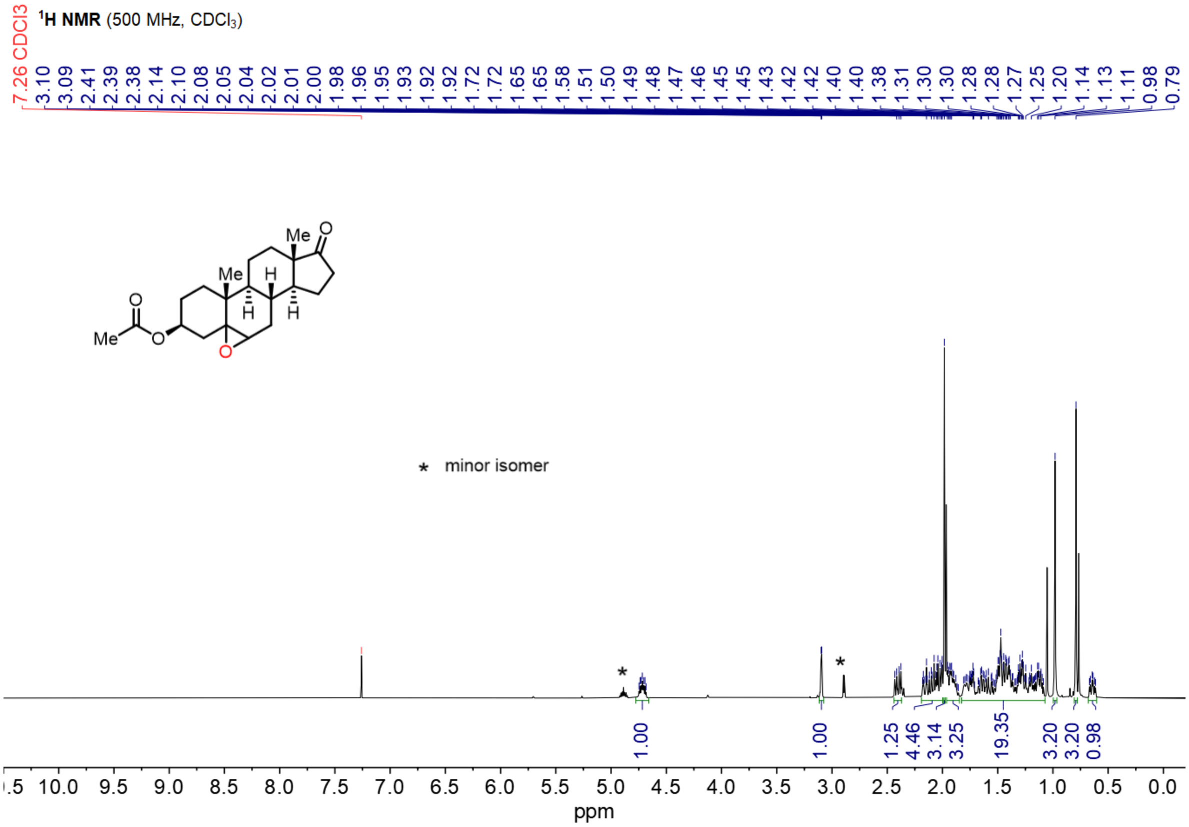

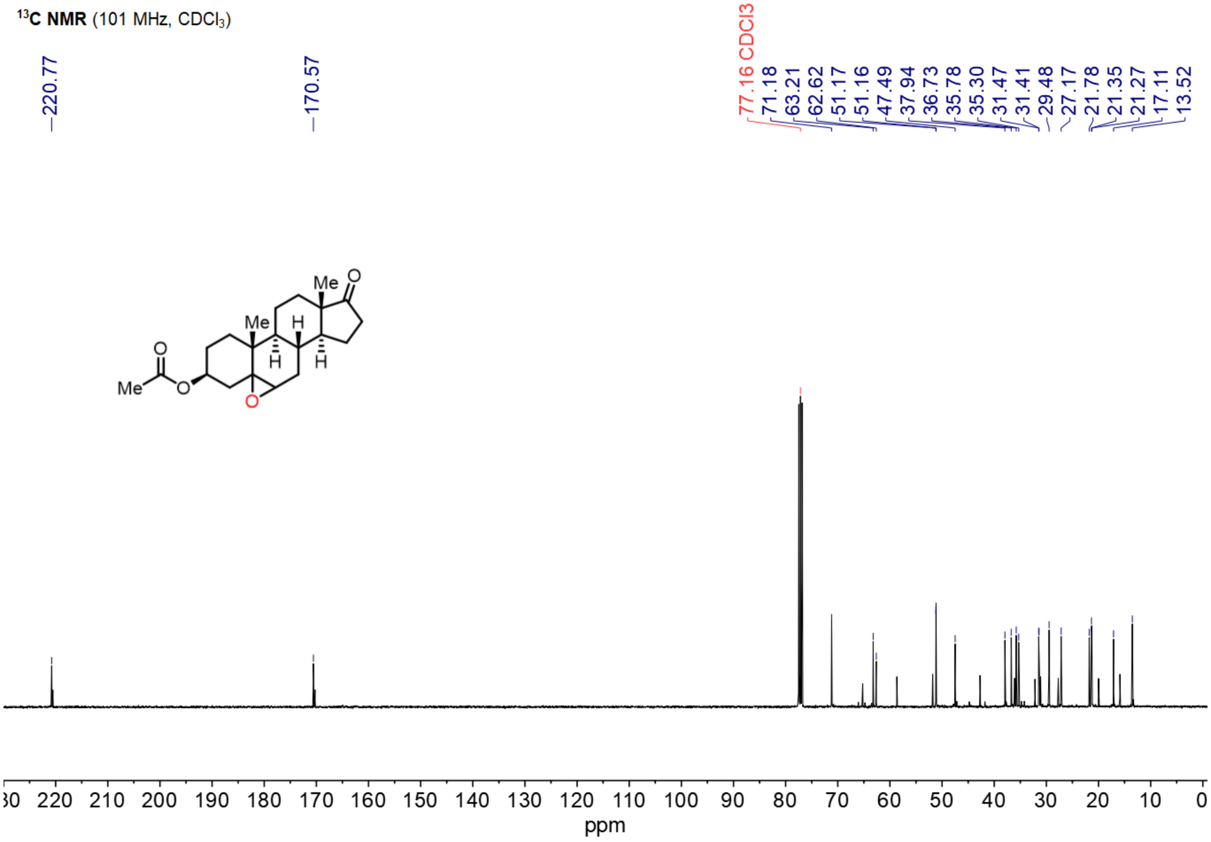

((2*R*,3*R*)-3-phenyloxiran-2-yl)methanol, **4y**:

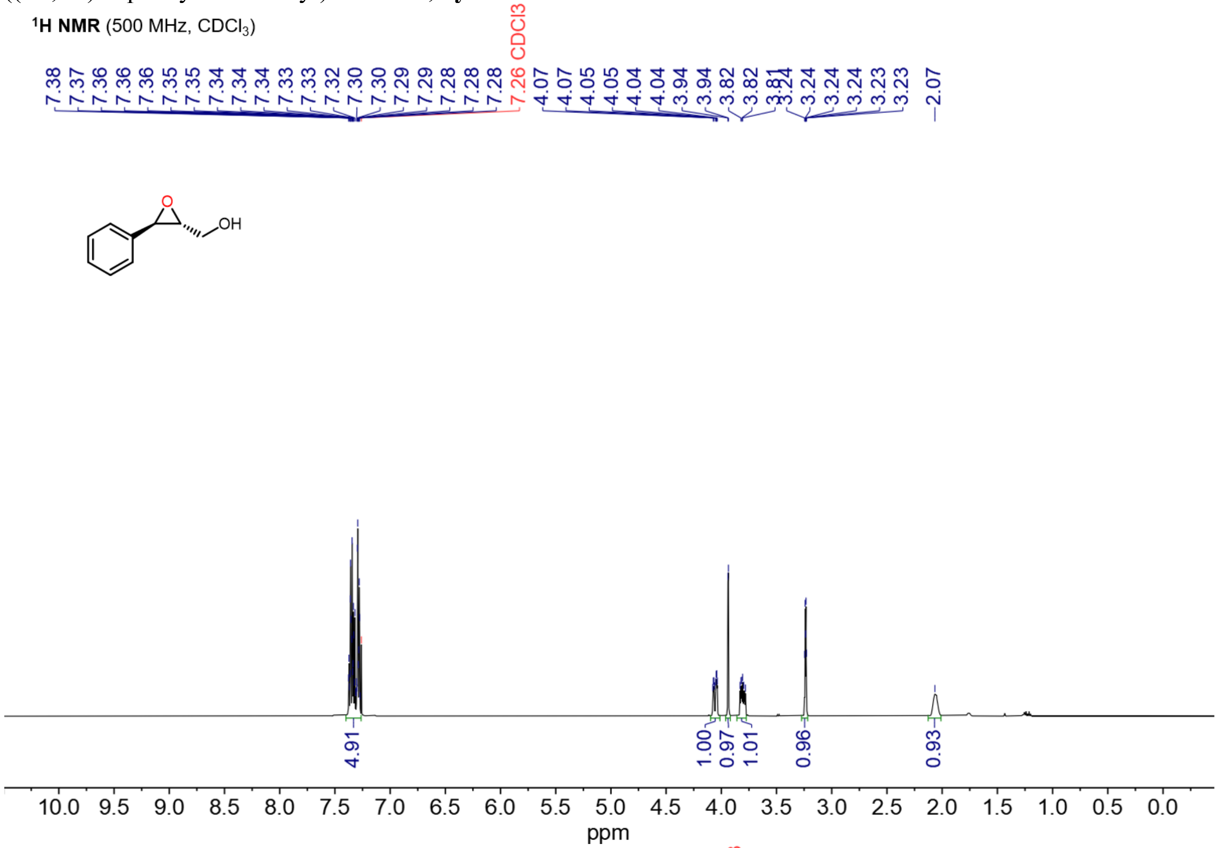

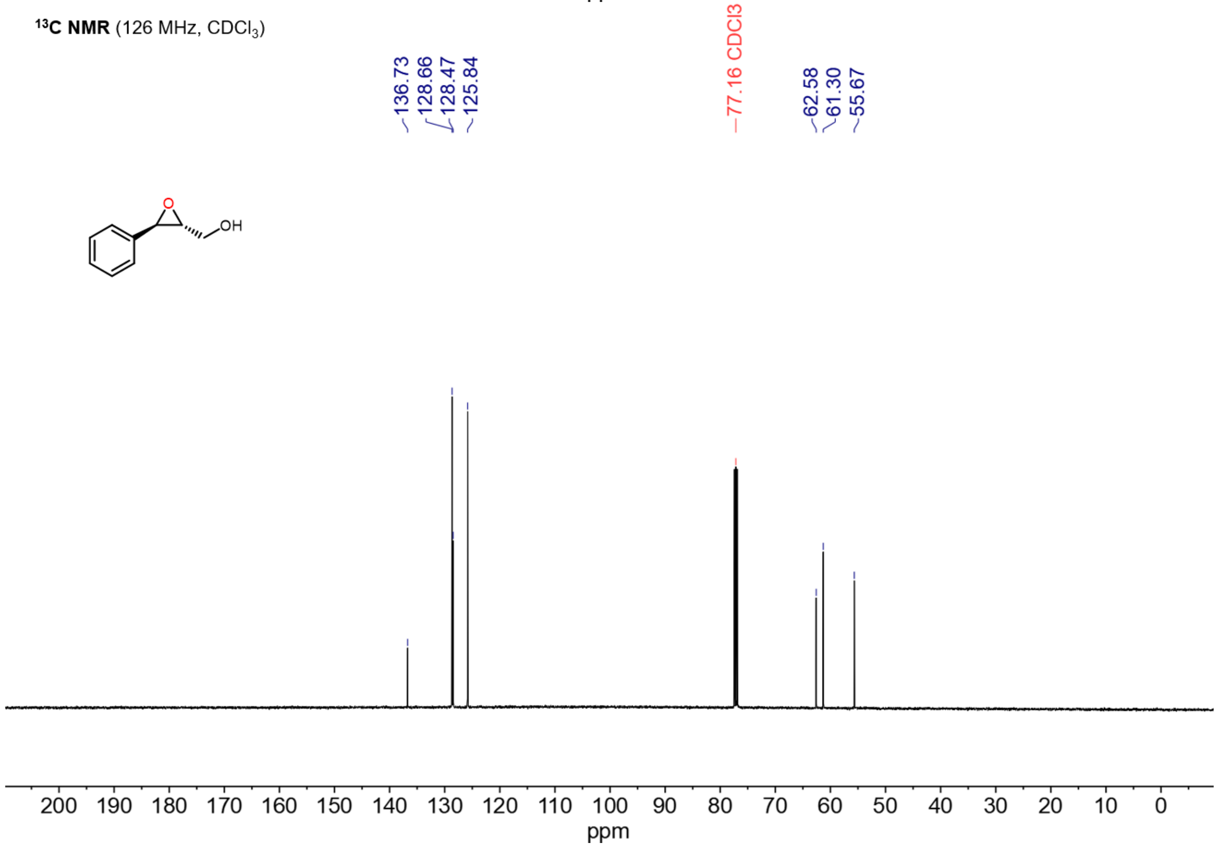

2-(2-(oxiran-2-yl)ethyl)isoindoline-1,3-dione, **4z**:

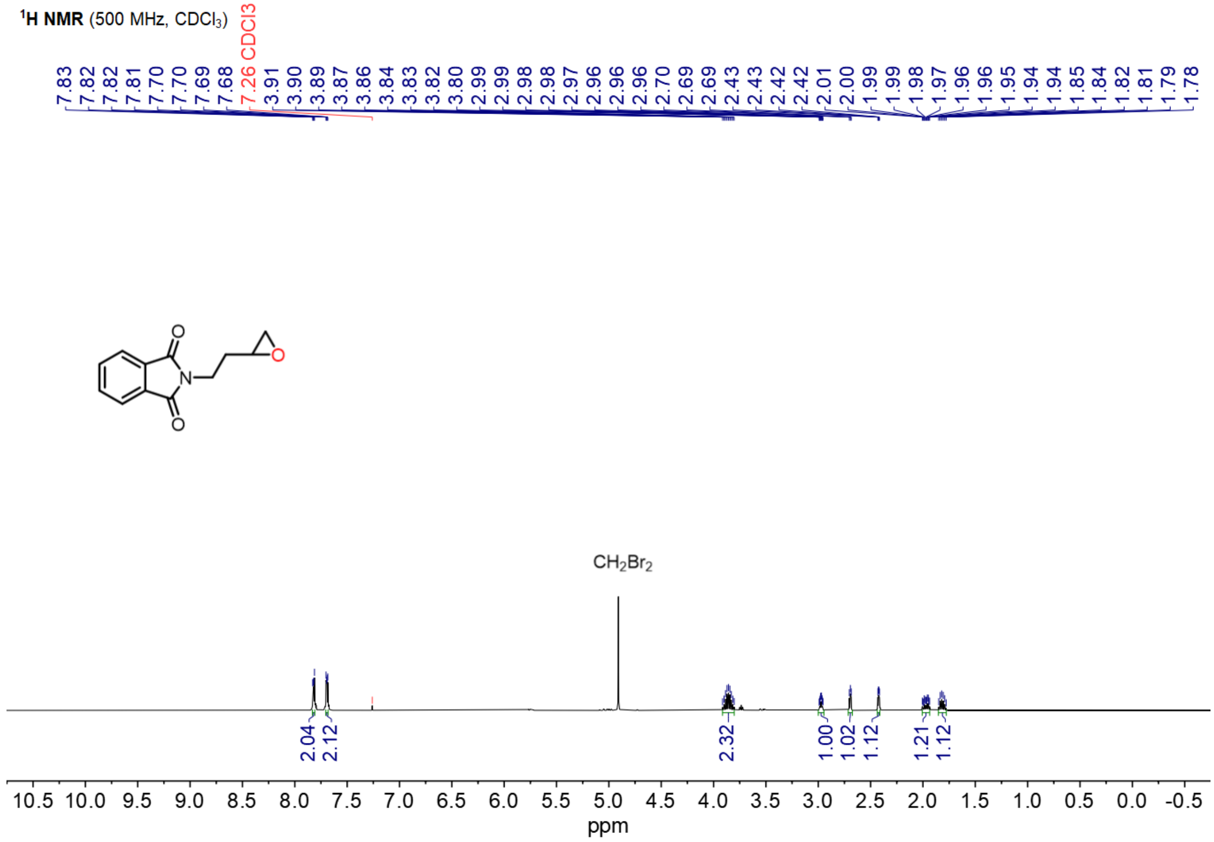

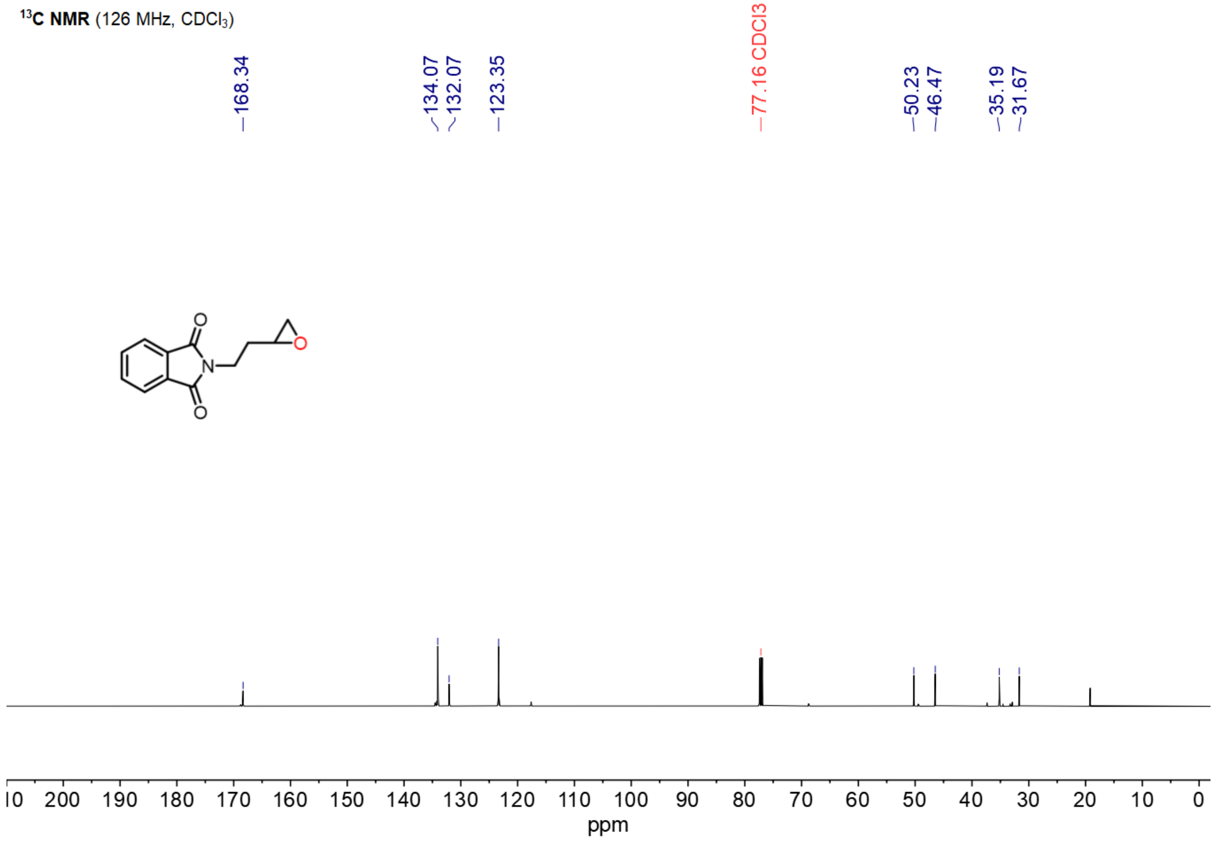

1,6-dioxaspiro[2.5]octane, **2g**:

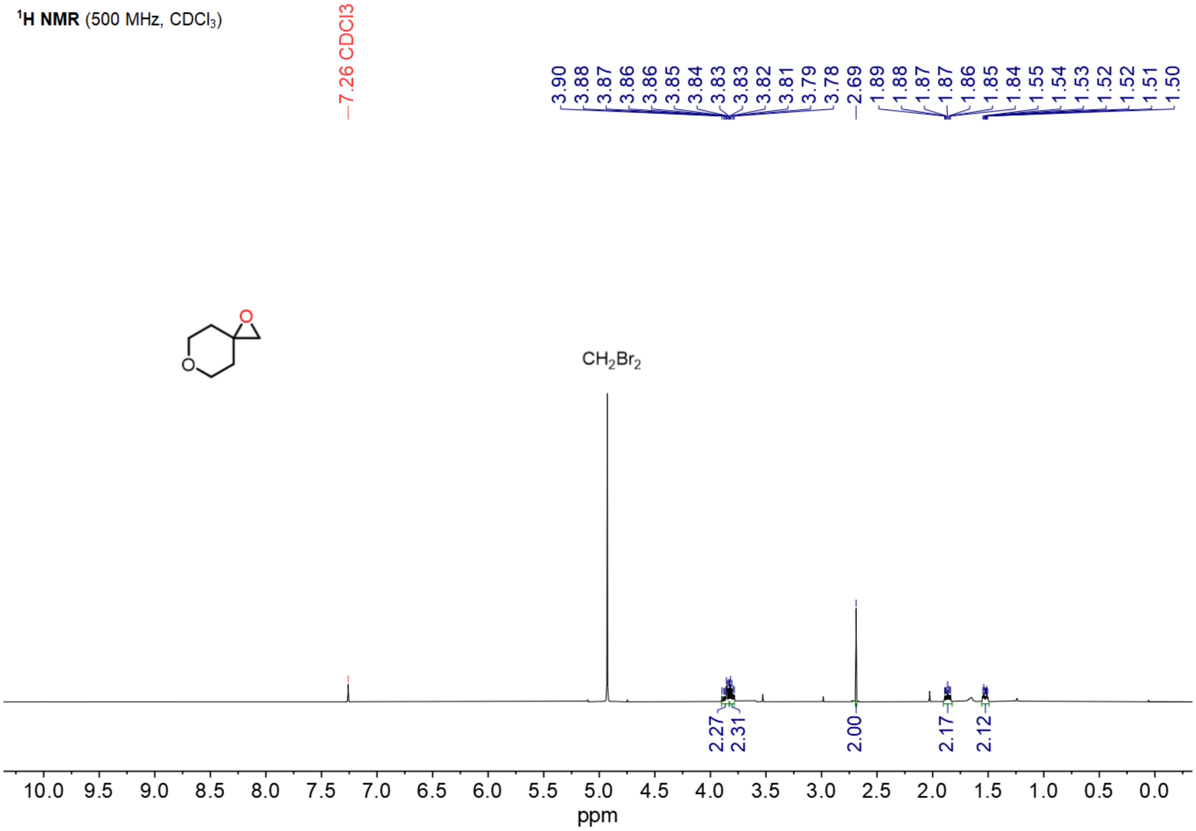

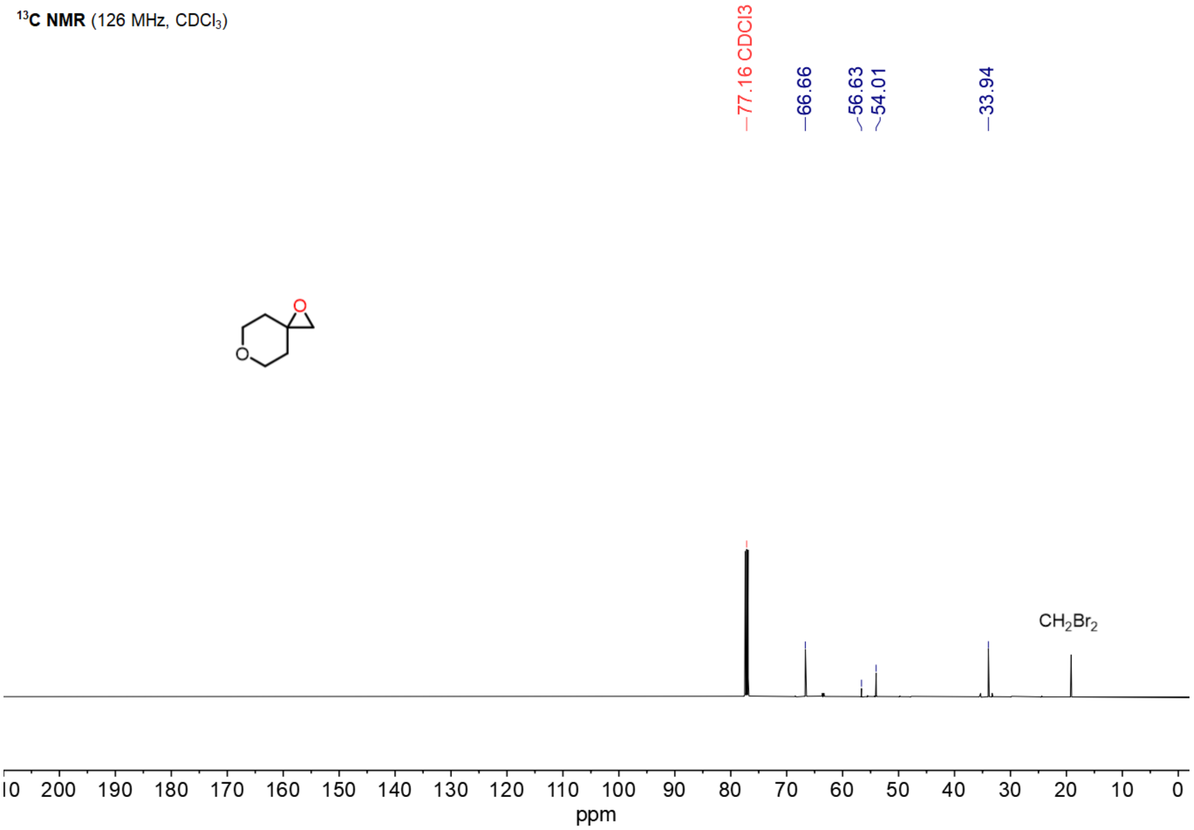

tert-butyl 1-oxa-6-azaspiro[2.5]octane-6-carboxylate, **2h**:

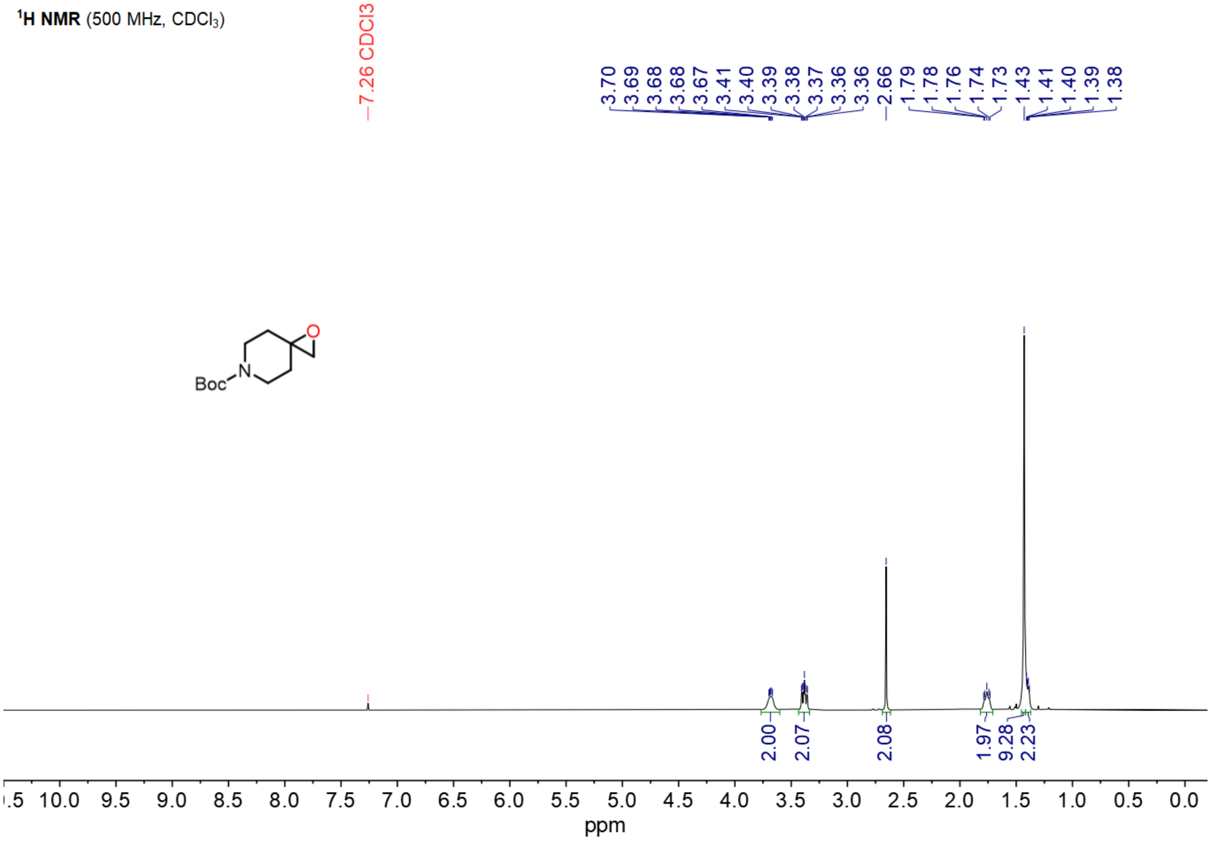

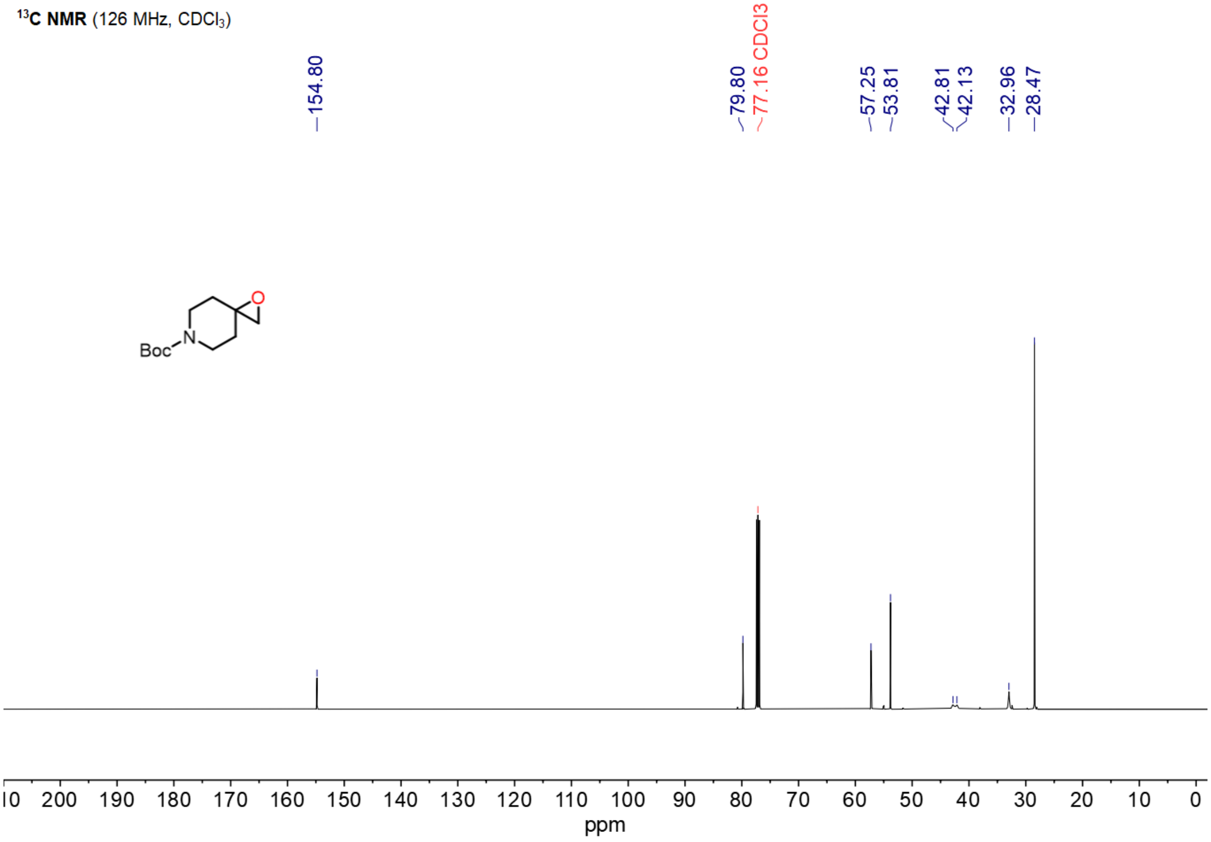

2-(oxiran-2-yl)isoindoline-1,3-dione, **2l**:

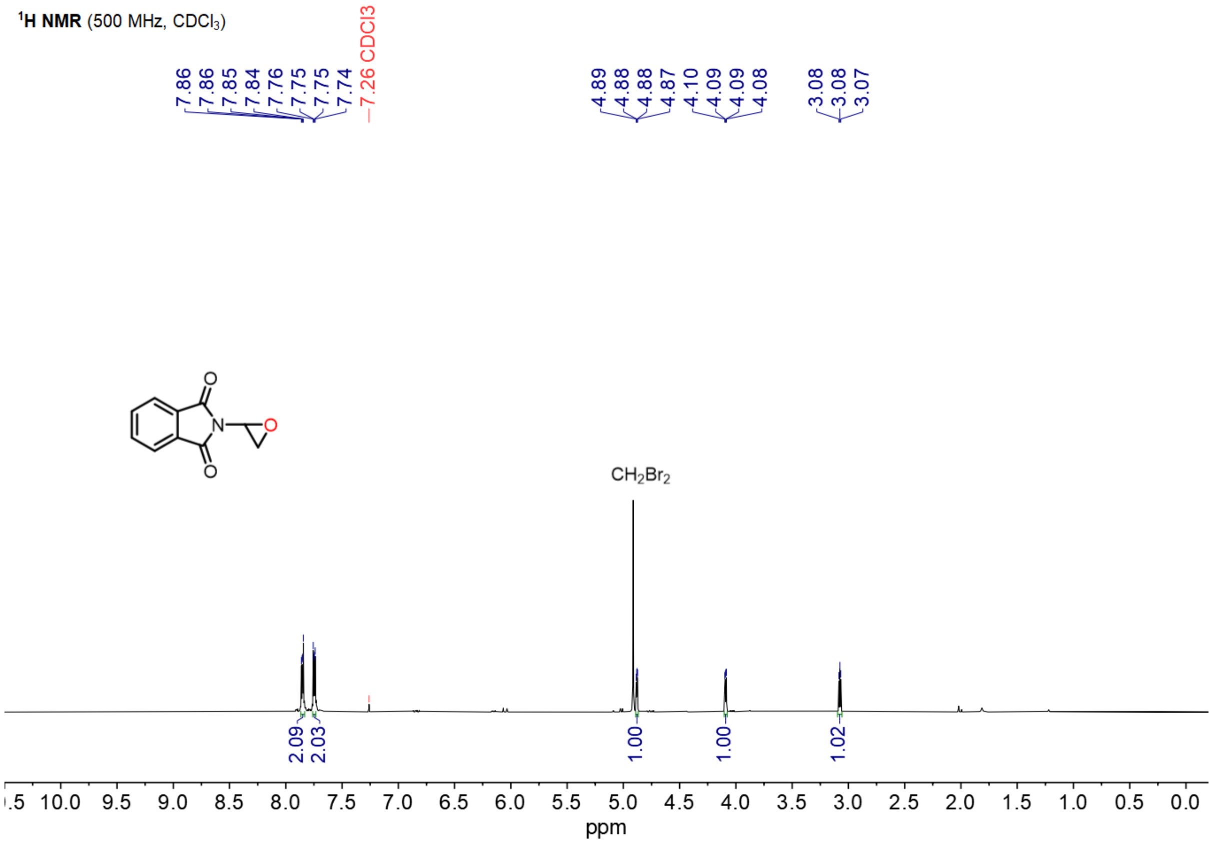

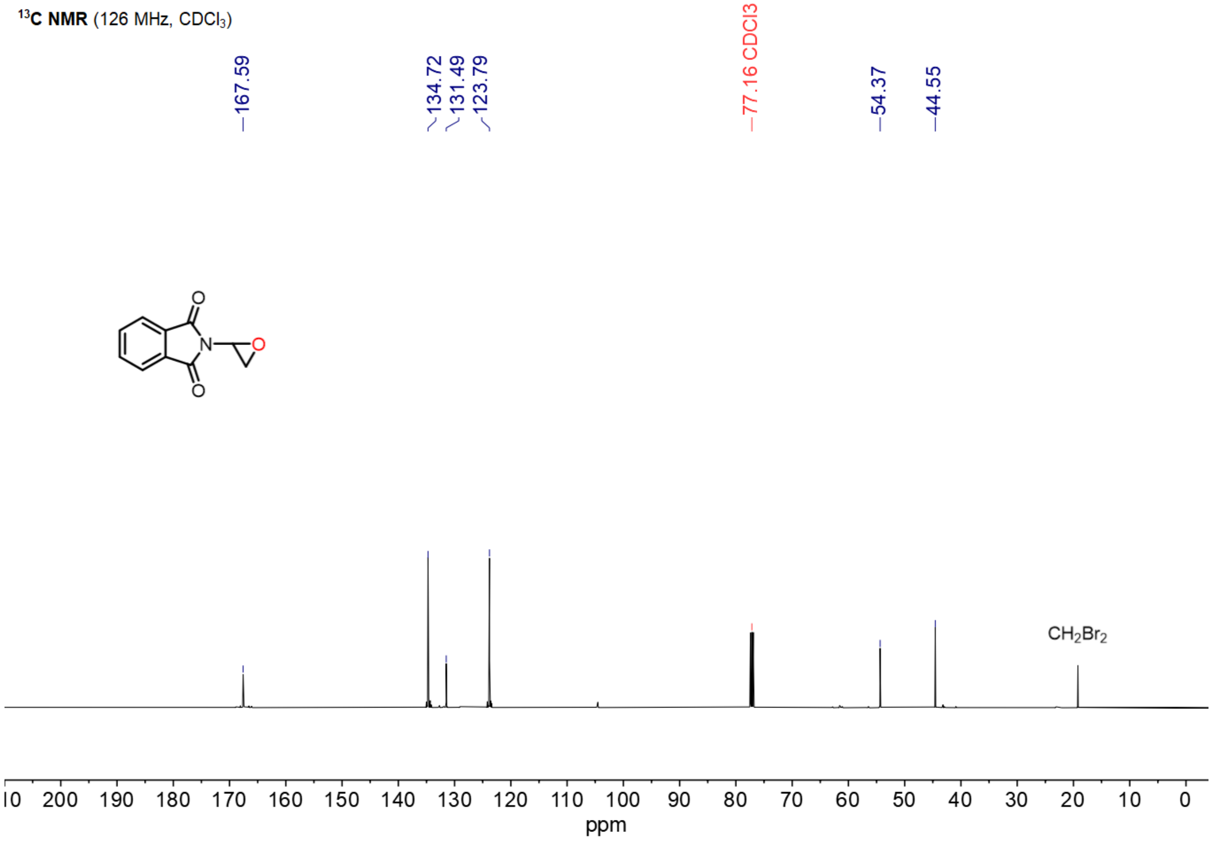

ethyl (*E*)-3-(3-methyloxiran-2-yl)acrylate, **6a**:

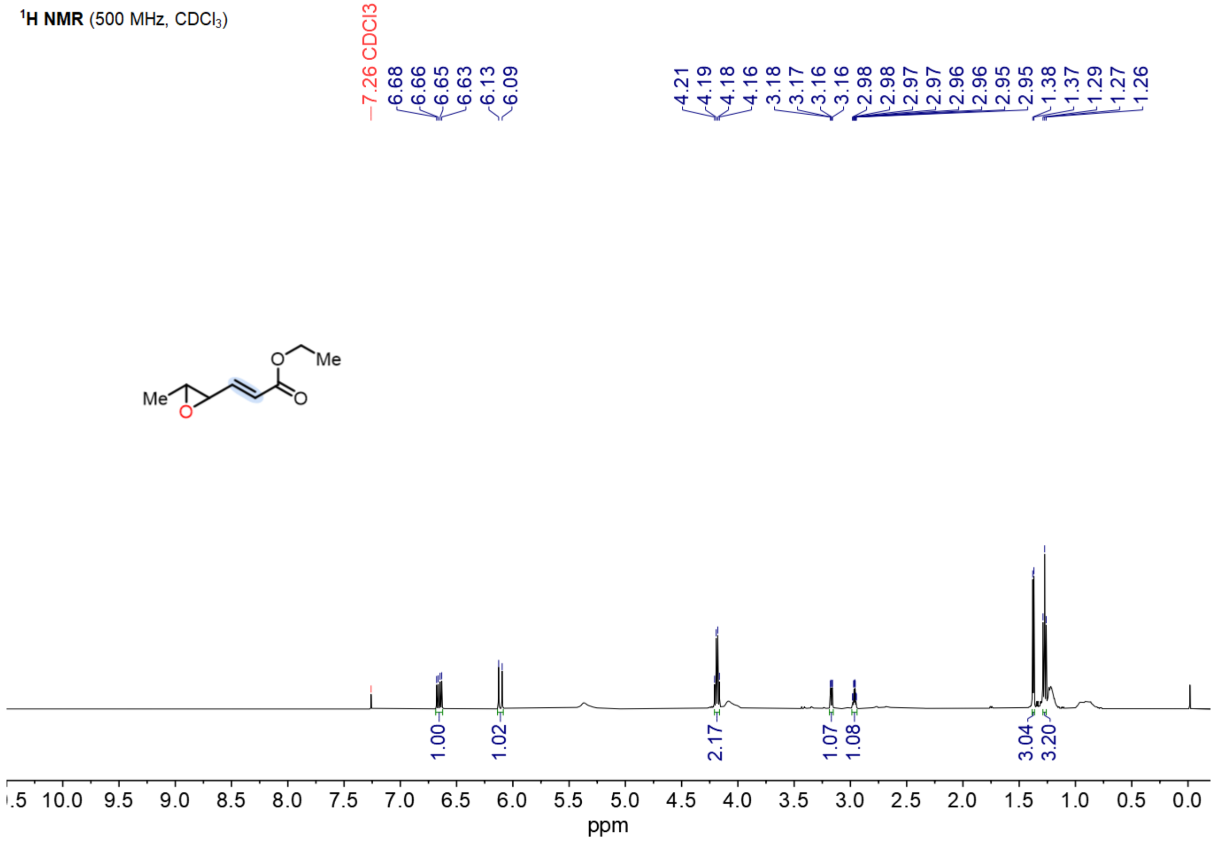

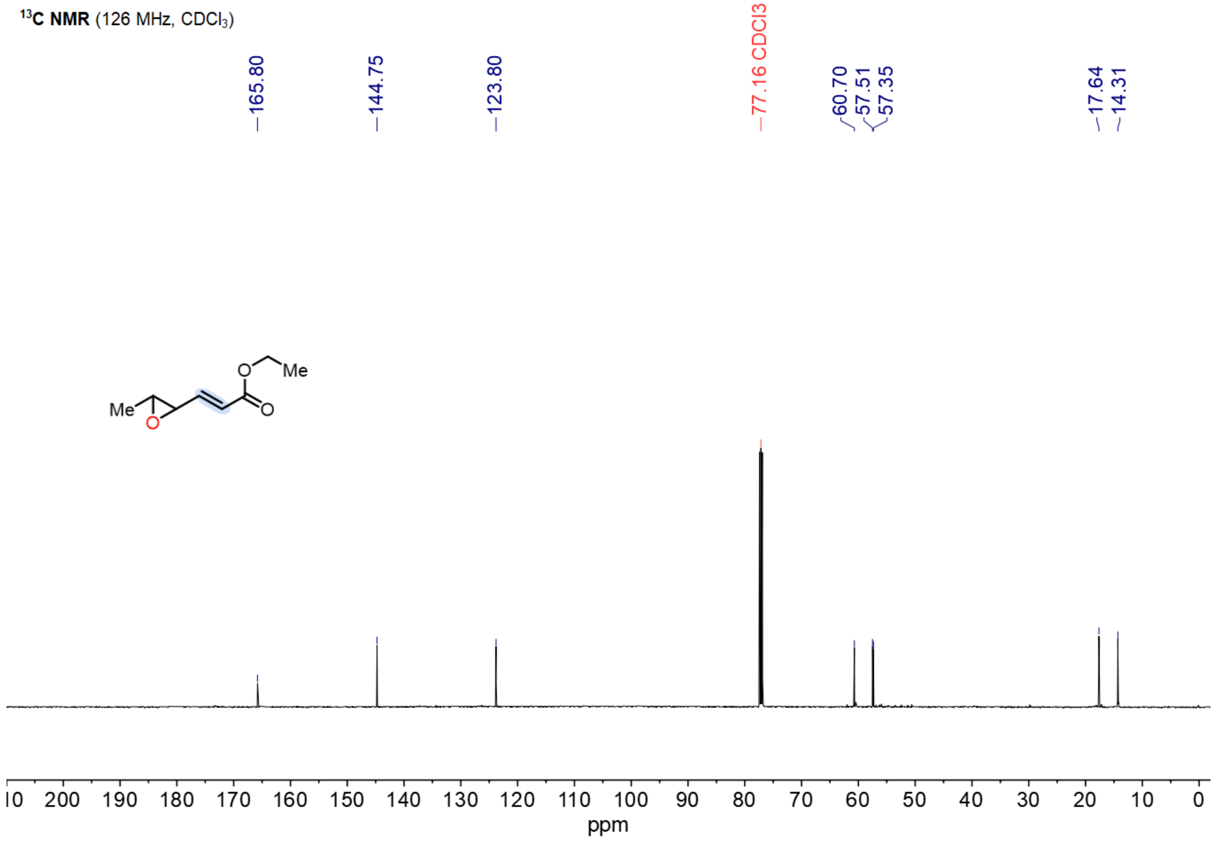

(*Z*)-9-oxabicyclo[6.1.0]non-4-ene, **6b**:

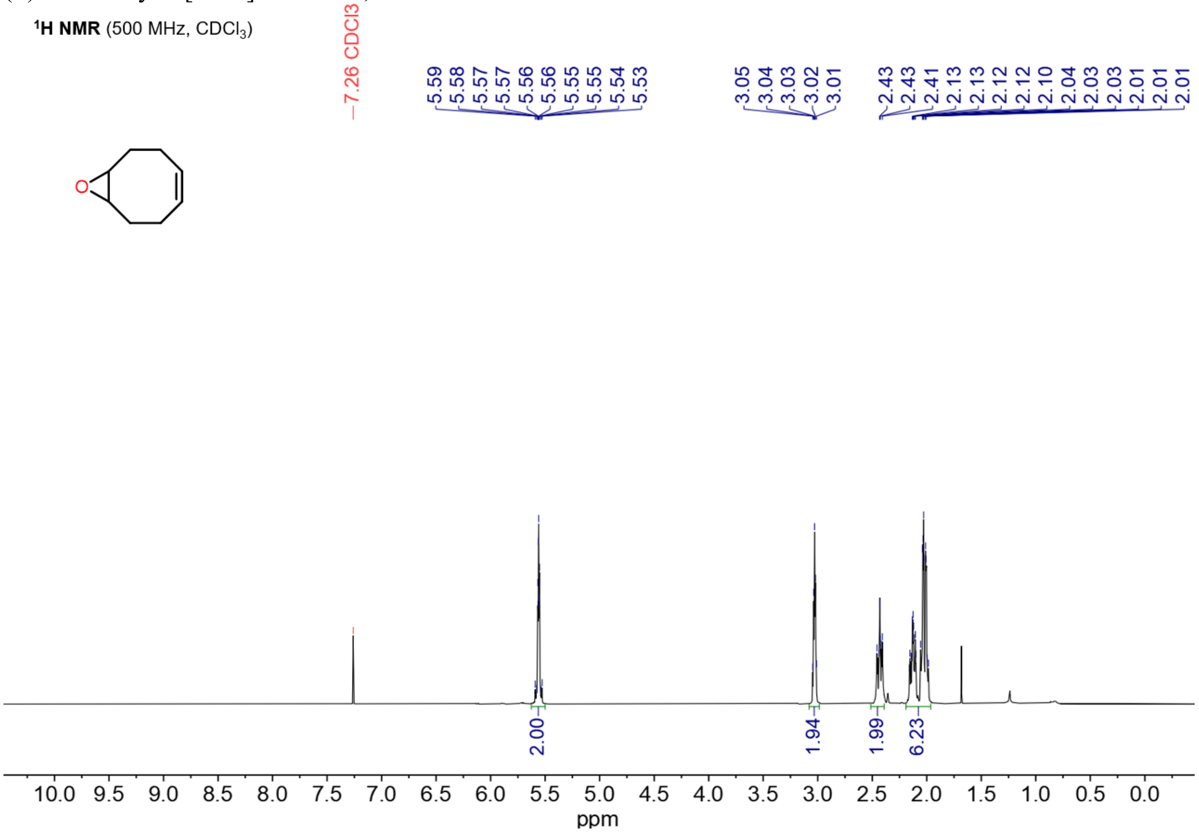

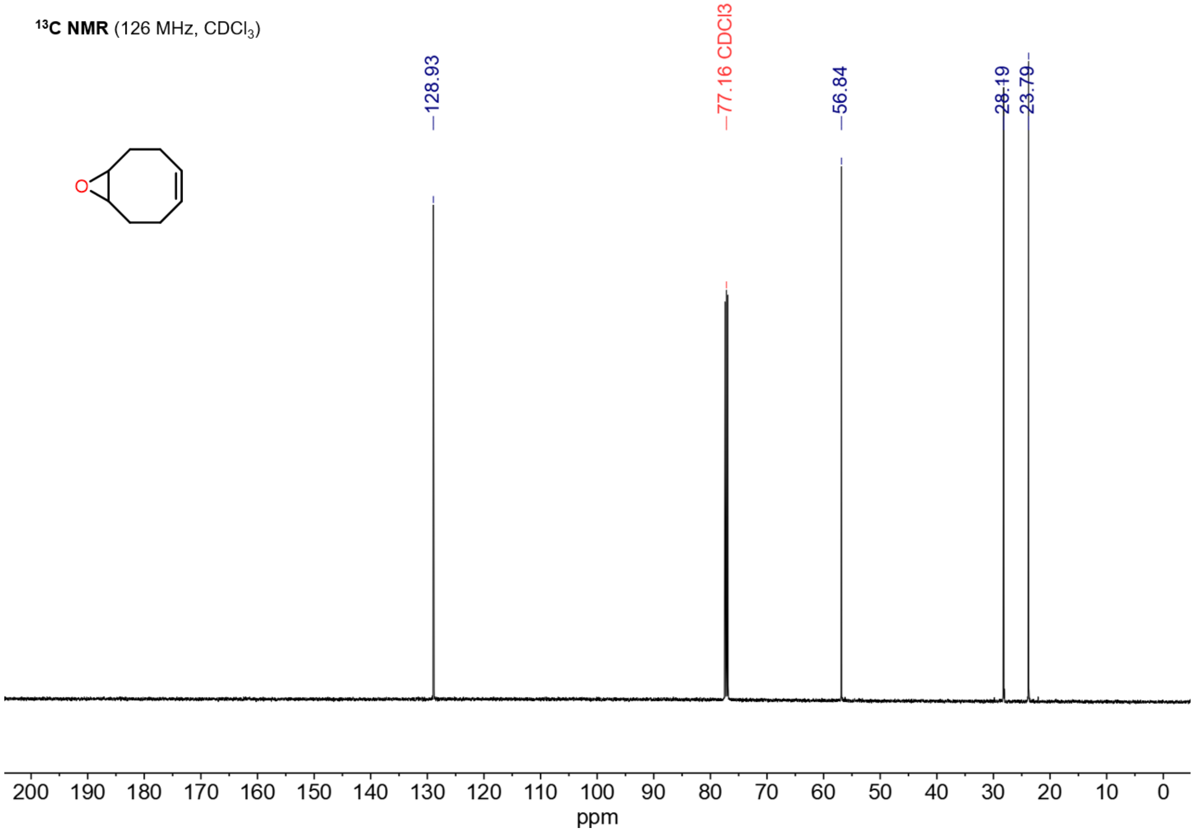

(*E*)-2-phenyl-3-styryloxirane, **6c**:

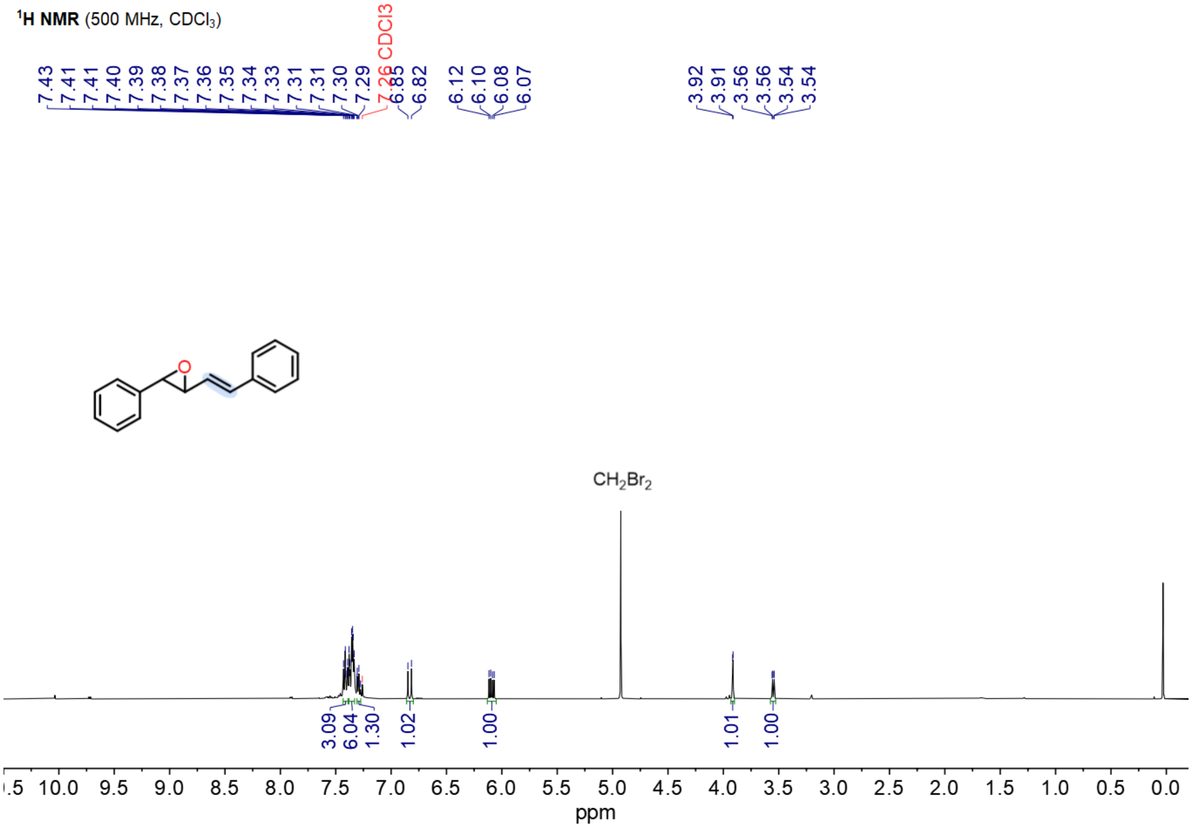

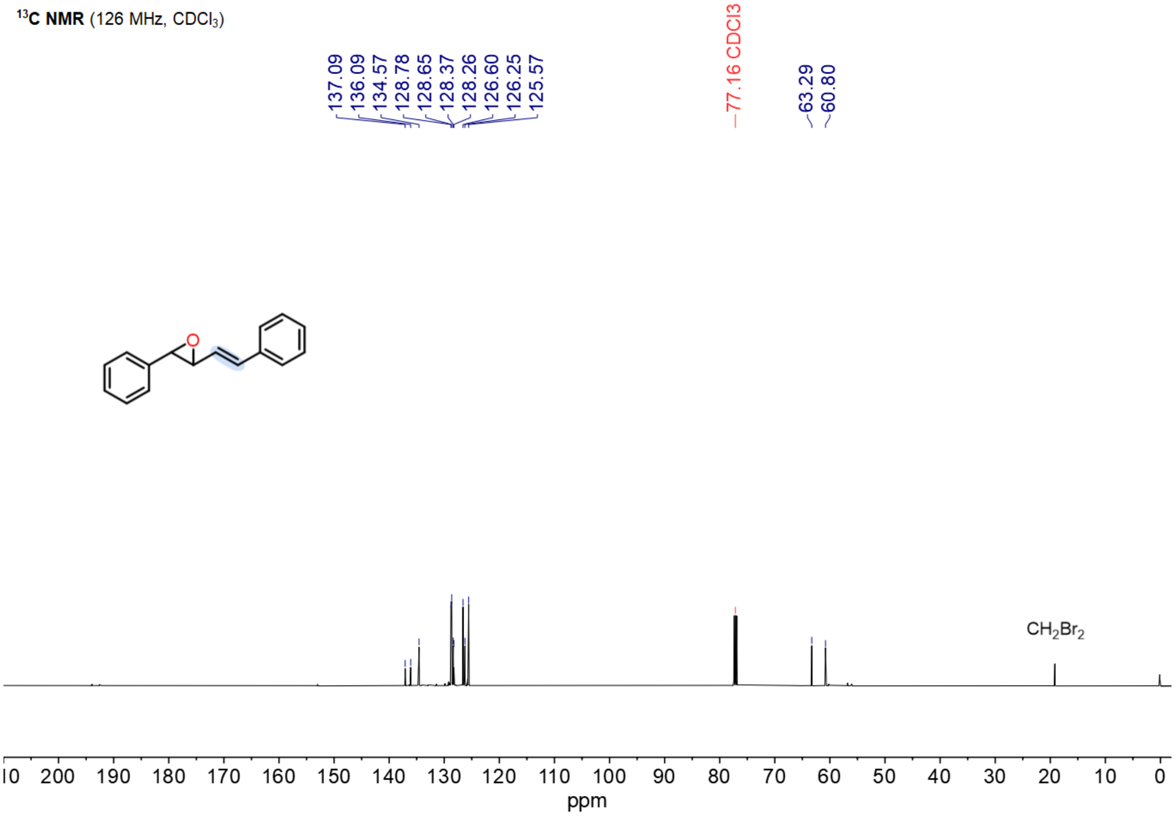

5-(3,3-dimethyloxiran-2-yl)-3-methylpent-1-en-3-yl propionate, **6d**:

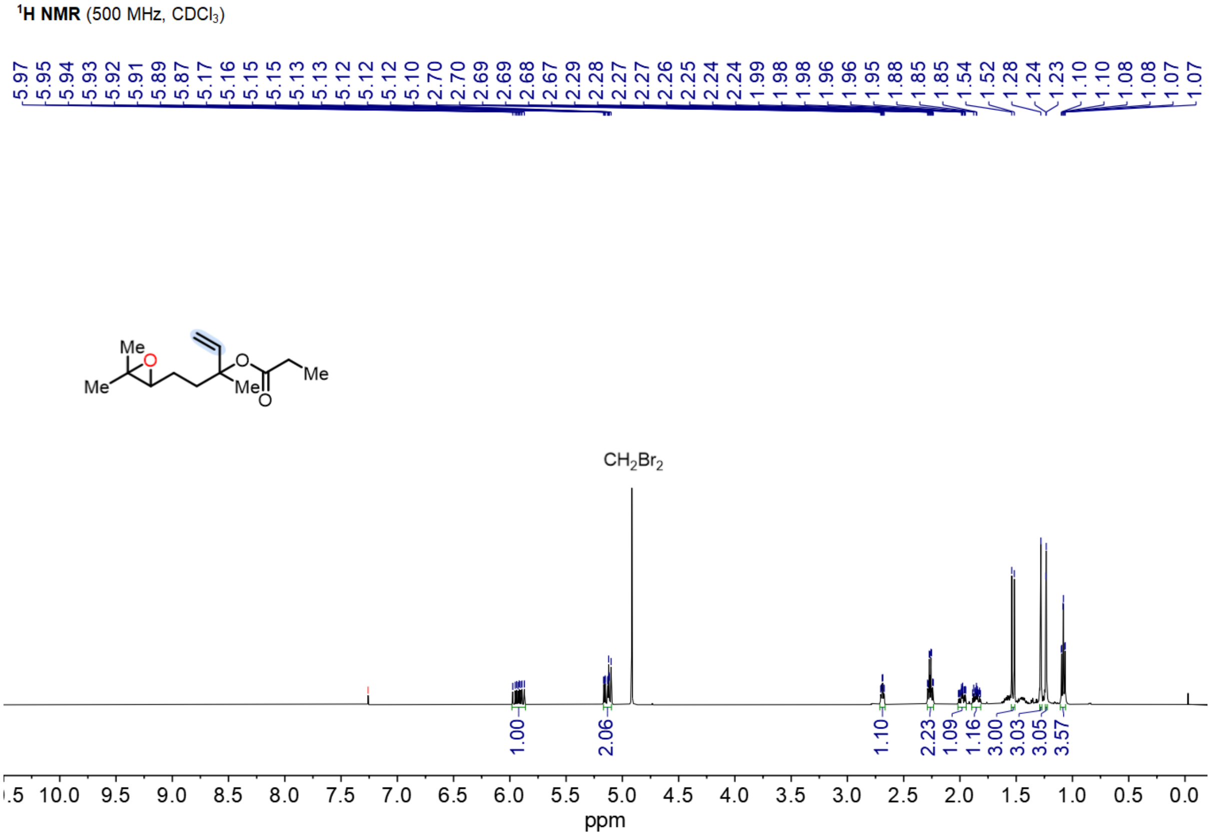

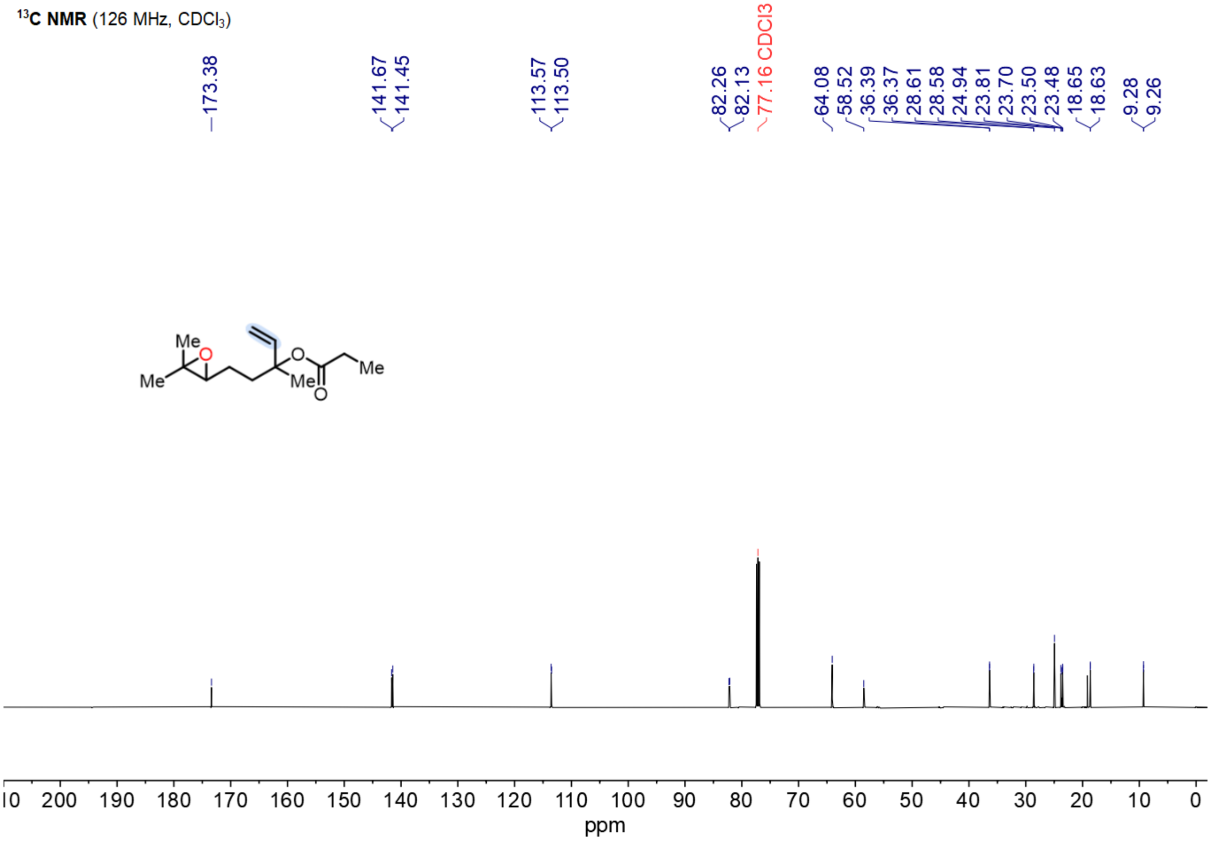

(*E*)-5-(3,3-dimethyloxiran-2-yl)-3-methylpent-2-en-1-yl propionate, **6e**:

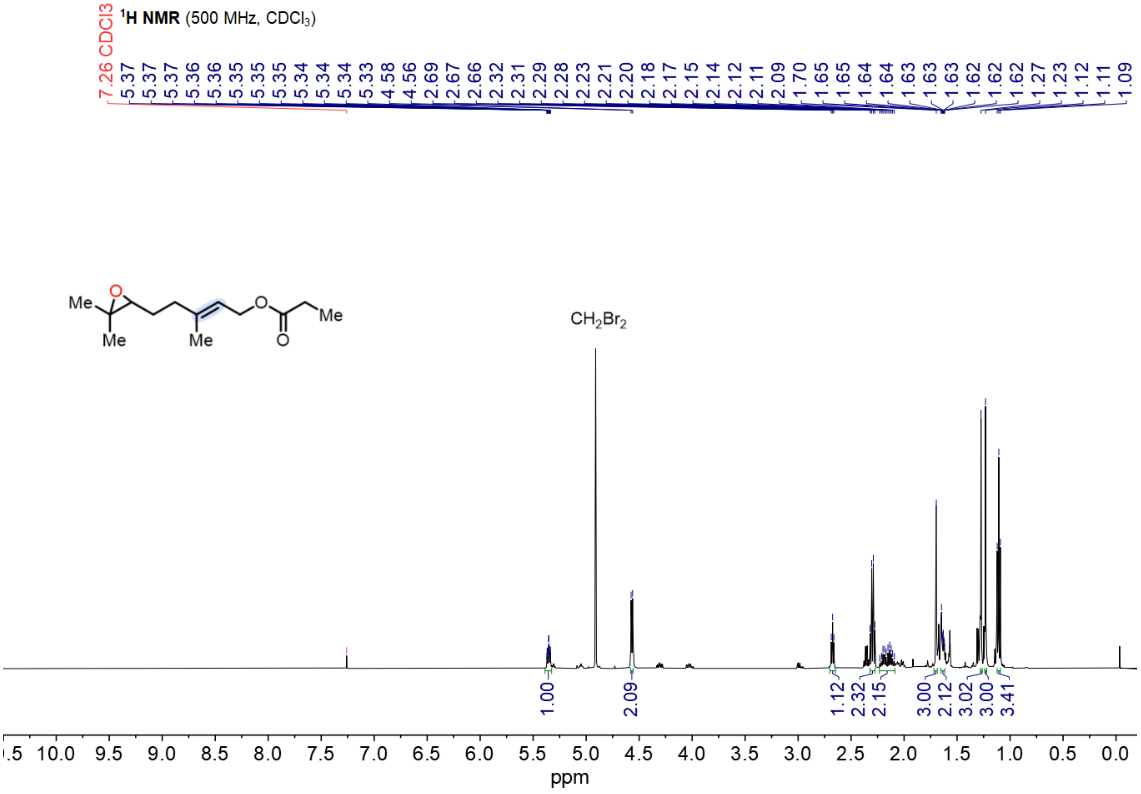

mono-:*di*-epoxide (6:1) selectivity determination:

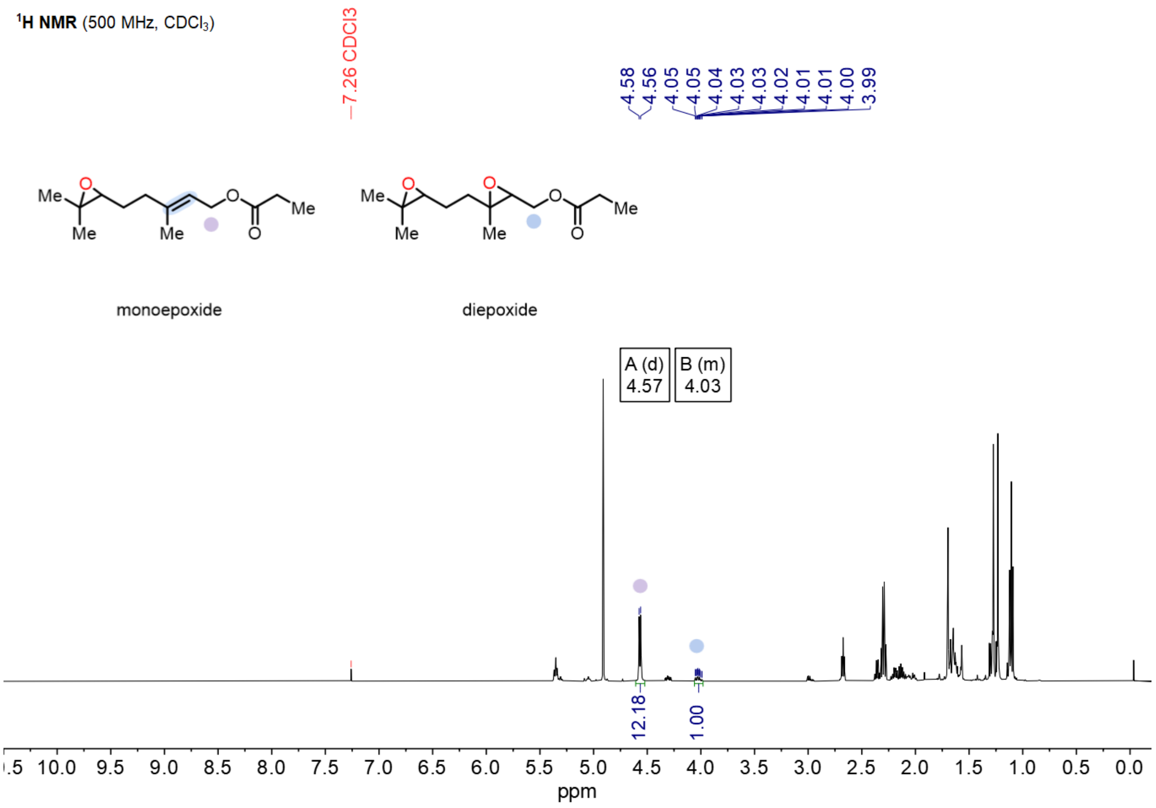

3,3'-diphenyl-2,2'-bioxirane, **6c’**:

^{1}H NMR (400 MHz, $CDCl_3$)

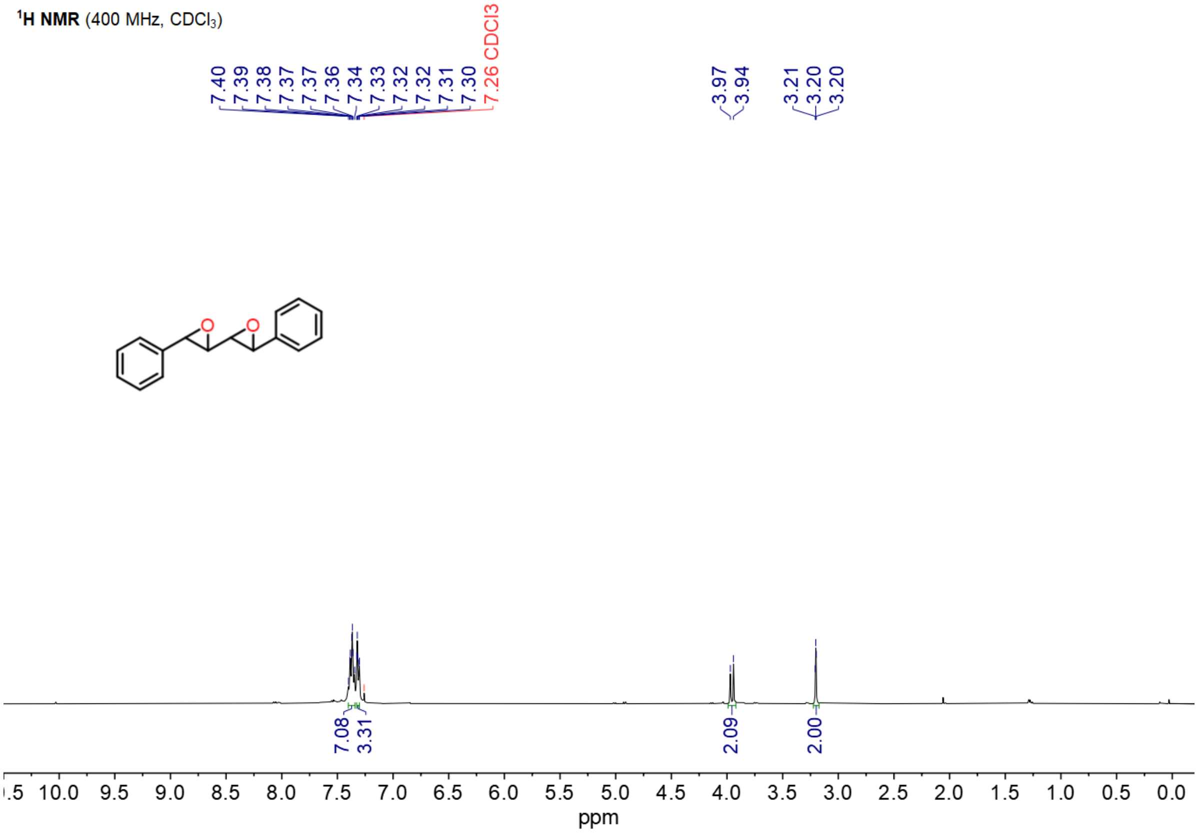

^{13}C NMR (101 MHz, $CDCl_3$)

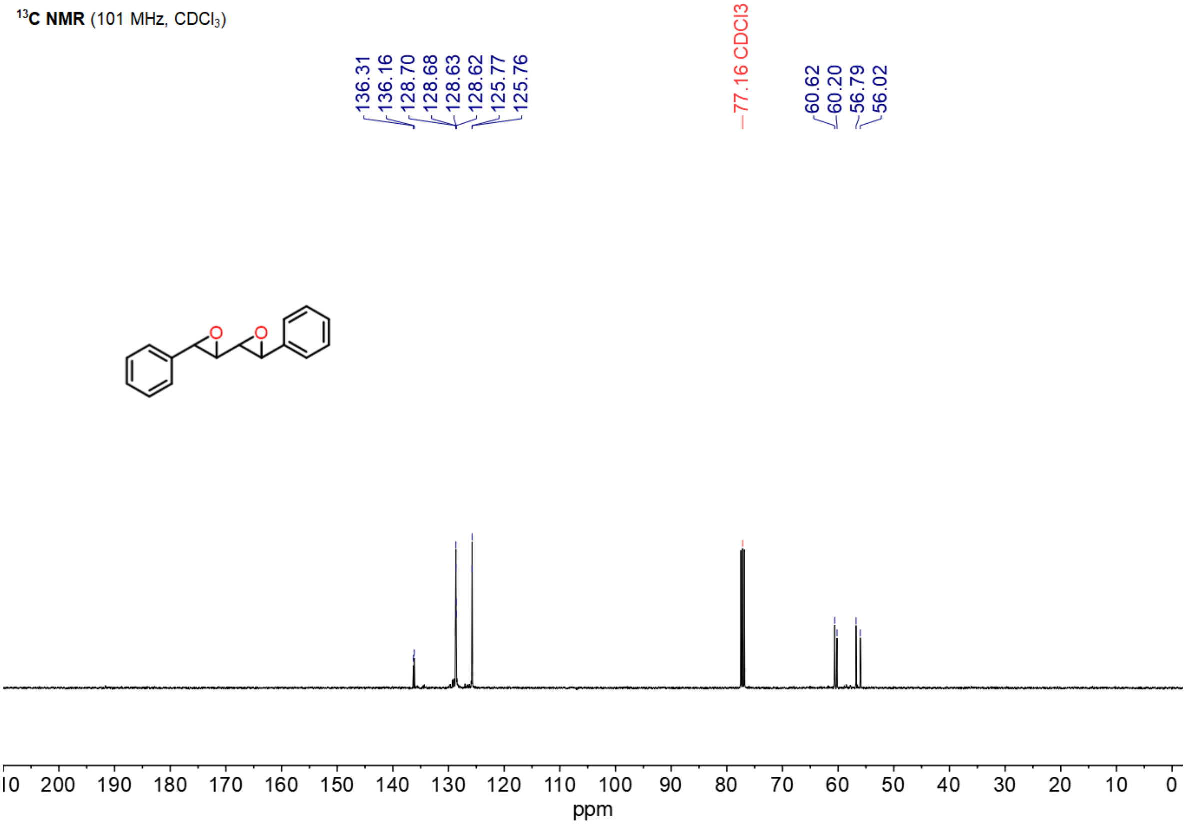

propane-1,2,3-triyl tris(8-(3-octyloxiran-2-yl)octanoate), **6f**:

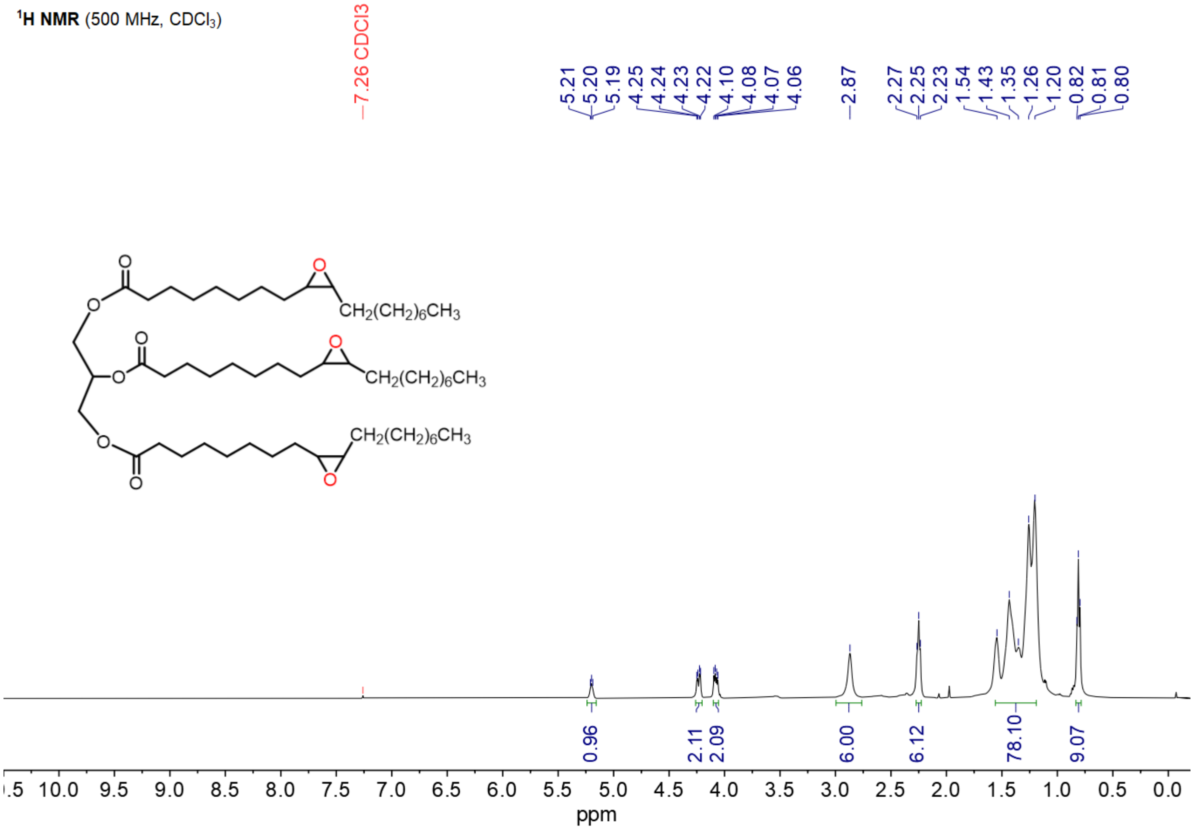

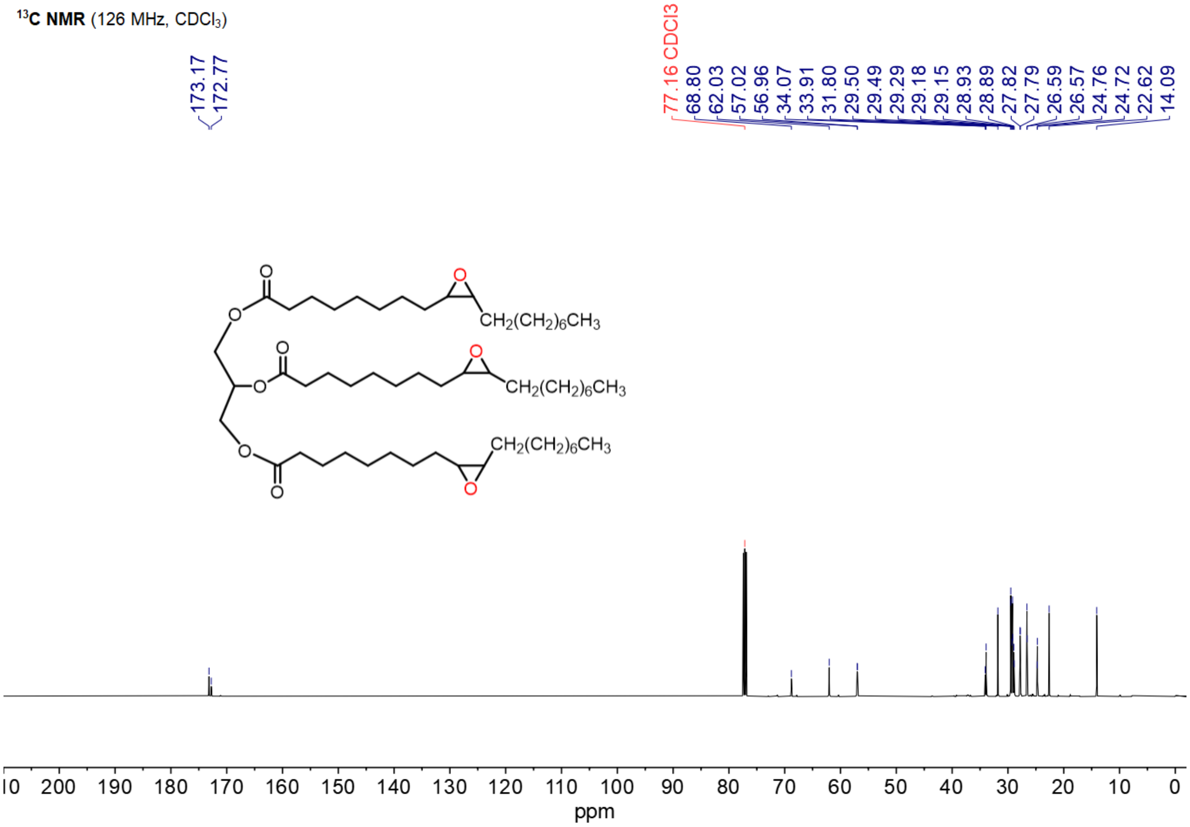

(3-(2-(3,3-dimethyloxiran-2-yl)ethyl)-3-methyloxiran-2-yl)methyl propionate, **6e’**:

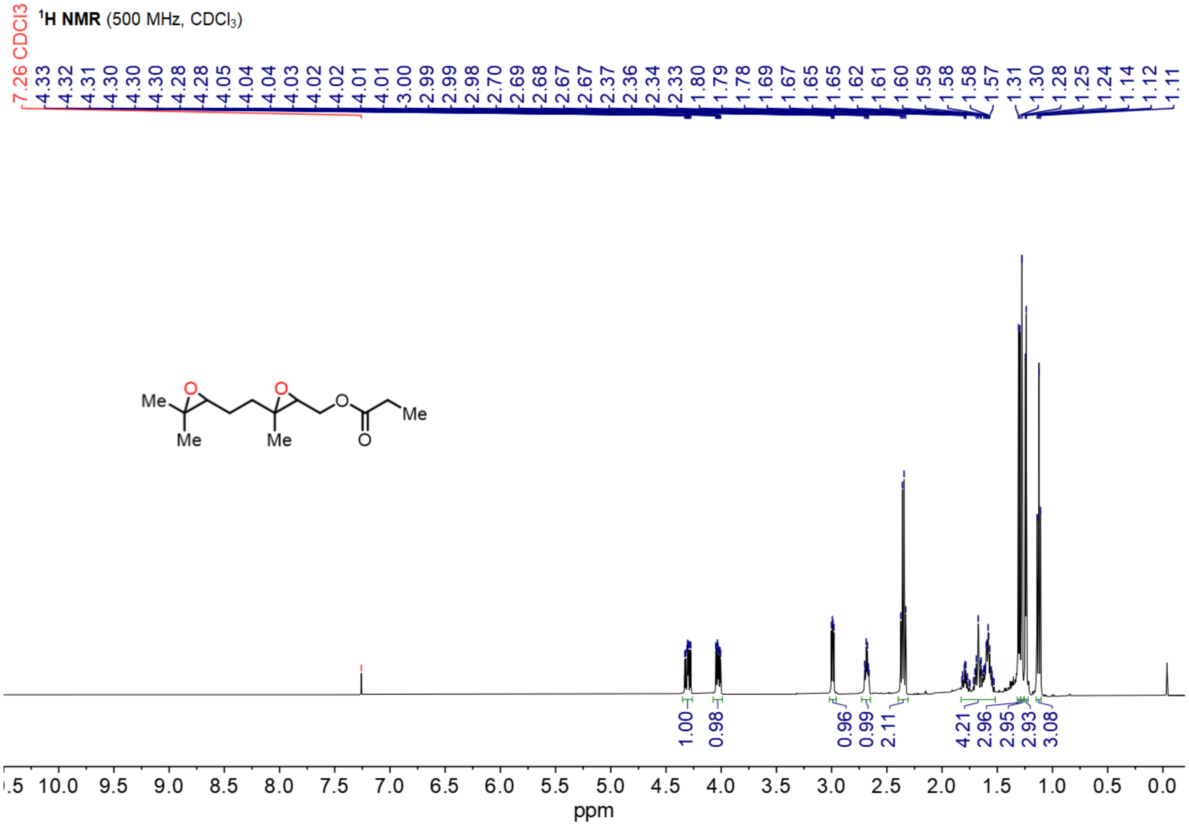

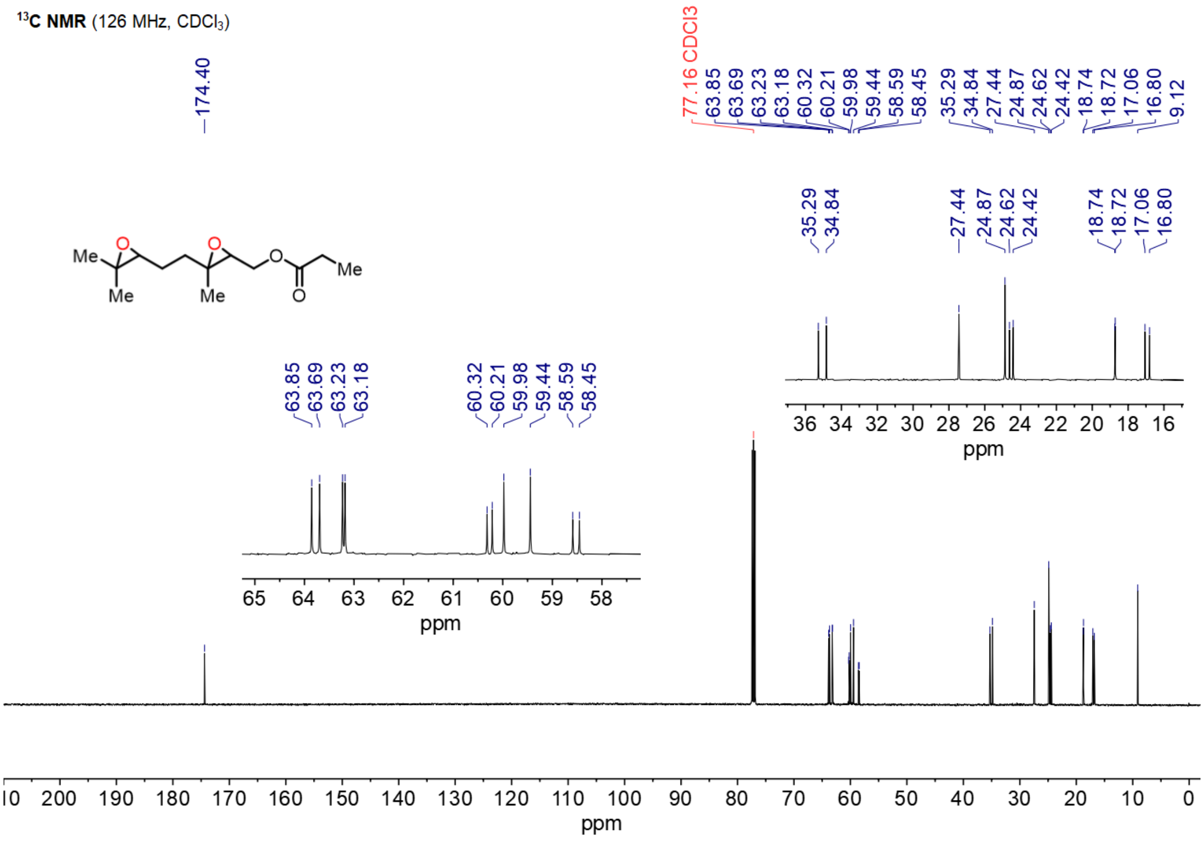

1,2-bis(3-(2-(3-(2-(3,3-dimethyloxiran-2-yl)ethyl)-3-methyloxiran-2-yl)ethyl)-3 methyloxiran-2-yl)ethane, **6g**:

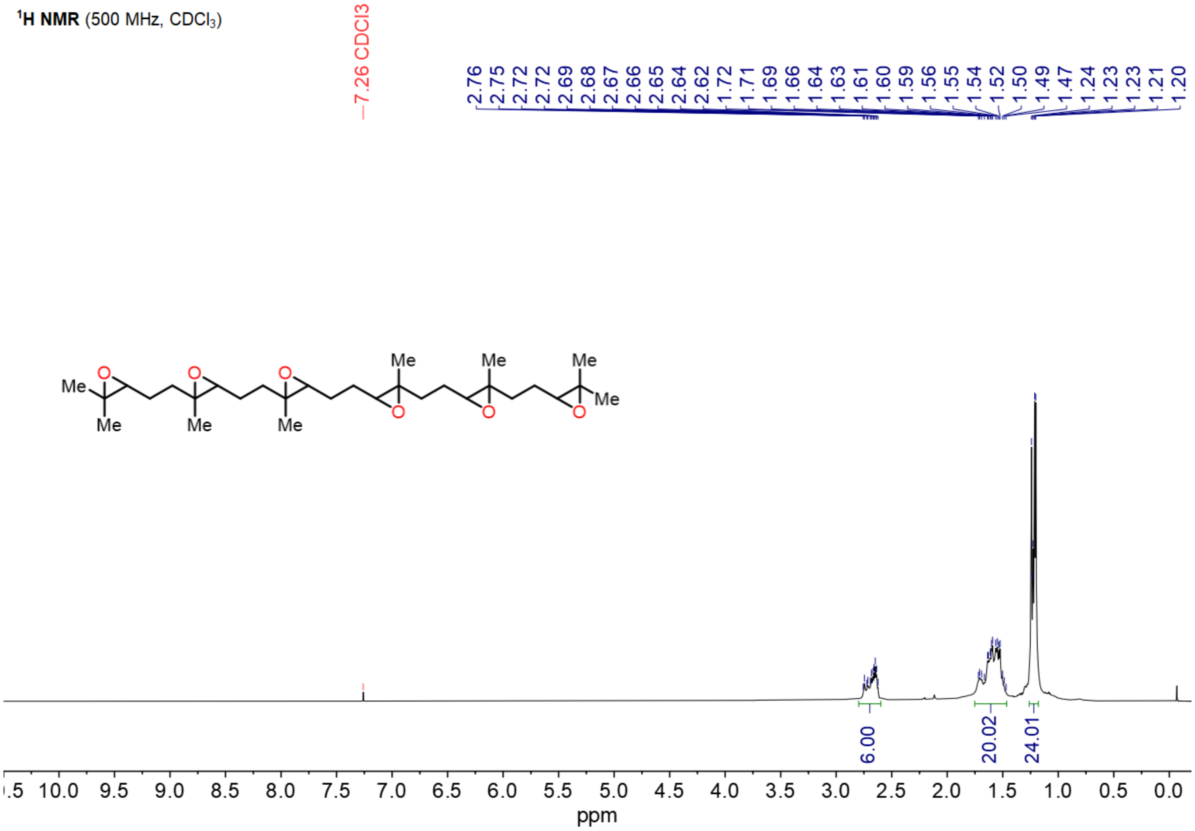

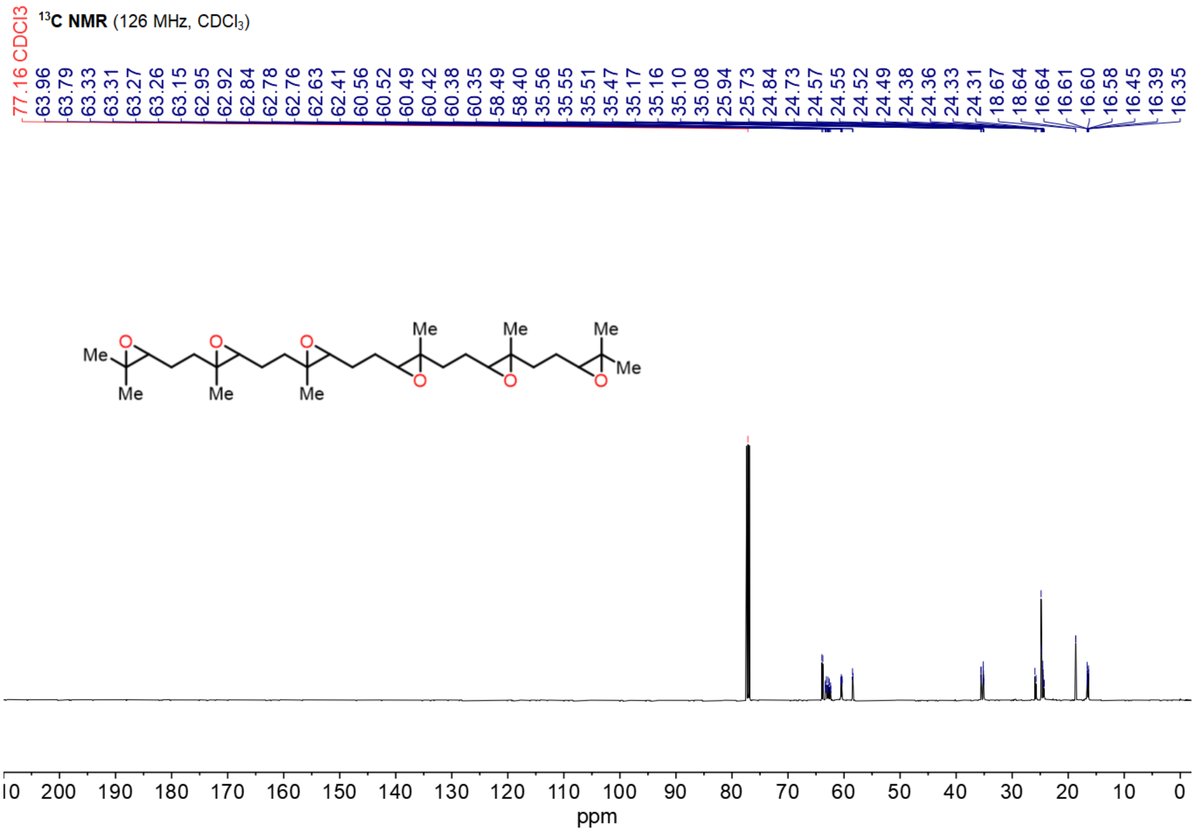

1,2-Anhydro-3,4,6-tri-*O*-acetyl-*a*-D-glucopyranose, **2i**:

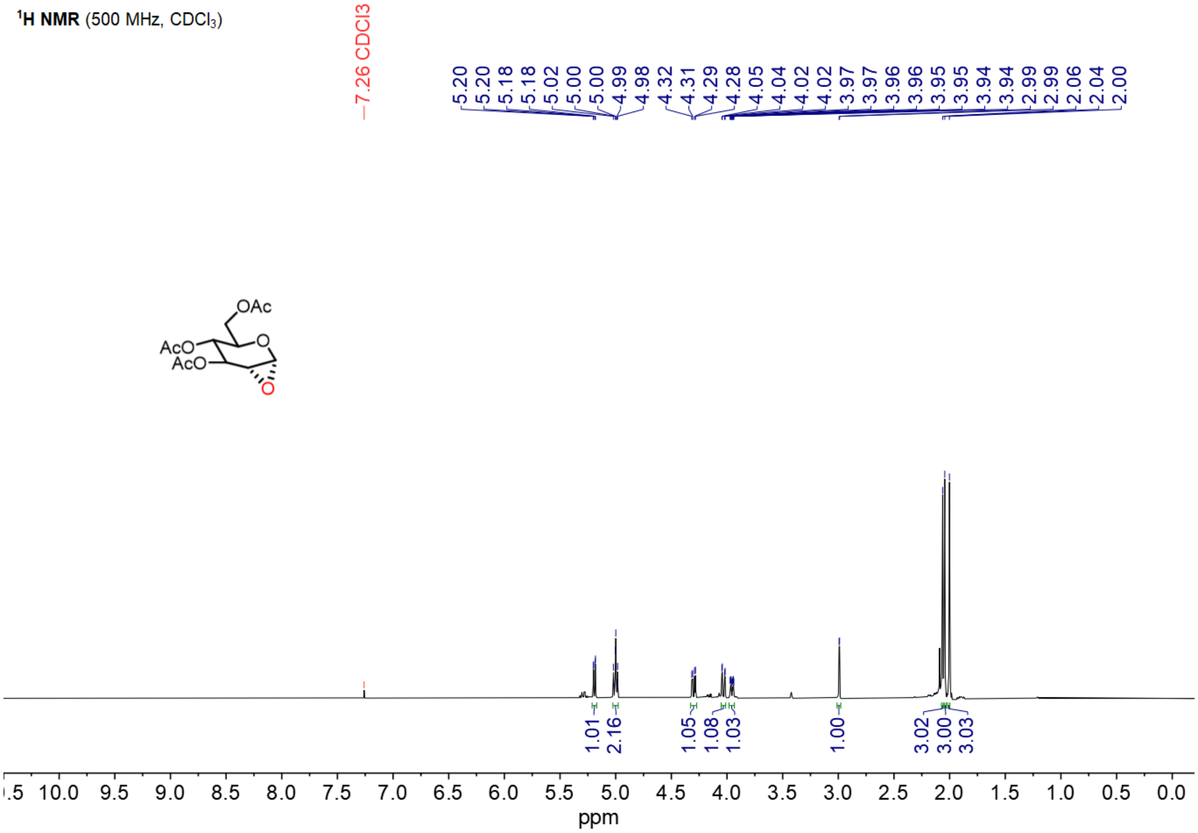

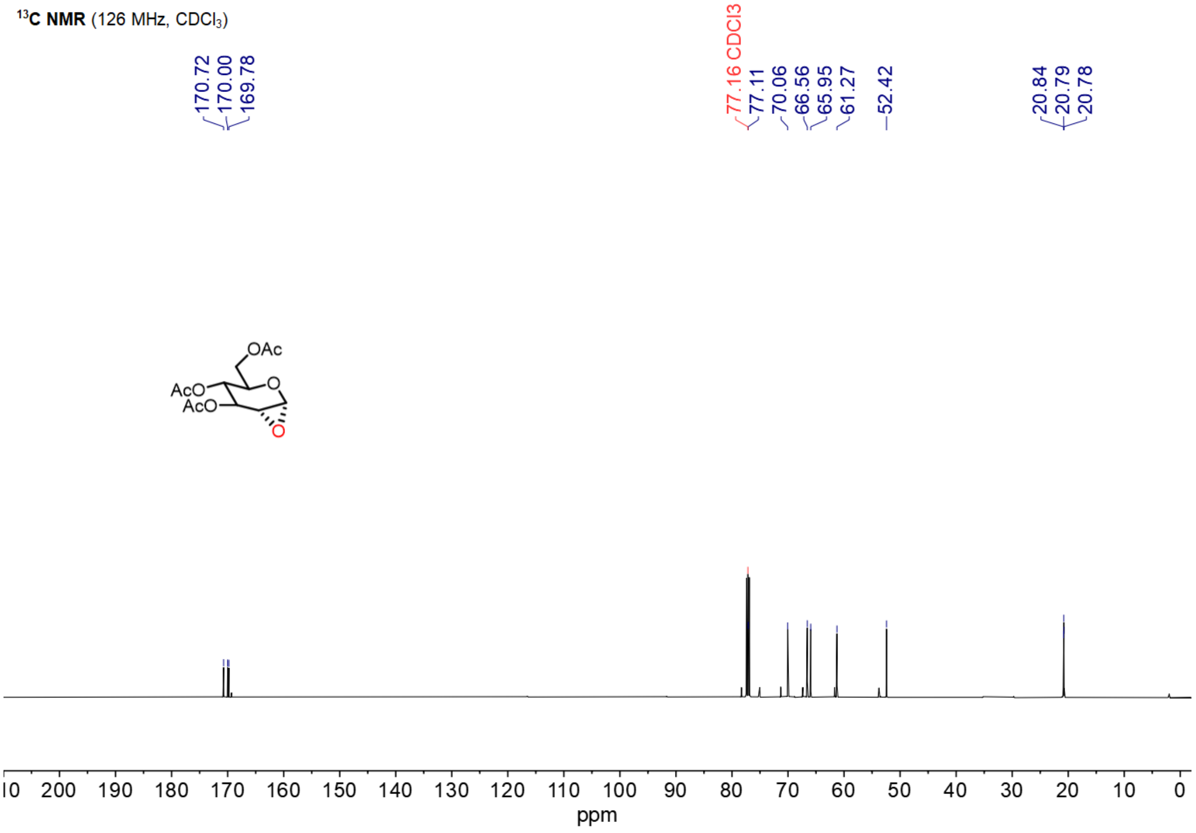

1,2-Anhydro-3,4,6-tri-*O*-acetyl-*a*-D-galactopyranose, **6h:**

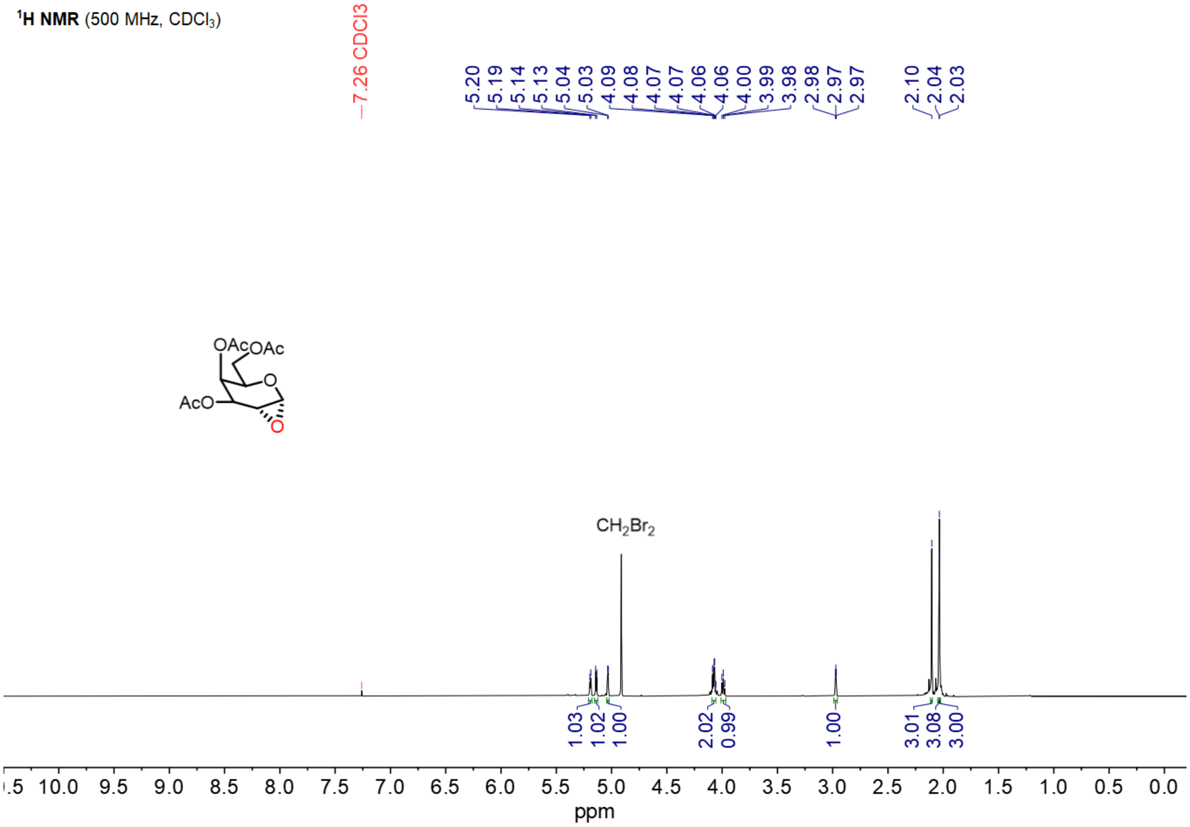

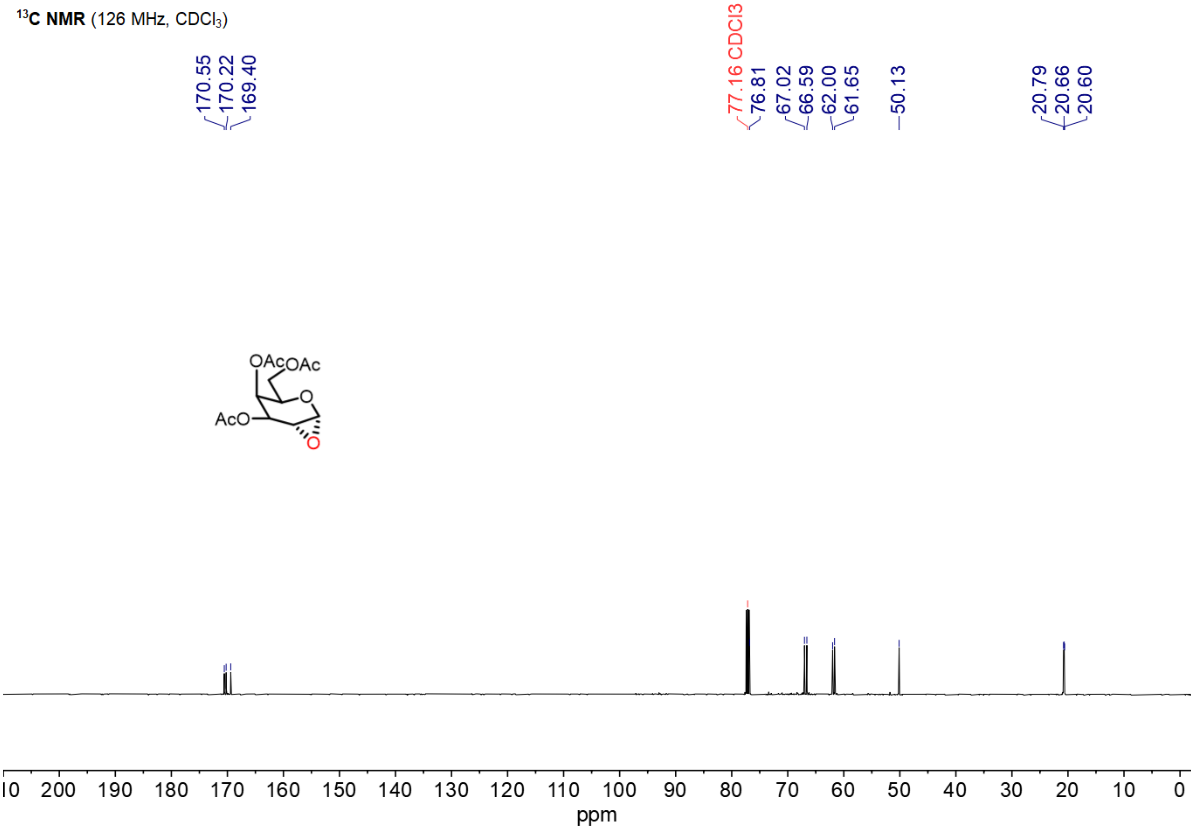

1,2-Anhydro-3,4,6-tri-*O*-benzoyl-*a*-D-galactopyranose, **6i**:

^{1}H NMR (500 MHz, $CDCl_3$)

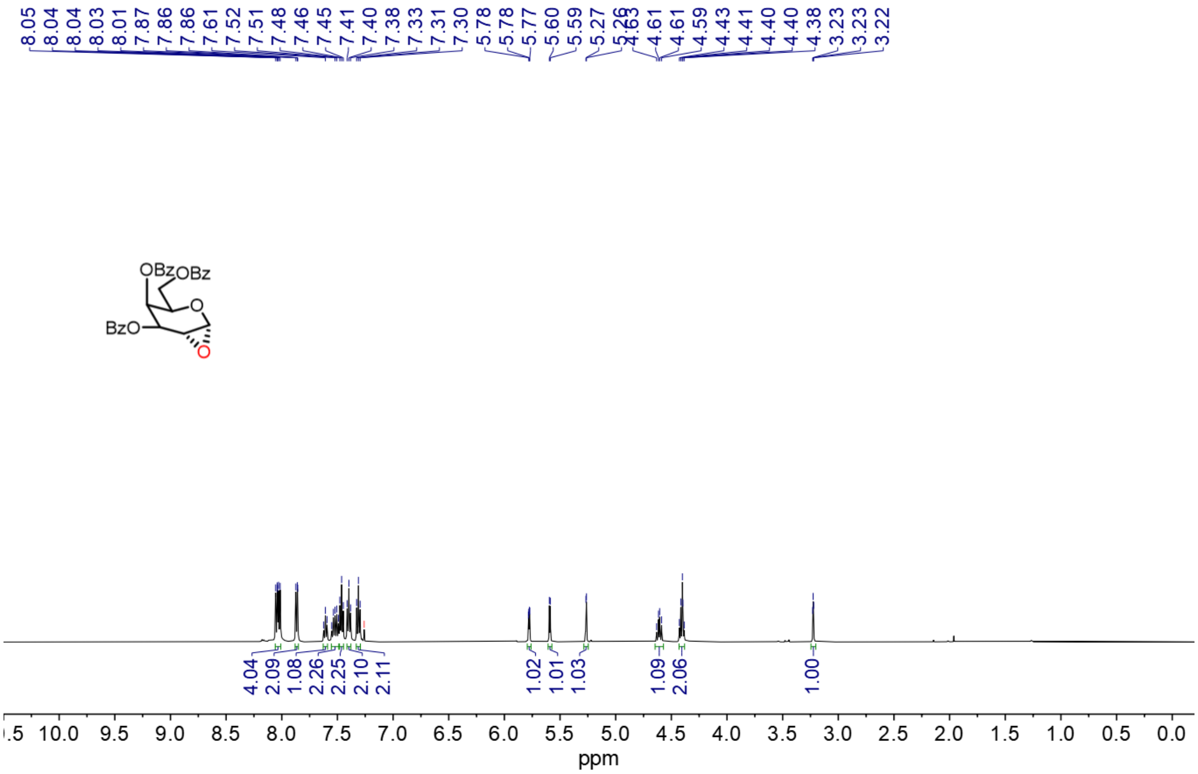

^{13}C NMR (126 MHz, $CDCl_3$)

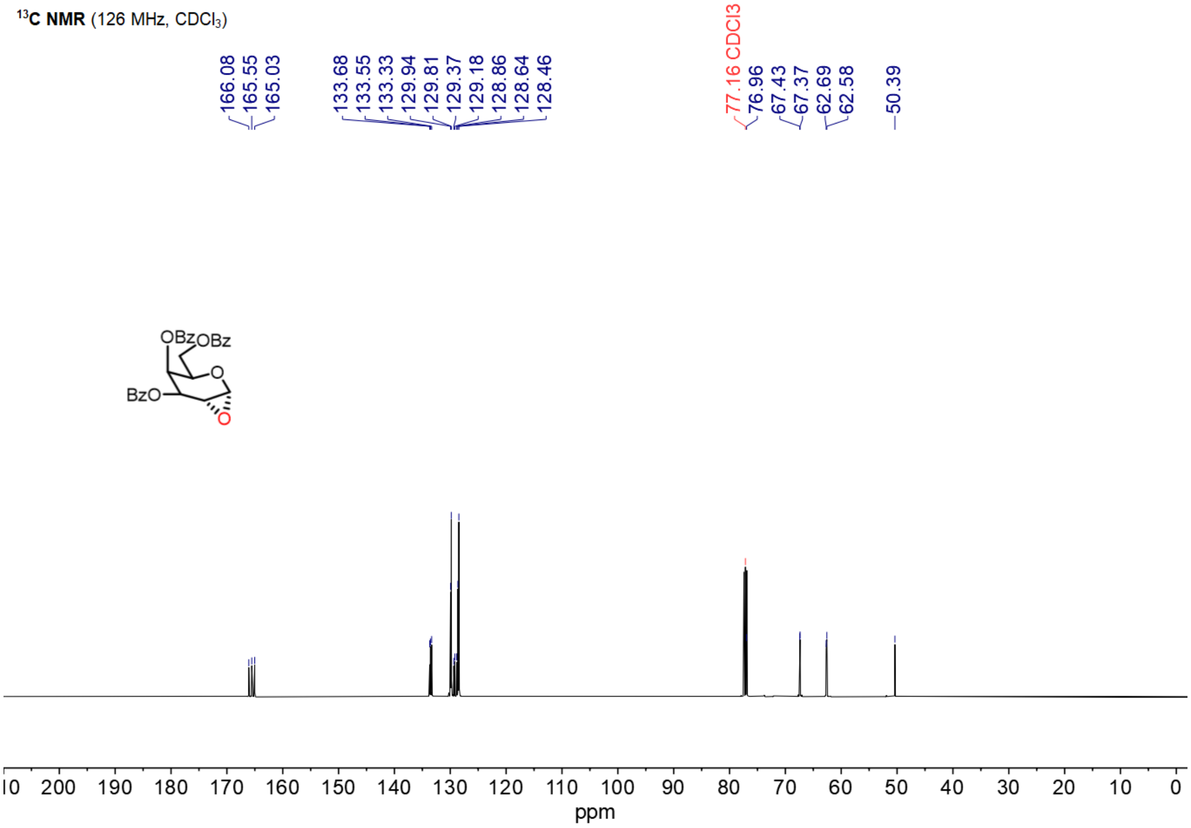

Methyl 2-hydroxy-3,4,6-tri-O-acetyl-β-D-glucopyranoside:

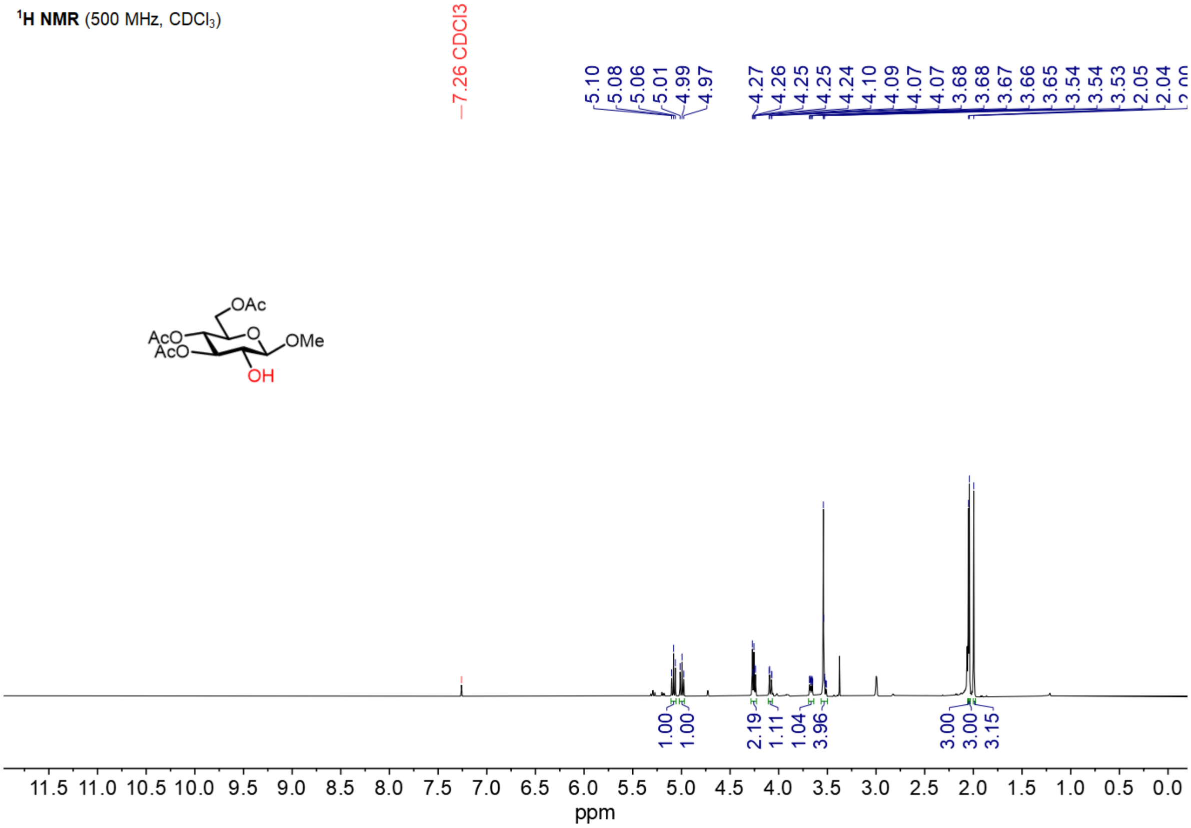

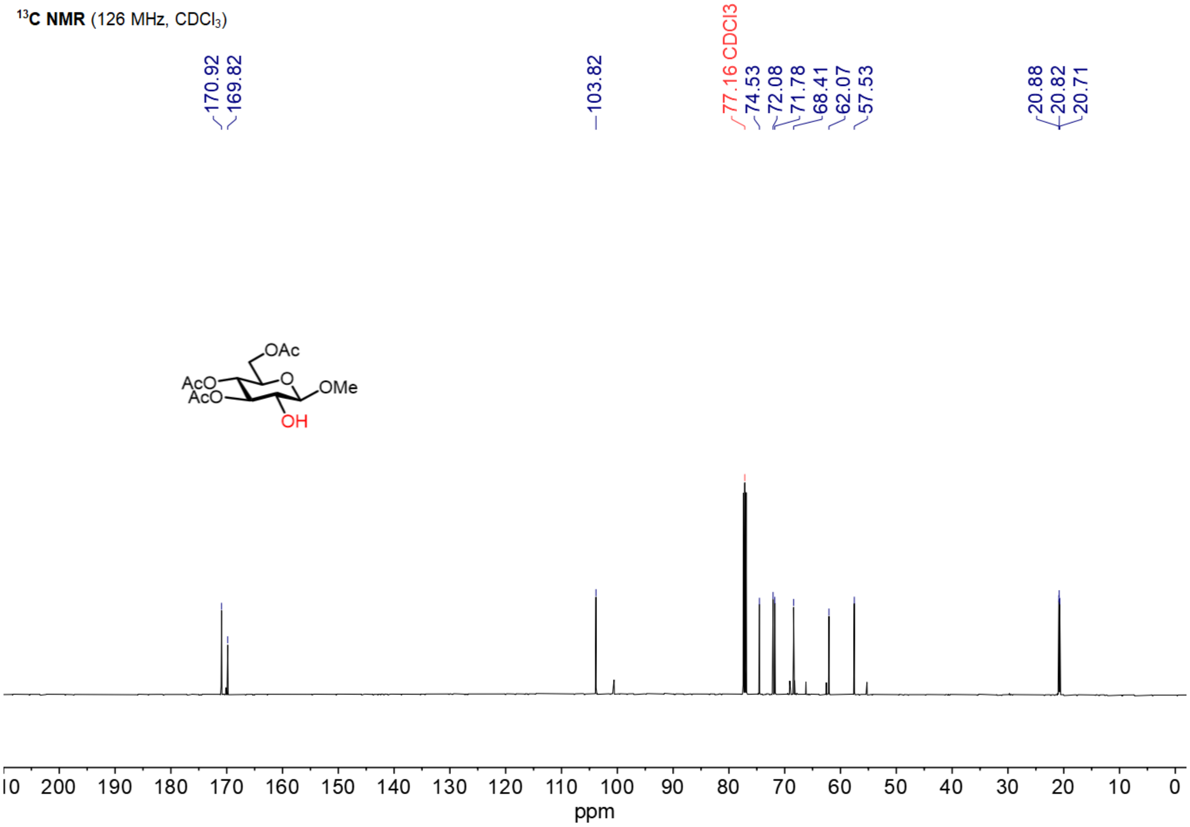

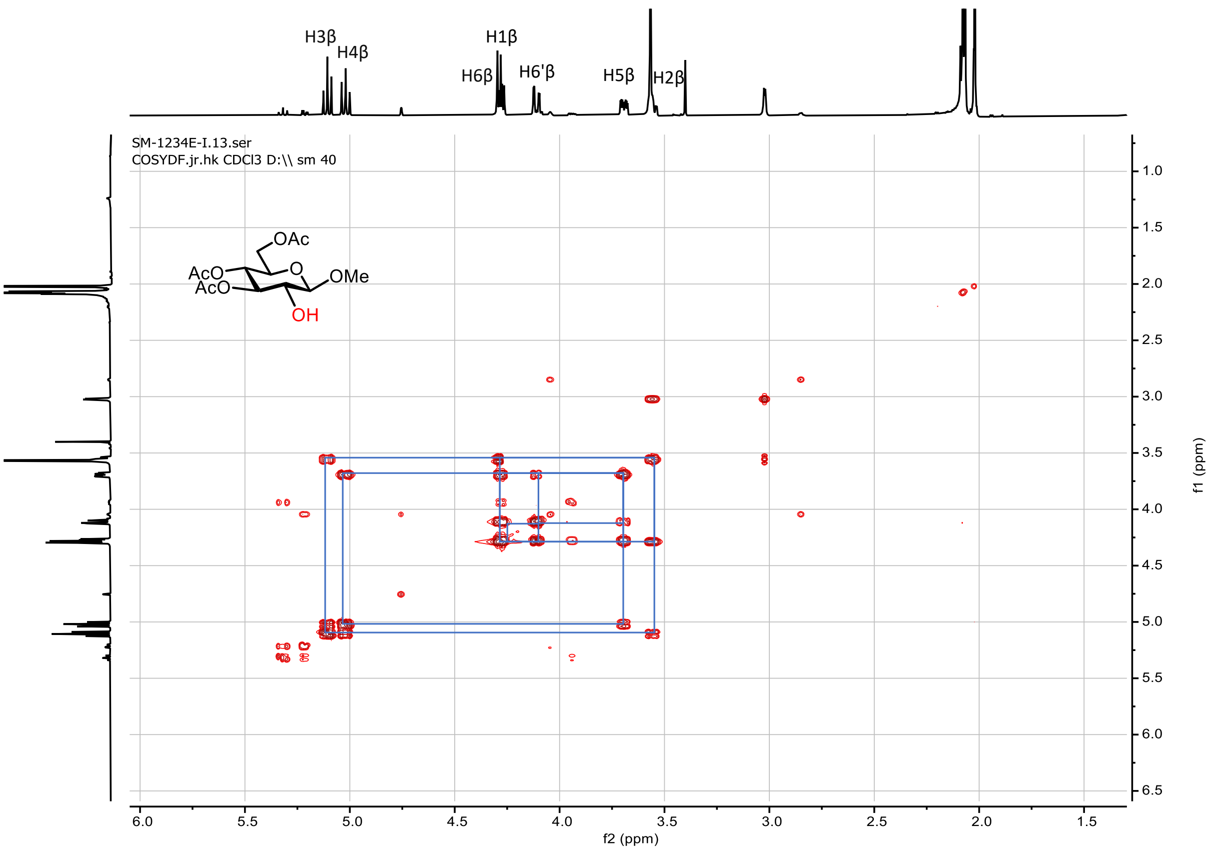
H3β
H4β
H1β
H6β
H6'β
H5β
H2β
SM-1234E-I.13.ser
COSYDF.jr.hk CDCl3 D:\\ sm 40
OAc
AcO
AcO
O
OMe
OH
f1 (ppm)
f2 (ppm)

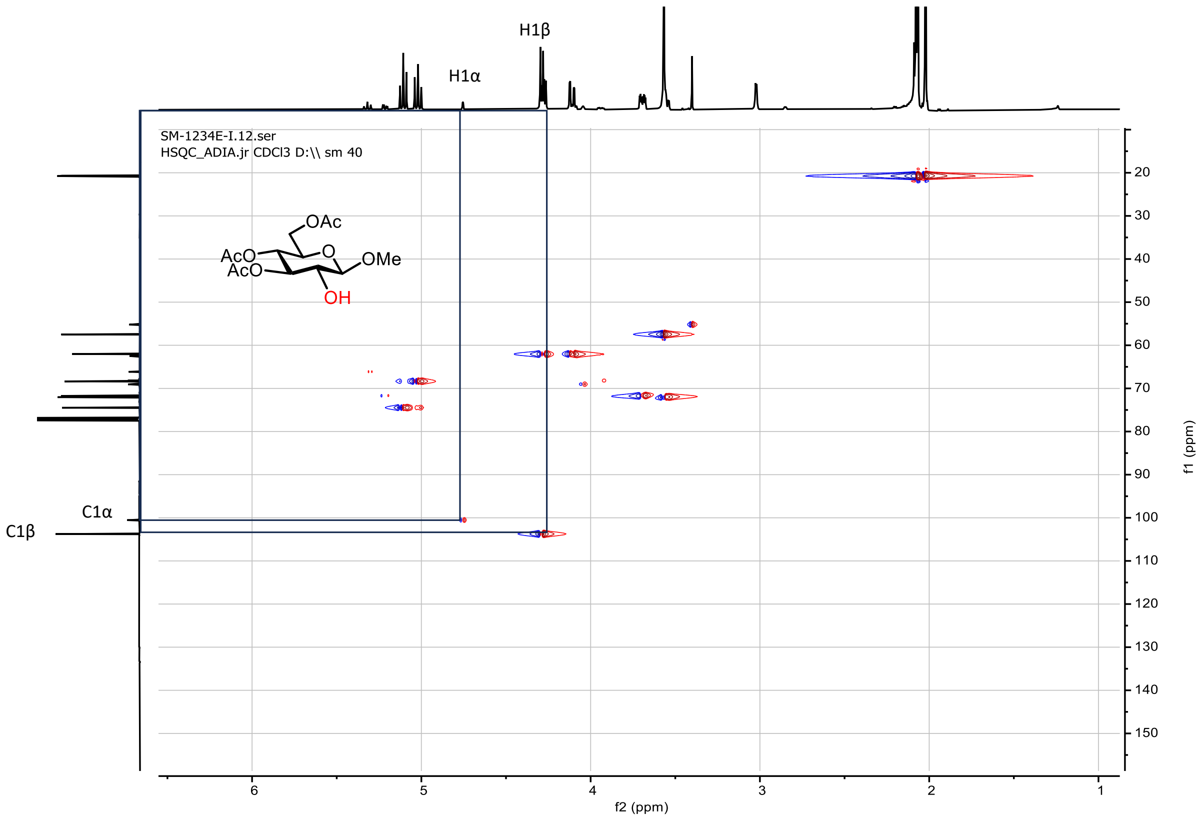
H1β
H1α
SM-1234E-I.12.ser
HSQC_ADIA.jr CDCl3 D:\\ sm 40
OAc
AcO
AcO
O
OMe
OH
C1α
C1β
f1 (ppm)
f2 (ppm)

Methyl 2-hydroxy-3,4,6-tri-O-benzoyl-β-D-galactopyranoside:

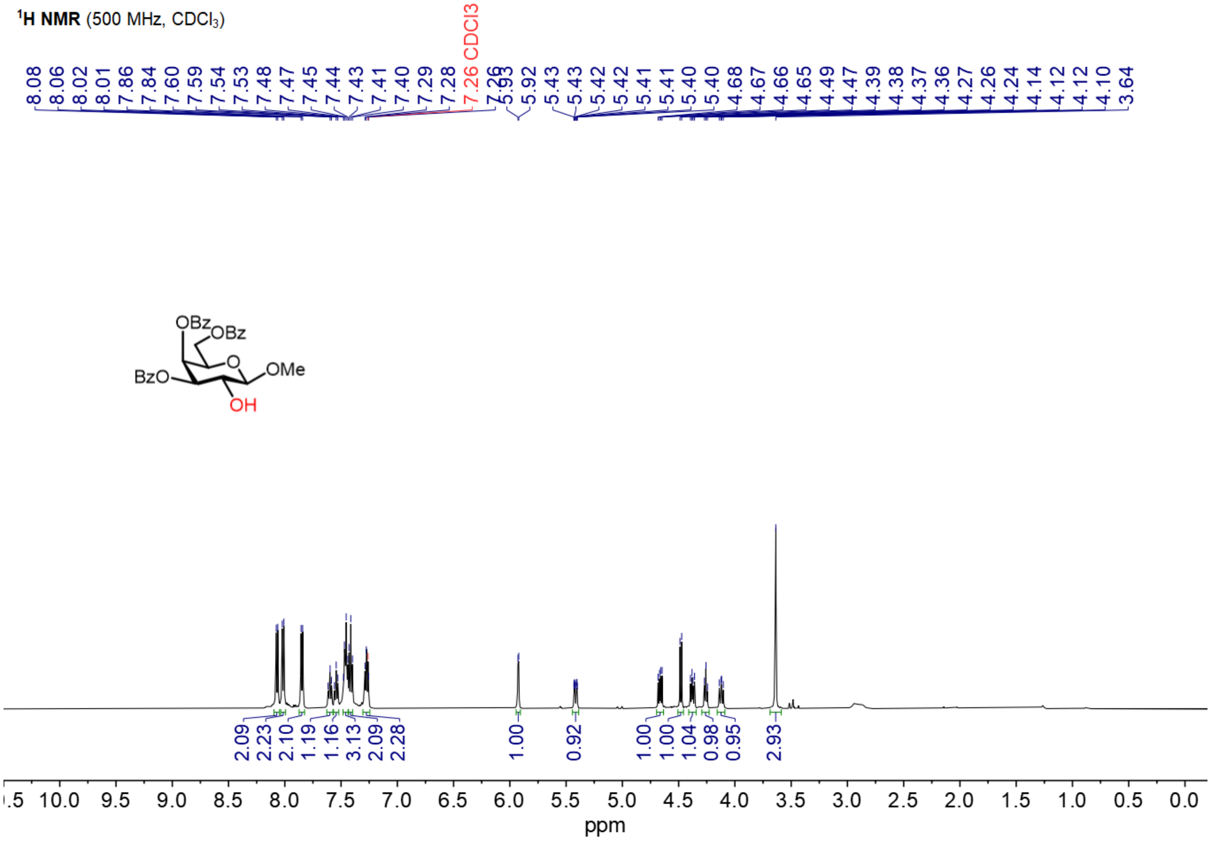

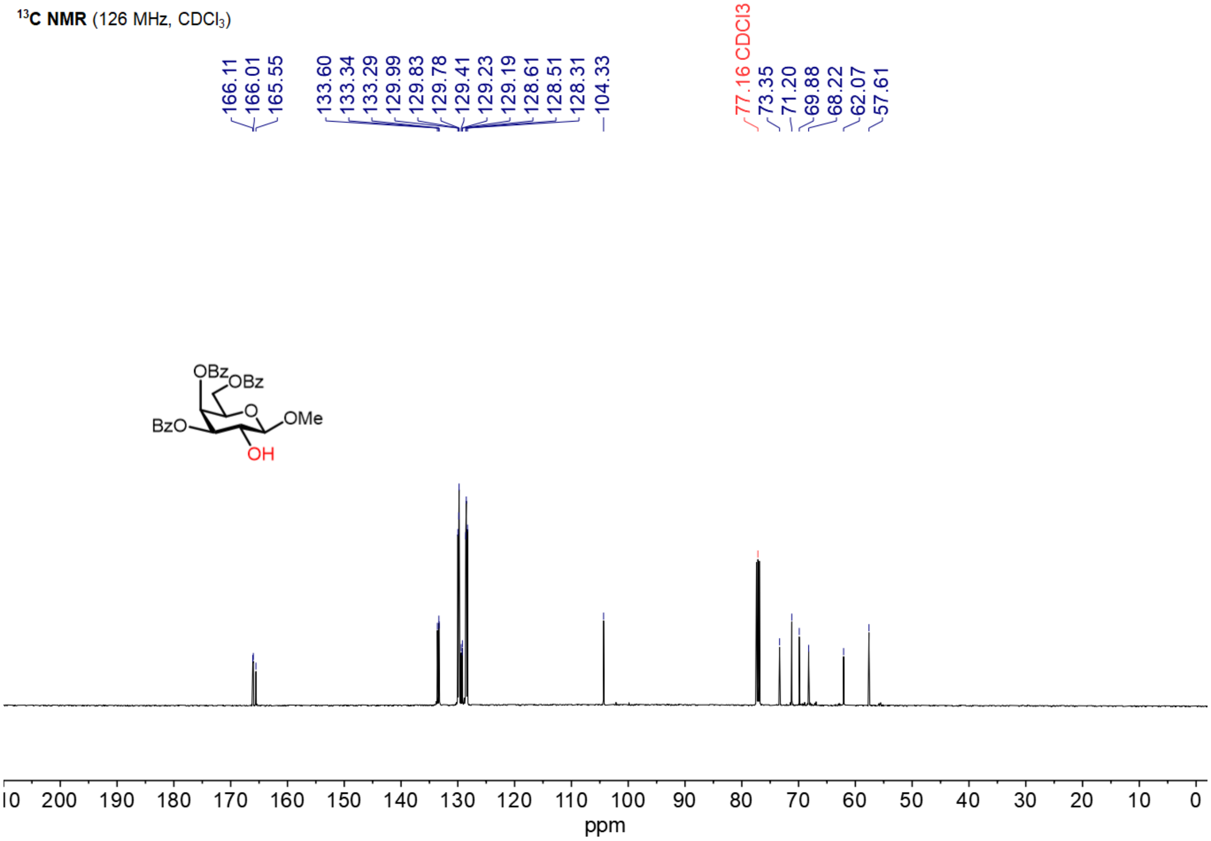

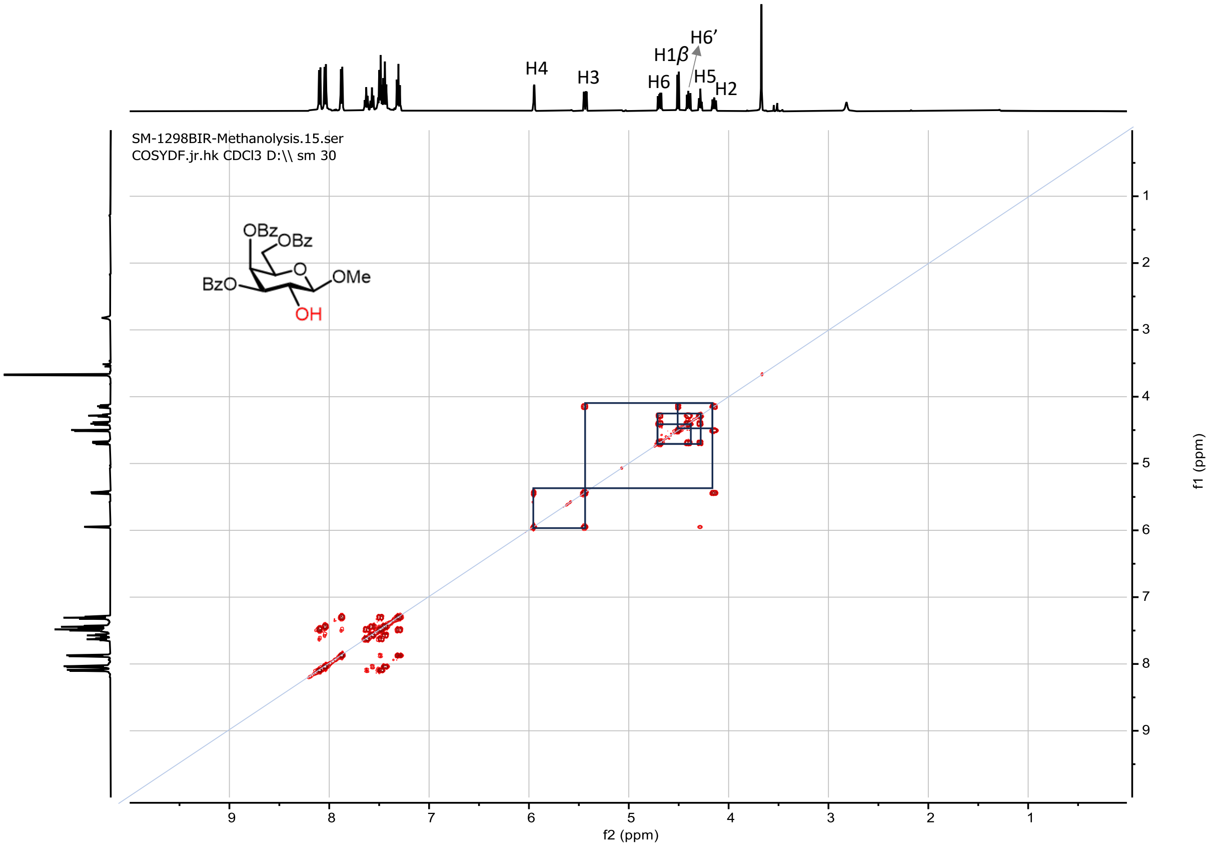
H4
H3
H1β
H6'
H6
H5
H2
SM-1298BIR-Methanolysis.15.ser
COSYDF.jr.hk CDCl3 D:\\ sm 30
OBz
OBz
BzO
O
OMe
OH
f2 (ppm)
f1 (ppm)

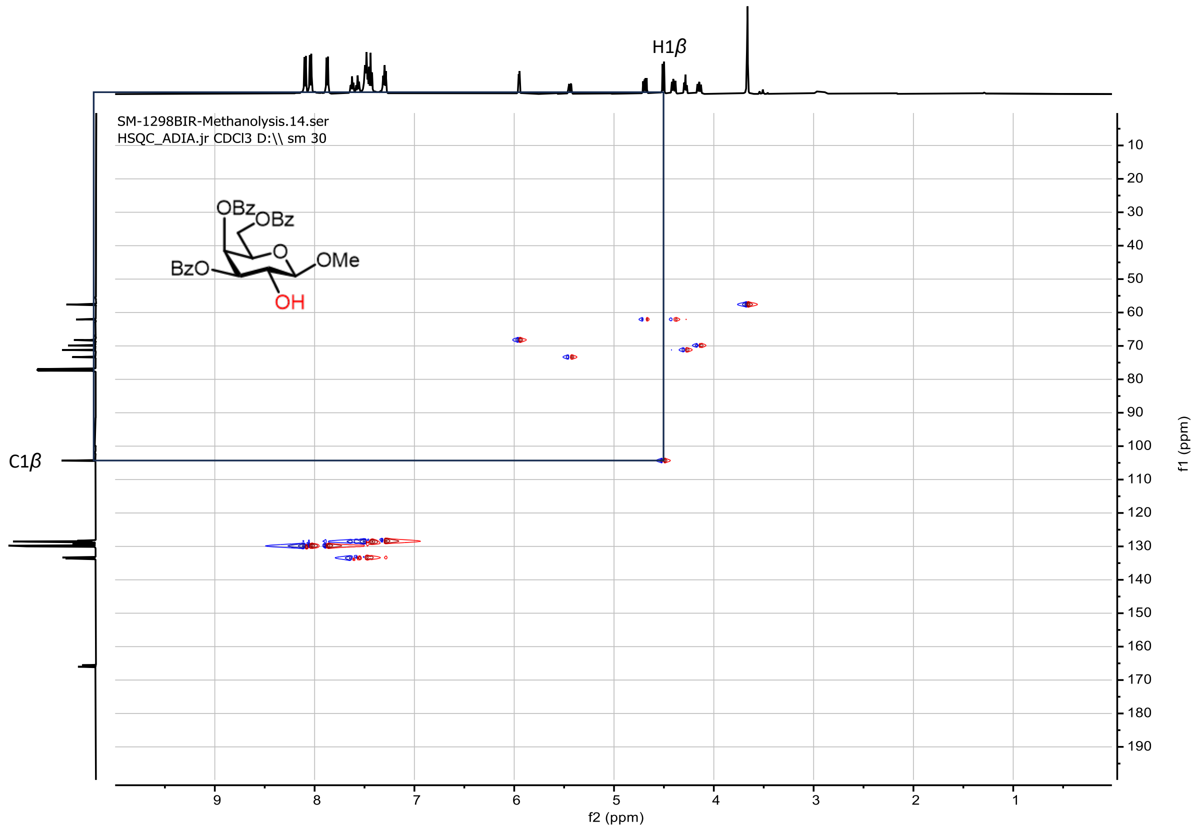
H1β
SM-1298BIR-Methanolysis.14.ser
HSQC_ADIA.jr CDCl3 D:\\ sm 30
OBz
OBz
BzO
O
OMe
OH
C1β
f2 (ppm)
f1 (ppm)
